# Global Variation in Out‐of‐Pocket Payments for Cancer Surgery

**DOI:** 10.1002/wjs.12637

**Published:** 2025-06-29

**Authors:** Joana F. F. Simoes, Maria Picciochi, Adesoji Ademuyiwa, Adewale Adisa, Theophilus Anyomih, Aneel Bhangu, Jose A. Calvache, Dhruva Ghosh, Kate Jolly, Mwayi Kachapila, Ismail Lawani, Dmitri Nepogodiev, Faustin Ntirenganya, Raymond Oppong, Stephen Tabiri, Antonio Ramos‐De la Medina, NIHR Global Health Research Unit on Global Surgery

**Affiliations:** ^1^ NIHR Global Health Research Unit on Global Surgery University of Birmingham Birmingham UK; ^2^ Faculty of Clinical Sciences College of Medicine University of Lagos Lagos Nigeria; ^3^ Department of Surgery Obafemi Awolowo University Ife‐Ife Nigeria; ^4^ Department of Anaesthesiology Universidad del Cauca Popayán Colombia; ^5^ Department of Anaesthesiology Erasmus MC Rotterdam Rotterdam the Netherlands; ^6^ Department of Paediatric Surgery Christian Medical College Ludhiana India; ^7^ School of Health Sciences University of Birmingham Birmingham UK; ^8^ Health Economics Unit Department of Applied Health Sciences University of Birmingham Birmingham UK; ^9^ Faculty of Health Sciences of Cotonou University of Abomey‐Calavi Cotonou Benin; ^10^ Department of Surgery CMHS ‐ University of Rwanda Butare Rwanda; ^11^ University for Development Studies, School of Medicine Tamale Teaching Hospital Tamale Ghana; ^12^ Hospital Espanol de Veracruz Veracruz Mexico

**Keywords:** cancer, catastrophic expenditure, global surgery

## Abstract

**Introduction:**

Cancer is one of the most expensive global health challenges and surgery is needed in most cases. This study aimed to describe out‐of‐pocket payments for cancer surgery across country income groups.

**Methods:**

This was a preplanned secondary analysis from an international prospective cohort study of consecutive patients undergoing cancer surgery in October 2020. Out‐of‐pocket payments (OOPP) to cover most of the cost of cancer surgery were compared across country income groups. Other funding sources were also described as follows: public funds, insurance, or others. A logistic regression model was used to identify variables independently associated with OOPP in low‐ and middle‐income countries.

**Results:**

There were 24,498 patients included from 1332 hospitals from 108 countries. Overall, 6.4% (1571/24,498) had OOPP to cover most of the cost of their cancer surgery. OOPP rates varied across country income groups: 0.5% (89/16,680) in HICs, 5.7% (272/4784) in UMICs, 38.6% (1008/2614) in LMICs, and 48.1% (202/429) in LICs. Besides the country income, male sex (OR 1.16, 95% CI 1.02–1.32, and *p* = 0.024) and elective surgery (OR 1.31, 1.04–1.67, and *p* = 0.022) were associated with OOPP for cancer surgery.

**Discussion:**

Patients accessing cancer surgery in LMICs are at an increased risk of catastrophic expenditure. Governments should prevent this by developing health insurance plans that cover elective cancer surgery, possibly involving diverse stakeholders. The interpretation of gender‐related risks demands deeper understanding of the ability to pay out‐of‐pocket and access care.

## Graphical abstract

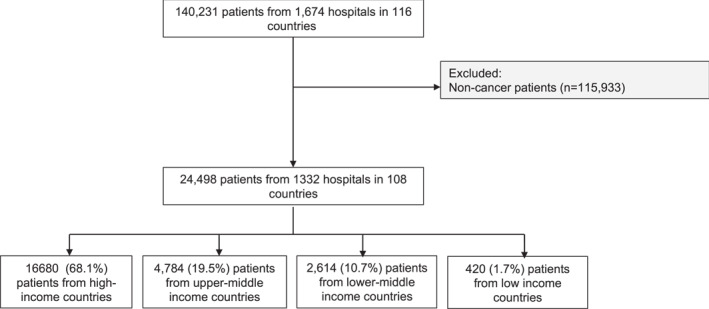

## 1 Introduction

Achieving universal health coverage (UHC) implies providing the necessary healthcare to all individuals while protecting them from financial hardship [1]. Monitoring global and country‐level UHC requires measuring out‐of‐pocket payments and their consequences in the form of catastrophic expenditure (OOPP for healthcare above a threshold of the household income) and impoverishing expenditure (OOPP for healthcare pushing households below the poverty line) [2]. The global rate of catastrophic health expenditure is 13.2% of the world's population, meaning that 996 million people suffer from it. This makes patients avoid seeking care when they need it and is a recognized barrier to achieving UHC [3, 4].

In the next decade, noncommunicable diseases, where cancer is included, are expected to increase and be the leading mortality cause [5]. Therefore, anticipation and planning to protect patients that will need to receive care for it needs to happen now to ensure that achieving UHC is on track. Looking in more detail at variation and finance of cancer care, solid tumors are the most common and will need surgery to be cured [6, 7]. Catastrophic expenditure attributable to accessing surgical care has been estimated to affect 81.2 million people, and 3.7 billion people are expected to be at a risk of catastrophic expenditure if they require surgical and anesthetic care [8, 9]. Although these estimates were modeled, that is the only data at a global level available. Smaller scale studies evaluated catastrophic expenditure but they were in other diseases or focused on a single disease or a single country [10, 11, 12].

The last WHO report on financial protection showed that catastrophic health expenditure rates were highest in middle‐income countries, whereas impoverishing expenditure was highest in lower‐middle and low‐income countries [3]. The variation of OOPP for surgical cancer care across income settings is unknown, but previous modeling studies highlighted higher rates of OOPP in low and middle‐income countries [8]. The primary aim of this study was to describe out‐of‐pocket payments for cancer surgery across income country groups. Considering that OOPP rates were expected to be higher in LMICs, the secondary aim was to identify characteristics of patients and procedures associated with higher rates of OOPP in LMICs to inform decision‐making in these settings.

## 2 Methods

### 2.1 Study Design and Setting

This study was a preplanned analysis of the SurgWeek, a prospective international cohort study [13]. Any hospital where surgery was performed was eligible to take part. Surgery was defined as any procedure routinely performed by a surgeon in an operating theater. The study followed the principles of collaborative research and was led by the NIHR Global Health Research Unit on Global Surgery, at the University of Birmingham, as described elsewhere [13, 14]. Data were collected from all consecutive patients undergoing any type of surgery for a minimum period of 1 week and a maximum of 4 weeks during October 2020. Follow‐up data were collected 30 days after surgery, from clinical records whenever possible or by direct patient assessment if this best met local treatment pathways.

### 2.2 Study Approvals

The study was registered on clinicaltrials.gov (registration number NCT04509986) and approvals were obtained as per the country level and local regulations. Being an observational cohort study without changes to normal care pathways, some countries did not require an ethical approval (e.g., approved as a clinical audit in the United Kingdom), some required only a local ethical approval at the hospital level (e.g., Portugal and Spain), and others required the national level approval (e.g., Australia, Sweden, Brazil, and India). National leads oversaw the national approval submission whenever needed, and hospital leads had the ultimate responsibility of ensuring the necessary approvals were granted at the time of data entry. Informed consent for data collection was obtained whenever required according to regulations in place.

### 2.3 Study Participants

All consecutive patients undergoing elective or emergency operations for cancer were included in this analysis. Both adults and children were included. Surgery as day‐case or as inpatient were also included. Procedures that were performed for diagnostic purposes only (e.g., breast biopsy) and endoscopic procedures (e.g., colonoscopy) were excluded and a full list is available in Supporting Information S1: Table 1. These were excluded considering were either classified as minor and/or not routinely performed in theater by a surgeon at a global level.

### 2.4 Data Variables and Definitions

Data were collected on patient characteristics, surgical characteristics, and payment types. Patient characteristics included age at the time of surgery, which was stratified into < 40 years, 40 to 69, and 70 or above, sex, preoperative risk according to the American Society of Anesthesiologists physical status (ASA), and perioperative cardiac risk assessment as per the revised cardiac risk index (RCRI), which is shown in Supporting Information S1: Table 2. Surgical characteristics included urgency of surgery, Bupa grades, which included major and minor/intermediate, surgical intent (curative or palliative), surgical approach and surgical specialty. Payment types included the funding sources to pay for most of the cost of cancer surgery, including government funds (e.g., national health system), health insurance (prepaid by the patient or their employer), external funds, and grants or other providers.

### 2.5 Study Outcomes

The primary outcome of this study was out‐of‐pocket payments for cancer surgery, defined as most of the cost of surgery being paid out‐of‐pocket by patients. Out‐of‐pocket payments were defined as payments made directly by the patients at the time‐of‐service use that is at the time of the cancer surgery. The cost of cancer surgery included the cost of the infrastructure use, staff time, and other items necessary to deliver surgical care during the admission for surgery. Nonmedical (travel, accommodation, and food), indirect costs (income loss), and costs of subsequent admissions were not included in this definition.

### 2.6 Statistical Analysis

All categorical data were presented with absolute frequencies and percentages. Ninety‐five percent CIs of the main outcome (OOPP) were given for the different income groups. This dataset did not include any continuous variables. To test associations between patient characteristics and OOPP, an unadjusted analysis was performed with chi‐squared tests. To adjust it, a random effects logistic regression model was used. This model looked at OOPP as an outcome and included prespecified patient, disease, and setting features that could be influencing the fact that the outcome was observed in a particular patient. The subgroup analyses of the most common cancers included were conducted. All data were handled and analyzed in *R studio,* statistical software (version 4.1.1).

## 3 Results

**FIGURE 1:**
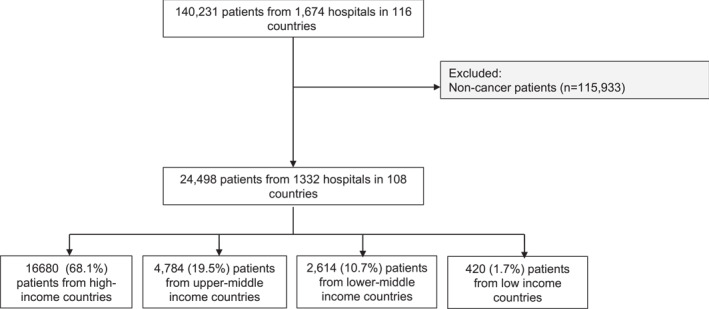
Flowchart of included patients.

A total of 24,498 patients with cancer were included from 1332 hospitals in 108 countries. Data were collected from all country income groups, with 68.1% (16,680/24,498) being from high‐income countries, 19.5% (4784/24,498) from upper‐middle income countries, 10.7% (2614/24,498) from lower‐middle income countries, and 1.7% (420/24,498) from low‐income countries. The number of patients and hospitals by country income groups is shown in Figure 1. The highest recruiting countries per income group were as follows: United Kingdom, Italy, and the United States of America (high‐income group); Turkey, Brazil, and Colombia (upper‐middle income group); India, Egypt, and Pakistan (lower‐middle income group); and Ethiopia, Syria, and Sudan (low‐income group) as shown in Supporting Information S1: Table 3.

Most of the patients were adults, aged between 40 and 69 years old (57.8% [14,173/24,498]). Approximately half of the patients were female (54.5% [13,355/24,498]). Patients with ASA grades I–II were more common (64.9% [15,909/24,498]). There were more patients with a revised cardiac risk index of one to two (15,256 patients [62.3%]) than other levels (Table 1). Patients being treated in low‐middle‐income countries were distinct from those from HICs. Patients with cancer surgery in LMICs were younger (18.4% vs. 7% were aged under 40 years old), had fewer comorbidities (75.5% vs. 60.0% were ASA grade 1–2), had a higher number of palliative (14.9% vs. 9%), and had open surgeries (77.8% vs. 59.1%) as fully described in Table 1.

**TABLE 1:** Patient and surgery characteristics in patients from HICs and LMICs.

	HICs (*n* = 16,680)	LMICs (*n* = 7818)
Age groups		
< 40 years	1165 (7.0%)	1440 (18.4%)
40–69 years	9111 (54.6%)	5062 (64.7%)
> 70 years	6404 (38.4%)	1316 (16.8%)
Sex		
Female	8774 (52.6%)	4581 (58.6%)
Male	7906 (47.4%)	3237 (41.4%)
ASA grade		
Grades 1–2	10,008 (60.0%)	5901 (75.5%)
Grades 3–5	6663 (40.0%)	1917 (24.5%)
Cardiac risk (RCRI)		
0	5335 (32.0%)	3097 (39.6%)
1–2	10,755 (64.5%)	4501 (57.6%)
≥ 3	585 (3.5%)	218 (2.8%)
Missing	1	2
Urgency		
Emergency	1205 (7.2%)	712 (9.1%)
Elective	15,474 (92.8%)	7106 (90.9%)
Missing	1	0
Surgical grade		
Major	11,779 (70.6%)	6089 (77.9%)
Minor/Intermediate	4900 (29.4%)	1727 (22.1%)
Missing	1	2
Intent of surgery		
Curative or diagnostic	15,160 (91.0%)	6643 (85.1%)
Palliative	1507 (9.0%)	1165 (14.9%)
Missing	13	10
Operative approach		
Open	9861 (59.1%)	6085 (77.8%)
Minimally invasive	6219 (37.3%)	1497 (19.2%)
Hybrid	198 (1.2%)	87 (1.1%)
Converted to open	401 (2.4%)	148 (1.9%)
Missing	1	1
Specialty		
Breast surgery	2481 (14.9%)	1513 (19.4%)
Cardiac surgery	23 (0.1%)	6 (0.1%)
Colorectal surgery	3179 (19.1%)	1397 (17.9%)
Dentistry	4 (0.0%)	2 (0.0%)
Endocrine surgery	554 (3.3%)	478 (6.1%)
General surgery	831 (5.0%)	355 (4.5%)
Gynecology	1133 (6.8%)	625 (8.0%)
Head and neck surgery	1248 (7.5%)	693 (8.9%)
Hepatobiliary surgery	1131 (6.8%)	384 (4.9%)
Neurosurgery	515 (3.1%)	310 (4.0%)
Esophagogastric surgery	588 (3.5%)	333 (4.3%)
Ophthalmology	67 (0.4%)	31 (0.4%)
Orthopedics	183 (1.1%)	144 (1.8%)
Other procedures	358 (2.1%)	129 (1.7%)
Plastic surgery	914 (5.5%)	257 (3.3%)
Spinal surgery	134 (0.8%)	47 (0.6%)
Thoracic surgery	1077 (6.5%)	215 (2.8%)
Urology	2215 (13.3%)	884 (11.3%)
Vascular surgery	44 (0.3%)	13 (0.2%)
Missing	1	2

*Note:* Data presented as *n* (%).

Abbreviation: RCRI, revised cardiac risk index.

The most common type of cancer was colorectal cancer (4576/24,498 [18.7%]), followed by breast (3994/24,498 [16.3%]) and urological cancers (3099/24,498 [12.7%]). Most included patients underwent elective surgeries (92.2% [22,580/24,498]) and major procedures (72.9% [17,868/24,498]). The intent of the surgery was curative or diagnostic in 89.0% of the patients (21,803/24,498) and palliative in 11.0% (2672/24,498). The operative approach was planned and performed as open in 65.1% of the patients (15,946/24,498) as shown in Table 2.

**TABLE 2:** Number of patients with cancer by surgical specialty, with urgency of surgery, surgical grade, and surgical intent by specialty.

Surgical specialty	Urgency of surgery		Surgical grade		Surgical intent		Total
	Emergency	Elective	Major	Minor/Intermediate	Curative or diagnostic	Palliative	
Colorectal	864 (18.9)	3712 (81.1)	4466 (97.6)	110 (2.4)	3718 (81.3)	855 (18.7)	4573
Breast	21 (0.5)	3973 (99.5)	2445 (61.2)	1549 (38.8)	3916 (98.1)	75 (1.9)	3991
Urology	125 (4.0)	2974 (96.0)	1540 (49.7)	1559 (50.3)	2726 (88.0)	371 (12.0)	3097
Head and neck	100 (5.2)	1841 (94.8)	1124 (57.9)	817 (42.1)	1821 (94.0)	117 (6.0)	1938
Gynecology	30 (1.7)	1728 (98.3)	1556 (88.5)	202 (11.5)	1652 (94.0)	106 (6.0)	1758
Hepatobiliary	62 (4.1)	1453 (95.9)	1513 (99.9)	2 (0.1)	1374 (90.8)	139 (9.2)	1513
Thoracic	52 (4.0)	1240 (96.0)	1255 (97.1)	37 (2.9)	1146 (88.8)	144 (11.2)	1290
General	213 (18.0)	973 (82.0)	523 (44.1)	663 (55.9)	986 (83.2)	199 (16.8)	1185
Plastic	11 (0.9)	1160 (99.1)	72 (6.1)	1099 (93.9)	1130 (96.5)	41 (3.5)	1171
Endocrine	9 (0.9)	1023 (99.1)	1032 (100.0)	0 (0.0)	1004 (97.5)	26 (2.5)	1030
Esophagogastric	76 (8.3)	844 (91.7)	917 (99.6)	4 (0.4)	770 (83.6)	151 (16.4)	921
Neurosurgery	186 (22.5)	639 (77.5)	825 (100.0)	0 (0.0)	623 (75.8)	199 (24.2)	822
Other	29 (6.0)	458 (94.0)	130 (26.7)	357 (73.3)	461 (94.7)	26 (5.3)	487
Orthopedics	49 (15.0)	278 (85.0)	207 (63.3)	120 (36.7)	218 (66.7)	109 (33.3)	327
Spinal	61 (33.7)	120 (66.3)	180 (99.4)	1 (0.6)	92 (51.1)	88 (48.9)	180
Ophthalmology	13 (13.3)	85 (86.7)	0 (0.0)	98 (100.0)	93 (94.9)	5 (5.1)	98
Vascular	9 (15.8)	48 (84.2)	54 (94.7)	3 (5.3)	43 (75.4)	14 (24.6)	57
Cardiac	6 (20.7)	23 (79.3)	29 (100.0)	0 (0.0)	23 (79.3)	6 (20.7)	29
Dentistry	0 (0.0)	6 (100.0)	0 (0.0)	6 (100.0)	5 (83.3)	1 (16.7)	6

*Note:* Data presented as *n* (). Missing data: surgical specialty *n* = 3; urgency of surgery *n* = 1; surgical grade *n* = 3; surgical intent *n* = 23. Percentages given by row to represent % of emergent, elective, curative, and palliative surgery by surgical specialty.

**FIGURE 2:**
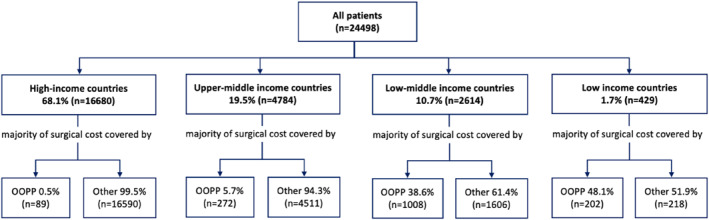
Rates of out‐of‐pocket payments to pay for most of the cancer surgery cost by the country income group. OOPP, out of pocket payments.

**FIGURE 3:**
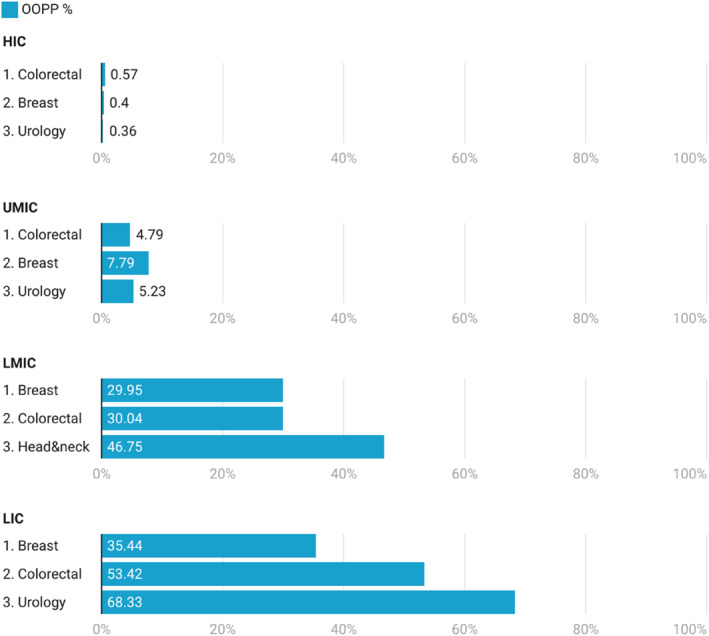
OOPP rates for the three most common types of surgery across country income groups. Missing data for the method of payment *n* = 2 and for type of surgery *n* = 3. HICs, high‐income countries; LICs, low‐income countries; LMICs, lower‐middle‐income countries; UMICs, upper‐middle‐income countries.

From all the patients included in the study, 6.4% (1571/24,498, 95% CI 6.1%–6.7%) paid out‐of‐pocket payments (OOPP) for most of their cancer surgery. The proportion of surgeries where most of the payment was OOPP was different across income settings, being 0.5% (89/16,680, 95% CI 0.4%–0.6%) in high‐income countries, 5.7% (272/4784, 95% CI 5.1%–6.4%) in upper‐middle income, 38.6% (1008/2614, 95% CI 36.7%–40.5%) in lower‐middle income, and 48.1% (202/429, 95% CI 42.3%–51.9%) in low‐income (Figure 2). The rates of OOPP across the three most common surgery types in each income setting also varied but were always higher in lower‐income settings as shown in Figure 3. The rates of OOPP were consistently higher in LMICs regardless the urgency and the intent of the procedures. OOPP were consistently higher in elective procedures across all income groups as shown in Supporting Information S1: Figure 1.

**FIGURE 4:**
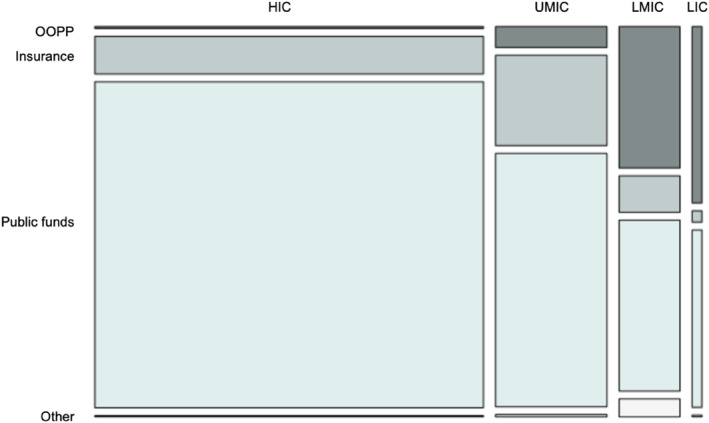
Mosaic plot of funding sources to pay for the most of the cost of cancer surgery by the country income group. This chart displays the proportion of patients within each setting who pay for most of their cancer surgery through OOPP, insurance, public funds, or other funding sources. Actual percentages are shown in the previous flowchart (Figure 16). Missing data for the method of payment *n* = 2. HICs, high‐income countries; LICs, low‐income countries; LMICs, low and middle‐income countries; UMICs, upper‐middle‐income countries.

Other funding sources were used to pay most of the cancer surgery, with public or government funds being the most common at the global level (79.8% [19,541/24,498]), followed by insurance (12.9% [3164/24,498]) and other providers (0.9% [220/24,498]). The use of these public and insurance funds to pay for cancer surgery was variable across income groups, with high‐income countries having the highest use of public funds (87.8% [14,646/16,680]) and upper‐middle income countries using insurance more commonly (24.2% [1158/4784]) as shown in Figure 4 and Supporting Information S1: Figure 2.

Of the 7818 patients included from low‐ and middle‐income countries, 19.0% (1482/7818) paid out‐of‐pocket for most of their cancer surgery cost. The rates of OOPP based on cancer type and per country are summarized in Supporting Information S1: Figure 3 and Supporting Information S1: Table 4, respectively. The OOPP rates were higher in young patients (24% in under 40 years old, *p* < 0.001) and with fewer comorbidities (20.2% for ASA grades 1–2, *p* < 0.001) as shown in Table 3. Urgency of surgery and surgical grade were not associated with higher OOPP rates.

**TABLE 3:** Patient and surgical features associated with out‐of‐pocket payments for most of the cost of surgery in LMICs (unadjusted analysis).

	Non‐OOPP	OOPP	*p* value
Age			
< 40 years	1094 (76.0%)	346 (24.0%)	< 0.001
40–69 years	4108 (81.2%)	953 (18.8%)	
> 70 years	1133 (86.1%)	183 (13.9%)	
Sex			
Female	3750 (81.9%)	830 (18.1%)	0.027
Male	2585 (79.9%)	652 (20.1%)	
ASA grade			
Grades 1–2	4708 (79.8%)	1192 (20.2%)	< 0.001
Grades 3–5	1627 (84.9%)	290 (15.1%)	
Cardiac risk (RCRI)			
0	2472 (79.8%)	625 (20.2%)	0.014
1–2	3673 (81.6%)	827 (18.4%)	
≥ 3	189 (86.7%)	29 (13.3%)	
Missing	1	1	
Urgency			
Emergency	584 (82.0%)	128 (18.0%)	0.515
Elective	5751 (80.9%)	1354 (19.1%)	
Surgical grade			
Major	4934 (81.0%)	1154 (19.0%)	1.000
Minor/Intermediate	1400 (81.1%)	327 (18.9%)	
Missing	1	1	
Intent			
Curative or diagnostic	5421 (81.6%)	1221 (18.4%)	0.002
Palliative	906 (77.8%)	259 (22.2%)	
Missing	8	2	
Operative approach			
Open	4892 (80.4%)	1192 (19.6%)	0.022
Minimally invasive	1240 (82.8%)	257 (17.2%)	
Hybrid	73 (83.9%)	14 (16.1%)	
Converted to open	130 (87.8%)	18 (12.2%)	
Missing	0	1	

*Note:* Data presented as *n* (%).

Abbreviation: RCRI, revised cardiac risk index.

**FIGURE 5:**
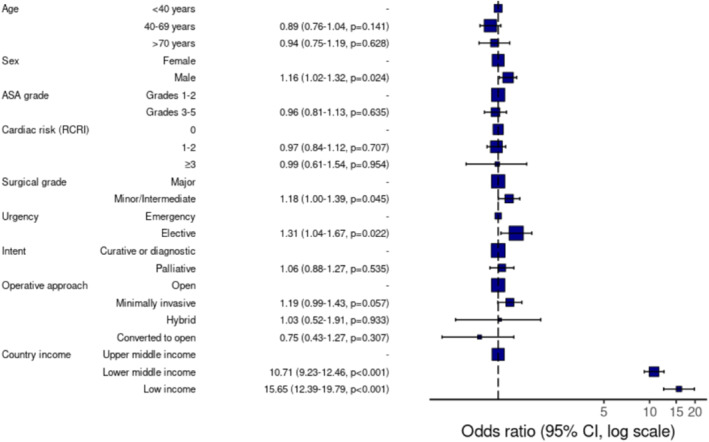
Forest plot of the logistic regression model of characteristics associated with OOPP for most of the cancer surgery cost. Model metrics: Number in data frame = 7818, number in model = 7804, missing = 14, AIC = 6144.9, C‐statistic = 0.783, and H&L = chi‐squared (8) 16.20 (*p* = 0.040). RCRI, revised cardiac risk index.

After adjustment, the characteristics independently associated with OOPP for most of the cost of cancer surgery were male sex (OR 1.16, 95% CI 1.02–1.32, *p* = 0.024), elective surgery (OR 1.31, 1.04–1.67, and *p* = 0.022), and lower country income groups (OR 10.71, 95% CI 9.23–12.46, and *p* < 0.001 for lower‐middle income countries and OR 15.65, 95% CI 12.39–19.79, and *p* < 0.001 for low‐income countries compared to upper‐middle‐income countries), which is shown in Figure 5 and Supporting Information S1: Table 4.

The subgroup analyses of patients with breast cancer and of patients with colorectal cancer showed that the only characteristic independently associated with OOPP for most of the cost of cancer surgery was the income group as fully described on Supporting Information S1: Tables 5–6.

## 4 Discussion

This study showed that patients with cancer in low‐ and middle‐income countries were more likely to pay out‐of‐pocket for most of the cost of surgery. Country income was associated with OOPP for cancer surgery in all analyses and subgroups, suggesting that patients who live in economically challenged contexts lack financial protection more often. These findings are particularly important to low and middle‐income countries with high representation in this study (India, Turkey, Brazil, Colombia, and Egypt).

The distribution of prepaid mechanisms used across income groups to pay for cancer surgery is likely to reflect the organization of their healthcare systems. Public funds are more often used in high‐income countries, where national health systems funded through taxes are more common and where the healthcare expenditure is higher, for example in the United Kingdom (12.16% of GDP) [15]. Although publicly funded healthcare systems have their own limitations, particularly when responding to a high volume of patients, they seem to be one of the most reliable ways to avoid catastrophic expenditure [3]. The work developed by the national surgical, obstetric, and anesthesia plans tried to provide a roadmap to achieve this in low and middle‐income countries [16]. Upper‐middle countries had the highest rate of insurance use, reflecting the well‐developed private health sector [17], insurance initiatives, and lower health expenditure, which is 3.34% of GDP in India for instance [15, 18].

Male sex and elective surgery remained significantly associated with OOPP after adjustment, with potential implications for healthcare policy. This supports advocacy for more investment in financing elective care, but the ratio of elective/emergency surgery in this study (more than 90% elective) makes this finding difficult to interpret. A possible selection bias needs to be acknowledged as patients requiring emergency surgery could have been treated more often in nonparticipating hospitals, being underrepresented in this study. It is also possible that patients requiring emergency surgery were more frequently from disadvantaged backgrounds, with higher odds of advanced disease, and might have struggled to access surgical treatment, therefore being out of the study [12, 19]. Furthermore, WHO resolutions influence government resolutions and these have been more focused on emergency care, which might have led to financial protection initiatives that include this group of patients and not elective surgery patients.

Similarly, careful considerations need to be drawn from the fact that men pay more frequently out‐of‐pocket for most of the cost of their cancer surgery. This result can reflect that men have a higher income or more disposable money. This is in line with previous studies where gender disparities in healthcare financing were explored [20], showing that men were likely to incur higher healthcare expenses and women had less access to healthcare and therefore would experience less financial burden [10, 21, 22].

Decision‐making on the types of surgery that require more financial support going forwards needs to balance OOPP rates but also the burden of the disease and how common surgery is required. For example, OOPP rates were similar in the most commonly performed surgeries (breast, colorectal, and urology), which supports these for higher public investment. The fact that these were the most common procedures in this study is a result of the incidence of cancer types but also the surgical case mix of the participating hospitals network, which can be different at whole population levels.

To our knowledge, there is no previous study reporting out‐of‐pocket payments for surgical care in more than 100 countries, likely because this is difficult to collect at a global scale. The findings of this study are widely applicable given that it captured data from a broad range of clinical settings. Although it can introduce heterogeneity, the fact that several types of cancers were included allows wide generalizability of findings, making these data more useful to advocate for additional funding toward multiple cancers [7].

Several limitations need to be acknowledged in this study. The main limitation is that although the study sample size is large, the granularity of the data are low, limiting the conclusions that can be drawn from it. More details on the type of disease (e.g., stage), costs of treatment parts (e.g., theater costs and admission costs), and money spent by the patients while paying out‐of‐pocket and socioeconomic level of patients would be useful to identify the most vulnerable subgroups of patients or types of treatment benefiting from the investment. Secondly, the inclusion criteria constitute a selection bias in itself, as patients requiring surgery but unable to afford or access it were not included in the study and might be the most disadvantaged of all. The fact that most patients underwent elective and curative surgery probably reflects the profile of patients being treated in the participating hospitals, introducing an inevitable selection bias that is commonly seen in global studies [23, 24]. Thirdly, the representation of low‐income settings in this study is not optimal, with fewer patients being included as country income decreased. The lower participation from low‐income countries might have produced a stronger association than the one seen in practice. This poses limitations in the practical application of the findings and further studies should be focused in this group of countries.

The methodological decision to exclude HIC patients from the models identifying predictors of OOPP aimed to avoid confounding, as the rate of OOPP in HICs was expected to be very low and patients accessing surgery to have quite different characteristics [12]. This could influence the results and make them less interpretable in the LMICs setting, where OOPP for cancer surgery were expected to be a larger problem.

The fact that the OOPP rates in this study were relatively low is probably related to the way that the data were collected. In theory, any OOPP can put households at risk of financial toxicity, depending on the scale of the OOPP compared to the household income. However, OOPP made for most of the cost of surgery are more likely to be high and have an impact on household finances, working as a better marker of financial burden than OOPP in general. Ideally, the total cost of treatment and household income would have been collected but this is very challenging at an international level, especially when the sources of funding for surgery are a secondary outcome of the main study. Additionally, this study did not include indirect nor opportunity costs of surgical care.

These findings highlight the need for action in policy in primary and secondary/tertiary care. At secondary and tertiary care levels, increased investment in prepaid mechanisms to protect patients with cancer from OOPP is needed, particularly in low‐income settings. Successful initiatives to protect patients from out‐of‐pocket payments need to be disseminated to accelerate patient protection [18] (e.g., insurance coverage in India promoted by Ayushman Bharat—Pradhan Mantri Jan Arogya Yojana plan [25]). Besides out‐of‐pocket payments, other studies have reported various financial distress strategies that can impact several generations (e.g., loans and property selling) [26].

Further research would provide more depth, as this study did not assess the consequences of the OOPP for households, a study exploring catastrophic expenditure rates, or financial hardship is needed to understand the real financial impact of these costs. Future studies including all patients with cancer eligible for treatments, rather than only the ones receiving it, would be able to capture the financial struggles of a wider group of patients and the impact on treatment completion.

Finally, health reforms are challenging in LMICs, where multiple providers have competing interests within the healthcare system and payment sources are usually not centralized in the government. Regulation of providers and reorganization of payment mechanisms is needed, with an initial focus on the poorest, which are at the highest risk of adverse health and economic outcomes. This has been done successfully in other countries, such as in India, with the Rashtriya Swasthya Bima Yojana plan [27]. Tax‐based or insurance‐based initiatives should be explored within each country, evaluating OOPP and catastrophic expenditure before and after implementation to inform the initiatives that might work best.

## Author Contributions

**Joana F. F. Simoes:** conceptualization, data curation, formal analysis, funding acquisition, investigation, methodology, project administration, resources, software, supervision, validation, visualization, writing – original draft, writing – review and editing. **Maria Picciochi:** investigation, methodology, project administration, visualization, writing – review and editing. **Adesoji Ademuyiwa:** writing – review and editing. **Adewale Adisa:** writing – review and editing. **Theophilus Anyomih:** writing – review and editing. **Aneel Bhangu:** writing – review and editing. **Jose A. Calvache:** writing – review and editing. **Dhruva Ghosh:** writing – review and editing. **Kate Jolly:** writing – review and editing. **Mwayi Kachapila:** writing – review and editing. **Ismail Lawani:** writing – review and editing. **Dmitri Nepogodiev:** writing – review and editing. **Faustin Ntirenganya:** writing – review and editing. **Raymond Oppong:** writing – review and editing. **Stephen Tabiri:** writing – review and editing. **Antonio Ramos‐De la Medina:** writing – review and editing. **NIHR Global Health Research Unit on Global Surgery:** conceptualization, funding acquisition, investigation, methodology, project administration, resources, software, supervision, validation, visualization, writing – original draft, writing – review and editing.

## Conflicts of Interest

The authors declare no conflicts of interest.

## Supporting information

Supporting Information S1

## Data Availability

Anonymized data are available upon request from the writing group, and successful completion of a data sharing agreement through an Application Programming Interface linked to the REDCap data server hosted at University of Birmingham, Birmingham, UK.
