# Supplementary material for: Global Variation in Out‐of‐Pocket Payments for Cancer Surgery
Source: World J Surg. 2025 Jun 29;49(8):2207–16. doi: 10.1002/wjs.12637 (PMC12338400; doi:10.1002/wjs.12637)
Supplement: Supplementary file 1 — Supporting Information S1 [file WJS-49-2207-s001.docx]

**Supplement**

**Global variation in out-of-pocket payments for cancer surgery**

NIHR Global Health Research Unit on Global Surgery

A full list of collaborating authors is shown in Section 1

**Corresponding author:**

**Keywords:** Global surgery, cancer, catastrophic expenditure.

**Funding:** This study was supported by NIHR Global Health Research Unit Grant (NIHR133364). The funders had no role in study design or writing of this report. The views expressed are those of the authors and not necessarily those of the National Health Service, the NIHR or the UK Department of Health and Social Care.

**Appendices**

| Section 1 – List of authors | Pages 1-82 |
| --- | --- |
| Section 2 – Supplementary tables and materials | Pages 83-88 |

**Section 1: List of authors (all co-authors are PubMed citable)**

**Writing group:**

Joana FF Simoes*, Maria Picciochi*, Adesoji Ademuyiwa, Adewale Adisa, Theophilus Anyomih, Aneel Bhangu, Jose A Calvache, Dhruva Ghosh, Kate Jolly, Mwayi Kachapila, Ismail Lawani, Dmitri Nepogodiev, Faustin Ntirenganya, Raymond Oppong, Stephen Tabiri, Antonio Ramos-De la Medina. (listed alphabetical)

*denotes joint first authors.

**Statistics:**

Joana FF Simoes, Omar M Omar.

#### **Operations Committee** (alphabetical):

| Waheed-Ul-Rahman Ahmed, Leah Argus, Alasdair Ball, Aneel Bhangu*, Edward P Bywater, Ruth Blanco-Colino, Amanpreet Brar, Daoud Chaudhry, Brett E Dawson, Irani Duran, Muhammed Elhadi, James C Glasbey, Rohan R Gujjuri, Conor S Jones, Ewen M Harrison, Sivesh K Kamarajah, James M Keatley, Samuel Lawday, Elizabeth Li, Harvinder Mann, Ella J Marson, Kenneth A Mclean, Dmitri Nepogodiev*, Lisa Norman, Riinu Ots, Oumaima Outani, Maria Picciochi, Irène Santos, Catherine Shaw, Joana FF Simoes, Elliott H Taylor, Isobel M Trout, Chris Varghese, Mary L Venn, William Xu.  *Co-chairs |
| --- |

#### **Dissemination Committee** (alphabetical by country):

| **Chair:** Joana FF Simoes; **Albania**: Irida Dajti, Arben Gjata; **Algeria**: Salah Eddine Oussama Kacimi; **Argentina**: Luis Boccalatte, Maria Marta Modolo; **Australia**: Daniel Cox, Peter Pockney, Philip Townend; **Austria**: Felix Aigner, Irmgard Elisabeth Kronberger; **Azerbaijan**: Elgun Samadov; **Bahrain**: Amer Alderazi; **Bangladesh**: Kamral Hossain; **Barbados**: Greg Padmore; **Belgium**: Gabrielle van Ramshorst; **Benin**: Ismaïl Lawani; **Bosnia and Herzegovina**: Anis Cerovac, Samir Delibegovic; **Brazil**: Glauco Baiocchi, Gustavo Mendonça Ataíde Gomes, Igor Lima Buarque; **Bulgaria**: Muhammad Gohar, Mihail Slavchev; **Cameroon**: Chukwuemeka Nwegbu; **Canada**: Arnav Agarwal, Amanpreet Brar, Janet Martin, Joshua Ng-Kamstra; **Chile**: Maria Marta Modolo, Maricarmen Olivos; **China**: Wenhui Lou, Dong-Lin Ren; **Colombia**: Jose Andres Calvache, Carlos J- Perez Rivera; **Croatia**: Ana Danic Hadzibegovic, Tomislav Kopjar, Jakov Mihanovic; **Cuba**: Pablo Mijahil Avilés Jiménez; **Cyprus**: Nikolaos Gouvas; **Czech Republic**: Jaroslav Klat, René Novysedlák; **Democratic Republic of the Congo**: Nicolas Amisi; **Denmark**: Peter Christensen, Alaa El-Hussuna; **Dominican Republic**: Sylvia Batista; **Ecuador**: Eddy Lincango-Naranjo; **Egypt**: Sameh Emile; **El Salvador**: Danilo Alfonso Arévalo Sandoval; **Ethiopia**: Hailu Dhufera, Samuel Hailu, Mengistu G Mengesha; **Finland**: Joonas H Kauppila; **France**: Alexis P Arnaud; **Georgia**: Zaza Demetrashvili; **Germany**: Markus Albertsmeier, Hans Lederhuber, Markus W. Löffler; **Ghana**: Daniel Kwesi Acquah, Bernard Ofori, Stephen Tabiri; **Greece**: Symeon Metallidis, Georgios Tsoulfas; **Guatemala**: Maria-Lorena Aguilera-Arevalo, Gustavo Recinos; **Hungary**: Tamás Mersich, Dániel Wettstein; **India**: Dhruva Ghosh; **Indonesia**: Gabriele Kembuan; **Iran**: Peiman Brouki Milan, Mohammad Hossein Khosravi, Masoud Mozafari; **Iraq**: Ahmed Hilmi; **Ireland**: Helen Mohan; **Israel**: Oded Zmora; **Italy**: Gaetano Gallo, Francesco Pata, Gianluca Pellino; **Japan**: Yuki Fujimoto, Naoto Kuroda, Sohei Satoi; **Jordan**: Mohamad K. Abou Chaar, Faris Ayasra; **Kazakhstan**: Ildar Fakhradiyev; **Kenya**: Intisar Hisham Said Hamdun; **Korea, Republic (South)**: Jang Jin-Young; **Kuwait**: Mohammad Jamal; **Lebanon**: Lina Karout; **Libya**: Muhammed Elhadi; **Lithuania**: Aiste Gulla; **Madagascar**: Fanjandrainy Rasoaherinomenjanahary, Luc Hervé Samison; **Malaysia**: April Camilla Roslani; **Mexico**: Iran Irani Durán Sánchez, Diana Samantha Gonzalez, Laura Martinez, María José Martínez, Alejandra Nayen, Antonio Ramos-De la Medina; **Mongolia**: Jade Nunez; **Morocco**: Oumaima Outani; **Namibia**: Pueya Rashid Nashidengo; **Nepal**: Rakesh Shah, Ashish Lal Shrestha; **Netherlands**: Pascal Jonker, Schelto Kruijff, Milou Noltes, Pieter Steinkamp; **New Zealand**: Chris Varghese, Deborah Wright; **Nigeria**: Lukman Abdur-Rahman, Adesoji Ademuyiwa, Adewale Adisa, Babatunde Osinaike, Justina Seyi-Olajide, Omolara Williams, Emmanuel Williams; **North Macedonia**: Sofija Pejkova; **Oman**: Zainab Al Balushi; **Pakistan**: Ahmad Uzair Qureshi, Raza Sayyed; **Palestine**: Mustafa Abo Mohsen, Sadi A. Abukhalaf; **Panama**: Moises Cukier; **Paraguay**: Hugo Gomez-Fernandez; **Peru**: Sebastian Shu Yip, Ximena Paola Vasquez Ojeda; **Philippines**: Marie Dione Sacdalan; **Poland**: Piotr Major; **Portugal**: José Azevedo, Miguel F. Cunha, Irène Santos; **Qatar**: Ahmad Zarour; **Romania**: Eduard-Alexandru Bonci, Ionut Negoi; **Russia**: Sergey Efetov, Viktor Kochetkov, Andrey Litvin; **Rwanda**: Jc Allen Ingabire, Georges Bucyibaruta, Ntirenganya Faustin, Sosthene Habumuremyi, Alphonsine Imanishimwe, Haragirimana Jean de Dieu, Emmanuel Munyaneza, Isaie Ncogoza; **Saudi Arabia**: Ehab Alameer; **Senegal**: Abdourahmane Ndong; **Serbia**: Dejan Radenkovic; **Singapore**: Min Hoe Chew, Frederick Koh, James Ngu; **Slovakia**: Arpád Panyko; **Slovenia**: Uros Bele, Jurij Aleš Košir; **Somalia**: Hassan Daoud; **Spain**: Ruth Blanco-Colino, Ana Maria Minaya Bravo; **Sri Lanka**: Umesh Jayarajah, Dakshitha Wickramasinghe; **Sudan**: Mohammed Elmujtba Adam Essa Adam; **Sweden**: Martin Rutegård, Malin Sund; **Switzerland**: Michel Adamina, Eleftherios Gialamas, Karoline Horisberger; **Syria**: Muhammad Alshaar; **Taiwan**: Abel Huang; **Thailand**: Varut Lohsiriwat; **Trinidad and Tobago**: Shane Charles; **Tunisia**: Haithem Jlassi; **Turkey**: Arda Isik, Sezai Leventoğlu; **Uganda**: Hervé Monka Lekuya, Hervé Monka Lekuya, Herman Lule; **Ukraine**: Slava Kopetskyi; **United Arab Emirates**: Hayder Alsaadi, Sattar Alshryda; **United States**: Osaid Alser, Brittany Bankhead-Kendall, Kerry Breen, Haytham Kaafarani, Hassan Mashbari; **Uruguay**: Fernando Bonilla Cal; **Yemen**: Hamza Al-Naggar; **Zambia**: Mayaba Maimbo; **Zimbabwe**: Dennis Mazingi. **Non-country specific:** Tom Abbott, Michel Adamina, Melika Akhbari, Ruth Benson, Shivam Bhanderi, Bruce Biccard, Edward Caruana, Sohini Chakrabortee, Reema Chapatwala, Ainhoa Costas-Chavarri, Andreas K Demetriades, Anant Desai, Salomone Di Saverio, Thomas Drake, John Edwards, Jonathan Evans, Marco Fiore, Samuel Ford, Christina Fotopoulou, Alexander Fowler, Kaori Futaba, Ian Ganly, Harelimana Grace James, Ewen Griffiths, Alessandro Gronchi, Peter Hutchinson, Gabriella Yael Hyman, Joseph Incorvia, Ritu Jain, Michael Jenkinson, Tabassum Khan, Stephen Richard Knight, Angelos Kolias, Søren Kudsk-Iversen, Tsun Yu Kwan, Elaine Leung, Julio Mayol, Siobhan McKay, John G. Meara, Emily Mills, Susan Moug, Akshay Patel, Roberto Perinotti, Henry E. Rice, Keith Roberts, Andrew Schache, Richard Shaw, Neil Smart, Matthew Stephens, Grant D. Stewart, Ella Teasdale, Peter Vaughan-Shaw, Raghavan Vidya, Naomi Wright, Funmilola Wuraola, Natalie Zimmelman. **Corporate members:** Association of Surgeons in Training (ASiT), European Society of Coloproctology (ESCP), EuroSurg, G4 Alliance, GlobalPaedSurg, Global Initiative for Children’s Surgery (GICS), ItSURG, Irish Surgical Research Collaborative (ISRC), Italian Society of Colorectal Surgery (SICCR), PTSurg, S-ECCO, South African Society of Anaesthesiologists, SpainSurg. |
| --- |

#### Hospital Leads:

| **Albania**: Ervis Agastra *(Regional Hospital of Durres, Durres)*; Dariel Thereska *(University Hospital Center Nene Tereza, Tirana)*; Irida Dajti *(University hospital Koco Gliozheni, Tirana)*. |
| --- |
| **Argentina**: Sergio Martin Lucchini *(Sanatorio Allende - Sede Cerro, Allende, Cordoba)*; Veronica Laudani *(Hospital General de Niños Pedro de Elizalde, Buenos Aires)*; Luis Boccalatte *(Hospital Italiano de Buenos Aires, Buenos Aires)*; Carina Chwat *(Hospital Universitario Austral, Buenos Aires)*; Ivana Ines Pedraza Salazar *(Instituto Oncológico Alexander Fleming, Ciudad Autónoma Buenos Aires)*; Diana Alejandra Pantoja Pachajoa *(Clinica Universitaria Reina Fabiola, Cordoba)*; Sergio Martin Lucchini *(Sanatorio Allende - Sede Nueva Cordoba, Cordoba)*; Agustin Duro *(Hospital Prof Dr Bernardo A Houssay, Provincia de Buenos Aires)*; José Alfredo Calderón Arancibia *(Hospital Público Materno Infantil de Salta, Salta)*. |
| **Australia**: Daniel Cox, Giuliana D’Aulerio, Nagendra Dudi-Venkata, Natasha Egoroff, Shebani Farik, Natalie Lott, Jana-Lee Moss, Sarah Rennie, Lorwai Tan, Chris Varghese, Uyen Giao Vo, David Watson, David Watters, Deborah Wright *(Oceania Coordinating Committee)*; Tim Bright, Paul Hollington, Xuanyu Zhou *(Flinders Medical Centre, Adelaide)*; Hidde M Kroon *(Royal Adelaide Hospital, Adelaide)*; Anthony Farfus *(The Queen Elizabeth Hospital, Adelaide)*; John Barker *(Armidale Rural Referral Hospital, Armidale)*; Eleanor Watson *(Ballarat Base Hospital, Ballarat)*; Sean Stevens *(Colac Area Health, Colac)*; Haider Latif *(University Hospital Geelong, Geelong)*; Amanda Caroline Dawson *(Gosford Hospital, Gosford)*; Alwin Chuan *(Liverpool Hospital, Liverpool)*; Vijayaragavan Muralidharan *(Austin Hospital, Melbourne)*; Enoch Wong *(Box Hill Hospital, Melbourne)*; Travis Ackermann *(Casey Hospital, Melbourne)*; Maurizio Pacilli *(Monash Childrens Hospital, Melbourne)*; Russell Hodgson *(Northern Hospital, Melbourne)*; Alexander Heriot *(Peter MacCallum Cancer Centre, Melbourne)*; Peter Choong *(St Vincent’s Hospital, Melbourne)*; Wendy Brown *(The Alfred Hospital, Melbourne)*; Surjit Lidder *(The Royal Melbourne Hospital, Melbourne)*; Justin Yeung *(Western Health - Footscray hospital and Sunshine hospital, Melbourne)*; Luke Traeger *(Mount Gambier and Districts Health Service, Mount Gambier)*; Guillermo Regalo *(Belmont District Hospital, Newcastle)*; Ralph Gourlay *(Calvary Mater Newcastle, Newcastle)*; Peter Pockney *(John Hunter Hospital, Newcastle)*; Peter Pockney *(Maitland Hospital, Newcastle)*; Sarit Badiani *(Bankstown Hospital, Sydney)*; Cherry Koh *(Lifehouse, Sydney)*; Soni Putnis *(Wollongong Public Hospital, Wollongong)*; Amanda Caroline Dawson *(Wyong Public Hospital, Wyong)*. |
| **Bahrain**: Fayza Haider *(Salmaniya Medical Complex, Manama)*. |
| **Bangladesh**: Ashrarur Rahman Mitul *(Dhaka Shishu (Children) Hospital, Dhaka)*. |
| **Belgium**: Niels Komen *(University Hospital Antwerp, Antwerp)*; Bert Dhondt *(AZ Rivierenland, Bornem)*; Serge Cappeliez, Manon Pigeolet *(CHU de Charleroi, Charleroi)*; Gabrielle van Ramshorst *(University Hospital of Ghent, Gent)*; Martijn Schoneveld, Jasper Stijns *(UZ Brussel, Jette)*; Wouter Oosterlinck *(UZ Leuven, Leuven)*; Nicolas Flamey *(AZ Delta, Roeselare)*. |
| **Benin**: Cyrille Kpangon *(Centre Hospitalier Universitaire de Zone de Suru-Lére, Cotonou)*; Mouhamed Agbadebo *(Zoumè - Hôpital de Zone de Dassa-Zoumè, Dassa)*; Sèmèvo Romaric Tobome *(Centre Hospitalier Départemental de l’Atacora, Natitingou)*; Ismaïl Lawani *(Centre Hospitalier Universitaire et Departemental Oueme Plateau, Porto Novo)*. |
| **Bosnia and Herzegovina**: Anis Cerovac *(University Clinical Center Tuzla, Tuzla)*. |
| **Brazil**: Aldo Vieira Barros *(Hospital Santa Casa de Misericordia de Maceio, Maceio)*; Samuel Aguiar Júnior, Glauco Baiocchi, Heloisa Galvão do Amaral Campos, Jefferson Gross, Felipe José Fernandez Coimbra, Luiz Paulo Kowalski, Fabiana Makdissi, Suely Nakagawa, Joao Pedreira Duprat Neto, Jose Guilherme Vartanian, Guilherme Yazbek, Stenio C Zequi *(A.C. Camargo Cancer Center, São Paulo)*; Ronald Flumignan *(Hospital São Paulo, São Paulo)*. |
| **Bulgaria**: Mihail Slavchev *(University Hospital Eurohospital, Plovdiv)*. |
| **Canada**: Natalia Jaworska *(Foothills Medical Centre - University of Calgary, Calgary)*; Angela Dell *(University of Alberta Hospital, Edmonton)*; Harsha Shanthanna *(St. Joseph’s Healthcare Hamilton, Hamilton)*; Janet Martin *(London Health Sciences Centre and St Josephs Health Care London, London)*; Abdollah Behzadi *(Trillium Health Partners, Mississauga)*; Carolyn Nessim *(The Ottawa Hospital, Ottawa)*; Michelle Mozel *(Eagle Ridge Hospital, Port Moody)*; Pascal St-germain *(Centre Hospitalier Universitaire de Québec, Québec)*; Crispin Russell *(Saint John Regional Hospital, Saint John)*; Gary Groot *(Saskatoon City Hospital/Royal University Hospital/St. Paul’s Hospital, Saskatoon SK)*; Najib Safieddine *(Michael Garron Hospital, Toronto)*; Duminda Wijeysundera *(St. Michael’s Hospital, Toronto)*; Antoine Eskander *(Sunnybrook Hospital, Toronto)*; Sami Chadi *(Toronto Western Hospital, Toronto)*; Shawn MacKenzie *(Royal Columbian Hospital, Vancouver)*; Alana Flexman *(Vancouver General Hospital, Vancouver)*. |
| **Chile**: Fernando Heredia *(Clínica Universitaria de Concepción, Concepción)*; Maria Marta Modolo *(Hospital Barros Luco Trudeau, Santiago)*; Julio Villanueva *(Hospital Clínico San Borja-Arriarán, Santiago)*; Sofia Waissbluth *(Hospital Clinico Universidad Católica, Santiago)*; Roberto Macchiavello *(Hospital de Urgencia Asistencia Pública Dr Alejandro del Río, Santiago)*; Mario I Escudero *(Hospital San Jose, Santiago)*; Tyare Fuentes *(Hospital Sótero de Río, Santiago)*; Ximena Mimica *(Instituto Oncologico Fundacion Arturo Lopez Perez, Santiago)*; Maricarmen Olivos *(Roberto del Río Children’s Hospital, Santiago)*. |
| **Colombia**: Dinimo Bolivar Saenz *(Clinica Colsubsidio Calle 94, Bogota)*; Lina Caicedo *(Clinica Reina Sofia, Bogota)*; Juan Pablo Alzate, Joaquin Luna *(Clínica Universitaria Colombia, Bogota)*; Nestor Fabian Pedraza Alonso *(Colombiana de Trasplantes, Bogota)*; Camilo Ortiz Silva *(El Tunal, Bogota)*; Carlos J- Perez Rivera *(Fundacion Cardioinfantil-IC, Bogota)*; Juliana Rodriguez *(Fundacion Santa Fe de Bogota, Bogota)*; Liliana Silva-Igua, Martha Luz Torres *(Hospital Universitario Mayor, Bogota)*; Lina María Trujillo *(Instituto Nacional de Cancerologia, Bogota)*; Albaro José Nieto Calvache *(Fundación Valle del Lili, Cali)*; Julián Balanta-Melo *(Hospital Universitario del Valle Evaristo García, Cali)*; Rafael Figueroa - Casanova *(Clinica Avidanti - Ibague, Ibague)*; Oscar-Julián García-Montoya *(Clínica Avidanti - Manizales, Manizales)*; Carlos Andres Marulanda Toro, Marcela Velez Botero *(SES Hospital de Caldas, Manizales)*; Maria Clara Mendoza Arango *(Hospital Universitario San Vicente Fundacion, Medellin)*; Eneida Diaz Martinez *(IMAT Oncomedica, Monteria)*; Valentina Gutiérrez Perdomo *(Hospital Universitario Hernando Moncaleano Perdomo, Neiva)*; Jose Andres Calvache *(Hospital Susana Lopez de Valencia, Popayan)*; Jose Andres Calvache *(Hospital Universitario San José, Popayán)*; Emileth Montenegro *(Clínica Avidanti Santa Marta, Santa Marta)*. |
| **Croatia**: Jakov Mihanovic *(Zadar General Hospital, Zadar)*. |
| **Cuba**: Pablo Mijahil Avilés Jiménez *(Hospital Pediátrico Juan Manuel Márquez, La Habana)*. |
| **Cyprus**: Nikolaos Gouvas *(Nicosia General Hospital, Nicosia)*. |
| **Czech Republic**: René Novysedlák *(Motol University Hospital, Prague)*. |
| **Dominican Republic**: Julia Rodriguez-Abreu *(CEDIMAT - Centro de Diagnóstico, Medicina Avanzada, Laboratorio y Telemedicina, Santo Domingo)*; Dolores Mejía *(Hospital General Plaza de la Salud, Santo Domingo)*. |
| **Ecuador**: Eddy Lincango-Naranjo *(AXXIS Hospital Quito Ecuador, Quito)*. |
| **Egypt**: Galal Abouelnagah, Sameh Shehata *(Alexandria Main University Hospital, Alexandria)*; Ahmed Hossam Eldin Fouad Rida *(El Hadara University Hospital Alexandria University, Alexandria)*; Ramy A. Hassan, Mahmoud M. Saad *(Assiut University Hospital, Assiut)*; Mohamed Reda Loaloa *(Benha University Hospital, Benha)*; Badr Mostafa, Mohamed Qassem *(Ain Shams University Specialized Hospital, Cairo)*; Mohamed Fahmy *(Al Zahraa University Hospital, Cairo)*; Hesham Abozied *(EL-Hussein University Hospital, Al-Azhar University, Faculty Of Medicine, Cairo)*; Ahmed Y Azzam *(Damietta Specialized Hospital, Damietta)*; Sherief Ghozy *(sheikh zayed specialized hospital, giza)*; Asser Sallam *(Suez Canal University Hospital, Ismailia)*; Ahmed Shehta *(Gastrointestinal surgery center, Mansoura)*; Sameh Emile *(Mansoura University Hospital, Mansoura)*; Mohamed Abdelkhalek *(Oncology Center Mansoura University, Mansoura)*; Rehab Samaka *(Menofia University Hospital, Menofia)*; Amr Morsy *(Minya University Hospital, Minya)*; Ahmed Elshawadfy Sherif *(National Liver Institute, Menoufia University, Shibin Elkom)*. |
| **El Salvador**: Danilo Alfonso Arévalo Sandoval *(Clínica de Gineco-Oncología Dr. Danilo Arévalo, San Salvador)*. |
| **Ethiopia**: Abraham Negussie *(ALERT center, Addis Ababa)*; Tigist Fisseha *(Eka kotebe General Hospital, Addis Ababa)*; Kibruyisfaw Shumbash *(Myungsung Christian Medical Centre, Addis Ababa)*; Metasebia Abebe *(Saint Paul Hospital Millennium Medical College, Addis Ababa)*; Samuel Hailu *(Tikur Anbessa Specialized (Black Lion) Hospital, Addis Ababa)*; Seid Mohammed Yasin *(Yekatit 12 hospital medical college, Addis Ababa)*; Yemisirach Bizuneh Akililu *(Zewditu Memorial Hospital, Addis Ababa)*; Abebe Megersa *(Ambo University Referral Hospital, Ambo)*; Teshome Tefera *(Arbaminch General Hospital, Arbaminch)*; Melatework Assefa *(Adisalem Primary Hospital, Bahir Dar)*; Bahru Atnafu *(Bahir Dar University Tibebe Ghion Specialized Hospital, Bahir Dar)*; Bereket Tsegaye *(Debre Berhan Comprehensive Specialized Hospital, Debre Birhan)*; Yoseph Solomon Bezabih *(Debre Markos Comprehensive specialized Hospital, Debre Markos)*; Silamlak Sisay *(Dessie Referral Hospital, Dessie)*; Kebebe Bekele *(Maddawalabu University Goba Referral Hospital, Goba)*; Moa Jira *(Hiwot Fana specialized University Hospital, Harar)*; Mengistu G Mengesha *(Hawassa University Comprehensive Specialized Hospital, Hawassa)*; Habtamu Derilo *(Wachemo University Nigist Elleni Mohammed Memorial Referral Hospital, Hossana)*; Eyueal Degefa *(Jimma University Medical Center, Jimma)*; Anteneh Tadesse *(Mekelle University Ayder Comprehensive Specialised Hospital, Mekelle)*; Melkamu Nidaw *(Pawi General Hospital, Pawi)*. |
| **Finland**: Elise Sarjanoja *(Länsi-Pohja Central Hospital, Kemi)*; Joonas H Kauppila *(Oulu University Hospital, Oulu)*. |
| **France**: Sylvie Testelin *(CHU Amiens, Amiens)*; Sophie Boucher *(CHU Angers, Angers)*; Lionel Jouffret *(Centre Hospitalier Avignon, Avignon)*; Zaher Lakkis *(CHU Besançon, Besancon)*; Alban Zarzavadjian Le Bian *(Avicenne Hospital, Bobigny)*; Luke Harper *(CHU Bordeaux, Bordeaux)*; Marc Danguy des Déserts *(Military Hospital Clermont Tonnerre (Hôpital des Armées), Brest)*; Benoît André *(Centre Hospitalier Intercommunal de Castres-Mazamet, Castres)*; Karem Slim *(Ferrand - CHU Clermont-Ferrand, Clermont)*; Romain Verhaeghe *(Clinique des 2 Caps, Coquelles)*; Andrea Police *(Hôpital Simone Veil, Eaubonne)*; Edouard Girard *(CHU Grenoble-Alpes, Grenoble)*; Alexandre Chebaro *(CHU Lille Hôpital Claude Huriez, Lille)*; Armande Subayi Nkembi *(CHU Lille hôpital Jeanne de Flandres, Lille)*; Laurent Arnalsteen *(Hôpital Privé La Louvière, Lille)*; Quentin Ballouhey *(CHU Limoges, Limoges)*; Diane Mege *(Timone, Marseille)*; Clement Jeandel *(CHU Montpellier (Lapeyronie), Montpellier)*; Emilie Duchalais *(CHU Nantes, Nantes)*; Pierre-Alban Bouche *(Hôpital Cochin - APHP, Paris)*; Gilles Manceau *(Hôpital européen Georges-Pompidou, Paris)*; Célia Crétolle *(Hôpital Necker Enfants Malades - APHP, Paris)*; Erik Hervieux *(Hôpital Trousseau - APHP, Paris)*; Noémie Girard *(Institut Curie, Paris)*; Agathe Seguin-Givelet *(institut mutualiste montsouris, Paris)*; Sebastien Gaujoux *(Pitie Salpetriere, Paris)*; Belinda De Simone *(Centre Hospitalier Intercommunal Poissy Saint Germain en Laye, Poissy)*; Matthieu Boisson *(CHU Poitiers, Poitiers)*; Damien Bergeat *(CHU Rennes - Hopital Pontchaillou, Rennes)*; Alexis P Arnaud *(CHU Rennes - Hopital Sud, Rennes)*; Fabien Fredon *(Junien - Centre Hospitalier Roland Mazoin, Saint)*; Francesco Nappi *(Centre Cardiologique du Nord, Saint Cenis)*; Radwan Kassir *(CHU Reunion, Saint Denis)*; Aurélien Scalabre *(CHU Saint Etienne, Saint Etienne)*; Federico Migliorelli *(Centre Hospitalier Intercommunal des Vallées de l’Ariège, Saint Jean de Verges)*; Romain Verhaeghe *(CMCO Côte d’Opale, Saint Martin Boulogne)*; Anne-Cecile Ezanno *(Hia Begin, St Mande)*; Barbara Seeliger *(HUS, Pole Hépato-Digestif / IHU-Strasbourg, Strasbourg)*; Charlotte Vaysse *(CHU Toulouse, Toulouse)*; Helene Charbonneau, Vincent Misrai *(Clinique Pasteur, Toulouse)*; Olivier Abbo *(Hôpital des Enfants -CHU Toulouse, Toulouse)*; Martina Aida Angeles *(Institut Claudius Regaud - Institut Universitaire du Cancer de Toulouse, Toulouse)*; Laurent Brunaud *(lès-Nancy - CHRU NANCY, Vandoeuvre)*. |
| **Georgia**: Zaza Demetrashvili *(N.Kipshidze Central University Clinic, Tbilisi)*. |
| **Germany**: Ali Modabber *(University Hospital Aachen, Aachen)*; Sebastian Wolf *(University Hospital Augsburg, Augsburg)*; Carsten Kamphues *(Charité University Medicine - Campus Benjamin Franklin, Berlin)*; Philipp Höhn *(St. Josef-Hospital, Bochum)*; Tim R. Glowka *(University Hospital Bonn, Bonn)*; Alexander Christopher Rokohl *(University Hospital of Cologne, Cologne)*; Ulrich Bork *(University Hospital Carl Gustav Carus, Technical University Dresden, Dresden)*; Georg Fluegen *(University Hospital Duesseldorf, Duesseldorf)*; Raymund E. Horch *(Universitätsklinikum Erlangen, Erlangen)*; Andrea Schmedding, Andreas Schnitzbauer *(Frankfurt University Hospital, Goethe University, Frankfurt)*; Helge Eberbach, Daniel Schlager *(University Medical Center Freiburg, Faculty of Medicine, Freiburg)*; Fritz Spelsberg *(Klinikum Fürstenfeldbruck, Fürstenfeldbruck)*; Lena Keppler *(Partenkirchen - Klinikum Garmisch-Partenkirchen, Garmisch)*; Andreas Hecker *(University Hospital Giessen and Marburg, Giessen)*; Susanne Wolfer *(University Hospital Goettingen / Universitätsmedizin Goettingen, Goettingen)*; Ulrich Ronellenfitsch *(University Hospital Halle, Halle)*; Christine Nitschke *(University Medical Center Hamburg-Eppendorf, Hamburg)*; Christian Peiper *(Evangelisches Krankenhaus Hamm, Hamm)*; Ibrahim Hakami *(KRH Nordstadt-Siloah Hospitals, Hannover)*; Stefan Welter *(Lung Clinic Hemer, Hemer)*; Karine Nikolaieva *(Sana Klinikum, Hof)*; Andreas Roth *(University Hospital Leipzig, Leipzig)*; Judith Lindert *(University Hosital Schleswig- Holstein, Lübeck)*; Konstantinos Gousias *(St Marien Hospital Lünen, Lünen)*; Anke Rissmann *(University Hospital Magdeburg, Magdeburg)*; Valerie Catherine Linz *(University Hospital Mainz, Mainz)*; Nuh Rahbari, Marie-Claire Rassweiler-Seyfried *(Mannheim University Medical Center (Universitätsmedizin Mannheim), Mannheim)*; Anna Eleonora Gut *(Isarklinikum, München)*; Jens Gempt, Daniel Reim, Arthur Wagner *(Klinikum Rechts der Isar TUM School of Medicine, Munich)*; Markus Albertsmeier, Alexander M. Keppler *(Ludwig Maximilian University of Munich - Großhadern, Munich)*; Mircea Gabriel Stoleriu *(Asklepios Pulmonary Hospital, Munich Gauting)*; Tim Saier *(Berufsgenossenschaftliche Unfallklinik Murnau, Murnau)*; Josef Stadler *(RoMed Klinik Prien am Chiemsee, Prien am Chiemsee)*; Julia Christina Kaiser *(Klinikum St. Hedwig, Barmherzige Brüder, Regensburg)*; Stefan M. Brunner, Karin Pfister *(University Hospital Regensburg, Regensburg)*; Jonas Herzberg *(Krankenhaus Reinbek St. Adolf-Stift, Reinbek)*; Kai Nowak, Tobias Reinhard *(RoMed Klinikum Rosenheim, Rosenheim)*; Gregor A. Stavrou *(Klinikum Saarbruecken, Saarbruecken)*; Alfred Königsrainer, Christian Konrads, Markus Quante *(University Hospital Tuebingen, Tuebingen)*; Simon Laban *(University Hospital Ulm, Ulm)*; Silke Pusch von *(Kreisklinik Wolfratshausen, Wolfratshausen)*; Markus Hirschburger *(Klinikum Worms, Worms)*; Johannes Doerner *(Helios Universitätsklinikum Wuppertal (Universität Witten/Herdecke), Wuppertal)*; Armin Wiegering *(University Hospital Würzburg, Würzburg)*. |
| **Greece**: Ekaterini Christina Tampaki *(KAT Athens General Hospital, Athens)*. |
| **Guatemala**: Alitza Gutiérrez Ruiz *(Hospital Universitario Esperanza, Guatemala)*; Alejandra Rodas *(Centro Clínico Cabeza y Cuello, Guatemala City)*; Ana Lucía Portilla, Gustavo Recinos *(Hospital General De Enfermedades, Guatemala City)*; Maria-Lorena Aguilera-Arevalo, Jacqueline Carrera *(Hospital General San Juan De Dios, Guatemala City)*; Amalia Barrios Duarte *(Hospital Herrera Llerandi, Guatemala City)*; Megan Lowey *(Sanatorio Las Majadas, Guatemala City)*; Sabrina Barillas *(Symmetria, Guatemala City)*. |
| **India**: Atul Suroy *(India Coordinating Committee)*; Dhaivat Vaishnav *(Zydus Hospital, Ahmedabad Gujarat)*; Raghunandan Gorantlu Chowdappa *(Shri Krishna Hospital and pramukhswami medical college, Anand , Gujarat)*; Irappa Madabhavi *(Kerudi Cancer Hospital, Bagalkot)*; Dhananjaya Bhat *(Aster RV Hospital, Bangalore)*; Sunil Kumar Venkatappa *(Victoria Hospital, Bangalore)*; Sumit Thakar *(Sri Sathya Sai Institute of Higher Medical Sciences, Bengaluru)*; Kavitha Jain *(Sri Shankara Cancer Hospital and Research Centre, Bengaluru)*; Aruna Kumar *(Gandhi Medical College and Sultania Zanana Hospital, Bhopal)*; Manoj Nagar *(ALL INDIA INSTITUTE OF MEDICAL SCIENCE BHOPAL, BHOPAL)*; Tushar Mishra, Arunkumar Sekar *(All India Institute Of Medical Sciences - Bhubaneswar, Bhubaneswar)*; Anand Gupta *(Government medical college hospital, Chandigarh)*; Lileswar Kaman, Madhivanan Karthigeyan, Manjul Tripathi *(Postgraduate Institute of Medical Education & Research, Chandigarh, India, Chandigarh)*; Ashwin Rammohan *(Dr.Rela Institute & Medical Centre, Chennai)*; Sudheer Othiyil vayoth, Anupama Rajanbabu *(Amrita Institute of Medical Sciences Hospital, Cochin)*; Anbukkani Subbian *(Kovai Medical Centre and Hospital, Coimbatore)*; Rahul Gupta *(Synergy Institute of Medical Sciences, Dehradun)*; Monish Raut *(Artemis Health Institute, Gurugram)*; Nissi Evelyn. R *(Government Dental College and Hospital, Hyderabad)*; Lavanya Kannaiyan *(Rainbow children’s hospital, Hyderabad)*; Dr. Anil Matai *(Santokh ba Durlabhji memorial hospital, Jaipur)*; Sanjeev Misra *(All India Institute of Medical Sciences (AIIMS), Jodhpur, Jodhpur)*; Vishal Bhende *(Shree Krishna Hospital, Karamsad)*; Sathish Muthu *(Government Hospital, Velayuthampalayam, Karur)*; Indranil Ghosh *(Institute of Neurosciences, Kolkata, Kolkata)*; Abhishek Sharma *(Tata Medical Center, Kolkata)*; Ankur Bajaj, Shiv Rajan *(King George’s Medical University, Lucknow)*; Gaurav Agarwal *(Sanjay Gandhi Post Graduate Institute Of Medical Sciences, Lucknow)*; Pranay Pawar *(Christian Medical College & Hospital, Ludhiana)*; Philip Alexander *(Lady Willingdon Hospital, Manali)*; M Vijayakumar Vijayakumar *(Yenepoya medical college hospital, Mangalore)*; BM Zeeshan Hameed, Badareesh L *(Kasturba Medical College Hospital, Manipal, Manipal)*; Navneet Kumar Chaudhry *(Maharishi Markandeshwar Institute of Medical Sciences & Research, Mullana, Ambala, Haryana)*; Lipika Baliarsing, Satish Dharap *(BYL Nair Hospital, mumbai)*; Amruta Kulkarni *(Fortis Hospital Mulund, Mumbai)*; Yuvaraja Thyavihally *(Kokilaben Dhirubhai Ambani Hospital, Mumbai)*; Rahul Deo Sharma *(Lilavati Hospital & Research Centre, Mumbai)*; C S Pramesh *(Tata Memorial Hospital, Mumbai)*; Rajesh Soni *(Soni Hospital, Nagpur)*; Surya Kumar Dube, Shilpa Sharma *(All India Institute of Medical Sciences, New Delhi)*; Harvinder Singh *(Indian Spinal Injuries Center, New Delhi)*; Lovenish Bains *(Maulana Azad Medical College, New Delhi)*; Rahul Ghodke *(Sanjay Clinic & Apollo Hospital, New Mumbai)*; Ashwani Kumar *(Government Medical College, Patiala)*; Vivek Sodhai *(Lokmanya Hospital for Special Surgery, Pune)*; Suvendu Maji *(Bengal cancer foundation(BIMS Hospital), Purba Bardhaman)*; Somprakas Basu *(All India Institute Of Medical Sciences, Rishikesh)*; Chandrashekhar Mahakalkar *(Acharya Vinoba Bhave Rural Hospital, Sawangi (Meghe), Wardha)*; Ravi Kannan *(Cachar Cancer Hospital and Research Centre, Silchar)*; Asif Mehraj *(Sher-i-Kashmir Institute of Medical Sciences, Srinagar)*; Ranganath N *(KVG Medical College & Hospital, Sullia)*; Ashish Phadnis *(jupiter hospital, thane)*; I Yadev *(Government Medical College Thiruvananthapuram, Thiruvananthapuram)*; Alfie Kavalakat *(Jubilee Mission Medical College & Research Institute, Thrissur)*; Rohin Mittal *(Christian Medical College & Hospital, Vellore)*; Karthik Chandra Vallam *(Mahatma Gandhi cancer hospital and research institute, Visakhapatnam)*. |
| **Iran, Islamic Rep.**: Hamed Akhavizadegan, Esmaeil Rezghi Maleki, Naser Yousefzadeh Kandevani *(Baharloo hospital, Tehran)*. |
| **Ireland**: Hilary Ikele, Catherine McNestry *(Mayo University Hospital, Castlebar)*; Christina Fleming, Stephen O’Brien *(Cork University Hospital, Cork)*; Sami Abd Elwahab, Niall Davis, Mohsen Javadpour *(Beaumont Hospital, Dublin)*; Brendan McDonnell *(Coombe Women and Infants University Hospital, Dublin)*; Clare O Connor *(Rotunda Hospital, Dublin)*; Jarlath Bolger, Cillian Clancy, Stefanie M Croghan, Noel Donlon *(St James’s Hospital, Dublin)*; Carolyn Cullinane *(St Vincent’s University Hospital, Dublin)*; Ben Creavin, Muheilan Muheilan *(Tallaght Hospital, Dublin)*; Helen Earley *(University Hospital Galway, Galway)*; Syed Mohammad Umar Kabir *(LETTERKENNY UNIVERSITY HOSPITAL, Letterkenny)*; Muhammad Fahadullah *(University Hospital Limerick, Limerick)*; Éanna Ryan *(Tullamore University Hospital, Tullamore)*; Tara Connelly *(University Hospital Waterford/University College Cork, Waterford)*. |
| **Israel**: Oded Zmora *(SHAMIR MEDICAL CENTER, BE’ER YA’AKOV)*. |
| **Japan**: Daisuke Hashimoto *(Kansai Medical University, Osaka)*. |
| **Jordan**: Majdi Ali Alqudah *(Al Iman Hospital, Ajloun)*; Amer Alajalen *(Al Karak Hospital, Alkarak)*; Rand Y. Omari *(Abdali Hospital, Amman)*; Faris Ayasra, Abdulrahman Qasem *(Al-Basheer Hospital, Amman)*; Yazan Alawneh *(Ibn Al Haitham Hospital, Amman)*; Amer Ahmad, Omar Aladawi, Bourhan Alrayes, Hanan Haidar, Shatha Husain, Faisal Qassem *(Islamic Hospital, Amman)*; Adnan Sumadi *(Istiklal Hospital, Amman)*; Ala’a Abu Salhiyeh, Balqees Mahmoud Al-Manaseer, Zaid Alsunna, Hazim Ra’ed, Faten Reyad Bani Hamad *(Jordan University Hospital, Amman)*; Amro Abuleil *(Jordanian royal medical services, Amman)*; Mohamad K. Abou Chaar *(King Hussein Cancer Center, Amman)*; Elmi Ahmed Mohamed Jimaale *(The Speciality Hospital, Amman)*; Marah Abu-Mehsen, Noor Olaywah, Omar Wafi *(King Abdullah University Hospital/ Jordan University of Science and Technology, Ar Ramtha)*; Hazim Ababneh *(Ar Ramtha Govermental Hospital, Irbid)*; Luai Abu-Ismail, Almu’atasim Khamees *(Irbid Specialty Hospital, Irbid)*; Ahmad Alkhatib *(Princess Basma Hospital, Irbid)*. |
| **Kazakhstan**: Raikhan Bolatbekova *(Almaty Oncology Centre, Almaty)*; Mukhtar Kulimbet *(City Cardiology Center, Almaty)*; Talgat Nurgozhin, Timur Saliev, Baurzhan Zhussupov *(City Clinical Hospital No. 4, Almaty)*; Ydyrys Almabayev, Ildar Fakhradiyev, Timur Saliev *(Karasay Central District Hospital, Almaty)*; Dilyara Kaidarova *(Kazakh Institute of Oncology and Radiology, Almaty)*. |
| **Libya**: Khalil Tamoos *(Alabyar General Hospital, Alabyar)*; Ahmed Aqeelah *(Althawra Hospital Albyda, Albayda)*; Alsnosy abdullah Khalefa mohammed *(Almarj Teaching Hospital, Almarj)*; Faraj Al maadany, Ghadah Alkadeeki *(Al-jalaa Teaching/Trauma Hospital, Benghazi)*; Milad Gahwagi *(Benghazi Medical Center, Benghazi)*; Wafa Aldressi *(N. B. Hospital, Benghazi)*; Mohamed Amnaina *(Al-Wahda Hospital, Darna)*; Arowa hassan abdulrahman Alansari *(Ghadames general hospital, Ghadames)*; Akram Alkaseek *(Gharyan Central Hospital, Gharyan)*; Ghozlan Yagoub *(Alhikma hospital, Misurata)*; Anass Ben Amer, Marwa Salem *(Misurata Central Hospital, Misurata)*; Ayman Almugaddami *(Nalut Central Hospital, Nalut)*; Dania Burgan *(National Cancer Institute, Sabratha - Libya, Sabratha)*; Mohammed Abdelkabir *(Sabha Medical Center, Sebha)*; Khayriyah Alshareef *(Tripoli - Yashfeen Clinic, Tajora)*; Rayet al islam Ben jouira *(National Heart Centre, Tajoura Heart Center Hospital, Tajoura)*; Ayman Meelad *(Tarhuna General Hospital, Tarhuna)*; Ahmad Bouhuwaish *(Tobruk Medical Center, Tobruk)*; Sumayya Essayah Dwaga, Houda Khalifa *(Aljala Maternity Hospital, Tripoli)*; Bushray Almiqlash *(Alkhadra Hospital, Tripoli)*; Taha Suliaman *(Crown of Health Clinic, Tripoli)*; Mohammed Alawami *(Medical Care Clinic, Tripoli)*; Fras Elhajdawe *(Metiga Hospital, Tripoli)*; Hajir Aboazamazem *(Tripoli Central Hospital, Tripoli)*; Ibrahim Ellojli, Ahmed Msherghi *(Tripoli Medical Center/ Tripoli University Hospital, Tripoli)*; Ismail Ali Saleh *(Zintan Teaching Hospital, Zintan)*; Mohammed Alayan *(Zliten Teaching Hospital, Zliten)*. |
| **Malaysia**: April Camilla Roslani *(University Malaya Medical Centre, Kuala Lumpur)*. |
| **Namibia**: Marcel Didier Ndayishyigikiye *(Ongwediva Medipark Teaching Hospital, Ongwediva)*; Akutu Munyika *(Onandjokwe Intermediate Hospital, Oniipa)*; Philipp Plarre *(Mediclinic Cottage Hospital, Swakopmund)*; David W Borowski *(Welwitschia Hospital, Walvis Bay)*; Pueya Rashid Nashidengo *(Windhoek Central Academic Hospital, Windhoek)*. |
| **Netherlands**: Milou Noltes, Pieter Steinkamp *(University Medical Center Groningen, Groningen)*. |
| **New Zealand**: Cameron Wells *(Middlemore Hospital, Auckland)*; Rebecca Teague *(North Shore Hospital, Auckland)*; Brodie Elliott *(Christchurch Hospital, Christchurch)*; David Kieser *(Southern Cross Hospital, Christchurch)*; Omar Mohyieldin *(Dunedin Public Hospital, Dunedin)*; Chris Varghese *(Waikato Hospital, Hamilton)*; Nick McIntosh *(Hawke’s Bay Hospital, Hastings)*; Cheyaanthan Haran, Sarah Rennie *(Wairarapa Hospital, Masterton)*; Jasmin King *(Taranaki Base Hospital, New Plymouth)*; Jeong Ha *(Wellington Regional Hospital, Wellington)*; Matthew James McGuinness *(Whangarei Hospital, Whangarei)*. |
| **Nigeria**: Opeoluwa Adesanya *(Federal Medical Centre, Abeokuta)*; Julius Olaogun *(Ekiti - Ekiti State University Teaching Hospital, Ado)*; Akinola Akinmade *(Afe Babalola University Multi-System Hospital, Ado Ekiti)*; Kefas Bwala *(Abubakar Tafawa Balewa University Teaching Hospital Bauchi, Bauchi)*; Peter Agbonrofo *(University of Benin Teaching Hospital, Benin City)*; Akinwale Afolabi *(Kebbi - Federal Medical Centre, Birnin)*; Usang Usang *(University of Calabar Teaching Hospital, Calabar)*; Sebastian Ekenze *(University of Nigeria Teaching Hospital, Enugu)*; Samson Olori *(University of Abuja Teaching Hospital, Gwagwalada)*; Taiwo Akeem Lawal *(University College Hospital, Ibadan)*; Justina Seyi-Olajide *(Lagos University Teaching Hospital, Idi Araba)*; Abiodun Okunlola *(Federal Teaching Hospital, Ido Ekiti, Ido Ekiti)*; Omolara Williams *(Lagos State University Teaching Hospital, Ikeja)*; Adewale Adisa *(Ife - Obafemi Awolowo University Teaching Hospitals Complex, Ile)*; Lukman Abdur-Rahman *(University of Ilorin Teaching Hospital, Ilorin)*; Stephen Kache *(Barau Dikko Teaching Hospital, Kaduna)*; Danjuma Sale *(Epsilon Specialist Hospital Barnawa, Kaduna)*; Lofty-John Anyanwu *(Aminu Kano Teaching Hospital, Kano)*; Chukwuma Okereke *(Federal Medical Centre, Owo, Ondo State)*; Musliu Adetola Tolani *(Ahmadu Bello University Teaching Hospital, Zaria)*. |
| **North Macedonia**: Venko Filipce *(University clinic for neurosurgery, Faculty of medicine, University St.Cyril and Methodius, Skopje)*; Lazar Todorovic *(University Clinic for Pediatric Surgery, Skopje)*; Sofija Pejkova *(University Clinic for Plastic and Reconstructive surgery, Faculty of Medicine, University St. Cyril and Mthodius, Skopje)*; Sotir Stavridis *(University Clinic of Urology, Skopje)*. |
| **Oman**: John George Massoud *(Khoula Hospital, Muscat)*; Sareyah Alsibai *(Sultan Qaboos University Hospital, Muscat)*. |
| **Pakistan**: Rizwan Sultan *(Islamabad medical complex, Islamabad)*; Humera Naz Altaf, Abu Bakar Hafeez Bhatti *(Shifa international hospital, Islamabad)*; Shahzad Hussain Waqar *(The Pakistan Institute of Medical Sciences, Islamabad)*; Aliya Aziz *(Aga Khan University, Karachi)*; Asad Ali Kerawala *(Cancer Foundation Hospital, Karachi)*; Lajpat Rai *(Dr Ruth K.M. Pfau Civil Hospital, Karachi)*; Mariyah Anwer *(Jinnah Post Graduate Medical Center, Karachi)*; Aiman Tariq *(National Institute of Cardiovascular Diseases, Karachi)*; Bushra Ayub *(Patel Hospital, Karachi)*; Sami ullah Niazi *(South City Hospital, Karachi)*; Muhammad Yasir Naseem *(Government Mian Meer Hospital Lahore, Lahore)*; Muhammad Zeeshan Sarwar *(King Edward Medical University - Mayo Hospital, Lahore)*; Muhammad Imran Khokhar *(Lahore General Hospital, PGMI, AMC, Lahore)*; Imdad Ahmad Zahid *(Services Hospital Lahore, Lahore)*; Haroon Javaid Majid *(Shaikh Zayed Hospital, Lahore)*; Nabila Talat *(The Children’s Hospital & The Institute of Child Health Lahore, Lahore)*; Muhammad Asif *(District Headquarter Hospital - Mandi Bahauddin, Mandi Bahauddin)*; Muhammad Hamid Chaudhary *(Chaudhary pervaiz elahi institute of Cardiology, Multan)*; Umer Farooq *(Tehsil Headquarters Hospital Pasrur, Pasrur district Sialkot)*; Siddique Ahmad *(Hayatabad Medical Complex, Peshawar)*; Waleed Mabood *(Khyber teaching hospital, Peshawar)*; Syed Imran Bukhari *(Lady Reading Hospital, Peshawar)*; Muhammad Tariq *(North West General Hospital and Research Centre, Peshawar)*; Eesha Yaqoob *(Benazir Bhutto Hospital, Rawalpindi)*; Saad Javed *(District Headquarter Hospital - Rawalpindi, Rawalpindi)*; Saad Javed *(Holy Family Hospital, Rawalpindi)*; Muhammad Usman Malik *(District Headquarter & Teaching Hospital - Sargodha, Sargodha)*; Hassan Nawaz Yaqoob *(Social Security Hospital, Sheikhupura, Sheikhupura)*. |
| **Panama**: Moises Cukier *(Pacifica Salud Hospital, Panama)*. |
| **Peru**: Glenda Marina Falcon Pacheco *(Instituto Regional de Enfermedades Neoplásicas del Sur, Arequipa)*; Robinson Mas Melendez, Arazzelly Del Pilar Paucar Urbina *(Ayacucho Regional Hospital, Ayacucho)*; Jose Rios Chiuyari *(Hospital regional de lambayeque, Lambayeque)*; Carlos Eduardo Otiniano Alvarado *(Arzopispo Loayza National Hospital, Lima)*; Lorena Fuentes Rivera Lau *(British American Hospital, Lima)*; Giuliano Borda-Luque, Milagros Niquen-Jimenez *(Cayetano Heredia National Hospital, Lima)*; Claudia Arias *(Edgardo Rebagliati Martins National Hospital, Lima)*; Sergio Zegarra *(Guillermo Almenara National Hospital, Lima)*; Jenner Betalleluz Pallardel *(Hospital de Emergencias Jose Casimiro Ulloa, Lima)*; Regina Amparo Ugarte Oscco *(Hospital Emergencia Ate Vitarte, Lima)*; Gian Mendiola *(Hospital Santa Rosa de Lima, Lima)*; Yahaira Tatiana Carpio Colmenares *(SANNA - Clínica El Golf, Lima)*; Carlos Shiraishi Zapata *(Hospital Il Talara, Talara)*; Maria Rosa Ortiz *(Hospital Belén de Trujillo, Trujillo, La Libertad)*. |
| **Philippines**: Marie Dione Sacdalan *(Philippine General Hospital, University Of The Philippines Manila, Manila)*. |
| **Poland**: Piotr Major *(Jagiellonian University Medical College, Krakow)*. |
| **Portugal**: Filipe Castro Borges *(Hospital Garcia de Orta, Almada)*; Octavio Viveiros *(Clínica de Santo António - Grupo Lusíadas, Amadora)*; Pedro Serralheiro *(Hospital Dr. Antonio José de Almeida, Cascais)*; Paulo Santos-Costa *(IPO Coimbra, Coimbra)*; Filipa Mendes *(Centro Hospitalar Cova da Beira, Covilha)*; Miguel Rocha Melo *(Hospital do Espirito Santo, Evora)*; Paulo Cardoso, Ana Soares *(Hospital Faro, Centro Hospitalar Universitario do Algarve, Faro)*; José Azevedo *(Hospital da Horta, E.P.E., Horta)*; Rita Gonçalves Pereira *(Centro Hospitalar de Leiria, E.P.E., Leiria)*; Nelson Silva *(Hospital CUF Infante Santo, Lisboa)*; André Caiado *(Instituto Português de Oncologia de Lisboa Francisco Gentil, Lisboa)*; Maria Luís Sacras *(Centro Hospitalar Universitário Lisboa Central, Lisbon)*; Pedro Azevedo *(Hospital Beatriz Angelo, Loures)*; Rui Almeida-Reis *(Centro Hospitalar do Tamega e Sousa, Penafiel)*; Miguel F. Cunha *(Centro Hospitalar Universitario do Algarve - Unidade de Portimão, Portimao)*; João Oliveira *(Centro Hospitalar do Porto, Porto)*; Jorge Nogueiro, Mafalda Sampaio-Alves *(Centro Hospitalar e Universitário de São João, Porto)*; Luciana Cidade Costa *(Hospital da Prelada, Porto)*; Catarina Baía *(IPO Porto, Porto)*; Ana Cláudia Deus *(Hospital do Litoral Alentejano, Santiago do Cacém)*; Rita Branquinho *(Centro Hospitalar Médio Tejo, Tomar)*; André Marçal *(Centro Hospitalar de Trás-os-Montes e Alto Douro, E.P.E., Vila Real)*; André Tojal *(Centro Hospitalar Tondela-Viseu, Viseu)*. |
| **Qatar**: Ahmad Zarour *(Hamad General Hospital, Doha)*; Ahmad Zarour *(Hazm Mebaireek General Hospital, Doha)*. |
| **Romania**: Silviu Tiberiu Makkai-Popa *(Sf. Constantin Hospital, Brasov)*; Aurel Mironescu *(Spitalul Clinic De Copii Brasov, Brasov)*; Florin Grama *(Coltea Clinical Hospital, Bucharest)*; Elena Adelina Toma *(Elias Emergency Hospital, Bucharest)*; Ionut Negoi *(Emergency Clinical Hospital Bucharest, Bucharest)*; Daniela Filipescu *(Emergency Institute for Cardiovascular Diseases ‘Prof. Dr. C.C. Iliescu’, Bucharest)*; Nicolae Bacalbasa *(Fundeni Clinical Institute, Bucharest)*; Natalia Motas *(Institute of Oncology Prof Dr Al Trestioreanu, Bucharest)*; Sebastian Ionescu *(Maria Sklodowska Curie Emergency Hospital, Bucharest)*; Octav Ginghina *(Saint John Emergency Hospital, Bucharest)*; Radu Costea, Narcis Octavian Zarnescu *(University Emergency Hospital Bucharest, Bucharest)*; Radu Drasovean *(Cluj-Napoca Emergency County Hospital, Cluj)*; Eduard-Alexandru Bonci *(Prof Dr Ion Chiricuta Institute of Oncology, Cluj)*; Mihail-Gabriel Dimofte *(Regional institute of Oncology Iasi, Iasi)*; Vlad Porumb *(Spitalul Clinic de Urgență Militar Dr Iacob Czihac, Iasi)*. |
| **Russian Federation**: Mikhail Kirov *(City Hospital #1, Arkhangelsk)*; Yegor Molitvin *(Engels City Clinical Hospital Number 1, Engels)*; Andrey Litvin *(Immanuel Kant Baltic Federal University, Regional Clinical Hospital, Kaliningrad)*; Vadim Pykhteev *(Territorial hospital #2, Krasnodar)*; Marianna Raevskaya *(AV Vishnevsky Center of Surgery, Moscow)*; Sergey Efetov *(IM Sechenov First Moscow State Medical University, Moscow)*; Aleksandr Butyrskii *(Municipal Emegency Hospital No.6, Simferopol)*. |
| **Saudi Arabia**: Mushabab Alshahrani *(aseer central hospital, abha)*; Azah Althumairi *(Ahsa - King Abdulaziz National Guard Hospital, Al)*; Nasser Alzerwi *(Majmaah - King Khalid General Hospital, Al)*; Ahmed Al Ameer *(King Abdullah Hospital, Bisha)*; Ahmed Al Ameer *(Maternity and Children’s Hospital, Bisha)*; Tariq Madkhali *(King Fahad Specialist Hospital, Dammam)*; Abddulrahman Saleh Almulhim *(King Fahad Hospital Hofuf, Hofuf)*; Salman Ghazwani *(King Fahd Central Hospital, Jazan University, jazan)*; Abdu Ayoub *(Prince Mohd Bin Nassir Hospital, Jazan)*; Othman Iskander *(Sabia General Hospital, Jazan)*; Mohammed Ghunaim *(International Medical Center, Jeddah)*; Mohammed Alharthi *(King Abdulaziz University Hospital, Jeddah)*; Turki M Alzaidi *(King Fahad Armed Forces Hospital, Jeddah)*; Azah Althumairi *(King Khalid National Guard Hospital, Jeddah)*; Mohammad Alyami *(King Khalid Hospital, Najran)*; Abdulrahman Al Amri *(Najran university hospital, Najran)*; Azah Althumairi *(King Abdulaziz Medical City, Riyadh)*; Abdullah AlFakhri *(King Fahad Medical City, Riyadh)*; Amal Alhefdhi *(King Faisal Specialist Hospital, Riyadh)*; Sharfuddin Chowdhury *(King Saud Medical City, Riyadh)*; Thamer Nouh *(King Saud University, Riyadh)*; Ameen Alshehri *(Prince Mohammed bin Abdulaziz Hospital, Riyadh)*; Abdulrahman Alzahrani *(Security Forces Hospital, Riyadh)*; Dr Yousef Alalawi *(King Salman Armed Forces Hospital, Tabuk)*; Selmy Awad *(King Faisal Medical Complex, Taif City)*. |
| **Senegal**: Ibrahima Konate, Abdourahmane Ndong, Jacques Tendeng *(Louis - Saint-Louis Regional Hospital, Saint)*. |
| **Singapore**: Nan Zun Teo *(Changi General Hospital, Singapore)*; Frederick Koh *(Sengkang General Hospital, Singapore)*. |
| **Slovenia**: Jurij Aleš Košir *(University Medical Centre, Ljubljana)*; Uros Bele *(University Medical Centre, Maribor)*. |
| **Somalia**: Sabra Aqil *(Hargeisa Group Hospital, Hargeisa)*. |
| **Spain**: Cristina Barrena López, Ana Sánchez Mozo *(Complejo hospitalario universitario de Albacete, Albacete)*; Antonio Rodriguez Infante *(San Agustín University Hospital, Avilés)*; Patricia Caja Vivancos, Mikel Prieto *(Hospital Universitario Cruces, Barakaldo)*; Igor Alberdi San Roman *(San Eloy Hospital, Barakaldo)*; Laura Gomez Fernandez *(Consorci Sanitari de Terrassa, Barcelona)*; Josep Maria Muñoz Vives *(Fundació Althaia - Xarxa Assistencial Universitària de Manresa, Barcelona)*; Anna Carreras-Castañer, Berta Díaz-Feijoo, Ramon Sieira-Gil, Victor Turrado-Rodriguez *(Hospital Clinic Barcelona, Barcelona)*; Anna Sánchez López, Santiago Sánchez-Cabús *(Hospital de la Santa Creu i Sant Pau, Barcelona)*; Marta Jimenez Toscano *(Hospital del Mar, Barcelona)*; MªPilar Canals Sin, Saura García Laura, Oriol Martin Sole, Pedro Palazon Bellver, Sonia Pérez-Bertólez, Jordi Prat-Ortells, Mireia Riba Martínez, Josep Rubio-Palau, Xavier Tarrado *(Hospital Sant Joan de Deu, Barcelona)*; Jorge Nuñez *(Hospital Universitario Mutua de Terrassa, Barcelona)*; Veronica Alonso Mendoza, Coro Bescós, Eloy Espin-Basany, Martin Espinosa-Bravo, Daniel Gil-Sala, Susana González-Suárez, Nuria Montferrer Estruch, Jorge Nuñez, Lucia Porteiro Mariño, Ana Rodríguez-Tesouro, Fabian Rojas Portilla, M Pilar Tormos Pérez, Inmaculada Vives *(Vall d’Hebron University Hospital, Barcelona)*; Unai Garcia De Cortazar, Kiara Tudela *(Hospital Universitario de Basurto, Bilbao)*; Aitor Landaluce-Olavarria *(Hospital Urduliz, Bizkaia)*; Mercedes Estaire Gómez *(Hospital General Universitario de Ciudad Real, Ciudad Real)*; Jorge Almoguera *(Hospital Universitario de Fuenlabrada, Fuenlabrada)*; Bakarne Ugarte-Sierra *(Usansolo - Hospital Universitario de Galdakao, Galdakao)*; Virginia Jimenez *(Getafe University Hospital, Getafe)*; Marta Bertrand, Laura Cárdenas Puiggrós, Olga Delisau-Puig, Jorge Garcia-Adamez, David Julià Bergkvist, Eloy Maldonado-Marcos *(Hospital Universitari de Girona Dr. Josep Trueta, Girona)*; Lucia Diego García *(Hospital Universitario de Guadalajara, Guadalajara)*; Marta Roldón Golet *(Hospital General San Jorge, Huesca)*; Iván Soto-Darias *(Complejo Hospitalario Universitario Insular-Materno Infantil, Las Palmas de Gran Canaria)*; Aida Cristina Rahy-Martín *(Hospital Universitario de Gran Canaria Doctor Negrín, Las Palmas de Gran Canaria)*; Diego Enjuto, Adolfo Ramos-Luengo *(Severo Ochoa University Hospital, Leganés)*; Juan Delgado Fernandez, Carolina Lugo Duarte, Cristina Ojeda Thies *(12 de Octubre University Hospital, Madrid)*; Lucila Marquez *(Hospital Central de la Cruz Roja San Jose y Santa Adela, Madrid)*; Diana Crego Vita *(Hospital Central De La Defensa Gomez Ulla, Madrid)*; Jana Dziakova *(Hospital Clinico San Carlos, Madrid)*; Ana Maria Minaya Bravo *(Hospital del Henares, Madrid)*; Jorge Caño Velasco, Olga Mateo-Sierra, Begoña Quintana-Villamandos, Cristina Rey Valcarcel, Javier Rio *(Hospital General Universitario Gregorio Marañón, Madrid)*; Laura Román García de León *(HOSPITAL PUERTA DE HIERRO MAJADAHONDA, Madrid)*; Marcello Di Martino, Jorge Prada *(Hospital Universitario de la Princesa, Madrid)*; Javier Serrano González *(Hospital Universitario de Torrejón de Ardoz, Madrid)*; Manuel Losada *(Hospital Universitario del Sureste, Madrid)*; Jose Tomas Castell Gomez, Ramon Corripio-Sanchez, Alexander Forero-Torres, José Manuel Morales-Puebla, Hanna Perez-Chrzanowska, Santiago Valderrabano Gonzalez, Alvaro Yebes, Ignacio Zapardiel *(Hospital Universitario la Paz, Madrid)*; Manuel Diez Alonso, Nelson Morales Palacios *(Hospital Universitario Principe de Asturias, Madrid)*; Alberto Cabañero Sánchez, Fátima Sánchez Fernández *(HOSPITAL UNIVERSITARIO RAMON Y CAJAL, Madrid)*; Alfredo Abad Gurumeta, Ane Abad-Motos, Fernando Corella, Javier Ripollés-Melchor, Rosa Sanz-Gonzalez *(Infanta Leonor University Hospital, Madrid)*; Marta Alcaraz Fuentes *(HOSPITAL SANITAS LA ZARZUELA, MADRID)*; Maria Teresa Fernández Martín *(Hospital Medina del Campo, Medina del Campo (Valladolid))*; Pablo Calvo Espino *(Hospital Universitario de Móstoles, Móstoles)*; Milagros Carrasco Prats, Antonio-José Fernández-López, Damián García Escudero, Vanesa Garcia Soria, Jesús Aarón Martínez Alonso, Miguel Ruiz-Marín *(Hospital General Reina Sofía, Murcia)*; Beatriz Gómez Pérez, Joaquin Moya-Angeler *(Hospital Universitario Virgen de la Arrixaca, Murcia)*; Daniel Fernández Martínez *(Hospital Universitario Central de Asturias (HUCA), Oviedo)*; Heura Llaquet Bayo *(Hospital de Palamós, Palamós)*; Enrique Colás-Ruiz *(Hospital Universitario Son Llàtzer, Palma de Mallorca)*; Susana Bella Romera, M.teresa Gavaldà Pellicé, Misericòrdia Jordà Solé, Enrique Jose Ruiz Velasquez *(Hospital Universitari Sant Joan, Reus)*; Bernardo Núñez *(Corporación Sanitaria Parc Taulí, Sabadell (Barcelona))*; Raul Jimenez, Jon Zabaleta *(Hospital Universitario Donostia, San Sebastian)*; Maria Jose González-Gimeno, Irene Ortega Vázquez, Antonio Perez Ferrer *(Infanta Sofía University Hospital, San Sebastian de Los Reyes)*; Rubén Martín-Láez, Marcelo Moreno Suarez *(Marqués de Valdecilla University Hospital, Santander)*; Miguel Angel Freiria Eiras *(Hospital HM Rosaleda, Santiago de Compostela)*; Irene Ramallo-Solís *(Hospital Universitario Virgen del Rocio, Sevilla)*; Juan-Carlos Gomez-Rosado *(Hospital Universitario Virgen Macarena, Seville)*; Jose Ramon Oliver Guillen *(Hospital Santa Bárbara, Soria)*; Mar Achalandabaso Boira *(Hospital Universitari De Tarragona Joan XXIII, Tarragona)*; Juan Carlos Catalá Bauset *(Consorcio Hospital General Universitario, Valencia)*; Julio Domenech *(Hospital Arnau de Vilanova, Valencia)*; Rafael Badenes *(Hospital Clínico Universitario de Valencia, Valencia)*; Juan Carlos Bernal-Sprekelsen *(Hospital Universitario Doctor Peset, Valencia)*; Jorge Sancho-Muriel *(Hospital Universitario y Politécnico La Fe, Valencia)*; Beatriz De Andrés-Asenjo *(Hospital Clínico Universitario de Valladolid, Valladolid)*; Francisco J Tejero-Pintor *(Hospital Universitario Río Hortega, Valladolid)*; Marc Vallve-Bernal *(Hospital Insular Nuestra Señora de Los Reyes, Valverde)*; Alba Vazquez Melero *(Gasteiz - Hospital Universitario Araba, Vitoria)*; Laura Sánchez Blasco *(Hospital General de la Defensa, Zaragoza)*; Jorge Escartin *(Hospital Royo Villanova, Zaragoza)*; Victoria Duque Mallén *(Hospital Universitario Miguel Servet, Zaragoza)*. |
| **Sri Lanka**: Selvaratnam Srishankar *(Teaching Hospital Anuradhapura, Anuradhapura)*; Umesh Jayarajah, Charitha Sooriyabandara *(District General Hospital Chilaw, Chilaw)*; Oshan Basnayake *(Cancer Institute Maharagama, Colombo)*; Nalaka Gunawansa, Dakshitha Wickramasinghe *(National Hospital of Sri Lanka, Colombo)*; Prabuth dulanjan Weeraddana *(Base Hospital Horana, Sri Lanka, Horana)*; Thanusan Vimalakanthan *(Teaching Hospital,Jaffna, Jaffna)*; Shanthamoorthy Gishanthan *(District General Hospital Polonnaruwa, Polonnaruwa)*; Pramodh Chandrasinghe *(North Colombo Teaching Hospital, Ragama)*. |
| **Switzerland**: Eleftherios Gialamas, Marc-Olivier Sauvain *(Hopital de Pourtales, Neuchatel)*. |
| **Syrian Arab Republic**: Ahmad Ghazal *(Aleppo University Hospital, Aleppo)*; Yusra Al-Sabbagh *(Al-Assad University Hospital, Damascus)*; Turki Alhassoun *(Al-Bairouni University Hospital, Damascus)*; Sara Maa Albared *(Al-Hilal Hospital, Damascus)*; Antoine Naem *(Al-Mouwasat University Hospital, Damascus)*; Hareth Alnahr *(Damascus Hospital, Damascus)*; Ghassan Jisry *(Obstetrics and gynaecology University hospital, Damascus)*; Ali Hammed *(Tishreen University Hospital, Latakia)*. |
| **Turkey**: Arda Isik *(Erzincan University Hospital, Erzincan)*. |
| **Uganda**: Okedi Francis Xaviour *(Adjumani General Hospital, Adjumani)*; Gaston Turinawe *(Hoima regional referral hospital, Hoima)*; Isaac Mubezi *(Iganga district hospital, Iganga)*; Franck K. Sikakulya *(Kampala International University Teaching Hospital, Ishaka)*; Andrew Kakeeto *(Jinja regional referral hospital, Jinja)*; Dr. Wilberforce M. Kabweru, Hervé Monka Lekuya *(Mulago Referral hospital, Kampala)*; Ronald Kiweewa *(St Francis Hospital Nsambya, Kampala)*; Herman Lule *(Kiryandongo Hospital, Kigumba)*; Paul Matovu *(Kisiizi Hospital, Rukungiri)*; Otolia Isaac *(Soroti Regional Referral Hospital, Soroti)*. |
| **United Arab Emirates**: Mohamed Mashhour *(Mediclinic Alnour Hospital, Abu Dhabi)*; Amin El Helw *(Sheikh Shakhbout Medical City, Abu Dhabi)*; Sattar Alshryda, Safeena Kherani, Awadelkarim Mohamed *(Al Jalila Children’s Speciality Hospital, Dubai)*; Ferial Mohamed Ali Abbas *(Dubai Hospital, Dubai)*; Diary Mohammed *(Latifa Women and Children Hospital, Dubai)*; Ehab Aldlyami *(Medcare Orthopaedic and Spine, Dubai)*; Rakesh Kundra, Mohamed Mashhour *(Mediclinic City Hospital Dubai, Dubai)*; Mohamed Mashhour *(Mediclinic Wellcare Hospital, Dubai)*; Antony Louis Rex Michael *(Neurospinal Hospital, Dubai)*; Hayder Alsaadi *(Rashid Hospital, Dubai)*; Kareem S. Khalil *(Al-Qassimi hospital, Sharjah)*. |
| **United Kingdom**: Rachel Dbeis, Shafaque Shaikh *(Aberdeen Royal Infirmary, Aberdeen)*; Jenny Ferry *(Nevill Hall Hospital, Abergavenny)*; Aiman Jamal, Haleema Siddique *(Stoke Mandeville, Wycombe General, Aylesbury)*; Rishi Das, Nikhil Ponugoti *(Basingstoke and North Hampshire Hospital, Basingstoke)*; Sivesh K Kamarajah *(Queen Elizabeth Hospital Birmingham, Birmingham)*; Pornjittra Rattanasirivilai, Asma Sultana *(East Lancashire Hospitals NHS Trust, Blackburn)*; Frances Mosley *(Bradford Royal Infirmary, Bradford)*; Matthew Chan *(Southmead Hospital, Bristol)*; Antony Bateman, Gareth Davies-Jones *(University Hospitals of Derby and Burton, Burton upon Trent)*; Fanourios Georgiades, Grant D. Stewart *(Addenbrooke’s Hospital, Cambridge)*; Navid Ahmadi, Aman Coonar *(Royal Papworth Hospital, Cambridge)*; Mariam Baig, Chetan Khatri, Arthika Surendran *(University Hospitals Coventry and Warwickshire NHS Trust, Coventry)*; Julian Sonksen *(Russell’s Hall Hospital, Dudley)*; Robert Sinnerton *(Ninewells Hospital, Dundee)*; Caitlin Brennan *(Royal Infirmary of Edinburgh, Edinburgh)*; Gemma Faulkner, Michael Greenhalgh *(Royal Bolton Hospital, Farnworth)*; Hannah Emerson, Kiran Singisetti *(Gateshead Health NHS Foundation Trust, Gateshead)*; Joshua Totty *(Hull University Teaching Hospitals NHS Trust, Hull)*; Michael Wilson *(Forth Valley Royal Hospital, Larbert)*; Terence Lo *(St James’s University Hospital Leeds, Leeds)*; Harriet Corbett, Ijeoma Okonkwo *(Alder Hey in the Park, Liverpool)*; Gill Arbane, Kariem El-Boghdadly *(Guy’s and St Thomas’ Hospitals, London)*; Cyrus Kerawala *(The Royal Marsden NHS Foundation Trust, London)*; Chetan Parmar *(The Whittington Hospital, London)*; Tom Abbott, Michael Bath, Funlayo Odejinmi *(Whipps Cross University Hospital, London)*; Jayesh Sagar, Rishi Talwar *(Luton and Dunstable University Hospital, Luton)*; Samuel Newman *(Royal Lancaster Infirmary, Morecambe)*; John Hammond, John Moir *(Newcastle Upon Tyne Hospitals NHS Foundation Trust, Newcastle upon Tyne)*; Natalie Duric, Tamas Szakmany *(Royal Gwent Hospital, Newport)*; Ahmar Iftikhar Talib, Mina Youssef *(Norfolk and Norwich University Hospital, Norwich)*; Christopher Lewis-Lloyd *(Nottingham City Hospital, Nottingham)*; Christopher Lewis-Lloyd *(Queens Medical Centre, Nottingham)*; Mariam Lami *(John Radcliffe Hospital, Oxford)*; Khurram Ayub, Benjamin Dean *(Nuffield Orthopaedic Centre, Oxford)*; Supriya Balasubramanya, Sathya Lakpriya, Luke Rogers *(Derriford Hospital, Plymouth)*; Paul Turner *(Royal Preston Hospital, Preston)*; Mark Maher *(Royal Berkshire Hospital, Reading)*; Kohila Sigamoney *(Salford Royal Hospital, Salford)*; John Edwards *(Sheffield Teaching Hospital NHS Foundation Trust, Sheffield)*; Jihène El Kafsi, John Hardie *(Frimley Health NHS FT - Wexham Park, Slough)*; David Johnson *(Stepping Hill Hospital, Stockport)*; Christin Henein *(Sunderland Eye Infirmary, Sunderland)*; Marianne Hollyman *(Musgrove Park Hospital, Taunton)*; Ketan Agarwal, Simon Powell *(Wirral University Teaching Hospital, Wirral)*; Govind Singh Chauhan *(Royal Wolverhampton NHS Trust, Wolverhampton)*. |
| **United States**: Rakesh Patel *(Johnston Memorial Hosptital, Abingdon)*; Joel Gagnier *(University Of Michigan Medical Center, Ann Arbor, MI)*; Heather Carmichael *(University of Colorado Anschutz Medical Campus (CU Anschutz), Aurora)*; Kristofor A. Olson *(Dell Seton Medical Center at the University of Texas, Austin)*; Eric Etchill *(Johns Hopkins Hospital, Baltimore, MD)*; Joseph Incorvia *(Boston Children’s Hospital, Boston)*; Sameer Hirji *(Brigham and Women’s Hospital, Boston)*; Matthew Naunheim *(Massachusetts Eye and Ear, Boston)*; Frederick Drake *(Boston Medical Center, Boston, MA)*; Haytham Kaafarani *(Massachusetts General Hospital, Boston, MA)*; Caroline Reinke *(Atrium Health Carolinas Medical Center, Charlotte)*; Anna Alecci *(Rush University Medical Centre, Chicago, IL)*; Dennis Vaysburg *(University of Cincinnati Medical Center, Cincinnati)*; Jennifer Rodriquez *(Memorial Hospital, Colorado Springs)*; Emily Shih *(Baylor University Medical Center, Dallas)*; Vin Shen Ban *(University of Texas Southwestern, Dallas)*; Julia Coleman *(Denver Health, Denver)*; Henry E. Rice *(Duke University Medical Center, Durham, NC)*; Krista Kaups *(University of California San Francisco (UCSF) - Fresno, Fresno)*; Emmanouil Giorgakis *(University of Arkansas for Medical Sciences, Little Rock)*; Maggie DiNome *(University of California Los Angeles, Los Angeles)*; Neal Bhutiani *(University of Louisville Hospital and Norton Hospital, Louisville)*; Omar Alnachoukati *(Medical Center of the Rockies, Loveland, Colorado)*; Brittany Bankhead-Kendall *(University Medical Center Lubbock, Lubbock, Texas)*; Taylor Aiken, Thomas Diehl *(University of Wisconsin, Madison)*; Ankush Gosain *(Le Bonheur Children’s Hospital, Memphis, MA)*; Rishi Rattan *(University of Miami Hospital, Miami, FL)*; Muhammad Owais Abdul Ghani *(Vanderbilt University Medical Center, Nashville)*; Nensi Melissa Ruzgar *(Yale New Haven Hospital, New Haven, CT)*; Anna Liveris *(New York City Health and Hospitals - Jacobi Medical Center, New York)*; Nina Glass *(The University Hospital, Newark, NJ)*; Charu Paranjape *(Newton Wellesley Hospital, Newton, MA)*; Theresa Chin *(University of California Irvine, Orange)*; Antonio Meola *(Stanford Health Care, Palo Alto, CA)*; Kristina Nicholson *(University of Pittsburgh Medical Center, Pittsburgh, PA)*; John Squiers *(Baylor Scott & White The Heart Hospital, Plano, Texas)*; Stephanie Lueckel *(The Rhode Island Hospital, Providence, RI)*; Janani Reisenauer *(Mayo Clinic, Rochester, MN)*; Rachael Callcut *(UC Davis Medical Center, Sacramento)*; Ahmed Mansour *(UT Health San Antonio (UTHSA), University Hospital (UHS), San Antonio)*; Allison Berndtson *(University of California San Diego, San Diego)*; Lucy Kornblith *(University of California San Francisco, UCSF (use separate entry for Fresno), San Fransisco)*; Sara Seegert *(ProMedica Toledo Hospital, Toledo)*; Paulo Martins *(UMass Memorial Hospital, Worcester, MA)*. |
| **Yemen, Rep.**: Hamza Al-Naggar, Mohammed Al-Shehari *(Al-Thawra Modern General Hospital, Sana’a)*; Ibrahim Al-Raimi *(University of Science & Technology Hospital, Sana’a)*. |
| **Zimbabwe**: Allan Ngulube *(Mpilo Central Hospital, Bulawayo)*; Maphios Siamuchembu *(United Bulawayo Hospitals, Bulawayo)*; Willard Mushiwokufa *(Gweru Provincial Hospital, Gweru)*; Busisiwe Mlambo *(Parirenyatwa hospital, Harare)*; Simbarashe Chinyowa *(Sally Mugabe Central Hospital, Harare)*. |

#### Local Collaborators:

| **Albania**: Ervis Agastra *(Regional Hospital of Durres, Durres)*; Enton Bollano, Kostandin Gjyli, Dariel Thereska *(University Hospital Center Nene Tereza, Tirana)*; Irida Dajti, Jola Kerpaci, Enxhi Vrapi, Lorena Zijaj *(University hospital Koco Gliozheni, Tirana)*. |
| --- |
| **Algeria**: Bahraoui Djahida, Belabbes Fatima zohra, Kouidri Khadidja *(Ibn sina, Adrar)*; Oussama Bali, Nassim Benallel, Ismahene Lalmi *(Public hospital establishment of Sig, Mascara)*; Meriem Abdoun, Nesrine Aouabed, Souad Bouaoud, Kamel Bouchenak, Assia Haif, Zineddine Soualili *(Saadna Mohamed Abdnour (Abdnoor) Teaching Hospital, Setif)*. |
| **Argentina**: Joaquin Bastet, Agustín Bianco, Daniel Capitaine, Jorge Centeno Lozada, Carlos Esquivel, Rodrigo Figueroa, Manuel Garcia, Pablo Martín García, José Ignacio Gerchunoff, Fernando Martinez lascano, Jose Mondino, María Emilia Muriel, Carmignani Pablo, Martin Passadore, Alejandra Tornini, Rogelio Traverso, Lara Vargas, Gerardo Zanoni *(Sanatorio Allende - Sede Cerro, Allende, Cordoba)*; Norberto Berber, Estefanía Cotta, Marcela Di Vincenzo, Esteban sebastian Gallino, Cecilia Gigena, Bianca Grassano, Veronica Laudani, Ignacio Lugones, Constanza Madrid, Maximiliano Maricic, Antonio Alberto Martinez, Andres Rosso, Pablo Scher, Sofia Tachella, Damaris Idara Anabel Zezular *(Hospital General de Niños Pedro de Elizalde, Buenos Aires)*; Carla Abuawad, Agustin Albani Forneris, Carola Allemand, Laura Gisela Alvarez Calzaretta, Luis Boccalatte, Jorge G. Boretto, Rocio Boudou, Rodrigo Brandariz, Martin Buljubasich, Arturo Burchakchi, Martin Buttaro, Juan Pablo Campana, Virginia Cano Busnelli, Tomas Carminatti, Agustina Florencia Castro Lalín, Julian Cereghini, María Sol Crespi Amor, Roberto Sebastián Croattini, Maria Sol Fernandez, Marcelo Figari, Uriel Fraidenraij, Diego Gallegos, Agustín Maria García-Mansilla, Marcos Gonzalez, Matias Ignacio Gonzalez, Esteban Gonzalez Salazar, Jeremias Goransky, Guillermo Hernandez Gauna, Felipe Higuera, Fernando Holc, Esteban Gabriel Jauregui, Juan Larrañaga, Juan Liyo, Lionel Llano, Pablo Lobos, Emilia Luzzi, Gustavo Mastroianni, Santiago Miguel Mata-Suarez, Horacio F. Mayer, Santiago Mc Loughlin, Efrain Mendoza, Ricardo Esteban Mentz, Pedro Mercado, Pablo Moyano, Florencia Noll, Diego Odetto, Agustina Rene Oliva, Rafael Perez Vidal, Catalina Poggi, Eduardo Jorge Premoli, María Lourdes Ramos, Patricio Rosas, Luciano Rossi, Gustavo Rossi, Jose Saadi, Jordán Scherñuk, Pablo Slullitel, María Verona Stang, María Victoria Taboada, Sebastian Tirapegui, Constanza Uffelmann, Carlos Vaccaro, Roberto Vagni, Ana Clara Valerio, Celeste Soledad Zarratea *(Hospital Italiano de Buenos Aires, Buenos Aires)*; Carina Chwat, Silvina Montal, Brian Morris, Pedro Valdez *(Hospital Universitario Austral, Buenos Aires)*; Fernando Diaz-Couselo, Ivana Ines Pedraza Salazar, Luciana Sabatini *(Instituto Oncológico Alexander Fleming, Ciudad Autónoma Buenos Aires)*; Fernando Andres Alvarez, Micaela Avila, Nicole Benitez Benitez, Nicolás Bruera, Marcelo Doniquian, Manuel Gielis, Julian Liaño, Florencia Llahi, Facundo Mandojana, Walter Páez, Diana Alejandra Pantoja Pachajoa, Matias Parodi, Héctor Picon molina, Agustin Pinsak, German Viscido *(Clinica Universitaria Reina Fabiola, Cordoba)*; Roberto Badra, Diego Belisle, Hernan Borla, Georgina Eberle, Agustin Esteban, Carlos Ignacio Ferrero, Micaela Furlan, José Sebastian García, Lucas Granero, Mariel Henzenn, Rodrigo Juaneda, Sergio Martin Lucchini, Esteban Politi Vidal, Guillermo Romero Reyna, José Gabriel Yaryura Montero *(Sanatorio Allende - Sede Nueva Cordoba, Cordoba)*; Maria Mercedes Caubet, José Luis D’Addino, Agustin Duro *(Hospital Prof Dr Bernardo A Houssay, Provincia de Buenos Aires)*; José Alfredo Calderón Arancibia *(Hospital Público Materno Infantil de Salta, Salta)*. |
| **Aruba**: Deepu Daryanani, Martijn Gosselink, Alex Ponson *(Dr. Horacio E Oduber Hospital, Oranjestad)*. |
| **Australia**: Mohamed Afzal, Mathew Amprayil, Mark Brooke-Smith, John Chen, James Grantham, Benjamin Gricks, Amanda Hii, Nikhil Kundu, David Liu, Charles Livingston, Matthew Marshall-Webb, Christina McVeay, Hamish Moore, Gavin Nair, Bee Shan Ong, Dominic Parker, Victoria Rudolph-Stringer, Malgorzata Szpytma, Ravi Vissapragada, Melissa Yun Wee, Geoffrey Yuet Mun Wong, Xuanyu Zhou *(Flinders Medical Centre, Adelaide)*; Dylan Richard Barnett, Melissa Bochner, John Bolt, Karel Buddingh, Brendon Coventry, Sean Davis, Joseph Dawson, Stuart Denham, Christopher Dobbins, Nagendra Dudi-Venkata, Robert Fitridge, Siang Wei Gan, Izhar-Ul Haque, Matheesha Herath, Shivangi Jog, Harsh Kanhere, Hidde M Kroon, Beatrice Kuang, Yick Ho Lam, Virginia Lambert, Alicia Lim, Grace Maina, Cea-Cea Moller, Eu Nice Neo, Eu Ling Neo, Alain Nguyen, Shalvin Prasad, Jennifer Roy, Christine Russell, Tarik Sammour, Nicholas Smith, Richard Smith, Conrad Stranz, Saam Tourani, Steven Tran, Lucinda Van de Ven, Leigh Warren, Robert Whitfield, Jamie Wormald, Yijie Yin *(Royal Adelaide Hospital, Adelaide)*; Christopher Bierton, Benjamin Cribb, Anthony Farfus, Katarina Foley, Daniel Ong, Matthew Watson *(The Queen Elizabeth Hospital, Adelaide)*; John Barker, Jacob Hampton, James Karam, Anya Rugendyke, Tyson Zhang *(Armidale Rural Referral Hospital, Armidale)*; Carolyn Vasey, Eleanor Watson *(Ballarat Base Hospital, Ballarat)*; Eduardo Apellaniz, Adam Frankel, Lara Gahan, Glen R Guerra, Amanda Liesegang, Nicholas Lutton, Andrew Maurice, Scott Miron, Aveechal Prasad, Ashleigh Sercombe, Thanusan Sivapalan, Syed Danial Syed Ahmad, Chin Li Tee, Lachlan Yaksich *(Princess Alexandra Hospital, Brisbane)*; Martin Batstone, Omar Breik, Thomas Young *(Royal Brisbane and Women’s Hospital, Brisbane)*; Michael Field, Benjamin Scott, Sean Stevens *(Colac Area Health, Colac)*; Gabriella Charlton, Si Chen, Henry Yan Chi Cheung, Neha Gauri, Ross Hayhurst, Sophie James, Seyoung Jang, Fangzhi Jia, Helen Zhang *(Concord Repatriation General Hospital, Concord West, Sydney)*; Randeep Aujla, Michael Finsterwald, Digby Percy, Alvin Tanaya, Anand Trivedi, Uyen Giao Vo *(Fremantle Hospital, Fremantle, Western Australia)*; Andrew Aylett, Richard Grills, Angela Holmes, Matthew Ming Kei Kwok, Anton Lambers, Leo Lambers, Haider Latif, Evania Lok, Sonal Nagra, Richard Page, Nicholas Paltoglou, Vishwakar Panuganti, Sophie Riddell, Gareth Rudock, Hannah Tan, Kirk Underwood, David Watters, Daniel Youssef *(University Hospital Geelong, Geelong)*; Ricci Amoils, Nazli Bahtigur, Savitha Bengeri, Lily Builth-Snoad, Hock Ping Cheah, Paul Chen, Kenneth Chew, Alyssa Chong, Sara Clark, Rhea Darbari Kaul, Amanda Caroline Dawson, Eliya Devan, Christina Ewington, Aleeza Fatima, Sophia Fitt, John Gaul, Paloma Ghosal, Amy Gojnich, Indu Gunawardena, Peter Hamer, Samuel Holmes, Zhen Hou, Danielle Jolly, James Dimitri Kane, Micayla Kaufman, Tanishq Khandelwal, Sukhwant Khanijaun, Kelvin Kwok, Edward Latif, Sharon Laura, Vivian Lee, Catherine Leung, Ina Liang, Peter Lin, Elizabeth Weng Yan Lun, Haili Luo, Victor Ly, Jolande Ma, Brooke Macnab, Richard McGee, Stephanie Miles, William Munro, Harry Narroway, Tin Yau Ngan, Anthony Noor, Upuli Pahalawatta, Melissa Park, Cameron Parkin, Rita Poon, Shanmugam S Somasundaram, Claudia Saab, Renwick Simpson, Dana Steinel, Emily Taylor, Adrian Tchen, Shu Thong, Kelvin Tran, Kevin Tree, Lucille Vance, Ken Wong, Victor Yu, Michael Zhang *(Gosford Hospital, Gosford)*; Aram Cox, David Chi Hau Tan, Nova Thani *(Royal Hobart Hospital, Hobart)*; Shefali Das, Taigh Macdonald, Paul Salama, Gagandeep Sandhu, Rachel Shadbolt *(Joondalup Health Campus, Joondalup, Western Australia)*; Du Phan, David Townend *(Lismore Base Hospital, Lismore)*; Alwin Chuan, Eunmaro Ju, Sang mi Lee *(Liverpool Hospital, Liverpool)*; Stephen Barnett, Daniel Cox, Hajar Hasan Kheslat, Andrew Higgins, Chloe Jamieson-Grigg, Victoria Jenkins, Brett Larner, Vijayaragavan Muralidharan, Marcos Perini, David Proud, Kirby Qin, Georgina Riddiough, Sivendran Seevanayagam, Damien M. Wu, Chris Zhao *(Austin Hospital, Melbourne)*; Sonali Aggarwal, Vinna An, Melanie Battershell, Elliot Chan, King Tung Cheung, Anna Drake, Janindu Goonawardena, Benjamin Hunt, Christopher Ip, Vikram Iyer, Anshini Jain, Joshua Kealey, Chen Lew, Christopher Seng Hong Lim, Tara Luck, Sean Mackay, Natalie Maher, Georgia Maroske, Nicholas Roubos, Shomik Sengupta, Christopher Steen, Zac Tsigaras, Zachary Tuttle, Salena Ward, Marli Williams, Enoch Wong *(Box Hill Hospital, Melbourne)*; Travis Ackermann, Evie Yeap, Jessie Zhou *(Casey Hospital, Melbourne)*; Amy Coates, Nora Mutalima, Ton Tran *(Dandenong Hospital, Melbourne)*; Ramesh Nataraja, Maurizio Pacilli, Claire Sharpin *(Monash Childrens Hospital, Melbourne)*; David Bird, Kay Tai Choy, Sarah Condron, Jurstine Daruwalla, Isabela dos Anjos, Hanna El-Khoury, Robert Fabian, Andrew Gillard, Rebecca Greenop, Russell Hodgson, Michael Issa, Sharon Lee, Krinal Mori, Nikki Petrakis, Maryum Qureshi, Danielle Sabella, Prassannah Satasivam, Niranjan Sathianathen, Sean Ezekiel Seow, Amanda Shen, Margaret Shi, Meher Tabassum, Rodrigo Teixeira, Josephine Vivian-Taylor, Jennifer Wheatley *(Northern Hospital, Melbourne)*; Alexander Heriot, Sally Shepherd, Mikael Soucisse *(Peter MacCallum Cancer Centre, Melbourne)*; Sebastian King, Brendan O’Connor, Warwick Teague *(Royal Children’s Hospital, Melbourne)*; Simon Banting, Lynn Chong, Peter Choong, Sharnel Clatworthy, Angela Cochrane, Adrian Fox, Michael Wei Hii, Anna Isaacs, Mary Ann Johnson, Brett Knowles, Andrew Newcomb, Veronique Price, Jaishankar Raman, Matthew Read, Alistair Rowcroft, Lillian Taylor, Salena Ward, Gavin Wright *(St Vincent’s Hospital, Melbourne)*; Wendy Brown, Prem Chana, Kalai Shaw *(The Alfred Hospital, Melbourne)*; Jacob Bock, Jordan Cory, Katharine Drummond, Jack Lahy, Surjit Lidder, Thomas McIntire, Benjamin Price, Claire Stark *(The Royal Melbourne Hospital, Melbourne)*; Alex Besson, Richard Gartrell, Brianne Lauritz, Ha My Ngoc Nguyen, Chui Foong Ong, Meron Pitcher, Lorna Scullion, Howard Tang, Danielle Taylor, Brigid Wolf, Justin Yeung *(Western Health - Footscray hospital and Sunshine hospital, Melbourne)*; Luke Traeger, Matthew Watson, Matthias Wichmann *(Mount Gambier and Districts Health Service, Mount Gambier)*; Amelia Davis, Amelie Maurel, Kyle Raubenheimer *(Armadale Health Service, Mount Nasura)*; Cassidy Campbell, Ashley Colaco, Maria Julia Corbetta Machado, Abbie Heffernan, Costa Karihaloo, Isabella Ludbrook, Sean SW Park, Bibi Nabeeha Peerally, Guillermo Regalo, Camila Singhai, Heidi Stevens, Sergey Vavilov *(Belmont District Hospital, Newcastle)*; Anne-Marie Aubin, Ralph Gourlay, Margaret Harris, Zhi Ying Lim, Rebecca Spring, Melissa Stieler *(Calvary Mater Newcastle, Newcastle)*; Nicholas Adamson Barnes, Ahmad Seraj Alam, Ali Alsoudani, Francesco Amico, Rebecca Anning, Zsolt J. Balogh, Alison Blatt, Scott Cairns, Jesse Carroll, Andrew Caterson, Adam Christie, Daron Cope, Tyson Dale, Grace Dennis, Anchal Duggal, Brendan Ennis, Mark Fenton, Yi Xin Joanna Fu, Ana Galevska-Dimitrovska, Jonathan Gani, Scott Gelzinnis, Madelyn Gramlick, Claudia Hadlow, Ruth Hardstaff, Munish Heer, Merran Holmes, Bridget Hone, Sophie Hu, Linna Huang, Ramiz Iqbal, Niall Jefferson, Dimithi Kasthurirathne, Stephen Kuo, Wayne Yan Lau, Hsin-Ping Liang, Daniel Lim, Rosalina Lin, Jack McDonogh, Elysse Mcilwain, Yan Joyce Ming, Christine O’Neill, Ryan James Ocsan, Benjamin Oosthuizen, Nicole Organ, James Otieno, Felicity Park, Amanda Paterson, Marisol Perez Cerdeira, Luke Peters, Josefin Petersson, Peter Pockney, Sonia Rubbo, Venesa Siribaddana, Kabilan Thurairajah, Caleb Ting, Antonia Watson, Teagan Way, Elvina Wiadji, Ellisha Willoughby *(John Hunter Hospital, Newcastle)*; Ashley Bailey, Samuel Broadbent, Viswanathan Narayanan, Samuel Stephens, Anna Wilkes, Jie Zhao *(Maitland Hospital, Newcastle)*; Mohammed Ballal, Carl D’Souza, Clara Forbes, Matthew Goss, Monique Haddleton, Jacinda Harty, Dickon Hayne, Nicole Hew, Wee Ling Koh, Shawn Lee, Shahbaz S Malik, Naseer Mohammed Abdul, Jana-Lee Moss, Toby Richards, Yi Th Ng Seow, Anand Trivedi, Uyen Giao Vo, James Wong *(Fiona Stanley Hospital, Perth)*; Leigh Archer, Nur Sabrina Binti Babe Azaman, Tina Dilevska, Jonathan Foo, Tasvinder Hans, Shirley Jansen, Mohit Kumar, Christopher Leeson, Terence Pham, Fernando Picazo Pineda, Saravanan Rajakumar, Supisara Suk-Udom, Jonathan Tan, Benjamin Thurston, Bichen Zhao *(Sir Charles Gairdner Hospital, Perth)*; Shivangi Gupta, Lachlan Hou, Nivedan Jeyamanoharan, James Leigh, Wei Shearn Poh, Michael Sala *(St John of God Midland Public and Private Hospital, Perth)*; Rebecca Crothers, Madhulika Dravid, Ben Harrison, Sumayya Islam, Conor McCartney, Henco Nel *(St John of God Subiaco Hospital, Perth)*; Natasha Pearson *(Goulburn Valley Health Hospital, Shepparton)*; Christos Apostolou, Sarit Badiani, Christophe Berney, Richard Chou, Sam Hanna, Andrew Lam, Vanessa Ma, Salonee Shubhen Phanse *(Bankstown Hospital, Sydney)*; Michael Elliott, Daniel Phung *(Lifehouse, Sydney)*; Jessica Barry, Eleanore Clark-Mackay, Jennifer Cope, Richard Halliwell *(Westmead Hospital, Westmead)*; Ji Chen, Marie Shella De Robles, Alyssa Llorando, Humaira Haider Mahin, Soni Putnis, Dominic Rao, Faisal Syed *(Wollongong Public Hospital, Wollongong)*; Daniel Abulafia, Benjamin Buckland, Timothy Cordingley, Ashe DeBiasio, Andrew Drane, Patrick Ireland, Danielle Jolly, Benji Julien, Lan-Hoa Le, Austin Yeon Suk Lee, Eu Jhin Loh, Andrew Middleton, Brienna Mortimer, Samuel Sebastian, Brooke Short, Peter Stewart, Stephanie van Ruyven, Daniel Wong, Charry Zhang, Bonnie Zhu, William Ziaziaris *(Wyong Public Hospital, Wyong)*. |
| **Austria**: Aran Leitner, Lukas Tolzman, Matthias Zitt *(Krankenhaus der Stadt Dornbirn, Dornbirn)*; Gergely Rakos, György Székely *(Krankenhaus der Barmherzigen Brüder Eisenstadt, Eisenstadt)*; Gabriel Djedovic, Ingmar Königsrainer *(Landeskrankenhaus Feldkirch, Feldkirch)*; Felix Aigner, Caterina Allmer, Barbara Herritsch, Martin Mitteregger, Christian Schauer, Gerald Seitinger, Carmen Siebenhofer, Stefan Uranitsch *(Barmherzige Brüder Krankenhaus, Graz, Graz)*; David Duller, Erwin Mathew, Christoph Skias *(Krankenhaus der Elisabethinen, Graz)*; Alexandros Andrianakis, Armin Belarmino, Petra Brinskelle, Christoph Castellani, Tina Cohnert, Melanie Fediuk, Andrea Fink, Clemens Holzmeister, Judith Kahn, Josip Kresic, Andreas Leithner, Joerg Lindenmann, David Lumenta, Birgit Michelitsch, Saulius Mikalauskas, Sebastian P. Nischwitz, Paul Puchwein, Andrej Roj, Peter Schemmer, Georg Singer, Freyja-Maria Smolle-Juettner, Holger Till, Axel Wolf *(Medical University of Graz, Graz)*; Tibor Oliver Andraschofsky, Rafael Angerer, Daniel Arco, Claudia Kaufmann, Nura Kilic, Peter Widschwendter *(Landeskrankenhaus Hall, Hall in Tirol)*; Reinhard Angermann, Marlies Bauer, Nicole Bergmann, Christian Freyschlag, Martin Gisinger, Can Gollmann-Tepeköylü, Michael Graber, Alexander Haim, Carina Harasser, Bettina Härter, Jakob Hirsch, Markus Hofer, Johannes Holfeld, Anna Lena Huber, Martina Kralinger, Dietmar Krappinger, Irmgard Elisabeth Kronberger, Marlene Kuen, Michael Liebensteiner, Franka Messner, Alex Messner, Felix Naegele, Marijana Ninkovic, Yvonne Nowosielski, Antonia Osl, Cornelia Ower, Leo Pölzl, Teresa Rauchegger, Daniel Reimer, Johannes Riecke, Anton H. Schwabegger, Filipp Sokolovski, Anna Strimmer, Markus Süss, Martin Thaler, Julian Umlauft, Claus Zehetner, Alain G. Zeimet *(Innsbruck Medical University, Innsbruck)*; Jakob Allerstorfer, David Haslhofer, Daniel Hofer, Matthias Luger, Lorenz Pisecky, Nikolaus Poier, Nina Rubicz, Christoph Schmolmüller, Paul Zwittag *(Kepler University Hospital, Johannes Kepler University, Linz)*; Tobias Rossmann, Francisco Ruiz-Navarro, Harald Stefanits *(Kepler University Hospital, Johannes Kepler University of Linz (Neuromed Campus), Linz)*; Hend Elsayed, Peter Habertheuer, Tereza Hajkova *(Konventhospital Barmherzige Brueder, Linz)*; Wolfgang Loidl, Ferdinand Luger, Amadeus Windischbauer *(Ordensklinikum Linz Elisabethinen, Linz)*; Ines Fischer, Reinhold Függer, Patrick Kirchweger, Thomas Saini *(Ordensklinikum Linz GmbH Barmherzige Schwestern, Linz)*; Julian Berger, Wolfgang Fraz, Arastoo Nia *(Landesklinikum Neunkirchen, Neunkirchen)*; Mihaly Kenez, Margit Nichita *(Krankenhaus Oberpullendorf, Oberpullendorf)*; Astrid Magele, Thomas Mayr, Chiara Noe *(Universitätsklinikum Sankt Pölten, Pölten)*; Reinhard Bittner, Kurosch Borhanian, Isabella Dornauer, Klaus Emmanuel, Ana Gabersek, Antonia Gantschnigg, Michael Grechenig, Ricarda Gruber, Jörg Hutter, Tarkan Jäger, Oliver Koch, Michael Lechner, Lisa Manzenreiter, Franz Mayer, Iris Muehlbacher, Jaroslav Presl, Daniel Rezaie, Philipp Schredl, Martin Varga, Michael Weitzendorfer, Angela Wimmer *(Paracelsus Medical University Salzburg, Salzburg)*; Eberhard Brunner, Michael de Cillia, Michaela Gruber, Martin Grünbart, Elmar Heinrich, Hannes Hoi, Vanessa Kemmetinger, Christof Mittermair, Victoria Mosshammer, Christian Obrist, Peter Paal, Judith Roesch, Elisabeth Russe, Tobias Schaetz, Jan Schirnhofer, Karl Schwaiger, Gottfried Wechselberger, Helmut Weiss *(Saint John of God Hospital Salzburg, Salzburg)*; Maximilian Lanner *(Kardinal Schwarzenberg Klinikum, Schwarzach im Pongau)*; Alf-Dorian Binder, Thomas Gürtler *(Universitätsklinikum Tulln, Tulln)*; Marcus Fink, Daniel Reichhold, Radoslava Stoyanova *(Barmherzige Schwestern Krankenhaus Wien, Vienna)*; Klara Beitl, Martin H. Bernardi, Philip Datler, Christopher Dawoud, Alex Farr, Philipp Foessleitner, Christoph Grimm, Felix Harpain, Mir Alireza Hoda, Simone Holawe, Marlene Kranawetter, Johannes Ott, Alexandra Perricos, Maximilian Pesta, Robert Pillerstorff, Christian Reiterer, Stefan Riss, Klara Rosta, Georg Scheriau, Edda Tschernko, Rene Wenzl, Dominik Wiedemann, Peter Wohlrab, Bernhard Zapletal, Matthias Zimmermann, Daniel Zimpfer *(General Hospital of Vienna, Vienna)*; Oliver Findl, Andreea Fisus, Manuel Ruiss *(Hanusch Hospital, Vienna)*; Melanie Komaz, Nikolaus Meindl, Florian Primavesi, Karl Heinz Stadlbauer, Stefan Stättner, Florian Steiner *(Salzkammergut Klinikum Vöcklabruck, Vöcklabruck)*; Ondrej Cerny, Eva Falkensammer, Hans Knotzer, Paul Köglberger, Bernhard Poidinger, Clemens Georg Wiesinger *(Klinikum Wels-Grieskirchen GmbH, Wels)*; Johannes Burtscher, Sebastian Rath, Felipe Trivik-Barrientos *(Landesklinikum Wiener Neustadt, Wiener Neustadt)*. |
| **Azerbaijan**: Gunay Aliyeva, Vuqar Behbudov, Gurbankhan Muslumov, Natiq Zeynalov *(Scientific Center of Surgery named M.Topchubashov, Baku)*. |
| **Bahrain**: Mahmood Alam, Fatema Alfayez, Fayza Haider, Batool Hasan, Maryam Mahdi, Husain Mulla, Kawthar Qader, Amr Saeed, Ahmed Shirazi *(Salmaniya Medical Complex, Manama)*; Aysha Albastaki, Nuha Birido, Hiba Hameed Chagla, Abdulla Dawaishan, Baheya Dawaishan, Abeer Farhan, Isam Juma, Asher Khan, Aqsa Mohammad eqbal Patel, Madhu Srinivasan, Fatema Waheed Akbar Mohamad Akbar Nawab Deen *(King Hamad University Hospital, Muharraq)*; Seemal AbdulQadir, Abdulla Jabr, Fauzia Maqsood *(Awali Hospital, Riffa)*; Noora Almoosa, Mai Naseer *(BDF hospital, West Riffa)*. |
| **Bangladesh**: Mohammed Shadrul Alam, AKM Khairul Basher, Shahnoor Islam *(Bangladesh Medical College Hospital, Dhaka)*; S.M. Nazmul Islam, Sabbir Karim, Ashrarur Rahman Mitul *(Dhaka Shishu (Children) Hospital, Dhaka)*; Muntasir Faisel, Sadia Khan, Iftekhar Ibne Mannan, Nawshin Nazia *(Popular Medical College Hospital, Dhaka)*. |
| **Barbados**: Malissa Bentham, Sasha Corbin, Alex Doyle, Asha Eastmond, Amelia Haynes, Margaret O’Shea, Greg Padmore, Emil Phillips, Keisha Walkes *(Queen Elizabeth Hospital, Bridgetown)*. |
| **Belarus**: Evgeniy L. Artyushkov, Mark L. Kaplan, Vladislav Straltsov *(Gomel City Clinical Hospital No 3, Gomel)*; Yauheniya Litvina, Alexei Lyzikov, Victor E. Tihmanovich *(Gomel Regional Clinical Cardiology Center, Gomel)*. |
| **Belgium**: Mafalda Borges, Tim Brits, Nicolas De Hous, Stefan De Wachter, Niels Komen, Tomas Menovsky, Xavier Mortiers, Dorien Vermeulen, Nils Vleminckx, Dirk Ysebaert *(University Hospital Antwerp, Antwerp)*; Julie Bontinck, Danny Bulthé, Bert Dhondt, Ricky Rasschaert, Anne-Sophie Van Haver *(AZ Rivierenland, Bornem)*; Koen Van Belle, Laurence Verstraeten, Manon Vounckx *(Europe Hospitals, Brussels)*; Serge Cappeliez, Sébastien D’ulisse, Yasmine De Bruyne, Badih El Nakadi, Tessely Heloise, Matteo Luisetto, Sotirios Marinakis, Maxime Maton, Manon Pigeolet, Claire Viste *(CHU de Charleroi, Charleroi)*; Elke Van Daele, Gabrielle van Ramshorst, Mathieu Vandeputte *(University Hospital of Ghent, Gent)*; Marc Duinslaeger, Daniel Jacobs-Tulleneers-Thevissen, Yanina Jansen, Ward Janssens, Rastislav Kunda, Nouredin Messaoudi, Michael Ruyssers, Martijn Schoneveld, Jasper Stijns, Ellen Van Eetvelde, Marian Vanhoeij *(UZ Brussel, Jette)*; Wouter Oosterlinck, Jef Van den Eynde, Raf Van den Eynde *(UZ Leuven, Leuven)*; Ahmed M. Chaoui, Nicolas Flamey *(AZ Delta, Roeselare)*. |
| **Benin**: Marcellin Akpla, Cyrille Kpangon, Souliath Lawani *(Centre Hospitalier Universitaire de Zone de Suru-Lére, Cotonou)*; Hermann Agossou, Hulrich Aouagbe Behanzin, Covalic Bokossa *(Hopital de Menontin, Cotonou)*; Mouhamed Agbadebo, Hubert Dewanon, Hugues Yome *(Hôpital de Zone de Dassa-Zoumè, Dassa-Zoumè)*; Alassan Boukari, Oswald Gbehade, Sèmèvo Romaric Tobome *(Centre Hospitalier Départemental de l’Atacora, Natitingou)*; Labissi Francois Amossou, Francis Dossou, Ismaïl Lawani *(Centre Hospitalier Universitaire et Departemental Oueme Plateau, Porto Novo)*. |
| **Bosnia and Herzegovina**: Tatjana Barišić, Miran Boras, Zdrinko Brekalo, Ivana Čuljak Blagojević, Ana Damjanović, Vedran Dragisic, Ana Dugandžić Šimić, Filip Gunaric, Martin Kajic, Darko Knežević, Tanja Krešić, Valentina Lasić, Ludvig Letica, Vlatka Martinovic, Iva Mikulic, Josip Miskovic, Hrvoje Pehar, Pejana Rastović, Irena Sesar, Violeta Šetka- Čuljak, Martina Soljic, Dejan Tiric, Vajdana Tomić, Anja Vasilj *(SKB University Clinical Hospital Mostar, Mostar)*; Anis Cerovac, Igor Hudic *(University Clinical Center Tuzla, Tuzla)*. |
| **Brazil**: Renato Aguera Oliver, Felipe Azenha Lamonica, Jorge De Medeiros, Henrique Donizetti Bianchi Florindo, Rodolfo Jose Favaretto Filho, Fernanda Costa Pereira, Rodrigo Rodrigues, Antonio Antunes Rodrigues Junior, Fernanda Ruiz de Andrade, Maisa Salvetti, Débora Schalge Campioto, Mayra Tuboi Lamonica, Gustavo Urbano, Ricardo Villela Prado *(Hospital Estadual Américo Brasiliense, Américo Brasiliense)*; Nivaldo Alonso, Carlos Ferreira dos Santos, Cristiano Tonello *(Hospital de Reabilitacao de Anomalias Craniofaciais de Bauru, Bauru)*; Robinson Esteves Pires, Ricardo Fernandes Rezende, Igor Reis *(Felicio Rocho, Belo Horizonte)*; Fernando Augusto Lima Marson, Erick Pires Ferreira, Camila Vantini Capasso Palamim *(Hospital Universitário São Francisco de Assis na Providência de Deus, Bragança Paulista)*; Marcelo Brandao, Tiago Henrique de Souza, Rebecca Maunsell *(Hospital De Clinicas da Unicamp, Campinas)*; Alfeu Accorsi Neto, Guilherme Accorsi, Murilo Francisco Fernandes, Camila Machareth, Barbara Viegas Moura *(Hospital Padre Albino, Catanduva)*; Vanessa Dias, Luciano Guarienti, Lia Regina de Sampaio, Cristiano Vendrame *(Supera Oncologia - Hospital Regional do Oeste, Chapeco)*; Tatiane Amorim Coelho, Karin Becker, Andre Dias, Camila Girardi Fachin, Monica Maria Gomes-da-Silva, Isabela Moraes, Amanda Pinto, Adriano Seikiti Stychnicki *(Complexo Hospital de Clínicas da UFPR, Curitiba)*; Nathalia Siqueira Julio, José Mauro dos Santos, Humberto Fenner Lyra Junior, João Carlos Costa de Oliveira, Tiago Rafael Onzi, Marlus Tavares Gerber *(Hospital Universitário Professor Polydoro Ernani de São Thiago- HU/UFSC/EBSERH, Florianopolis)*; Filipe Osni Coelho *(Maternidade Carmela Dutra, Florianópolis)*; Janaína Carla da Silva, Carolina Panis, Daniel Rech *(Ceonc - Hospital de Câncer de Francisco Beltrão, Francisco Beltrão-PR)*; Melissa Avelino, Lais Botacin, Mateus Capuzzo Gonçalves *(University Federal Hospital, Goiânia)*; Gustavo Mendonça Ataíde Gomes, Igor Lima Buarque, Amanda Lira dos Santos Leite, Laercio Pol-Fachin, Tainá Santos Bezerra, Aldo Vieira Barros *(Hospital Santa Casa de Misericordia de Maceio, Maceio)*; Alcimar lavareda dos Santos Junior Alcimar, Robson Amorim, Maria Eduarda Bellotti Leão, João José Corrêa Bergamasco, Cintia Cardoso Pinheiro, Rubem Alves Da Silva Neto, Elaine Francisca De Araújo, Lilian Guimaraes, Tatiana Liborio-Kimura, Isabelle Melo da Camara, Victor Ripardo Siqueira, Juan Rodriguez, Jeancarllo Silva, Rubem Alves Silva Junior *(Hospital Universitário Getúlio Vargas, Manaus)*; Cláudio Cardoso, Andre Guimaraes, Wislene Sarajane Moreira Alves, Agna Soares Da Silva Menezes *(Hospital Dilson Godinho, Montes Claros)*; Marcelo Araujo, Kattiucy Brito, Josie Marcelle Lira Albuquerque *(Hospital Universitário Antonio Pedro, Niteroi)*; Jairo Alberto Dussan-Sarria, Andressa Souza, Andre Ricardo Stüker *(Hospital Unimed, Novo Hamburgo)*; Tiago Bresciani, Luciana Cadore Stefani, Leandro Totti Cavazzola, Tainá Costa, Matheus Dasqueve, Gustavo De Bacco Marangon, Otávio Ritter Silveira Martins, Josy Rodrigues, Guilherme Roloff Cardoso, Brasil Silva Neto, Tilaê Soares, Aline Zanella *(Hospital de Clínicas de Porto Alegre, Porto Alegre)*; Lucas Torelly Filippi, Enilde Eloena Guerra, Ricardo Pedrini Cruz *(Hospital Nossa Senhora da Conceição, Porto Alegre)*; Antonio Nocchi Kalil, Gustavo Laporte, Moacyr Salem *(Irmandade da Santa Casa de Misericórdia de Porto Alegre, Porto Alegre)*; Murilo de Lima Brazan, Caio Antonio de Campos Prado, Elaine Christine Dantas Moises, Hilda Satie Suto, Alice Gadotti Yasuda, Ana Carolina Tagliatti Zani *(Centro de Referencia da Saude da Mulher de Ribeirao Preto - Mater, Ribeirao Preto)*; Roberto Cardoso Cardoso dos Santos, Geraldo Duarte, Edwaldo Edner Joviliano, Carolina Lourenço Gomes dos Santos, Silvana Maria Quintana, Edwin Tamashiro, Fabiana Cardoso Pereira Valera, Marília Veccechi Bijos Zaccaro *(Clinics Hospital, Ribeirao Preto Medical School, University of Sao Paulo, Ribeirao Preto)*; Isis Andreotti, Leonardo Lima, Wilson Salgado Jr. *(Hospital Estadual de Ribeirao Preto, Ribeirao Preto)*; Alonço da Cunha Viana Júnior, Lana Moutinho, Cora Pichler de Oliveira, Isabela Trindade Martins, Isabela Vieira Toledo *(Hospital Naval Marcílio Dias, Rio de Janeiro)*; Marco Antonio Correa Guimaraes-Filho *(Pedro Ernesto University Hospital, Rio de Janeiro)*; Rodrigo Arrivabeno, Claudio Bovolenta Murta, Danniel Frade Said, Jose Pontes Junior, Felipe Guimarães Pugliesi *(Hospital Brigadeiro, Sao Paulo)*; Paulo Gregorio, Alessandro Mariani, Fabio Minamoto *(Hospital das Clinicas da Faculdade de Medicina da Universidade de São Paulo, Sao Paulo)*; Juliana Lourenço da Silva, Bruno Muller, Caio Zanon *(Hospital Santa Paula, Sao Paulo)*; Vladimir C. Carvalho, Ana Lucia Munhoz Lima, Priscila R Oliveira, Jorge dos Santos Silva *(Instituto de Ortopedia e Traumatologia do Hospital das Clinicas da Faculdade de Medicina da Universidade de Sao Paulo, Sao Paulo)*; Mariana Faccini Teixeira, Luiz Paulo Kowalski, Marco Kulcsar, Leandro Matos, Kamilla Schmitz Nunes *(Instituto do Cancer do Estado de São Paulo, Sao Paulo)*; Joel Abdala Junior, Emne Abdallah, Samuel Aguiar Júnior, Glauco Baiocchi, Heloisa Galvão do Amaral Campos, Genival Barbosa Carvalho, Igor Correia de Farias, Cassia da Silva, Fabio Fernando Eloi Pinto, Luciana Facure, André Godoy, Jefferson Gross, Felipe José Fernandez Coimbra, Luiz Paulo Kowalski, Bruna Kupper, Fernanda Leite, Matheus de Melo Lôbo, Fabiana Makdissi, Henrique Mantoan, Narimã Marques, Tomas Marques, João Paulo Medici, Silvio Melo torres, Katheryne Merlos Garcia, Suely Nakagawa, Joao Pedreira Duprat Neto, Rafael Ribeiro Meduna, Ana Carolina Scintini Herbst, Silvana Soares Dos Santos, Bruna Tirapelli Gonçalves, Jose Guilherme Vartanian, Stenio C Zequi *(A.C. Camargo Cancer Center, São Paulo)*; Marcelo Antonini, Carolina Brienze, José Francisco Farah, Rodrigo Gonçalves, Vinicius Machado, Luis Roberto Nadal, Danilo Nadal Rodrigues, Adrieli Pansani, Maria Luiza Rocha, Marcelo Simonsen, Daniela Tsuchiya, Rafaela Vasques *(Hospital do Servidor Público Estadual - Francisco Morato de Oliveira, São Paulo)*; Jorge Amorim, Ana Alyra Carvalho, Rebeca Correia, Brena Costa dos Santos, Ronald Flumignan, Henrique Jorge Guedes Neto, Luis Nakano, Mariana Pereda, Vladimir Vasconcelos *(Hospital São Paulo, São Paulo)*; Jamile Barakat Awada, Guilherme Gava, Tiago Riuji Ijichi, Nam Jin Kim, Rafael Nunes, Fabio Pinto *(Notre Dame Intermédica - Hospital Salvalus, São Paulo)*; Thiago Henrique Sigoli Pereira, Gustavo Jardim Volpe *(Hospital Estadual Serrana, Serrana)*; Arthur Gatti, Caroline Nardi, Ramon Oliva *(Hospital Geral de Pirajussara, Taboão da Serra)*; Layze Braz de Oliveira, Herica Emilia Félix de Carvalho, Ivonizete Pires Ribeiro, Gabriel Renan Soares Rodrigues, Álvaro Francisco Lopes de Sousa *(Hospital Getúlio Vargas, Teresina)*; Michel Chebel, Paulo Henrique de Sousa Fernandes, Marcelo Augusto Faria Freitas, Juliano Rodrigues da Cunha *(Federal University of Uberlandia, Uberlandia)*; Michel Chebel, Paulo Henrique de Sousa Fernandes, Juliano Rodrigues da Cunha *(Uberlandia Medical Center, Uberlandia)*. |
| **Bulgaria**: Zeenia Ather, Dobromir Dimitrov, Emil Filipov, Muhammad Gohar, Elitsa Gyokova, Ivelina Ilieva, Tsvetomir Ivanov, Martin Karamanliev, Vasil Neykov *(University Hospital Dr Georgi Stranski, Medical University - Pleven, Pleven)*; Boyko Atanasov, Nikolay Belev, Mihail Slavchev *(University Hospital Eurohospital, Plovdiv)*; Paolina Kamenova, Kaloyan Tonev, Tsanko Yotsov *(University Hospital Medika, Ruse)*; Evguenia Hristova, Kolyo Spassov, Tsanko Tsankov *(Fifth City Hospital Sofia - 5th MBAL, Sofia)*; Dragomir Dardanov, Petar Gribnev, Manol Sokolov *(University Hospital Alexandrovska, Sofia)*. |
| **Cameroon**: James Brown, Chukwuemeka Nwegbu, John Tanyi *(Mbingo Baptist Hospital, Bamenda)*. |
| **Canada**: Heather Hurdle, Natalia Jaworska, Anthony MacLean, Joshua Ng-Kamstra, Michael Sander *(Foothills Medical Centre - University of Calgary, Calgary)*; Salim Al Riyami, Krittika Bali, David Bigam, Khaled Dajani, Angela Dell *(University of Alberta Hospital, Edmonton)*; Mehran Anvari, Rafik Bolis, David Choi, Susan Ellis, Michael Gupta, Wael Hanna, Dennis Hong, Cynthia Horner, Lea Luketic, Peter Moisiuk, Harsha Shanthanna, Yaron Shargall *(St. Joseph’s Healthcare Hamilton, Hamilton)*; Hilda Alfaro, Nawar Alkhamesi, Laura Allen, Muriel Brackstone, Eunice Chan, Davy Cheng, Jason Chui, Nelson Gonzalez, Brent Lanting, S. Danielle MacNeil, Janet Martin, Robert Mayer, Jacob McGee, Mahesh Nagappa, Nicholas Power, Agya Prempeh, Mehdi Qiabi, Hassan Razvi, Emil Schemitsch, Herman Sehmbi, Ushma Shah, Yamini Subramani, Edward Vasarhelyi, Kelly Vogt, Homer Yang *(London Health Sciences Centre and St Josephs Health Care London, London)*; Abdollah Behzadi, Amanpreet Brar, Ali Ghorbani Abdehgah *(Trillium Health Partners, Mississauga)*; Saba Balvardi, Liane Feldman, Julio Flavio Fiore Jr, Melissa Hanson, Brent Hopkins, Pepa Kaneva, Lawrence Lee, Julia Leonard *(McGill University Health Center, Montreal)*; Jad Abou-Khalil, Andre Martel, Carolyn Nessim, James Stevenson *(The Ottawa Hospital, Ottawa)*; Susan Lee, Richard Merchant, Michelle Mozel *(Eagle Ridge Hospital, Port Moody)*; Samuel Avoine, Laurence Belanger, Mathieu Belanger, Etienne Belzile, Anne-Sophie Blais, Sofia Boucher-Kovalik, Cindy Boulanger-Gobeil, Etienne Cardinal, Pierre-Olivier Champagne, Jonathan Cloutier, Simon Corriveau-Durand, Mathieu Cote, Maxime Cote, Mathilde Côté, Valérie Courval, Suzanne Demers, Christine Desbiens, Mehdi El Ouazzani, Karine Girard, Ève-Marie Girard, Karo Gosselin, Helene Khuong, Nathalie Labrecque, Eve-Lyne Langlais, Audrey Larouche, Andréane Lavallée, Madeleine Lemyre, Sarah Maheux-Lacroix, Melissa Marien, Patrick Marin, Julie Mauger, Genevieve Milot, Sylvie Nadeau, Sebastien Nguyen, Mélinda Paris, Stéphane Pelet, Marie Plante, Carole Plante, Frederic Pouliot, Marie Claude Renaud, Mathilde Sarlabous, Isabelle Schmit, Pascal St-germain, Raphaël St-germain *(Centre Hospitalier Universitaire de Québec, Québec)*; Brian Johnston, Crispin Russell *(Saint John Regional Hospital, Saint John)*; Gary Groot, Nicole Labine, Amit Persad, Hong Pham, Melissa Wood *(Saskatoon City Hospital/Royal University Hospital/St. Paul’s Hospital, Saskatoon SK)*; Riordan Azam, Najib Safieddine *(Michael Garron Hospital, Toronto)*; Amit Atrey, Andrew Beckett, Daniel Cohen, Sunit Das, Julian Daza, Cheryl Dunkerton, Yosef Ellenbogen, Ciara Hanley, Sorcha Kellett, Amir Khoshbin, Karim Ladha, Amanda McFarlan, Spencer Montgomery, Janneth Pazmino-Canizares, Hrishikesh Suresh, Duminda Wijeysundera *(St. Michael’s Hospital, Toronto)*; Antoine Eskander, Ravleen Vasdev *(Sunnybrook Hospital, Toronto)*; Maira Ahmed, Sami Chadi, Fred Gentili, Aristotelis Kalyvas, Matthias Millesi, Can Sarica, Taariq Shaikh, Leslie St. Jacques, Raha Tabasinejad, Gelareh Zadeh *(Toronto Western Hospital, Toronto)*; Marvin Hsiao, Nicole Jedrzejko, Shawn MacKenzie *(Royal Columbian Hospital, Vancouver)*; Peter Black, Claire Broe, Charlotte Dandurand, Rebecca Grey, Philemon Leung, Andrew Lindberg, Juan Mata Gutierrez, Kelly Mayson, Adam Meneghetti, Drew Phillips, John Street *(Vancouver General Hospital, Vancouver)*. |
| **Chile**: Fernando Heredia *(Clínica Universitaria de Concepción, Concepción)*; Carolina Carvajal Calderón, Daniel Alejandro Donoso Pizarro, Esteban Fernández, Maria Marta Modolo, Ángela Molero, Nastassja Mutarello, Jorge Núñez Lucic, Katherine Ochoa Gaete, Catalina Ruiz Lopez, Rina Sepúlveda, Camila Ulloa *(Hospital Barros Luco Trudeau, Santiago)*; Mauricio Barreda, Matias Dallaserra, Matías Günther Wood, Mauricio Sandoval Tobar, Julio Villanueva *(Hospital Clínico San Borja-Arriarán, Santiago)*; Francisco Garcia-Huidobro, Sofia Waissbluth *(Hospital Clinico Universidad Católica, Santiago)*; Jimena Dona, Roberto Macchiavello, Carolina Soto Diez *(Hospital de Urgencia Asistencia Pública Dr Alejandro del Río, Santiago)*; Mario I Escudero, Daniel Igor, Javier Mena, José Tomás Reyes *(Hospital San Jose, Santiago)*; Catalina Arredondo Soto, Jose Campos, Tyare Fuentes, Javier Gonzalez, Barbara Rivera *(Hospital Sótero de Río, Santiago)*; Jaime Altamirano-Villarroel, David Cohn, Luis Marin de Amesti, Ximena Mimica, Pedro Recabal, Camilo Sandoval *(Instituto Oncologico Fundacion Arturo Lopez Perez, Santiago)*; Bruno Catoia fonseca, Monica Contador, Andres Hodali, Camila Pincheira, Andrea Ramos Mantilla, Claudio Salas Garrido, Janina Torres, Marco Valenzuela *(Roberto del Río Children’s Hospital, Santiago)*. |
| **China**: Zehua Chen, Jiankun Hu, Jin Tao, Kun Yang, Yuexin Zhang *(West China Hospital, Sichuan University, Chengdu, Sichuan Province)*; Yuan Chen, Wang Cunchuan, Wah Yang *(The First Affiliated Hospital of Jinan University, Guangzhou)*; Xueli Bai, Tingbo Liang, Tao Ma *(The First Affiliated Hospital of Zhejiang University School of Medicine, Hangzhou)*; Wenhui Lou, Ning Pu, Hanlin Yin *(Zhongshan Hospital, Shanghai)*. |
| **Colombia**: Dinimo Bolivar Saenz, Camilo Caicedo, Diego Felipe Tellez Beltran *(Clinica Colsubsidio Calle 94, Bogota)*; Alejandro Escobar, Carolina Perez Granados, Lisbeth Alexandra Urueña Pinzon *(Clinica Palermo, Bogota)*; Carlos Bonilla, Joaquin Luna, Diana Santana *(Clinica Reina Sofia, Bogota)*; Carlos Bonilla, Diana Santana, Oscar Serrano *(Clínica Universitaria Colombia, Bogota)*; Andrea Garcia, Fernando Giron Luque, Nasly G. Patino-Jaramillo, Nestor Fabian Pedraza Alonso *(Colombiana de Trasplantes, Bogota)*; Laura Giselle Contreras Baquero, Nestor Augusto Muñoz Botero, Jhoana Andrea Murillo Castellanos, Camilo Ortiz Silva, Andrea Juliana Vega Calvera *(El Tunal, Bogota)*; Lina M. Acosta Buitrago, Maria Paz Bohórquez-Tarazona, Paulo Andrés Cabrera Rivera, Felipe Casas J, Valeria Cormane Alfaro, Julian Corso, Bayron Guerra, Albert Franz Guerrero-Becerra, Akram Kadamani Abiyomaa, Felix Ramon Montes, Manuel Santiago Mosquera Paz, Carlos J- Perez Rivera, Camilo Andrés Polanía Sandoval, Natalia Quintana, Carlos Fernando Roman Ortega, Carlos Santacruz, Manuela Téllez, Camilo Alejandro Velandia Sánchez *(Fundacion Cardioinfantil-IC, Bogota)*; Fernando Arias-Amézquita, Luis Felipe Cabrera Vargas, Natalia Cortes Murgueitio, Elkin Escorcia, Kemel Ahmed Ghotme Ghotme, Jorge Luis Gomez-Mayorga, Alvaro Felipe Guerrero Vergel, Gabriel Herrera-Almario, Eduardo Londono-Schimmer, Juan A. Mejia, Guillermo Monsalve, Juliana Rodriguez, Camilo Rodriguez, Juan Nicolas Rodriguez Niño, Luis Martín Rodríguez Ortegón, Daniel Sanabria *(Fundacion Santa Fe de Bogota, Bogota)*; Andres Alvarez, Arnulfo Andrade, Javier Ardila-Montealegre, David Baquero, Edgar Mauricio Barrios Vidales, María Alejandra Caicedo Giraldo, María Camila Carvajal, Maria Castillo, Maria Carolina Castillo Florez, María Angelica Cendales, Danny Conde Monroy, Henry Cortes, Mario Daniel, Jairo De la Peña, Elena Leonor Delgado-Nieto, Hernando Espitia, Carlos Figueroa Avendaño, Edgar Oswaldo Hernandez Burgos, Andres Isaza-Restrepo, German Londono, Margarita Maria Maldonado, Andrea Montenegro, Juan Carlos Navarro, Jorge Navarro-Alean, Katherine Parra Abaunza, Eliana Pulido, Erika M. Ramírez Amaya, Carlos Eduardo Rey Chaves, Natalia Andrea Rivera Rincón, William Mauricio Riveros Castillo, Lizeth Rodriguez Sanchez, Wilson Rubiano, Maria Russi, Juan Carlos Sabogal Olarte, Javier Mauricio Salgado Tovar, Maria Juliana Sanchez, Liliana Silva-Igua, David Mauricio Solano Varela, Martha Luz Torres, Paula Torres Gomez, Marcial Trillos, Ana María Vargas Patiño, Felipe Vargas-Barato, Juan pablo Villate leon, María Alejandra Wagner Useche *(Hospital Universitario Mayor, Bogota)*; Jesus Acosta, Adriana Almeciga, Luis Becerra Mendez, Rafael Jose Beltran, Ana Bonilla, Miguel Buitrago, Marino Cabrera, Lina Caicedo, Pedro Hernando Calderon Quiroz, Diego Camacho-Nieto, Carlos Andrés Carvajal Fierro, Sergio Cervera Bonilla, Sandra Diaz, Helena Facundo, Jorge Forero, Mauricio Garcia Mora, Karena Victoria Garcia Tirado, German Fabian Godoy Perez, Felipe Gonzalez, Oscar Guevara, Jairo Alonso Hernandez, David Ricardo Herrera Mora, Juan David Lalinde, Carlos Lehmann, Eduardo Leon Llanos, Byron Eduardo Lopez De Mesa Lopez, Ivan Mariño, Monica Medina, Raúl Eduardo Pinilla Morales, Angela Puerto, Ma. Andrea Quintero-Ortíz, Juliana Rendón Hernández, Juliana Rodriguez, Elio Fabio Sánchez Cortés, Alvaro Eduardo Sánchez Hernández, Diana Santana, Raul Suarez, Oscar Suescun, Lina María Trujillo, Marco Vanegas, Rodolfo Varela, Jorge Luis Velez Bernal, David Viveros-Carreño *(Instituto Nacional de Cancerologia, Bogota)*; Daniela Camargo Gómez, Angelica Fletcher, Abel Merchan *(Centro de Investigaciones Oncológicas Clínica San Diego - CIOSAD, Bogotá)*; Euler Javier Burbano Luna, Indira Cujiño, Albaro José Nieto Calvache, Mauricio Velasquez Galvis, Lina Maria Vergara Galliadi *(Fundación Valle del Lili, Cali)*; Orlando Abonia Gonzalez Abonia Gonzalez, Maria Fernanda Acuna Saravia, Roberto Arroyave, Jose Maria Barreto Angulo, Francisco Javier Bonilla-Escobar, Uriel Cardona, Ivan Castañeda Giacometto, José Luis Castillo, Diego Fernando Castillo-Cobaleda, Diego Jose Caycedo Garcia, Brenda Marcela Coll Tello, Juan Carlos Dueñas-Ramirez, Oscar Andres Escobar Vidarte, Luis Figueroa, Herney Garcia-Perdomo, Alden Gomez, Laura Gomez, Alejandro Gomez, Ana María Grande-Gil, David Guarin, Natalia Guzman, Enrique Herrera Castañeda, Marisol Hinaoui, Anuar Armando Idrobo Escobar, Christian kammerer Kammerer, Sergio Leon, Carlos Antonio Llanos Lucero, Angie López, Alexander Maximiliano Martinez-Blanco, Antonio José Montoya Casella, Ricardo Andres Niño Corredor, Sebastian Ordoñez, Diego Alfredo Palta Uribe, Andrea Carolina Perea Serna, July Ríos, Gabriel Ríos-Samper, Juan David Rivera Garcia, Juan Camilo Salcedo Moreno, John Sandoval, Guillermo Alberto Sarmiento Ramirez, Paola Andrea Tabares Romero, Ricardo Urzola, Iliana Maria Valdes-Duque, Miguel Velasquez, Lina Maria Villegas, James Zapata-Copete, José Omar Zorrilla Lara, Mauricio Zuluaga Zuluaga *(Hospital Universitario del Valle Evaristo García, Cali)*; Alejandra Echeverri Moreno, Juan Sebastian Figueroa, Rafael Figueroa - Casanova, Adolfo Enrique Gómez Ortiz, Maria Fernanda Gonzalez Mosos, Alejandro González-Orozco, Juan Jose Jaramillo Roncancio, María Camila Leyva Martínez, Monica Mosos, Juan Sebastian Ramirez, Henry Andrés Rodríguez, Juan David Saavedra Henao, María Alejandra Torrado Varón *(Clinica Avidanti - Ibague, Ibague)*; Oscar-Julián García-Montoya, Leidy Natalia Idarraga Ramírez, Lorena Ocampo *(Clínica Avidanti - Manizales, Manizales)*; Monica Cardona Marin, Ana Maria Marin Gonzalez, Carlos Andres Marulanda Toro *(SES Hospital de Caldas, Manizales)*; David Alejandro Mejia, Maria Clara Mendoza Arango, Luis Emiro Vanegas *(Hospital Universitario San Vicente Fundacion, Medellin)*; Yicel Alvarez Martinez, Sandra Aruachan Vesga, Dayana Vanessa Baron Fuentes, Alejandra Caballero salas, Eneida Diaz Martinez, Gustavo Antonio Martinez Estrada, Kelly Viloria Campo *(IMAT Oncomedica, Monteria)*; Juan Antonio Corralez Alvarez, Cesar David Galindo Regino, Angela Maria Giraldo Velasquez, Valentina Gutiérrez Perdomo, Carlos Enrique Melo Moreno, Jorman H. Tejada, Jesus Hernan Tovar, German Alirio Tovar *(Hospital Universitario Hernando Moncaleano Perdomo, Neiva)*; Edison Alexander Benavides Hernández, Jose Andres Calvache, Claudia Milena Orozco-Chamorro *(Hospital Susana Lopez de Valencia, Popayan)*; Gustavo Adolfo Angel, Christian Ali Buesaquillo, Jose Andres Calvache, Victor David Olave Montaño, Andrés Sánchez-Gómez *(Hospital Universitario San José, Popayán)*; Daniela Patricia Escalante Ureche, Jairo Martinez Garrido, Emileth Montenegro *(Clínica Avidanti Santa Marta, Santa Marta)*. |
| **Congo, Dem. Rep.**: Eben-ezer Genda *(Centre Hospitalier Saint-Vincent, Bukavu)*; Nicolas Amisi, Fabrice Eboma *(CENTRE HOSPITALIER IMEYA SECOURS, Kinshasa)*. |
| **Croatia**: Kristina Bitunjac, Karlo Grulović, Marijana Vučković *(General and Veterans Hospital ‘Hrvatski Ponos’ Knin, Knin)*; Goran Šantak *(County General Hospital Pozega, Pozega)*; Emanuel Borovic, Ana Bosak Versic, Damir Hasandić, Natasa Poldan Grabar, Suzana Srsen Medancic *(University Hospital Center Rijeka, Rijeka)*; Lucija Brkic, Sara Cokarić, Petra Pavic Palac, Zeljka Samac, Josipa Tomić, Marija Vrdoljak *(General Hospital Sibenik, Sibenik)*; Ivan Bacic, Bernarda Bakmaz, Nikolina Bratošević Vučičić, Domagoj Brzic, Samir Canovic, Emilio Dijan, Maja Grgec Dragicevic, Robert Karlo, Suzana Konjevoda, Petra Kovačević, Ivan Kovačić, Frane Markulić, Luka Matak, Jakov Mihanovic, Domagoj Morović, Gordan Perišić, Andrea Simic, Neven Skitarelić, Matea Veršić, Vanja Žufić, Matea Zuzul *(Zadar General Hospital, Zadar)*; Srdan Ante Anzic, Iva Carevic, Dubravka Heli Litvic, Duska Markov-Glavas, Tatjana Savic Jovanovic *(Children’s Hospital Srebrnjak, Zagreb)*; Goran Augustin, Jerko Biloš, Bojan Biočina, Vedrana Biosic, Dino Bobovec, Iva Botica, Boris Bumber, Petra Čerina, Ana Danic Hadzibegovic, Katarina Duric Vukovic, Hrvoje Gasparovic, Lucija Gatin, Kresimir Grsic, Ika Gugić Radojković, Ivan Jelčić, Zeljko Kastelan, Juraj Kolak, Tomislav Kopjar, Tomislav Kulis, Kristian Kunjko, Marjan Maric, Marcel Marjanović Kavanagh, Borna Milicic, Trpimir Morić, Miram Pasini, Luka Penezić, Drago Prgomet, Ratko Prstacic, Andreja Prtorić, Rudolf Radojković, Ivan Romić, Tomislav Sečan, Dora Škrljak Šoša, Juraj Slipac, Mislav Tomic, Jurica Zedelj, Toni Zekulić, Zoran Zimak *(University Hospital Centre Zagreb, Zagreb)*; Mia Lorencin, Ivica Luksic, Matija Mamic *(University Hospital Dubrava, Zagreb)*. |
| **Cuba**: Pablo Mijahil Avilés Jiménez, Norberto Miranda Espinsa, Ailén Sánchez Cruz *(Hospital Pediátrico Juan Manuel Márquez, La Habana)*. |
| **Cyprus**: Stavros A. Antoniou, Michael Kakas, Yiannis Panayiotou *(Mediterranean Hospital of Cyprus, Limassol)*; Heyam Almezghwi, Kalbim Arslan, Hasan Besim, Ali Özant, Necdet Özçay *(Near East University Hospital, Nicosia)*; Nikolaos Gouvas, Georgios Kokkinos, Panayiotis Papatheodorou, Ioanna Pozotou, Olga Stavrinidou, Anneza Yiallourou *(Nicosia General Hospital, Nicosia)*. |
| **Czech Republic**: Lukáš Burda, Michal Dosoudil, Lubomir Martinek *(Hospital & Oncological Centre Novy Jicin, Novy Jicin)*; Wladyslaw B. Gawel, Milan Lerch, Matúš Peteja, Jan Žatecký *(Slezská nemocnice v Opavě, p.o., Opava)*; Toman Daniel, Martin Formánek, Veronika Javurkova, Markéta Kepičová, Jaroslav Klat, Nicole Macečková, Petr Matousek, Ondrej Simetka, Petr Vavra, Karol Zelenik *(University Hospital Ostrava, Ostrava)*; Jan Černý, Sami Khan, Robert Lischke, René Novysedlák, Martin Přibyl, Temoore Younus *(Motol University Hospital, Prague)*; Yenuksha Amarasena, Karel Klíma, Setareh Pirmorad *(Všeobecná Fakultní Nemocnice, Prague)*; Zuzana Hotová, Aleš Mladěnka *(Nemocnice Třinec p.o., Třinec)*. |
| **Denmark**: Nulvin Bozo, Peter Christensen, Julie Lykke Harbjerg, Louise Hviid, Helle Ø Kristensen, Mira Mekhael, Mette Fugleberg Nielsen, Laerke Paulsen, Saija Sinimäki, Louise Zinck Mogensen *(Aarhus University Hospital, Aarhus)*; Peter Bonde, Anders Lyng Ebbehøj, Anne-Sophie Fenger, Aleksander Fjeld Haugstvedt, Christine Hangaard Hansen, Maria Lovisa Jönsson, Lars N Jorgensen, Peter-Martin Krarup, Anne-Louise Lihn, Christian Meyhoff, Helena Otte, Anas Ould Si Amar, Henrik Palm, Nis Schlesinger, Henry Smith, Ida Tryggedsson *(Bispebjerg Hospital, Copenhagen)*; Anne Reiss Axelsen, Jens Kristian Bælum, Mark Bremholm Ellebaek, Signe Bremholm Ellebæk, Anders Hogh *(Odense and Svendborg University hospital, Odense and Svendborg)*. |
| **Dominican Republic**: Maria Acosta, Yancy Acosta, Pedro Baez, Sylvia Batista, Luis Alfredo Betances, Luis Rodolfo Bonilla, Daniel Cabreja, Wilton Cabrera Cruz, María Mabel Collado Expósito, Aldo Crespo, Octavio Cruz-Pineda, Claudio Samuel D’óleo García, Pedro Díaz, Remberto Escoto, Jatnna Figueroa, Luis Garcia, U Garcia-Dubus Rodriguez, Humberto Gomez, Adrian Grullon, Fatherin Guerrero, Lillian Guzman, Thelma Rocío Jiménez Mosquea, Engels Lazala, Ariela Lopez, Leeany Maletta Francisco, Jhomayri Mercado, Herisardy Munoz, Jeffrey Paulino, Maricely Ambar Perez Fernandez, Rudeily Reyes, Lilia Rosa Reyes Guilamo, Ruben Rivas, Claudia Alejandra Rivas Torres, Ada Rodríguez, Julia Rodriguez-Abreu, Madelin Tamar Rosario Villa, Eleazar Santana, Joel Soto, Nassin Tactuk, Cristina Kristel Tonos Sardiñas, Raul Ubinas, Jose Victoria, Aaron Villegas *(CEDIMAT - Centro de Diagnóstico, Medicina Avanzada, Laboratorio y Telemedicina, Santo Domingo)*; Ricardo Acra-Tolari, Larissa Beltran, Luis Fernand Betances, Alejandro Blaubach, Denisse Idalia Campos Mejía, Nathalia Capellan, María Fernanda Cedeño Bruzual, Saray Cordero Spencer, Gabriella Cuevas Lantigua, Luis De jesus, Gabriela Díaz, Pedro Pablo Díaz Vásquez, Karla Disla, Jesús Antonio Echavarría Uceta, Paola Irina Eusebio Jimenez, Damaris Fernandez, Marlin Fernandez Camilo, Jiomar Manuel Figueroa Germosen, Maruel Fortunato, Leyla Gabriel Fernández, Alicia German Dihmes, Héctor Herrera, Elizabeth Leon Cuevas, Jose Alejandro Mata, Mirian Mateo De La Cruz, Dolores Mejia De la Cruz, Marcos Mirambeaux, Fabio Ortiz De La Cruz, Henry Paulino, Gabriela Pelletier, Merycarla Pichardo, Rodriguez Pumarol Próspero Enrique, Julio Rivas, Ann Stephany Sanchez Marmolejos, Roman Santana Santana, Irina Suero Almanzar, María Magdalena Vásquez Sánchez *(Hospital General Plaza de la Salud, Santo Domingo)*. |
| **Ecuador**: Nicolás Campuzano, Eddy Lincango-Naranjo, José Ricardo Negrete Ocampo *(AXXIS Hospital Quito Ecuador, Quito)*; Maria Armas, Alvaro Santiago LeMarie Guerra, Gustavo León Vizcaya *(Hospital Padre Carollo, Quito)*. |
| **Egypt**: Omar Ahmed Abdelwahab, Ahmed O. Elmehrath, Mohammed Ezzat Mostafa *(El Fayrouz Specialized Hospital, Abu Kabir, Al Sharqia)*; Gamal Amira, Ibrahim Sallam, Mohamed Sherief *(MISR Cancer Center, Al Jizah)*; Ahmed Abdelmajeed, Mostafa Abdou, Ahmed Abo Shanab, Aya Abodeeb, Roger Aboelkhel, Nour Eldin Abosamak, Amna Abou Bakr, Samar Aboubakr, Galal Abouelnagah, Yossof Abouelnagah, Omar AbouMadawy, Dina Adel, Nermeen Afifi, Sara Ahmed, Abedelrahman Ahmed, Mohamed AL Sayed, Abdelrahman Alberkamy, Mohamed Aldahma, Ahmed Ali, Mostafa Ali, Mohamed Amin Bakr, Alaa Anter, Doaa Asal, Fouad Ashoush, Olfat Ashraf, Abdel Rahman Ashraf, Rewan Atta, Salma Badr, Mohamed Bahaaeldin, Mostafa Bastawesy, Sara Darwish, Rasha El kharashy, Ziad Elassar, Seifeldin Elbadawy, Dina Elkhity, Alromisaa Elsaka, Manal Elsayed, Mostafa Elsayed Elsayed Hewalla, Mohamed Elshafey, Youssof Eshac, Fares Eshac, Amr Essameldin, Moataz Ewedah, Yara Ezz, Mohamed Farag, Inas Gadelkarim, Ziyad Gadelrab, Dina Gamal, Mira Ghaly, Nathalie Girgis, Omar Gouda, Nancy H. El Goweini, Nour Hafez, Youssef Hafez, Abdelkader Hamed Abdin, Rodina Hanno, Aliaa Hassanin, Mohamed Hassanin, Hanan M. Hemead, Abdelrahman Hemida, Maher Hosain, Ahmed Hossam, Hamza Hussein Aly Salama Aly, Mohamed Ibrahim, Abdelrahman Ibrahim, Nourhan Ibrahim, Noha Mohamed Salah Ibrahim Moussa Hamouda, Monica Iskander, Islam Khalifa, Mostafa Kotb, Nada Mahmoud, Alaa Mahmoud Abo shabana, Nora Mamdouh, Maram Metwalli, Khaled Metwally, Mahmoud Moghazy, Moustafa Mohamed, Mohamed Mourad, Esraa Moustafa, Kamilia Mubarek, Nour Eldin Nader, Ahmed Nasser, Samaa Omar, Mostafa Shehata Qatora, Mohamed Ragab, Marawan Ragal, Dina Ramadan, Rana Ramadan, Sara Ramadan, John Romany, Ahmed Sabry, Yasmeen Said, Sara Salamah, Ahmed Mostafa Saleh, Mona Saleh Mesbah Mohamed Elkaffas, Alaa Salem, Hashem Salim, Ahmed Samih, Ahmed Samir Abdelaal, Ahmed Shaheen, Yassmeen Sharafeldin Mohammed, Sameh Shehata, Abdelrahman Shehata, Mohamed Shemeis, Karim Shenit, Mennatallah Sheta, Asmaa Soffar, Yousef Tanas, Ahmed Tarek, Nermin Yehia *(Alexandria Main University Hospital, Alexandria)*; Omar Khaled Mohamed Eid, Ammar Yasser Abdulfattah, Ahmed ElSaghir, Mohamed Fouad Elganainy, Ahmed Hossam Eldin Fouad Rida, Omar Ibrahim Elsayed *(El Hadara University Hospital Alexandria University, Alexandria)*; Omar Elmandouh, Omar Hamam, Mostafa Ahmed Shehata *(Wingat Royal Hospital, Alexandria)*; Areej A. Abdelaziz, Ahmed M. Abbas, Wael Abd El-Ghani, Hossam Aldein S. Abd Elazeem, Mustafa Abd Elsayed, Ahmed Yassien Abd-Elkariem, Shimaa Abdalla, Mahmoud Abdel-Aleem, Khaled Abdelazeem, Mahmoud Abdelfattah, Mohammed Abdelhafez, Mohamed M. Abdelkarem, Ali Abdelraouf, Lamess Abdullaha, Hossam Abubeih, Moaiad Eldin Ahmed, Nagm Eldin Abu Elnga Ahmed, Ahmed Ahmed, Sarah Ahmed Saad, Sherif Alaa, Ali Alhussaini, Abdelrahman Ahmed Abdelrahman Ali, Ibrahim Ali, Wagdi Ali, Mohamed Ali Mohamed, Mohamed Ashraf, Muhammad Bassiouni, Mohamed G. El-adawy, Mohammad El-Sharkawi, Hussein Elkhayat, Khaled Elmaghraby, Shady Elsdfy, Mohamed Elsharkawy, Almoutaz Eltayeb, Mohamed Esmat Mohamed, Esraa Essam, Mahmoud Fahd, Osama Farouk, Rabea Gadelkareem, Ahmed Ghoneim, Mohammed Hamada Takrouney, Ahmed Hamdan, Abd El-Rahman Hamed, Ahmad Hasan, Ramy A. Hassan, Mohamed Abdelghafor Hassanin, Islam Hawal, Kerollos Henes, Mohamed Omar Herdan, Helal F. Hetta, Omar Ibrahem, Mostafa Ibrahim, Islam H. Ibrahim, Mahmoud Kamel, Mohamed Khallaf, Shrouk M.elghazaly, Abobakr Mahfouz, Osman Mahmoud, Wesam Mahran, Doha Mahrous, Abadeer Marsis, Ayman Mohamed, Aliae Mohamed Hussein, Abdelrahman Mohamed saad, Amr Mohamed Sayed, Mahmoud M Mohammed, Ahmed Mokhtar, Fatma A. Monib, Ahmed Nageeb, Mariam Albatoul Nageh, Mohammed Nageh, Nehal gamal Omar, Abdelrahman Ragab, Abdelrahman Ramadan, Abdallah Rashad Temerik, Mahmoud M. Saad, Aya Sabry, Hadeer Safwat, Omar Salah, Ahmed Elhussiny Salah Mahmoud, Ahmed Saleh, Mahmoud Sallam, Ahmed Samir, Reem Sayad, Esraa Sayed, Ahmed.s Sedik, Mohammed Shahine, Abdelrahman Shehata, Antonios Soliman, Wael Soliman, Mohamed Gamal Taher, Randa Wanees, Ebrahim Ahmed Yousof, Ahmed Youssef, Omar Zein Elabedeen *(Assiut University Hospital, Assiut)*; Ahmed Emad Sayed Hassan *(Al-Azhar University Hospital, Assuit)*; Shrouk Abdel Fattah, Ahmed Abostate, Samar Ali, Saad Ali Saad Salama, Mohamed Allam, Ahmed ALsadek, Ebrahim Arafa, Ahmed Barakat, Mohammed El Sherpiny, Gehad Elbehairy, Mahmoud Eleisawy, Abdelrahman Elgendy, Rewan Elhawary, Sherif Eltregy, Mahmoud Hamdy, Gehad Hassan, Aya Khalifa, Ahmed Mahmoud, Ahmed Mohamed Altukhy, Kareem Noah, Yasser Noureldin, Mostafa Nowar, Mohamed Reda Loaloa, Moustafa Saad, Ahmed Saad Elsaeidy, Khaled Saad Elsaeidy, Hesham Sabry, Mostafa Sameh, Ahmed Tarek Said-elnaby, Mohamed Zahed, Mohamed Zahran, Abdelrahman Zaid, Ahmed Zaki Zoghary *(Benha University Hospital, Benha)*; Mohamed Atef, Ahmed Mohamed, Mohamed Youssef *(Beni Suef University Hospital, Beni Suef)*; Mahmoud Hossameldin Saad Abdelhamid, Emad Alazab, Ahmed K. Awad, Amr Darwesh, Lobna El Fiky, Ibrahim ElGarhy, Badr Mostafa, Mohamed Adel Nassef, Mohamed Qassem, Mahmoud Shaban, Mahmoud Mohamed Mohamed Shalaby, Abdelrahman Wahba, Omar Youssif Omar Fouad *(Ain Shams University Specialized Hospital, Cairo)*; Menan Elsadek, Mohamed Fahmy, Dalia Gad *(Al Zahraa University Hospital, Cairo)*; Ahmed Abdelsamed, Hesham Abozied, AbdulHakeem Bayomy, Mohamed Elsalhy, Ahmed Fahim, Ahmed Seleim *(Al-Azhar University Hospitals, Cairo)*; Eman Afifi, Fatma Alzahraa Gamal, Mennatullah Gamal *(El Demerdash University Hospital, Cairo)*; Omnia Eldesouky, Mohamed hatem elmetwalli eldwini Eldwini, Mostafa Medhat Fahmy Fahmy, Ahmed Hussein, Abdallah Ouf, Omar Sami, Salem Shaat, Abdullah Wael Mostafa Khalil Bahi *(El Nozha Cairo Governorate, Heliopolis Hospital, Cairo)*; Abdurhman Atea, Ibrahim Gamal *(EL-Hussein University Hospital, Al-Azhar University, Faculty Of Medicine, Cairo)*; Mohamed El Kassas, Wael Omar, Ahmed Tawheed *(Endemic Medicine Department, Helwan University, Cairo)*; Saeid Al-oribi, Nuran Khaled Aly, Mahmoud ElFiky, Alaa Eldine Elmaghraby, Abdulrahman Elrahmany, Motaz Elsherbeeny, Youssef Helmy, Ahmed Nabil, Ahmed Samir Farahat, Mostafa Soliman, Amr Wassef *(Kasr Alainy Hospital, Cairo)*; Abdelrahman Abdelrahman, Seif ElSaban, Galal Ghaly, Rana Hamdy, Hamada Mondy *(National Cancer Institute, Cairo)*; Lubna Abdallah, Abdelrhman KZ Darwish, Mohamed Rabea *(The National Hepatology and Tropical Research Institute, Cairo)*; Mohammed A Azab, Ahmed Y Azzam *(Damietta Specialized Hospital, Damietta)*; Alzhraa Salah Abbas, Sherief Ghozy *(sheikh zayed specialized hospital, giza)*; Marwa El-Deeb, Mohamed Fawzy, Galal Ghaly, Maher Ibraheem *(Baheya Foundation for Treatment of Breast Cancer, Giza)*; Abdelrahman Bakry, Sarah Elnems, Randa Elsheikh, Mohamed Ibrahim Gbreel, Mahmoud Hafez, Mohamed Jammal, Khaled M Hamam, Abdelrahman M Makram, Omar Mohamed Makram, Salma Rabie, Sherine Yousery Askalany *(October 6 University Hospital, Giza)*; Mokhtar Mohamed Ibrahim Abushanab, Abdulrahman Eid, Mohammed Refaat Ibrahiem Amin El Ghalid *(Research institute of ophthalmology, Giza)*; Ahmed Adel abdelaty, Elsayed A. Fayad, Asmaa Radwan, Asser Sallam, Moataz Sallam, Ahmad Shokry *(Suez Canal University Hospital, Ismailia)*; Ahmed Mohamed Farouk, Ahmed Shehta *(Gastrointestinal surgery center, Mansoura)*; Ahmed Elfallal, Hossam Elfeki, Mohamed Elsaeed, Mahmoud Elsaid, Sameh Emile, Amany Makroum, Dina Mohamed elsaid, Mohamed Mostafa, Mohamed W Omar, Mohamed Rezk, Ahmed Sakr, Aly Sanad, Mostafa Shalaby, Mohammed Shawqy, Mohamed Shetiwy, Dr. Ashraf Shoma, Noura Tawakl, Asmaa Yunes *(Mansoura University Hospital, Mansoura)*; Mohamed Abdelkhalek, Amr Abouzid, Khalid Atallah, Rami Elmorsi, Ahmed Elsherbini, Khaled Gaballa, Omar Hamdy, Mohamed Hamdy, Islam Hany Metwally, Basel Refky, Mosab Shetiwy, Mohammed Zuhdy *(Oncology Center Mansoura University, Mansoura)*; Mohammed A Zahran, Fatima Abdellah, Ahmed Abdelmawla, Ahmed Abdrabou, Asmaa Abubakr, Rawda Al Gohary, Mohammed Hamdy Al-Shazly, Mohamed Ali, Abdallah R Allam, Aya Aposaeeda, Mohammed Asfour, Ammar Ayman, Abdelrahman Azzam Omran, ibrahim Tawfiq Daghash, Mahmoud Ahmed Ebada, Taher Eid, Ahmed El Kelany, Esraa El Shemy, Mohammed El-Hag-Aly, Omar ELgamal, Mahmoud Elghoury, Ahmed Farag ElKased, Takwa Hamed Ellakwa, Hamed Ellakwa, Amr Elmeanawy, Salma Elnoamany, Alaa Elsabagh, Abdelrahman Elsawey, Enas Elshabrawy, Seliman ELShakhs, Naira Elsoudy, Esraa Ezzat, Ahmed Fawzy, Abrar Gamal, Ahmed Gameel, Khaled Gharbia, Mohamed Ghonaim, Alaa Ghonaim, Ahmed Hafez, Abdelrahman Hafez, Zainab Ismail, Mohamed Khaled, Mohammed Meselhy, Pola Mikhail, Mervat Mohamed, Mohamed Mougahed, Abdulla Mustafa, Ahmed Nada, Janna Omran, Mohamed S. Ebiad, Ibrahiem Saleh, Nourhan Salem, Osama Salem, Rehab Samaka, Ahmed Samir, Salma Selim, Ghada Shalaby, Hoda Sherif, Hatem Soltan, Mahmoud Wahbah, Eman Zakaria Abdelbary, Ahmad Helmy Zayan *(Menofia University Hospital, Menofia)*; Abdelrahman Afify, Hossam Ali Hadiya, Dr Mohamed Jamal Elshref, Mostafa Mohamed Ahmed, Nourhan Nasser *(Minya University Hospital, Minya)*; Hesham Abdeldayem, Ibrahim Abdelkader Salama, Khaled Ammar, Islam Ayoub, Amr Aziz, mohammad Taha Badawy, Mohamed Balabel, Yahya Fayed, Emad Hamdy Gad, Maher Gomaha, Essam Hammad, Osama Hegazy, Elsayed Hegazy, Tarek Ibrahim, Mostafa Kallaf, Mahmoud Macshut, Ammar Magdy, Ahmed Oteem, Ahmed Sallam, Samy Samaan, Mohamed Sharshar, Ahmed Elshawadfy Sherif, Hany Shoreem, Elsayed Soliman, Hossam Eldeen Soliman, Taha Yassein, Hazem Zakaria *(National Liver Institute, Menoufia University, Shibin Elkom)*; Abdullah Eldaly, Sarah Mashaly *(El Menshawy General Hospital, Tanta)*; Sherief Abd-Elsalam, Wafaa Abdel-Elsalam, Ahmed Abdullah Shaalah, Mohamed Elbahnasawy, Ismael Elhalaby, Mohamed Hamada, Sarah Hamdy Soliman, Ahmed Hawila, Mohamed Sherif Morsy, Mohmed Naieem, Mohammed Nasreddin, Samar Salman, Sameh Sarsik, Ahmed Shabana, Engy Tolba, Mohamed Zagho *(Tanta University Hospital, Tanta)*. |
| **El Salvador**: Danilo Alfonso Arévalo Sandoval *(Clínica de Gineco-Oncología Dr. Danilo Arévalo, San Salvador)*. |
| **Estonia**: Liisa Kams, Tõnu Rätsep, Karolin Riips *(Tartu University Hospital, Tartu)*. |
| **Ethiopia**: Oumer Abdurehman, Azarias Admasu, Ataklitie Berhea, Yegeremu Eado, Nebiyou Hailu, Meklit Kidane, Abdurezak Mohammed, Khalid Mohammed, Abraham Negussie, Admasu Tibelt, Mersha Abebe Woldemariam, Lydya Yonael *(ALERT center, Addis Ababa)*; Megersa Alemu, Tigist Fisseha *(Eka kotebe General Hospital, Addis Ababa)*; Abenezer Tirsit Aklilu, Frehun Asele, Solomon Assefa, Dawit Azmach, Philimon Bekele, Dr.Mickyas Mamo, Nahom Maru, Haile Mekuria, Abel Menkir, Filagot Mikru, Kibruyisfaw Shumbash, Eyerusalem Siraw, Hilkiah Suga, Natnael Sumoro, Aklilu Teka, Efeson Thomas, Dereje Woldemariam, Estifanos Wubishet *(Myungsung Christian Medical Centre, Addis Ababa)*; Metasebia Abebe, Engida Abebe, Kirubel Abebe, Fitsum Asfaw, Eyerusalem Bergene, Mahder Eshete, Fitsum Gebreegziabher Gebrehiwot, Yetsedaw Gedefaw, Mulualem Wondafrash Mengesha, Netsanet Mengiste, Abeje Menjeta, Mahteme Bekele Muleta, Sena Sefera, Yonatan Tedla, Daniel Teklu, Henok Teshome, Leake Tirfe, Sahle Tsegabrhan, Milkias Tsehaye, Bereket Worku *(Saint Paul Hospital Millennium Medical College, Addis Ababa)*; Million Molla, Melka Supha, Tewodros Taye *(Saint Peter specialised hospital, Addis Ababa)*; Nebyou Abebe, Berhanu Alemu, Abera Chanie, Hailegebriel Degefu, Dawit Desalegn, Hiwot Gebre, Samuel Hailu, Husnia Hussen, Dawit Kassa, Tsegazeab Laeke, Tihitena Negussie Mammo, Samuel Negash, Abat Sahlu, Abraham Genetu Tiruneh, Amanuel Wolde, Hanna getachew Woldeselassie, Mnewer Y. Ahmed, Betelhem Zewdneh *(Tikur Anbessa Specialized (Black Lion) Hospital, Addis Ababa)*; Jibril Fentaw, Shemsedin Salia, Ketema Tabore, Seid Mohammed Yasin *(Yekatit 12 hospital medical college, Addis Ababa)*; Yemisirach Bizuneh Akililu, Addisalem Gurara *(Zewditu Memorial Hospital, Addis Ababa)*; Tassew Abreha, Teklebirhan Abrha, Gaym Beyene, Niguse Hailu, Mulu Atsbaha Weldu *(Adigrat General Hospital, Adigrat)*; Desalegn Abdissa, Abebe Megersa *(Ambo University Referral Hospital, Ambo)*; Teshome Tefera *(Arbaminch General Hospital, Arbaminch)*; Mequannet Tesfaw, Melatework Wolle *(Adisalem Primary Hospital, Bahir Dar)*; Dawit Asmamaw, Biniam Zemedu Assefa, Bahru Atnafu, Dereje Bedane, Ephrem Bekele, Aderaw Getie, Solomon Melkamu, Workineh Mengesha, Getachew Shumye, Gashaye Tagele, Abrham Amare Tesfa, Wubshet Workneh *(Bahir Dar University Tibebe Ghion Specialized Hospital, Bahir Dar)*; Sileshi Genetu, Eneyew Mebratu *(Gamby General Hospital, Bahir Dar)*; Lidya Chanyalew, Tilahun Deresse, Adissu Girma, Marta Seid, Dagim Shimelash, Abrham Shitaw, Bereket Tsegaye *(Debre Berhan Comprehensive Specialized Hospital, Debre Birhan)*; Tewabe Ayalew, Yoseph Solomon Bezabih, Tesfaye Diress, Berhanu Kassahun, Bersabeh Kassaye, Esubalew Mulugeta, Getasew Tesfaw, Beminet Yimenu *(Debre Markos Comprehensive specialized Hospital, Debre Markos)*; Semir Benecha, Silamlak Sisay *(Dessie Referral Hospital, Dessie)*; Mudesir Aman, Kebebe Bekele, Adem Ibrahim, Alem Mekete, Abdi Tesemma *(Maddawalabu University Goba Referral Hospital, Goba)*; Moa Jira, Adnan Abdulkadir Mohammed *(Hiwot Fana specialized University Hospital, Harar)*; Yasir Younis Abdullahi *(Jugal General Hospital, Harar)*; Ephrem Adem, Mahlet Ahmedin, Yared Assefa, Fitsum Ayde, Alazar Berhe, Zersenay Gebremeskel, Mengistu G Mengesha, Eneyew Getachew Siyoum, Sintayehu Teresa, Zinaye Wude, Fasika Yemer, Dagnachew Yohannes, Ewunetu Zeleke, Gulilat Zerihun *(Hawassa University Comprehensive Specialized Hospital, Hawassa)*; Derje Worku Degefe, Habtamu Derilo, Hankore Tamirat Derilo, Nebiyu Eliyas *(Wachemo University Nigist Elleni Mohammed Memorial Referral Hospital, Hossana)*; Firew Bayissa, Eyueal Degefa, Yadani Deressa, Lidya Gemechu, Tegenu Gurmu, Ashenafi Kasaye, Lemi Melese, Lemesa Muleta, Gersam Mulugeta, Seifu Taye, Abraham Teshome, Birhanu Tesso *(Jimma University Medical Center, Jimma)*; Dr.Gebreagziabher Gebrekirstos, Mhreteab Haile, Haftamu Kassa, Mohammed Saddik, Anteneh Tadesse *(Mekelle University Ayder Comprehensive Specialised Hospital, Mekelle)*; Alemneh Mengist, Melkamu Nidaw *(Pawi General Hospital, Pawi)*. |
| **Finland**: Elise Sarjanoja *(Länsi-Pohja Central Hospital, Kemi)*; Juuso Heikkinen, Olli Helminen, Heikki Huhta, Joonas H Kauppila, Tommi Kotkavaara, Matti-Aleksi Mosorin, Joel Pitkänen, Cheng Qian, Jaakko Sinikumpu, Henri Sova, Mikko Tastula, Ville P Virta *(Oulu University Hospital, Oulu)*. |
| **France**: Jérémie Bettoni, Stéphanie Dakpé, Bernard Devauchelle, Nolwenn Lavagen, Sylvie Testelin *(CHU Amiens, Amiens)*; François Bastard, Kim Bin, Sophie Boucher, Renaud Breheret, Olivier Fouquet, Alexandre Gueutier, Nicolas Henric, Alexis Kahn, Jean-Daniel Kün-Darbois, Didier Moukoko, Anna Pineau, Guillaume Podevin, Françoise Schmitt *(CHU Angers, Angers)*; Fadi Alshawared, Carlos Daniel Beyrne, Lionel Jouffret, Laurene Lugans, Lysa Marie-Macron *(Centre Hospitalier Avignon, Avignon)*; Alexandre Doussot, Zaher Lakkis *(CHU Besançon, Besancon)*; Omar Ahmed, Louy Alnajjar, Tommaso Cipolat Mis, Souha Fliss, Audrey Giocanti-Auregan, Thomas Gregory, Patrice Guiraudet, Adrien Le Fouler, Emmanuel Martinod, Ilaria Onorati, Marine Peretti, Julien Quilichini, Dana Radu, Tresallet Trésallet, Alban Zarzavadjian Le Bian *(Avicenne Hospital, Bobigny)*; Luke Harper *(CHU Bordeaux, Bordeaux)*; Christophe Andro, Marc Danguy des Déserts, Alexis Maffert *(Military Hospital Clermont Tonnerre (Hôpital des Armées), Brest)*; Benoît André, Tracy Chapman, Maxime Halden *(Centre Hospitalier Intercommunal de Castres-Mazamet, Castres)*; Julie Fayon, Catherine Mattevi, Karem Slim *(CHU Clermont-Ferrand, Clermont-Ferrand)*; Herjean Marion, Romain Verhaeghe *(Clinique des 2 Caps, Coquelles)*; Lynda Bendjemar, Charre Lionel, Elie Mikhael, Rosa Montero Macías, Andrea Police, Vincent Villefranque, Enrico Volpin *(Hôpital Simone Veil, Eaubonne)*; Edouard Girard, Bertrand Trilling *(CHU Grenoble-Alpes, Grenoble)*; Emmanuel Boleslawski, Alexandre Chebaro, Houlzé-Laroye Constance, Vincent Drubay, Mehdi El amrani, Clarisse Eveno, Katia Lecolle, Louis Martin, Barbara Noiret, Guillaume Piessen, Stephanie Truant, Philippe Zerbib *(CHU Lille Hôpital Claude Huriez, Lille)*; Estelle Aubry, Armande Subayi Nkembi *(CHU Lille hôpital Jeanne de Flandres, Lille)*; Laurent Arnalsteen, Franck Denimal, Antoine Lamblin *(Hôpital Privé La Louvière, Lille)*; Quentin Ballouhey, Benjamin Barrat, François Caire, Niki Christou, Laurent Fourcade, Jerome Laloze, Margaux Mekann Bouv-Hez, Henri Salle, Abdelkader Taibi, Jeremy Tricard, Julie Usseglio *(CHU Limoges, Limoges)*; Sophie Chopinet, Emilie Gregoire, Diane Mege *(Timone, Marseille)*; Marion Delpont, Clement Jeandel *(CHU Montpellier (Lapeyronie), Montpellier)*; Claire Blanchard, Vincent Crenn, Stéphane de Vergie, Waast Denis, Emilie Duchalais, Henri Fragnaud, Nicolas Regenet, Jerome Rigaud, Yoann Varenne *(CHU Nantes, Nantes)*; Philippe Anract, Maxime Barat, David Biau, Pierre-Alban Bouche, Raphaël Dautry, Anthony Dohan, Louis Idier, Elena Lang, Camille Thouny, Stylianos Tzedakis *(Hôpital Cochin - APHP, Paris)*; François Audenet, Antoine Cazelles, Alexandre Chamouni, Mehdi Karoui, Gilles Manceau, Arnaud Mejean *(Hôpital européen Georges-Pompidou, Paris)*; Célia Crétolle *(Hôpital Necker Enfants Malades - APHP, Paris)*; Pauline Clermidi, Erik Hervieux, Tristan Langlais, Lorenzo Leonelli, Emeline Maisonneuve, Beaud Nicolas, Ophelie Perrot, Doriane Prost, Anne Thomin, Thouement Thouement *(Hôpital Trousseau - APHP, Paris)*; Noémie Girard *(Institut Curie, Paris)*; Yoann Athiel, Richard Berry, Guillaume Boddaert, Stéphane Bonnet, Nathalie Cathala, Christel Conso, Christine Denet, Anaïs Laforest, Yael Levy-Zauberman, Petr Macek, Annick Mombet, Didier Ollat, Adriana Scamporlino, Agathe Seguin-Givelet, Frederic Zadegan *(institut mutualiste montsouris, Paris)*; Chamakhi Ahmed Amine, Baratte Baratte, Emmanuel Chartier-Kastler, Nathalie Chereau, Pierre-Antoine Colas, Igor Duquesne, Sebastien Gaujoux, Laurent Genser, Gaëlle Godiris Petit, Claire Goumard, Ariola Hasani, Chetana Lim, Sophie Martellotto, Charlotte Melot, Fabrice Menegaux, Ugo Pinar, Marc Pocard, Morgan Roupret, Olivier Scatton, Thomas Seisen, Noullet Séverine, Célia Turco *(Pitie Salpetriere, Paris)*; Elie Chouillard, Belinda De Simone *(Centre Hospitalier Intercommunal Poissy Saint Germain en Laye, Poissy)*; Paul Beganton, Matthieu Boisson, Denis Frasca, Thomas Kerforne *(CHU Poitiers, Poitiers)*; Damien Bergeat, Lisa Corbiere, Anis Gasmi, Marwane Ghemame, Sonia Guérin, Zine-Eddine Khene, Marie Livin, Fedy Mahmoud, Betty Maillot, Aude Merdrignac, Frederic Mouriaux, Fabien Robin, Laurent Sulpice, Charles Vazeux *(CHU Rennes - Hopital Pontchaillou, Rennes)*; Alexis P Arnaud, Nicolas Bertheuil, Soline Bonneau, Clément Thierry Cazemajou, Ianis Cousin, Coralie Defert, Elisa Fustec, Melodie Juricic, Vincent Lavoue, Gwenaël Mevel, Krystel Nyangoh Timoh, Annaëlle Renault, Philippe Violas *(CHU Rennes - Hopital Sud, Rennes)*; Lilian Schwarz, Jean Jacques Tuech *(CHU Rouen, Rouen)*; Tristan Morichau-Beauchant, Francesco Nappi *(Centre Cardiologique du Nord, Saint Cenis)*; Radwan Kassir, Frederique Sauvat *(CHU Reunion, Saint Denis)*; Elie Haddad, Aurélien Scalabre, Sophie Vermersch *(CHU Saint Etienne, Saint Etienne)*; Federico Migliorelli *(Centre Hospitalier Intercommunal des Vallées de l’Ariège, Saint Jean de Verges)*; Andrea Patrizi, Romain Verhaeghe *(CMCO Côte d’Opale, Saint Martin Boulogne)*; Fabien Fredon, Alexia Roux *(Centre Hospitalier Roland Mazoin, Saint-Junien)*; Adeline Aimé, Anne-Cecile Ezanno, Brice Malgras *(Hia Begin, St Mande)*; Zineb Cherkaoui, Antonio D’urso, Emanuele Felli, Cristians Alejandro Gonzalez, Mihaela Ignat, Didier Mutter, Patrick Pessaux, Barbara Seeliger, Michel Vix *(HUS, Pole Hépato-Digestif / IHU-Strasbourg, Strasbourg)*; Hugo Gornes, Charlotte Vaysse, Kélig Vergriete *(CHU Toulouse, Toulouse)*; Pierre Berthoumieu, Ludivine Genre, Hélène Le Gall, Vincent Misrai, Trocard Pierre *(Clinique Pasteur, Toulouse)*; Olivier Abbo, Marc Chalhoub *(Hôpital des Enfants -CHU Toulouse, Toulouse)*; Martina Aida Angeles, Mathilde Del, Alejandra Martinez *(Institut Claudius Regaud - Institut Universitaire du Cancer de Toulouse, Toulouse)*; Laurent Brunaud, Thomas Fuchs-Buder, Antoine Vancon *(CHRU NANCY, Vandoeuvre-lès-Nancy)*. |
| **Gabon**: Camara Abraham Faya, Elvam Asaph, Elisee Baruwa, Gwen Hofman, Solomon Machemedze, Michael Mayombo Idiata, Roger Muhemi, Olivier Ndizeye, Elysé Nkunzimana, Jennifer O’Connor, Zachary O’Connor, Simplice Tchoba *(Bongolo Hospital, Lebamba)*; Natacha Boumas *(Centre Hospitalier universitaire mère enfant Fondation Jeanne Ebori, Libreville)*. |
| **Georgia**: Zaza Demetrashvili, Grigol Devidze, Givi Pisarevi *(N.Kipshidze Central University Clinic, Tbilisi)*. |
| **Germany**: Sabine Baumgarten, Linda Grüßer, Frank Hölzle, Pascal Kowark, Ana Kowark, Ali Modabber, Rolf Rossaint, Benedikt Schäfer, Julia Wallqvist, Philipp Winnand, Sebastian Ziemann *(University Hospital Aachen, Aachen)*; Matthias Anthuber, Tobias Broecheler, Florian Edlinger, Yvonne Goßlau, Alexander Hyhlik-Duerr, Florian Maksymiw, Ehab Shiban, Florian Sommer, Björn Sommer, Sebastian Wolf, Sebastian Zerwes *(University Hospital Augsburg, Augsburg)*; Katharina Beyer, Carsten Kamphues, Johannes Christian Lauscher, Lucas D. Lee, Florian N Loch, Christian Schineis *(Charité University Medicine - Campus Benjamin Franklin, Berlin)*; Ilgar Aghalarov, Orlin Belyaev, Chris Braumann, Annika Enste, Tim Fahlbusch, Torsten Herzog, Philipp Höhn, Julian Horn, Julia Jedanowski, Julia Knipschild, Andreas Minh Luu, Prem Vignesh Mohan, Leonie Siemen, Illya Slobodkin, Johanna Josefine Strotmann, Waldemar Uhl, Katerina Wolf *(St. Josef-Hospital, Bochum)*; Mark Coburn, Eva Egger, Klaus Eichhorn, Jana Enderes, Frederick Far, Alina Franzen, Tim R. Glowka, Erdem Güresir, Alexis Hadjiathanasiou, Jörg C. Kalff, Zaki Kohistani, Steffen Manekeller, Alexander Mustea, Chris Probst, Thomas Randau, Florian Recker, Patrick Schuss, Nadine Straßberger-Nerschbach, Sebastian Strieth, Hendrik Treede, Cornelius J. van Beekum, Hartmut Vatter, Markus Velten, Tim O. Vilz, Dieter Wirtz, Maria Wittmann *(University Hospital Bonn, Bonn)*; Michael Behr, Dirk Rolf Bulian, Benedikt Marche, Tobias Moczko, Robin Otchwemah, Sissy-Amelie Schulz, Panagiotis Thomaidis *(Cologne-Merheim Medical Center (CMMC), Witten / Herdecke University, Cologne)*; Christiane Bruns, Claus Cursiefen, Christian Domröse, Hans Fuchs, Ludwig Maximilian Heindl, Michael R. Mallmann, Christoph Mallmann, Dominik Alexander Ratiu, Alexander Christopher Rokohl *(University Hospital of Cologne, Cologne)*; Ulrich Bork, Ulrich Canzler, Marius Distler, Sandra Korn, Cornelia Meisel, Marcus Meusel, Andrea Petzold, Christian Praetorius, Janusz von Renesse, Juergen Weitz, Pauline Wimberger *(University Hospital Carl Gustav Carus, Technical University Dresden, Dresden)*; Nour Alkhanji, Georg Fluegen, Stephen Fung, Kefah Jaber, Wolfram Trudo Knoefel, Christian Vay *(University Hospital Duesseldorf, Duesseldorf)*; Octavian Clonda, Tatiana Cottin, Evelina Juodiene, Domantas Juodis, Biljana Petrovic, Oleksandra Solodarenko *(Rottal-Inn-Kliniken, Eggenfelden)*; Johannes Binder, Robert Grützmann, Danilo Hackner, Raymund E. Horch, Stefanie Junker *(Universitätsklinikum Erlangen, Erlangen)*; Mani Arsalan, Severine Banek, Felix Chun, Sara Fatima Faqar-Uz-Zaman, Daniel Keese, Luis Kluth, Udo Rolle, Andrea Schmedding, Andreas Schnitzbauer, Arnaud Van Linden, Thomas Walther *(Frankfurt University Hospital, Goethe University, Frankfurt)*; Jörg Bayer, Jürgen Beck, Helge Eberbach, Christian Fung, Luisa Mona Kraus, Christian Leiber, Nicolas Neidert, Richard Sandkamp, Daniel Schlager, Oliver Schnell, Antonia Schulte, Jakob Strähle *(University Medical Center Freiburg, Faculty of Medicine, Freiburg)*; Stefanie Jarmusch, Helmut Franz Georg Novotny, Fritz Spelsberg *(Klinikum Fürstenfeldbruck, Fürstenfeldbruck)*; Johannes Becker, Christian Fulghum, Bernhard Gonschor, Marit Herbolzheimer, Lena Keppler, Sina Nicolaiciuc, Alexander Trulson, Holger Vogelsang, Benno Zimmermann *(Klinikum Garmisch-Partenkirchen, Garmisch-Partenkirchen)*; Amir Ali Akbari, Andreas Boening, Fabian Edinger, Andreas Hecker, Matthias Hecker, Michael Knitschke, Christian Koch, Martin Reichert, Michael Sander, Götz Schmidt, Emmanuel Schneck, Eberhard Uhl *(University Hospital Giessen and Marburg, Giessen)*; Silvia Flachs Nóbrega, Philipp Kauffmann, Clemens Miller, Marcus Nemeth, Denise Sievers, Susanne Wolfer *(University Hospital Goettingen / Universitätsmedizin Goettingen, Goettingen)*; Ulrich Kisser, Jorg Kleeff, Johannes Klose, Kerstin Lorenz, Nancy Papendick, Stefan Plontke, Ulrich Ronellenfitsch, Ingmar Seiwerth, Susanne Steer, Christoph Thomssen, Jörg Ukkat *(University Hospital Halle, Halle)*; Christian W. Dumpies, Isabel Fischer, Michael Gessner, John Hanke, Friederike Klauke, Sebastian Leuschner, Thomas Mendel, Katharina Müller, Birte Schmidt, Valentin Schreiter, Peter Stosberg, Franziska Vinz *(BG Klinikum Bergmannstrost, Halle (Saale))*; Beate Herbig, Johannes Sander, Thilo Maria Schulte *(Schön Klinik Hamburg Eilbek, Hamburg)*; Christian Stephan Betz, Julian Bewarder, Johannes Bier, Arne Böttcher, Simon Burg, Chia-Jung Busch, Lara Bußmann, Martin Gosau, Annika Heuer, Jakob Izbicki, Till Orla Klatte, Daniela König, Leon-Gordian Köpke, Nikolaus Moeckelmann, Christine Nitschke, Mark Praetorius, Matthias Priemel, Rupert Stadlhofer, Martin Stangenberg, Faik G. Uzunoglu, Lukas Wittig, Henrike Zech, Nina Zeller *(University Medical Center Hamburg-Eppendorf, Hamburg)*; Christian Peiper, Frederic Roux, Tsiona Spaeth *(Evangelisches Krankenhaus Hamm, Hamm)*; Roland Fricker, Thomas Müller, Lars Schröder *(Klinikum Hanau, Hanau)*; Mohammed Alasmari, Clara Boeker, Ibrahim Hakami, Ibrahim Abdullah Hakami, Julian W Mall *(KRH Nordstadt-Siloah Hospitals, Hannover)*; Ioannis Kyritsis, Stefan Welter *(Lung Clinic Hemer, Hemer)*; Christian Graeb, Kristin Huber-Strößner, Karine Nikolaieva *(Sana Klinikum, Hof)*; Daniela Branzan, Markus Doss, Ines Gockel, Carolin Jödicke, Georg Osterhoff, Christina Pempe, Andreas Roth, Robert Sucher *(University Hospital Leipzig, Leipzig)*; Tina Adler, Kathrin Kelly, Judith Lindert, Janica Merkle, Julia Siebert *(University Hosital Schleswig- Holstein, Lübeck)*; Christoph Hirche, Ulrich Kneser, Christian Tapking *(BG Trauma Center Ludwigshafen, Ludswigshafen am Rhein)*; Rachit Agrawal, Konstantinos Gousias, Homeira Qureischie *(St Marien Hospital Lünen, Lünen)*; Roland Croner, Lisa Koslowski, Hardy Krause, Frank Meyer, Anke Rissmann, Salmai Turial *(University Hospital Magdeburg, Magdeburg)*; Bilal Al-Nawas, Marco Johannes Battista, Jan Goedeke, Annette Hasenburg, Julia Heider, Valerie Catherine Linz, Lena Katharina Mueller, Simon Zeller *(University Hospital Mainz, Mainz)*; Sina- Louisa Patrizia Jentschura, Karl-Friedrich Kowalewski, Maximilian Kriegmair, David Männle, Nuh Rahbari, Marie-Claire Rassweiler-Seyfried, Christoph Reissfelder, Nicole Rotter, Claudia Scherl, Steffen Seyfried *(Mannheim University Medical Center (Universitätsmedizin Mannheim), Mannheim)*; Andreas Kirschniak, Jens Rolinger, Peter Wilhelm *(Kliniken Maria Hilf, Moenchengladbach)*; Franz G. Bader, Anna Eleonora Gut, Stephanie Ottl *(Isarklinikum, München)*; Alexandra Viktoria Behr, Alexandros Diamantis, Andreas Fichter, Jens Gempt, Florian Grill, Matthias Heck, Daniel Jira, Michael Kallmayer, Florestan Koll, Stefan Luhne, Bernhard Meyer, Robert Patachia, Ilaria Pergolini, Daniel Reim, Seyer Safi, Christoph Schäffer, Moritz Schirren, Arthur Wagner, Helmut Wegmann, Markus Wirth, Zhaojun Zhu *(Klinikum Rechts der Isar TUM School of Medicine, Munich)*; Ughur Aghamaliyev, Markus Albertsmeier, Mahmoud Almaghrabi, Wolfgang Böcker, Jan Bruder, Konstantin Frank, Viktoria Herterich, Verena Huber, Matthias Ilmer, Christian Kammerlander, Alexander M. Keppler, Carl Neuerburg, Viktor H. von Ehrlich-Treuenstätt, Jens Werner *(Ludwig Maximilian University of Munich - Großhadern, Munich)*; Nikolaus Börner, Florian Fegg, Daniela Hartmann, Roland Ladurner, Paris Liokatis, Kathrin Patzer, Justin Gabriel Schlager, Wenko Smolka, Petra Zimmermann *(Ludwig Maximilian University of Munich - Innenstadt, Munich)*; Rudolf Hatz, Diana Steinhart, Mircea Gabriel Stoleriu *(Asklepios Pulmonary Hospital, Munich Gauting)*; Claudio Glowalla, Tim Saier, Dorien Schneidmueller *(Berufsgenossenschaftliche Unfallklinik Murnau, Murnau)*; Karl Wilhelm Henkel, Josef Stadler, Martin Steiner *(RoMed Klinik Prien am Chiemsee, Prien am Chiemsee)*; Katharina Hölz, Julia Christina Kaiser, Christian Knorr *(Klinikum St. Hedwig, Barmherzige Brüder, Regensburg)*; Stefan M. Brunner, Britta Kuehlmann, Kyriakos Oikonomou, Karin Pfister, Lukas Prantl *(University Hospital Regensburg, Regensburg)*; Roland Flurschütz, Jonas Herzberg, Human Honarpisheh, Marie Kröger, Dominic Lepiorz, Charlotte Luths, Andreas Niemeier, Yara Sras, Tim Strate, Thore Winter *(Krankenhaus Reinbek St. Adolf-Stift, Reinbek)*; Kai Nowak, Tobias Reinhard *(RoMed Klinikum Rosenheim, Rosenheim)*; Thomas Freiman, Florian Gessler, Sae-Yeon Won *(University Hospital Rostock, Rostock)*; Gregor A. Stavrou, Rizky Widyaningsih *(Klinikum Saarbruecken, Saarbruecken)*; Sebastian Hoffmann, Ruth Christine Schäfer, Johannes Tobias Thiel *(BG Klinik, Tübingen)*; Elisa Bertolani, Alfred Königsrainer, Christian Konrads, Markus W. Löffler, Markus Quante, Christoph Steidle, Lisa Überrück, Can Yurttas, Patrick Ziegler, Alexander Zimmermann *(University Hospital Tuebingen, Tuebingen)*; Christian Bolenz, Davut Dayan, Jens Greve, Thomas K. Hoffmann, Wolfgang Janni, Simon Laban, Niklas Löbig, Fabienne Schochter, Julius Malte Vahl, Felix Wezel *(University Hospital Ulm, Ulm)*; Veronika Greif, Silke Pusch von, Stefan Schmidbauer *(Kreisklinik Wolfratshausen, Wolfratshausen)*; Markus Hirschburger, Imke Marsch, Rolf Schneider *(Klinikum Worms, Worms)*; Lars Boenicke, Stephan Degener, Johannes Doerner, Nici Markus Dreger, Franz Christian Horstmeier, Jakob Kruschwitz, Adrian Rombach, Nele Schmidt, Rose Seiberth, Jaswinder Singh, Marieke Smit, Friedrich-Carl von Rundstedt, Hubert Zirngibl *(Helios Universitätsklinikum Wuppertal (Universität Witten/Herdecke), Wuppertal)*; Joachim Diessner, Sabine Friedrich, Christoph-Thomas Germer, Philipp Helmer, Johannes Herrmann, Peter Kranke, Hubert Kübler, Johan Lock, Christopher Lotz, Rainer Meffert, Patrick Meybohm, Sophie Müller, Quirin Notz, Maria Popp, Nicolas Schlegel, Tobias Schlesinger, Benedikt Schmid, Magdalena Sitter, Andreas Steinisch, Agnes Treutlein, Anne van den Berg, Armin Wiegering, Thomas Erik Wurmb *(University Hospital Würzburg, Würzburg)*. |
| **Ghana**: Nana Kwaku Agyeman-Duah, Enoch Appiah, Ralph Armah, Christopher Asare, Lawrence Awere-Kyere, Dennis Daary, Delali Gakpetor, Stephen Minlah Allah, Ambe Obbeng, Dorcas Osei-Poku, Diana Puozaa, Enoch Tackie *(Greater Accra Regional Hospital, Accra)*; Nii Armah Adu-Aryee, Nelson Agboadoh, Offei Asare, Antoinette Bediako Bowan, George Darko Brown, Joe-Nat Clegg-Lamptey, Florence Dedey, Cedric Dery, Benjamin Sena Fenu, Marian Abedua Harrison, Philemon Kumassah, Ekins Kuuzie, Josephine Nsaful, David Olatayo Olayiwola, Cecilia Smith *(Korle-Bu Teaching Hospital, Accra)*; Charles Banka, Romeo Hussey *(Berekum Holy Family Hospital, Berekum)*; Diallo Abdoul Azize, Luke Adagrah Aniakwo, Yvonne Adofo-Asamoah, Evans Kofi Agbeno, Meshach Manu Agyapong, Thomas Agyen, Kwasi Agyen Mensah, Baba Alhaji Bin Alhassan, Mabel Amoako-Boateng, Peter Appiah-Thompson, Nita Asamoa-Manu Gyimah, Moses Asante- Bremang, Alvin Asante-Asamani, Henry Atawurah, Anthony Baffour Appiah, Richard Ogirma Baidoo, Ebikela Ivie Baidoo, Benedict Boakye, Abigail Boateng, Dora Dadoe, Makafui Seth Caleb-Joshua Kwasi Dayie, Samuel Debrah, Dr Kingsley Doku, Enti Enti, Sebastian Ken-Amoah, Patience Koggoh, Richard Kpangkpari, Patrick Maison, Samuel Mensah, Philip Mensah, Teresa Aba Mensah, Martin Tangnaa Morna, Jilac Nimako-Mensah, John Nkrumah, Michael Nortey, Emmaunel Owusu Ofori, Isabella Naa Morkor Opandoh, Ethel Osei-Tutu, Jefferson Owusu Adae, Kofi Quansah, Elizabeth Quartson, Ganiyu Adebisi Rahman, David Walawah, Makafui Yigah, Safia Yussif *(Cape Coast Teaching Hospital, Cape-Coast)*; Nuna Jiagge, Emmanuel Nachelleh *(Ho Teaching Hospital, Ho)*; Jane Acquaye, Kwabena Agbedinu, Fareeda Agyei, Akosua Agyemang-Prempeh, Kwabena Amo-Antwi, Michael Amoah, George Amoah, Yaw A Amoako, Daniel Gyawu Aning, Frank Ankobea-Kokroe, Dominic Annor Mintah, David Anyitey-Kokor, Adu Appiah-kubi, Joshua Arthur, Vincent Ativor, Isaac Barnor, Yasmine Braimah, Regina Darko- Asante, Anthony Davor, Mohammed Duah Issahalq, Mawutor Dzogbefia, Tano Emile, Papa Fiifi - Yankson, Valerie Gaveh, Senyo Gudugbe, Solomon Gyabaah, Frank Enoch Gyamfi, Adam Gyedu, Derrick Gyimah, Bernard Hammond, Boakye - Yiadom Jonathan, Yorke Joseph, Thomas Okpoti Konney, Anna Konney, Kwasi Kusi, Ishmael Kyei, Agbenya Lovi, Nuhu Naabo, Boateng Nimako, Beauty Nyadu, Obed Ofori Nyarko, Ben Blay Ofosu-Barko, Philip Peprah Oppong, Anita Osabutey, Martha Poku, Robert Sagoe, Abiboye Yifieyeh *(Komfo-Anokye Teaching Hospital, Kumasi)*; George Ansong *(Tamale Central hospital, Tamale)*; Adam Abass, Alhassan Abdul-Mumin, Theophilus Adjeso, John Abanga Alatiiga, Munira Amadu, Nathaniel Annan, German Azahares Leal, Mohammed Bukari, Alexis Buunaaim, Ernest Cheyuo, Latif Daboo Salifu, Michael Damah, Malcolm Dery, Edem Kojo Dzantor, Odoniel Guerra Garcia, Yabasin Iddrisu Baba, Adamu Issaka, Abdul-Jalilu Mohammed Muntaka, James Murphy, Yaa Nyarko Agyeman, Wisdom Opoku Amankwaa, Imoro Osman, Samuel Pie, Anwar Sadat Seidu, Mohammed Sheriff, Ana Maria Simono Charadan, Stephen Tabiri, Abraham Titigah, Mundashiru Yahaya, Musah Yakubu, Edwin Mwintiereh Ta-ang Yenli *(Tamale Teaching Hospital, Tamale)*. |
| **Greece**: Ioannis Grypiotis, Nikolaos Kiriakopoulos, Georgios Koliopoulos, Vasilis Kyvelos, Georgia Micha, Triada Papadopoulou *(‘Elena Venizelou’ General and Maternity hospital of Athens, Athens)*; Dimitrios Balalis, Evangelos Fradelos, Dimitrios Korkolis *(Agios Savvas Anticancer Hospital, Athens)*; Nicholas Alexakis, Kyveli Angelou, Dimitrios Haidopoulos, Anastasia Prodromidou, Alexandros Rodolakis, Nikolaos Thomakos *(Alexandra General Hospital, Athens)*; Dimitris Psychogios, Pantelis Antonakis, Konstantinos Bramis, Leonidas Chardalias, Ioannis Contis, Nikos Dafnios, Dionysios Dellaportas, Papalouka Dimitra, Georgios Fragkoulidis, Georgios Gkiokas, Antonios Gklavas, Theodoros Hadjizacharias, Dimitra Karageorgou, Manousos Konstadoulakis, Christina Kontopoulou, Dimitrios Massaras, Nikolaos Memos, Ioannis Papaconstantinou, Dimitrios Politis, Andreas Polydorou, Konstantinos Stamatis, Theodosios Theodosopoulos, Antonios Vezakis *(Aretaieion Hospital, Athens)*; Konstantinos Avgerinos, Jevgeni Katunin, Aristotelis Kechagias, Dionysia Kelgiorgi, Neoklis Kritikos, Pasi Pengermä *(Athens Bioclinic Hospital, Athens)*; Theodosios Bisdas, Argyrios Ioannidis, Michael Konstantinidis, Sofia Konstantinidou, Nikolaos Patelis *(Athens Medical Center, Athens)*; Maria Ioanna Antonopoulou, Eirini Deskou, Vasileios Kalles, Dimitrios K. Manatakis, Nikolaos Stamos, Nikolaos Tasis *(Athens Naval and Veterans Hospital, Athens)*; Nikolaos Arkadopoulos, Nikolaos Danias, Panagiota Economopoulou, Maximos Frountzas, Panagiotis Kokoropoulos, Nikolaos Michalopoulos, Jonida Selmani, Theodoros Sidiropoulos, Panteleimon Vassiliu *(Attikon University General Hospital, Athens)*; Kosmas I. Paraskevas *(Central Clinic of Athens, Athens)*; Dimitrios Bartziotas, Konstantinos Bouchagier, Ilias Galanis, Theodosis Kalamatianos, Stylianos Kapiris, Angeliki Kolinioti, Eleni Mavrodimitraki, Panagiotis Metaxas, Alexandrina Nikova, Michail Psarologos, Maria Sotiropoulou, George Stranjalis *(Evaggelismos General Hospital, Athens)*; Konstantinos Albanopoulos, Panagiotis Kondilis, Gavriella Zoi Vrakopoulou *(Evgenideio Hospital, Athens)*; Aristeidis Chrysovergis, Georgios Chrysovitsiotis, Evangelos Giotakis, Vasiliki Kanellopoulou, Efthymios Kyrodimos, Andreas Larentzakis, Pavlos Pantos, Vasileios Papanikolaou, Spyridon Potamianos, Charalampos Theodoropoulos, Alexandra Triantafyllou, Tania Triantafyllou *(Hippocratio General Hospital, Athens)*; Kleoniki Georgousi, Peter Panagiotou, Ekaterini Christina Tampaki *(KAT Athens General Hospital, Athens)*; Emmanouil Avramidis, George Babis, Evangelos Zafeiris *(Konstantopouleio General Hospital of Athens, Athens)*; Afroditi Antoniou, Nikolaos Bessias, Dimitris Maras, Theofanis Papas, Konstantinos Roditis, Ioannis Tsagkos, Paraskevi Tsiantoula *(Korgialenio-Benakio Hellenic Red Cross Hospital, Athens)*; Andreas Alexandrou, Efstratia Baili, Alexandros Charalabopoulos, Dimitrios Dimitroulis, Panagiotis Dorovinis, Zoe Garoufalia, Prodromos Kanavidis, Ioannis Karavokyros, Lysandros Karydakis, Stylianos Kykalos, Eleandros Kyros, Nikolaos Machairas, Aikaterini Mastoraki, Nikolaos Nikiteas, Alexandros Papalampros, Dimitrios Schizas, Antonia Skotsimara, Paraskevas Stamopoulos, Athanasios Syllaios, Alexis Terras, Nefeli Tomara, Gerasimos Tsourouflis, Ilias Vagios, Constantinos Zografos *(Laiko University Hospital, Athens)*; Konstantinos Apostolou, Nikolaos Georgopoulos *(Mediterraneo Hospital, Athens)*; Theofani Antoniou, Christina Antzaka, Areti Falara, Socrates Fragoulis, Panagiotis Ftikos, Fedra Matsouka, Konstantinos Perreas, Panagiota Rellia, Evangelia Samara, Anna Smirli, Androniki Tasouli, Apostolos Thanopoulos *(Onassis Cardiac Surgery Center, Athens)*; Petros Loukas Chalkias, Georgia Dedemadi, Panagiotis Mourmouris, Andreas Skolarikos, Nikoletta Theochari, Lazaros Tzelves *(Sismanoglio - Amalia Fleming General Hospital, Athens)*; Stylianos Gaitanakis, Theodoros Milas, Emmanuel Theodorakis *(Sotiria General Hospital of Thoracic Diseases, Athens)*; Paraskevi Karona, Pagona Kastanaki, Angelos Tzouganakis *(Chania General Hospital ‘St George’, Chania)*; Christos Agalianos, Ioannis Tsouknidas, Andreas Xenakis *(Naval and Veterans Hospital of Crete, Chania)*; Emmanuel Chrysos, Konstantinos Lasithiotakis, Taxiarchis Nikolouzakis, Sofia Xenaki, Evangelos Xynos *(University Hospital of Heraklion Crete, Heraklion Crete)*; Eftychios Lostoridis, Eleni-Aikaterini Nagorni, Antonio Pujante, Paraskevi Tourountzi *(Kavala General Hospital, Kavala)*; Aggeliki Al, Kyriaki Baxevanidou, Konstantinos Bouliaris, Matheos Efthimiou, Christos Kalfountzos, Georgios Koukoulis, Vasileios Lachanas, Konstantinos Petropoulos, Ioannis Tsitiridis *(General Hospital of Larissa ‘Koutlimpaneio and Triantafylleio’, Larissa)*; Fragkiskos Angelis, Eleni Arnaoutoglou, Ioannis Baloyiannis, Metaxia Bareka, Anna Bouronikou, Gregory Christodoulidis, Alexandros Daponte, Maria Fergadi, Nick Gkolias, Eleni Gkrinia, Jiannis Hajiioannou, Nikos Kalogritsas, Eleni Karoni, Christos Korais, Giorgos Krestinidis, Antigoni Ktisti, Dimitrios Magouliotis, Charikleia Maiou, Konstantinos Malizos, Maria Minasidou, Maria Ntalouka, Anna Maria Ntziovara, Effrosyni Palla, Konstantinos Perivoliotis, Fani Saini, Athina Samara, Athanasios Saratziotis, Charalampos Skoulakis, Efthymios Solomi, Konstantinos Stamoulis, Christos Dimitrios Terzoudis, Evangelia Tsironi, George Tzovaras, Kyriaki Vallianou, Dimitris Zacharoulis, Anna Ziogkou, Κωνσταντίνος Δακής *(General University Hospital of Larissa, Larrisa)*; Liolis Elias, Ioannis Maroulis, Francesk Mulita, Kerasia-Maria Plachouri, Michail Vailas *(General University Hospital of Patras, Patras)*; Charalampos Doitsidis, Eva Filo, Ioanna Gkalonaki, Eleni Kogia, Konstantina Kontopoulou, Magdalini Mitroudi, Christina Panteli, Ioannis Patoulias, Olga Ioulia Semkoglou, Dimitrios Sfoungaris, Ioannis Valioulis, Γιωργος Κουτσουμης *(G. Gennimatas Thessaloniki General Hospital, Thessaloniki)*; Ioannis Astreidis, Panagiotis Christidis, Orestis Ioannidis, Lydia Loutzidou, Antonis Mantevas, Konstantinos Paraskevopoulos, Dimitris Tatsis *(George Papanikolaou General Hospital of Thessaloniki, Thessaloniki)*; Apostolos Athanasiadis, Themistoklis Dagklis, Ioannis Kalogiannidis, Georgios Kapetanios, Apostolos Mamopoulos, Chrysoula Margioula-Siarkou, Stamatios Petousis, Ioannis Tsakiridis *(Hippocratio Hospital, Thessaloniki)*; Christos Anthoulakis, Chrysanthos Christou, Antonios Fantakis, Eirini Iordanidou, Christos Kaselas, Sousana Panagiotidou, Vasileios Papadopoulos, Kyriakos Papavasiliou, Athanasios Piachas, Ioannis Siasios, Ioannis Spyridakis, Theodoros Theodoridis, Andreas Tooulias, Eleftherios Tsiridis, Maria Tsopozidi, Georgios Tsoulfas *(Papageorgiou General Hospital, Thessaloniki)*. |
| **Guatemala**: María Alemán, Estuardo Brolo, Alitza Gutiérrez Ruiz, Ana Lucia Lemus *(Hospital Universitario Esperanza, Guatemala)*; Jennifer Greenberg, Krisna Mishel Morales Chew, José Rodrigo Oliva, Alejandra Rodas *(Centro Clínico Cabeza y Cuello, Guatemala City)*; María Alejandra De León Lima, Ismar Lopez Muralles, Ana Lucía Portilla, Gustavo Recinos, Felipe Solares *(Hospital General De Enfermedades, Guatemala City)*; Maria-Lorena Aguilera-Arevalo, Gaby Ajcip, Claudia Anton, Jacqueline Carrera, Jose Cojulun, Mario-Andrés Flores, Noriega José, Carlos Adolfo Marroquín Paiz, Steffanía Morales, Ramiro Najera, Eduardo Quiñónez Lorenzana, Pablo Rivera, Lesly Rodas, Victor Santos, Dianne Sosa, Natalia Ybarra *(Hospital General San Juan De Dios, Guatemala City)*; Amalia Barrios Duarte, José David Pérez Cajti, Carlos Régil *(Hospital Herrera Llerandi, Guatemala City)*; walter A Osorio, Rember Rosales Arriola, Luis-Fernando Talé-Rosales *(Hospital Juan Jose Arevalo Bermejo, Guatemala City)*; Danilo Herrera, Servio Tulio Torres Rodríguez, Sergio Alejandro Villeda *(Hospital San Vicente, Guatemala City)*; Joshua Anicetti, Megan Lowey, Andrea Michelle Lowey Medina *(Sanatorio Las Majadas, Guatemala City)*; Javier Ardebol, Kathia Barillas, Sabrina Barillas, Francis De Leon, Salvador Recinos *(Symmetria, Guatemala City)*; Miguel-Angel Marroquin-Alpirez, Yessica Yax *(Hospital Nacional de San Marcos, San Marcos)*. |
| **Hong Kong SAR, China**: Sophie Hon, Yuk Ho Liu, Alex Qinyang Liu, Shirley Liu *(Alice Ho Miu Ling Nethersole Hospital, Hong Kong)*; Hei Tung Natalie Chiu, Chi Man Tom Chow, Victor Hau, Ho Wai Ip, Brian Mak, Chung Ying Mok, Dennis Ng, Yin yu Eva Siu, Kiu Fung Wong *(North District Hospital, Hong Kong)*; Jingya Jane Pu, Yu-xiong Su *(Queen Mary Hospital, Pok Fu Lam)*; Kit Ying Au-Yeung, David Yuen Chung Chan, Albert Chan, Shannon Melissa Chan, Tsz Ching Chang, Tor Wo Chiu, Wang Kei Chiu, Kaori Futaba, Zhexi He, Man Fung Ho, Kevin Ki Wai Ho, Jacky Yan Kit Ho, Janet Wui Cheung Kung, Cheuk Ho Lam, Rainbow W.H. Lau, Samuel Ka Kin Ling, Hon Ting Lok, Tony Wing Chung Mak, Chi-Fai Ng, Simon Ng, Kelvin Kwok-Chai Ng, Calvin S.H. Ng, Michael Tim Yun Ong, Teresa Tan, Sui Fan Tang, Jeremy Yuen-Chun Teoh, Anthony Teoh, Bess Siu Yan Tsui, George Kwok Chu Wong, Randolph Wong, Kwok Chuen Wong, Chi Hang Yee *(Prince of Wales Hospital, Sha Tin)*. |
| **Hungary**: Zsuzsanna Antal, Attila Kalman, Peter Voros *(Ist Department of Pediatrics, Semmelweis University, Budapest)*; Kiarash Bahrehmand, Timea Echim, Tamás Mersich, Zoltan Novak, Tamás Sztipits, Dániel Wettstein *(National Institute of Oncology, Budapest)*; Laszló Ádám Bihari, László Hidi, Laszlo Piros, Balazs Rózsa, Peter Sotonyi, Lilla Szatai *(Semmelweis University (please use for all units), Budapest)*; György Herczeg, Bálint Pordány, Fanni Tornyi *(Szent Imre Egyetemi Oktatókórház, Budapest)*; Kristof Dede, Tamás Egyed, György Saftics *(Uzsoki Hospital, Budapest)*; Judit Kulcsicka-Gut, Zsófia Sipos, Dezso Toth *(Miskolc Academic County Hospital, Miskolc)*. |
| **India**: Bhavin Patel, Dhaivat Vaishnav *(Zydus Hospital, Ahmedabad Gujarat)*; Raghunandan Gorantlu Chowdappa, Hardil Majmudar, Saptak Mankad, Sohilkhan Pathan *(Shri Krishna Hospital and pramukhswami medical college, Anand , Gujarat)*; Sharathkumarkl L, Irappa Madabhavi, Lokesh Sasatti *(Kerudi Cancer Hospital, Bagalkot)*; Dhananjaya Bhat, Subramanyam Mahankali, Santhosh kumar Sampengere Annayappa *(Aster RV Hospital, Bangalore)*; Tulika Agrawal, Premkumar Anandan, Aditya Baindur, Manjunath Bd, Savitha C, Anitha G S, Sandeep Harigond, Tejeswini K K, Venkatesh Kesarla, Sunil Kumar Venkatappa, Mallikarjuna Manangi, Hareesh P B, Dr jyothi k r Ranjan, Athish Shetty, Santhosh Shivashankar Chikkanayakanahalli, Tanvi Sunil, Shreya Syamala, Kavya Tharanath *(Victoria Hospital, Bangalore)*; Aditya Atal, Niranjana Rajagopal, Sumit Thakar *(Sri Sathya Sai Institute of Higher Medical Sciences, Bengaluru)*; Srinath B S, Kavitha Jain, Vinod Nk, Thirumanikandan P L *(Sri Shankara Cancer Hospital and Research Centre, Bengaluru)*; Aruna Kumar, Nitu Mishra, Sushruta Shrivastava, Dr Rekha Wadhwani *(Gandhi Medical College and Sultania Zanana Hospital, Bhopal)*; Reyaz Ahmad, Zainab Ahmad Haq, Prateek Behera, Ritika Dhurwe, Rehan Haq, Vaibhav Jain, Anuj Jain, Sunaina Karna, Manoj Nagar, Kameshwarachari Pushpalatha, Sumit Raj, John Ashutosh Santoshi, Pooja Singh, Virendra Verma, Dr Vaishali Waindeskar, Dr Moorat Singh Yadav *(ALL INDIA INSTITUTE OF MEDICAL SCIENCE BHOPAL, BHOPAL)*; Zaheda Aziz, Koyel Chakraborty, Preetam Chappity, Gurudip Das, Debajyoti Datta, Saubhagya Kumar Jena, Madhabananda Kar, Susanta Khuntia, Pankaj Kumar, Ravi Kumar, Aswathi Kv, Abhijeet Mishra, Tushar Mishra, Swastik Mishra, Yash Mittal, Dillip Muduly, Ritesh Panda, Sibasish Panigrahi, Sucheta Parija, Saroj Patra, Bikram Rout, Rabi Sahu, Saurav Sarkar, Arunkumar Sekar, Sweta Singh, Sanjibani Sudha, Mahesh Sultania, Sujit Tripathy, Paulson Varghese *(All India Institute Of Medical Sciences - Bhubaneswar, Bhubaneswar)*; Anand Gupta, Rajeev Kansay, Robin Kaushik, Simrandeep Singh *(Government medical college hospital, Chandigarh)*; Sunil Kumar Gupta, Lileswar Kaman, Madhivanan Karthigeyan, Siddhant Khare, Vishal Kumar, Sandeep Mohindra, Ninad Patil, Pravin Salunke, Ajay Savlania, Kavindra Singh, Manjul Tripathi *(Postgraduate Institute of Medical Education & Research, Chandigarh, India, Chandigarh)*; Pradeep Krishna RV, Gomathy Narasimhan, Ashwin Rammohan, Mohamed Rela *(Dr.Rela Institute & Medical Centre, Chennai)*; Akhila Appukuttan, Swati Goudar, Shweta Mallick, Sudheer Othiyil vayoth, Anupama Rajanbabu, Christi Titus *(Amrita Institute of Medical Sciences Hospital, Cochin)*; Ezhir Selvan Chidambarasamy, Dr Devdas Madhavan, Anandan Murugesan, Kuppurajan Narayanasamy, Barani kumar P B Pb, Dr Firoz Rajan, Anbukkani Subbian, Paari Vijayaragavan *(Kovai Medical Centre and Hospital, Coimbatore)*; Rahul Gupta, Arvind Kumar *(Synergy Institute of Medical Sciences, Dehradun)*; Biplob Borthakur, Tapan singh Chauhan, Akhil Govil, Shubhra Gupta, Vijay Mohan Hanjoora, Monish Raut, Ashish Sharma, Aseem Srivastava, Saurabh Tiwari, Vartika Vishwani *(Artemis Health Institute, Gurugram)*; Nissi Evelyn. R, Karuna Sree Pendyala, Navakoti Prasad *(Government Dental College and Hospital, Hyderabad)*; Jyoti Bothra, Mainak Deb, Koushik Herle, Harish Jayaram, Lavanya Kannaiyan, Abirami Krithiga, Mukta W *(Rainbow children’s hospital, Hyderabad)*; Dr. Anil Matai, Pooja Nagpal, Prachi Pathak *(Santokh ba Durlabhji memorial hospital, Jaipur)*; Sumit Banerjee, Ramkaran Chaudhary, Gautam Ram Choudhary, Ankita Chugh, Pawan Dixit, Abhay Elhence, Nitesh Gahlot, Mayank Garg, Navdeep Kaur Ghuman, Deepak Jha, Prakash Kala, Amanjot Kaur, Vijay Madduri, Sanjeev Misra, Himanshu Pandey, Puneet Pareek, Manish Pathak, Kirtikumar J Rathod, Mahaveer Singh Rodha, Rahul Saxena, Naveen Sharma, Shashank Shekhar, Mahendra Singh, Pratibha Singh, Bhaskar Suryanarayanan, Jeewan Ram Vishnoi *(All India Institute of Medical Sciences (AIIMS), Jodhpur, Jodhpur)*; Shivang Amin, Vishal Bhende, Rohit Kumar, Tanishq Sharma *(Shree Krishna Hospital, Karamsad)*; Kesavan Murugesan, Sathish Muthu, Abhinav Balachandar Subbiah Ramasamy *(Government Hospital, Velayuthampalayam, Karur)*; Debarshi Chatterjee, C Gerber Gerber, Indranil Ghosh, Upasana Naskar *(Institute of Neurosciences, Kolkata, Kolkata)*; Gaurav Aggarwal, Sanjit Kumar Agrawal, Azaz Ahmed, Sujoy Gupta, Prateek Jain, Deepak Jain, Vishal Kewlani, Amrit Pipara, Noopur Priya, Roopak Raja, Sudip Shakya, Abhishek Sharma, Robin Thambudorai *(Tata Medical Center, Kolkata)*; Dimple Kharkongor, Anjoo Agarwal, Naseem Akhtar, Akshay Anand, Mona Asnani, Ankur Bajaj, Arun Chaturvedi, Akhilanand Chaurasia, Loreno E. Enny, Surabhi Garg, Sameer Gupta, Anoop kumar Jaiswal, Somil Jaiswal, Yashpal Jaware, Navneet Kala, Ruchi Karnatak, Apjit Kaur, Vijay Kumar, Manoj Kumar, Ambrish Kumar, Upander Kumar, Amit kumar Shrivastava, Seema Mehrotra, Brijesh Mishra, Anand Mishra, Dr Namrata, Bal Krishna Ojha, Ahmad Ozair, Uma Shanker Pal, Amita Pandey, Nancy Raja, Shiv Rajan, Pooja Ramakant, Ashutosh Roy, Rekha Sachan, Satyanarayan Sankhwar, Pushp Sankhwar, Divya Sarin, Ayushi Shukla, Sushil Singh, Mohit Singh, Kul Ranjan Singh, Renu Singh, Uma Singh, Urmila Singh, Vandana Solanki, Abhinav Arun Sonkar, Chhitij Srivastava, Parijat Suryawanshi, Vivek Tewarson, Rajat Verma, Manju Lata Verma, Awdhesh Yadav *(King George’s Medical University, Lucknow)*; Gaurav Agarwal, Gyan Chand, K.m.m.vishvak Chanthar, Dileep Hoysal, Anjali Mishra *(Sanjay Gandhi Post Graduate Institute Of Medical Sciences, Lucknow)*; Nitin Batra, Arun Bhatti, Rupali Chopra, Shakina David, Tapasya Dhar, Uma Kant Dutt, Rohini Dutta, Sumir Gandhi, Parvez David Haque, Ritu Jain, Paul Sudhakar John B, Sreejith Kannummal Veetil, Gurvinder Kaur, Navneet Kumar, Anil Luther, Anupam Mahajan, Amit Mahajan, Kavita Mandrelle, Shefin Mathews, Vishal Michael, Partho Mukherjee, Dr Pinki Pargal, Rajesh Paul, Pranay Pawar, Anupam Phillip, Rachel Phillips, Abhishek Samuel, Noel Singh, Inderjot Singh, Abhijit Singh, Sarvpreet Singh Grewal, Anusha Singhania, Selven Thirumalai, Ashish Varghese, Joshua Wesley *(Christian Medical College & Hospital, Ludhiana)*; Philip Alexander, Josy Thomas, Pradeep Zechariah *(Lady Willingdon Hospital, Manali)*; Mariam anjum Ifthikar, Rohan Thomas Mathew, Rohan Shetty, M Vijayakumar Vijaykumar *(Yenepoya medical college hospital, Mangalore)*; BM Zeeshan Hameed, Sufyan Ibrahim, Gayathri Jyothish, Sunil Krishna, Badareesh L, Stanley Mathew, Arjun Suresh kumar *(Kasturba Medical College Hospital, Manipal, Manipal)*; Navneet Kumar Chaudhry, Narinder Singh, Dr piyush kumar Sinha, Rachith Sridhar *(Maharishi Markandeshwar Institute of Medical Sciences & Research, Mullana, Ambala, Haryana)*; Sunny Agarwal, Srikant Balasubramaniam, Lipika Baliarsing, Swati Chhatrapati, Charulata Deshpande, Satish Dharap, Ashni Dharia, Sarita Fernandes, Suraj Gandhi, Mangesh Gore, Abhilash Jayakumar, Rameshwar Mhamane, Anand Nirgude, Sandesh Parab, Manish Patil, Amit Peswani, Anjana Sahu, Sarika Samel, Fagun Shah *(BYL Nair Hospital, mumbai)*; Dipti Haridas, Amruta Kulkarni, Vijay Shetty *(Fortis Hospital Mulund, Mumbai)*; Bejoy Abraham, Varun Agarwal, Quazi Ahmad, Ashishkumar Asari, Mohammad Ismail Attar, Nagaraja Sekhar Ayyalasomayajula, Sutej Bachawat, Dr shivani Bachhav, Vivek Badhe, Sanjiv Badhwar, Shubhabrata Banerjee, Dr VIKAS Basa, Vipul Bothara, Somnath Chattopadhyay, Dr Sohin Chaudhari, Rahul Chavan, Priyank Chawathe, Shailja Dadhich, Anuj Dalal, Avinash Date, Mandar Deshpande, Preetham Dev, Niren Dongre, Anirudha Doshi, Maya Gade, Shreyash Gajjar, Mohan Gawande, Amol Ghalme, Dr bhavisha Ghugare, Ishita Gupta, Manoj Jain, Divakar Jain, Saumya Sekhar Jenasamant, Vaishali Joshi, Vinay Joshi, Neha Kalwadia, Nandkishore Kapadia, Hari Bipin Radhakrishnan Kattana, Tirathram Kaushik, Akshat Kayal, Shama Kovale, Yogesh Kulkarni, Abhaya Kumar, Kranthi Kumar, Kashmira Kumawat, Vidyadhar Lad, Namrata Maskara, Rajesh Mistry, Smruti Ranjan Mohanty, Kanchan Motwani, Manoj Mulchandani, Mandar Nadkarni, Sanjay Pandey, Dr. Mrunal Parab, Dinshaw Pardiwala, Amrita Patkar, Neha Pawar, Abhijit Pawar, Abhinav Pednekar, Vishal Peshattiwar, Harshwardhan Pokharkar, Ojas Potdar, Amit Pothare, Faizan Rahmani, Sunil Rajput, Nalla Ramji Narendra, Anuradha Rao, Suresh Rao, Himanshu Rohela, Rajendra Sakhrekar, Dhanshree Salunkhe, Gursev Sandlas, Hrishikesh Sarkar, Dr Afroz Satpathy, Raghuram Sekhar, Yashwant Shelke, Sanket sadanand Shetty, Dr Shweta Shetye, Anshumala Shukla kulkarni, Umang Singal, Faisal Solanki, Rajendra Sonawane, Raghavendraswami Thete, Yuvaraja Thyavihally, Raj Vhatkar, Sameer Vora, Santosh Waigankar, Shruti Wasnik, Mona Yadav, Rammohan Yedave *(Kokilaben Dhirubhai Ambani Hospital, Mumbai)*; Hriday Acharya, Anant Bangar, Manoj Bharucha, Parag Dhumane, Santosh Karmarkar, Rajesh Nathani, Archana Nehe, Abhay Nene, Webster Jerry Noronha, Naresh Palapalle, Priyank Patel, Dilroop Poyyil, Rajeev Redkar, Munjal Shah, Rahul Deo Sharma, Shruti Tewari *(Lilavati Hospital & Research Centre, Mumbai)*; Ganesh Bakshi, Vikram Chaudhari, Anuja Deshmukh, Ashwin Desoouza, Stuti Gupta, Deepa Nair, Prakash Nayak, Shraddha Patkar, C S Pramesh, Ajay Puri, Sajid Qureshi, Prakash Shetty, Ts Shylasree, Purvi Thakkar, Shivakumar Thiagarajan, Virendra Kumar Tiwari, Saiesh Reddy Voppuru *(Tata Memorial Hospital, Mumbai)*; Rajesh Soni, Anushri Soni, Gira Soni *(Soni Hospital, Nagpur)*; Junaid Alam, Dinesh Bagaria, Minu Bajpai, Akshay Kumar Bisoi, Arvind Chaturvedi, Sandeep Chauhan, Narendra Choudhary, Rajendra Singh Chouhan, Sunil Chumber, Surya Kumar Dube, Kamran Farooque, Vasubabu Gudala, Amit Gupta, Mohit Joshi, Apoorva Kabra, Shashank Sharad Kale, Abhinav Kumar, Subodh Kumar, Dhruv Mahajan, Rajesh Malhotra, P Ramesh Menon, Biplap Misra, Samarth Mittal, Rajinder Parshad, Pratyusha Priyadarshini, Sushma Sagar, Pradeep Brijkishor Sharma, Shilpa Sharma, Vijay Sharma, Vivek Trikha, Mayank Tyagi *(All India Institute of Medical Sciences, New Delhi)*; Kuldeep Bansal, Harvinder Singh, Kalyan Varma *(Indian Spinal Injuries Center, New Delhi)*; Lovenish Bains, Anurag Mishra, Rajdeep Singh *(Maulana Azad Medical College, New Delhi)*; Rohit Bhardwaj, Abhishek Mittal, Sabarirajan Ponnusamy, Gyan Ranjan Singh, Isha Tuli *(Safdarjung Hospital, New Delhi)*; Dr Pramod Bhor, Dr Sanjay Dhar, Rahul Ghodke, Sachin Kale *(Sanjay Clinic & Apollo Hospital, New Mumbai)*; Manisha Aggarwal, Himani Gupta, Gurleen Kaur, Ashwani Kumar *(Government Medical College, Patiala)*; Ajinkya Deshpande, Anup Gadekar, Tanmay Jaysingani, Taufiq Panjwani, Rakesh Patil, Vivek Sodhai, Narendra Vaidya, Dr sunil kumar Vishwakarma, Utkarsha Wayal *(Lokmanya Hospital for Special Surgery, Pune)*; Debnarayan Dutta, Suvendu Maji *(Bengal cancer foundation(BIMS Hospital), Purba Bardhaman)*; Rajnish Arora, Somprakas Basu, Mohit Dhingra, Pankaj Kumar Garg, Amit Gupta, Farhanul Huda, Pankaj Kandwal, Ravi Kant, Rajkumar Kottayasamy Seenivasagam, Navin Kumar, Shashank Kumar, Lokavarapu Manoj joshua, Radheyshyam Mittal, Dharma Ram Poonia, Deepak Rajput, Saravanan Sadhasivam, Dr Sudhir Singh, Vivek Singh *(All India Institute Of Medical Sciences, Rishikesh)*; Kshitija Chandanwale, Chandrashekhar Mahakalkar, Melissa Philip, Vaibhav Thorat *(Acharya Vinoba Bhave Rural Hospital, Sawangi (Meghe), Wardha)*; Yousuf Choudhury, Devishmita Das, Mautushi Das, Subhadra Goala, Ravi Kannan, Farhana yasmin Laskar, Parbin Laskar, Kapil Malik, Poulome Mukherjee, Dr.ABHISHEK Sarkar, S Thoibisana Singha, M Nongalei Singha, Damayanti Singha, Gowtham Srungavarapu, Ritesh Tapkire, Siempui Tling *(Cachar Cancer Hospital and Research Centre, Silchar)*; Syed Muzamil Ishaq Andrabi, Gowhar Aziz Bhat, Nisar Chowdri, Robindera Kour, Asif Mehraj, Fazl Parray, Raahil Shah, Dr ZAMIR AHMAD Shah, Rauf Wani *(Sher-i-Kashmir Institute of Medical Sciences, Srinagar)*; Soujanya Adamala, Ravikanth Gowder, Siddhi Hegde, Mashitha M S, Ranganath N, Shreya Sreeram *(KVG Medical College & Hospital, Sullia)*; Dr.sushil Ankadavar, Darshan Bafna, Dr.Bharat Dhanani, Dr. Nikhil Ingle, Piyush Jadhao, Sinu Joseph, Swapnil Kapote, Shirin Karkada, Vijay Kumar, Parag Lad, Rajan Lohia, Suyog Madje, Venkateshwaran Narasiman, Ashish Phadnis *(jupiter hospital, thane)*; Meer Chisthi, Gejoe George, I Yadev *(Government Medical College Thiruvananthapuram, Thiruvananthapuram)*; Harihara Jothi, Alfie Kavalakat, Dr Dilber Pareed *(Jubilee Mission Medical College & Research Institute, Thrissur)*; Godwin David .C. Mathew, Livingston Abel, Mansi Agrawal, Rabindranath B, Manish Baldia, Ravi Kishore Barla, Manisha Beck, Santosh Benjamin, Jeremy Bliss, Lisa Cherian, Sreekar Devarakonda, Geley Ete, Arun Jacob Philip George, Amish Gohil, Deeptiman James, Mark Ranjan Jesudason, Lallu Joseph, Treasa Joseph, Kathir Joyson, Gomathi Karnan, Albert Kota, Pushplata Kumari, Anitha Loganathan, Vasanth Mark Samuel, John Mathew, Rohin Mittal, Senthil K Nathan, Joby Elizabeth Ninan, Ajay Philip, R.Priyadarshini Priyadarshini, Suganya S, Habie Samuel, Gilbert Samuel, Daniel Selvaraj, Srujan Sharma, Suraj Surendran, Hariharan T D, Santhosh Kumar Thangaraj, Vinotha Thomas, Varghese Thomas, John K Thomas, Harish Y S *(Christian Medical College & Hospital, Vellore)*; Manobhiram Boggavarapu, Karthik Chandra Vallam, Dr.murali Krishna Voonna *(Mahatma Gandhi cancer hospital and research institute, Visakhapatnam)*. |
| **Indonesia**: Marilaeta Cindryani Lolobali, Christopher Ryalino, Tjokorda Gde Agung Senapathi, Mahadewa Tjokorda, i Made Gede Widnyana *(RSUP Sanglah, Denpasar)*; Dita Aditianingsih, Aino Auerkari, Susilo Chandra, Fachreza Aryo Damara, Mohammad Adya Firmansha Dilmy, Achmad Kemal Harzif, Sidharta Kusuma Manggala, Dedy Pratama, Affan Priyambodo, Andi Ade Ramlan, Ratna Farida Soenarto, Adhrie Sugiarto, Ilham Utama Surya, Tamara Tango, Aida Rosita Tantri, Raihanita Zahra *(Cipto Mangunkusumo National General Hospital & Universitas Indonesia, Jakarta)*; Dedy Fachrian, Andi Hasyim, Ade Susanti *(Raden Mattaher General Hospital, Jambi)*; Teddy Saputra, Erwin Syarifuddin *(RS Ibnu Sina YW-UMI, Makassar)*; Andi Asadul Islam, Gabriele Kembuan, Hendra Pajan *(RSUP Dr. Wahidin Sudirohusodo, Makassar)*; Ahmad Hannan Amrullah, Khoirul Anam, Thirza Hadipranata, Julius Albert Sugianto *(Ngimbang General Hospital, Ngimbang)*; Aidyl Fitrisyah, Mayang Indah Lestari, Nur Rachmat Lubis, Rizal Zainal, Zulkifli Zulkifli *(Dr Mohammad Hoesin General Hospital, Palembang)*; Erick Gamaliel Amba, Warren Lie, Andika adiputra Thehumury *(RSUD SAWERIGADING PALOPO, PALOPO)*; Tedy Apriawan, Yunus Kuntawi Aji, Roidah Taqiyya Zahra Wathoni *(dr. Soetomo General Academic Hospital, Surabaya)*; Sumadi Lukman Anwar, Teguh Aryandono, Wirsma Arif Harahap, Juni Kurniawaty, Djayanti Sari, Artanto Wahyono, Yunita Widyastuti, Akhmad Yun Jufan *(Central General Hospital dr. Sardjito, Yogyakarta)*. |
| **Iran, Islamic Rep.**: Parisa Arjmand, Mohammad Etezadpour, Babak Ganjeifar, Masoumeh Hosseinpoor, Hassan Mehrad-Majd, Mohsen Rajati *(Ghaem Teaching Hospital, Mashhad)*; Reza Assadi, Ehsan Noori, Parisa Rajaei *(Imam Reza hospital, Mashhad)*; Samira Hajisadeghi, Mohammad Mehdizadeh, Mina Soltani *(Fatemiyeh Oral and Maxillofacial Surgery Center, Qom)*; Narges Alizadeh, Gholamreza Azarnia Azar Nia, Hamid Heidari, Seyedmohamad Hosseini Zavareh Hosseini Zavareh, Ali Moazami Pour Moazami Pour, Farokh Savaddar, Mostafa Vahedian *(Forqani Hospital, Qom)*; Hoora Amouzegar, Gholamreza Azarnia Azar Nia, Mojdeh Bahadorzadeh, Seyedeh Homa Hemmasi, Ahmad Kachoie, Ali Karimi Karimi, Sepideh Miraj, Monireh Mirzaie, Hossein Mokarami Mokarami, Amrollah Salimi *(Imam Reza Hospital, Qom)*; Roghayyeh Ahangari, Ali Ahmadvand, Ali Bashiri, Morteza Borhani, Mohammad Haidari, Mohammad Taghi Imani Khosroshahi, Shahrokh Jahan Bini, Mohsen Koosha, Mohammad Kazem Moslemi, Ali Naghibi, Shahrzad Tehrani, Zahra Yazdi *(Izadi Hospital, Qom)*; Seyyed Hassan Adeli, Majid Alborzi, Hamed Bagheri, Mohsen Eshraghi, Hassan Fatemi manesh, Mohammad Ghomeisi, Seyed fakhreddin Hejazi, Ahmad Kachoie, Saeed Madani, Nima Najafian motahaver, Samieh Norouzi, Mahdi Pezeshki Modarres, Ali Shafiee, Jamshid Vafaeimanesh, Hossein Yusefi *(Shahid Beheshti Hospital, Qom)*; Ahmad Kachoie, Saeed Madani *(Shohada Hospital, Qom)*; Elahe Hosseini, Pourya Medhati, Hamed Nikoupour *(Abu-Ali Sina Hospital Shiraz, Shiraz)*; Kayvan Aghazadeh, Shahin Bastaninejad, Payman Dabirmoghaddam, Reza Erfanian, Mohammadreza Firouzifar, Farrokh Heidari, Shirin Irani, Ebrahim Karimi, Ali Kouhi, Masoud Motasaddi zarandy, Mahtab Rabbani Anari, Saleh Sandoughdaran, Saeed Sohrabpour, Ardavan Tajdini, Nasrin Yazdani *(Amir Alam Hospital, Tehran)*; Hamed Akhavizadegan, Esmaeil Rezghi Maleki, Naser Yousefzadeh Kandevani *(Baharloo hospital, Tehran)*; Nima Bagheri, Seyed Amir Javadi, Seyed Hadi Kalantar, Farzaneh Keneshlou, Zahid Hussain Khan, SM Javad Mortazavi *(Imam Khomeini Hospital Complex(IKHC), Tehran)*; Sayedali Ahmadi, Jaber Hatam, Mohammad Hossein Khosravi *(Rasool-e-Akram Hospital, Tehran)*; Mohammad Hossein Nabian, Leila Oryadi zanjani, Fardis Vosoughi *(Shariati Hospital, Tehran)*; Mohammadreza Golbakhsh, Seyyed Hossein Shafiei, Babak Siavashi *(Sina Hospital, Tehran)*. |
| **Iraq**: Ali Al-Isawi, Mohammed Al-Masood *(Al-hillah Teaching Hospital, Babil)*; Dania Al-Najjar *(AlKhayal Medical Centre, Baghdad)*; Yarub Gahtan, Sara Nabil *(Alkindy teaching hospital, Baghdad)*; Najat Abdul Hameed, Jumana Abdul Hameed, Abdullah Ahmed *(Baghdad Medical City, Baghdad)*; Ahmed Hilmi *(Ibn Sina, Baghdad)*; Ali Akadh, Rand Hussein *(Zafaraniyah General Hospital, Baghdad)*; Abbas Aljebur, Sadik Hassan, Haithem Hussein Ali *(Basra children speciality hospital, Basra)*; Fahad Al-Hasani, Mustafa Wameedh Ibrahim, Mubder Mohammed Saeed *(Basra Teaching Hospital, Basra)*; Haidar Muhssein *(University of Kufa college of medicine, Najaf)*. |
| **Ireland**: Success Akindoyin, Hilary Ikele, Catherine McNestry *(Mayo University Hospital, Castlebar)*; Stevie Barry, Nikhil Dewan, Bosom Ekwere, Murtaza Essajee, Ream Langhe, Calista Marshall, Darya Musa *(Cavan General Hospital, Cavan)*; Zeeshan Ahmad, Emmet Andrews, Bruno Chan Chin, Mark Corrigan, Amy Edwards Murphy, Christina Fleming, Niamh Foley, Padraig Gardiner, Daniel Hechtl, Michelle Hsiao, Mohd yasser Kayyal, Shane Killeen, Maria Lyons, Stephen O’Brien *(Cork University Hospital, Cork)*; Cathy Burke, Matt Hewitt, Marwa Mohamed, Syeda Farah Nazir, Mei Yee Ng *(Cork University Maternity Hospital, Cork)*; Nor Azlia Abdul Wahab, Cathy Monteith, Oladayo Oduola *(Our Lady of Lourdes Hospital Drogheda, Drogheda, Co louth)*; Lylas Aljohmani, Timothy Nugent *(Beacon Hospital, Dublin)*; Sami Abd Elwahab, Paula Corr, Lauren Crone, Niall Davis, Johnathon Harris, Arnold Hill, Mohsen Javadpour, David Kearney, Robert Anthony Keenan, Deirdre Nolan, James Ryan *(Beaumont Hospital, Dublin)*; Mark Philip Hehir, Brendan McDonnell, Carmen Regan *(Coombe Women and Infants University Hospital, Dublin)*; Michael Geary, Fergal Malone, Claire McCarthy, Clare O Connor, Donal B O’Connor *(Rotunda Hospital, Dublin)*; Jarlath Bolger, Cillian Clancy, Shane Considine, Stefanie M Croghan, Noel Donlon, Emma Donohoe, Caroline Herron, John Larkin, Thomas Hugh Lynch, Barry Maguire, Rustom Manecksha, Andrea Mc Carthy, Katharina Nagassima, Erica O’Sullivan, Pat Rohan, Ryan Roopnarinesingh, Salloum Salloum *(St James’s Hospital, Dublin)*; Thomas Aherne, Mary Barry, Gareth John Bowen, Ellen Boyle, Carolyn Cullinane, Joseph Dowdall, Ann Hanly, Ahmed Hassanin, Helen Heneghan, Conor Hurson, Orlaith Kelly, Rory Kennelly, Aoife Kiernan, Sean T Martin, Nawar Masarani, Damian McCartan, Enda W McDermott, Ben Murphy, Kin Cheung Ng, Nwabundo Njeze, Aine O’Neill, Ruth S Prichard, Ned Quirke, Ian Sean Reynolds, Des Winter *(St Vincent’s University Hospital, Dublin)*; Rowan Casey, Ben Creavin, Mutaz Elamin, Amy Gillis, Dara Kavanagh, Michael Kelly, Áine McNamee, Muheilan Muheilan, Paul Neary, Patrick Owens, Akshaya Ravi, Paul Ridgway *(Tallaght Hospital, Dublin)*; Paul Carroll, Chris Collins, Amenah Dhannoon, Helen Earley, Amy Fowler, Aisling Hogan, Aoife Lowery, Peter McAnena, Charlie Timon, Stewart Walsh *(University Hospital Galway, Galway)*; Wisam Al-Ramli, Imran Azeem, Muhammad Usaama Bahadoor, Zsolt Bodnar, Hassan Elmusharaf, Faisal Saeed Hassan, Adam Hingum, Huilun Huan, Seamus Jennings, Syed Mohammad Umar Kabir, Mohamed Hamed Khalid, Mariya Kuteva, Syed Nadeem Mujtaba, Shanell Peeriyah, Hina Rehman, Muhammad assam Sarwar, Tony Shaju, Ian Stephens, Michael Sugrue, Joseph Thomas, Manvydas Varzgalis, Saqib Zeeshan *(LETTERKENNY UNIVERSITY HOSPITAL, Letterkenny)*; Alisha Jaffer, Larne Jones-Whiting, Colin Peirce *(University Hospital Limerick, Limerick)*; Sean Johnston, Seantee Lim, Éanna Ryan *(Tullamore University Hospital, Tullamore)*; Alwaleed Abdelgadir, Sara Ahmed, Youssef Al-Mukhaizeem, Tara Connelly, Fiachra Cooke, Clare Crowley, Ivor Cullen, Michael Flanagan, Amy Fogarty, Orna Glynn, Claudia Guerrero Martinez, Mohamed Alfatih Hamza, Mekki Hassan, Ibrahim Hegazy, Rhodri Hill, Amr Kazim, Azriny Shaziela Khalid, Muhammad Abdullah Khalid, Zubair Majeed, Aidan Manning, Peter McCullough, Seamus Murphy, Peter Neary, Anthony Noone, Gerrard O’Donoghue, John O’Kelly, Eddie Odonnell, Elaf Osman, Jessica Ryan, Rafeh Saeed *(University Hospital Waterford/University College Cork, Waterford)*. |
| **Israel**: Ahmad Abo Arar, Arsan Abu abed, Daniel Dykman, Ahmad Elnassasra, Inbar Gatot, Haim Gavriel, Ruthie Gold- Deutch, Nadav Haim, Jonathan Hammerschlag, Yehuda Hershkovitz, Ahmad Jaber, Adi Kenoshi, Ron Lavy, Omar Majadla, Limor Muallem-Kalmovich, Hilli Nativ, Igor Rabin, Yael Sandler, Oded Zmora, Osnat Zmora *(SHAMIR MEDICAL CENTER, BE’ER YA’AKOV)*; Miklosh Bala, Yonatan Avraham Demma, Yuri Fishman, Gad Marom *(Hadassah Medical Center, Jerusalem)*; Naor Avni, Ofra Carmel, Amicur Farkas, Michael Ron Freund, Lior Gonen, Yaacov Gozal, Dmitry Greenman, Stanislav Kocherov, Nevo Margalit, Orit Nahtomi Shick, Israel Alexander Ostrovsky, Rivka Pardes, Michal Perets, Tal Shahar, Henry Shapiro, James Tankel, David Teren, Reuven Yahud, Shlomo Yellinek *(Shaare Zedek Medical Center, Jerusalem)*. |
| **Italy**: Paolo Balercia, Lisa Catarzi, Giuseppe Consorti *(University Hospital Umberto, Ancona)*; Pasquale Cianci, Domenico Gattulli, Marina Minafra, Enrico Restini *(Lorenzo Bonomo, andria)*; Enrico Andolfi, Filippo Annino, Frezza Barbara, Alessia Biancafarina, Edoardo Bussolin, Marco De Prizio, Ulpjana Gjondedaj, Marilena Gubbiotti, Gianni Mura, Giuseppe Antonino Pellicano’, Giacomo Maria Pirola, Rezart Sulce *(Ospedale San Donato USL Toscana Sud Est, Arezzo)*; Gennaro Martines, Vincenzo Papagni, Arcangelo Picciariello *(Azienda Ospedaliero Universitaria Consorziale Policlinico Di Bari, Bari)*; Stefano Magnone, Michele Pisano, Elia Poiasina *(Papa Giovanni XXIII Hospital, Bergamo)*; Laura Alberici, Filippo Antonacci, Alessandro Arena, Angela Belvedere, Fabio Bernagozzi, Paolo Bernante, Pietro Bertoglio, Lorenzo Bianchi, Maria Bisulli, Barbara Bonfanti, Safia Boussedra, Jury Brandolini, Crescenzo Cacciapuoti, Stefano Cardelli, Riccardo Casadei, Matteo Cescon, Alessandro Cipolli, Riccardo Cipriani, Luca Contu, Francesco Costa, Niccolo’ Daddi, Eugenia De Crescenzo, Pierandrea De Iaco, Alessandra De Palma, Massimo Del Gaudio, Anna Nunzia Della Gatta, Giampiero Dolci, Giulia Dondi, Matteo Droghetti, Sergio Nicola Forti Parri, Elena Garelli, Chiara Gelati, Giuliana Germinario, Federico A. Giorgini, Carlo Ingaldi, Elio Jovine, Kenji Kawamukai, Antonio Lanci Lanci, Raffaele Lombardi, Maria Elisa Lozano Miralles, Claudio Marchetti, Michele Masetti, Francesco Minni, Daniele Morezzi, Daniele Parlanti, Alice Pellegrini, Anna Myriam Perrone, Anna paola Pezzuto, Marco Pignatti, Gianluigi Pilu, Valentina Pinto, Gilberto Poggioli, Silvana Bernadetta Puglisi, Diego Raimondo, Matteo Ravaioli, Claudio Ricci, Sara Ricciardi, Francesco Ricotta, Roberta Rizzo, Angela Romano, Matteo Rottoli, Riccardo Schiavina, Renato Seracchioli, Matteo Serenari, Margherita Serra, Piergiorgio Solli, Gioia Sorbi, Mario Taffurelli, Marta Tanzanu, Achille Tarsitano, Marco Tesei, Gabriele Vago, Tommaso Violante, Simone Zanotti *(IRCCS Azienda Ospedaliero-Universitaria di Bologna, Bologna)*; Raffaele Aspide, Giacomo Bertolini, Carlo Bortolotti, Alessandro Carretta, Ambra Caruso, Alfredo Conti, Carla De Vita, Filippo Friso, Emanuele La Corte, Diego Mazzatenta, Alessandro Pirina, Vittoria Rosetti, Carmelo Sturiale, Matteo Vincenzi, Matteo Zoli *(IRCCS Istituto delle Scienze Neurologiche di Bologna, Bologna)*; Francesco Castagnini, Davide Maria Donati, Tommaso Frisoni, Stefano Lucchini, Emanuela Palmerini, Francesco Traina *(IRCCS Istituto Ortopedico Rizzoli, Bologna)*; Anna Maria Baietti, Bruno Berselli, Silvia Bolognesi, Erich Fabbri, Francesco Farnia, Pietro Maremonti, Concetta Marganella, Ernesto Pasquini, Vito Antonio Piserchia, Gian Marco Prucher, Alessandra Razzaboni, Silvia Ricci, Giacomo Sollini, Caterina Testoni *(Ospedale Maggiore/Bellaria Carlo Alberto Pizzardi AUSL Bologna, Bologna)*; Mohammed Abu Hilal, Nine de Graaf, Roberta La Mendola *(Fondazione Poliambulanza, brescia)*; Giulia Arrigoni, Gian Luca Baiocchi, Elena Cagnazzi, Rossella D’Alessio, Francesco Doglietto, Federico Ferrari, Marco Fontanella, Batog Igor, Sarah Molfino, Franco Odicino, Pier Paolo Panciani, Giorgio Saraceno, Enrico Sartori, Luca Zanin *(ASST Spedali Civili, Ospedale di Brescia, Brescia)*; Giuseppe Esposito, Federica Frongia, Adolfo Pisanu, Mauro Podda *(Cagliari University Hospital, Cagliari)*; Nicola Cillara, Alessandro Cannavera Putzu, Raffaele Sechi *(Santissima Trinità - ATS Sardegna, Cagliari)*; Emmanuele Abate, Massimiliano Casati, Letizia Laface, Marcello Schiavo *(Ospedale Vittorio Emanuele III - Carate Brianza, Carate Brianza (MB))*; Fabio Marino, Fabrizio Perrone *(IRCCS ‘Saverio de Bellis’, Castellana Grotte (Ba))*; Paolo Annicchiarico, Alessandro Cappellani, Matteo Cavallo, Alessia Giaquinta, Rossella Gioco, Massimiliano Veroux, Pierfrancesco Veroux, Antonino Zanghì *(Azienda Ospedaliero- Universitaria Policlinico San Marco, Catania)*; Antonio Cianci, Arturo Lo Giudice, Maria Grazia Matarazzo, Giorgio Ivan Russo, Giuseppe Sarpietro, Carmen Emanuela Scandura *(Policlinic Hospital ‘G. Rodolico’, Catania)*; Ida Barca, Adriano Carnevali, Antonio Carpino, Maria Giulia Cristofaro, Gilda De Paola, Giuseppe Giannaccare, Daniela Novembre, Giuseppe Sammarco, Vincenzo Scorcia, Angeli Christy Yu *(University ‘Magna Graecia’ of Catanzaro, Catanzaro)*; Marcello D’Andrea, Lorenzo Mongardi, Luigino Tosatto *(Ospedale M. Bufalini, Cesena)*; Mirko Barone, Felice Mucilli, Angelo Muraglia *(Policlinico Santissima Annunziata, Chieti)*; Ottavia Caserini, Domenico Benvenuto Giuliani, Marco Monti, Alessia Morello, Edoardo Segalini *(ASST Crema, Crema)*; Felice Borghi, Desiree Cianflocca, Alberto Daniele, Danilo Donati, Enrico Gelarda, Giorgio Giraudo, Maria Carmela Giuffrida, Alessandra Marano, Elena Olearo, Vincenzo Pruiti Ciarello, Andrea Puppo, Valentina Testa *(Santa Croce e Carle Hospital, Cuneo, Cuneo)*; Marco Giacometti, Sandro Zonta *(San Biagio Hospital, Domodossola - VB, Domodossola)*; Eleonora Monti, Andrea Porta, Daniele Sambucci *(Ospedale Sacra Famiglia Fatebenefratelli, Erba)*; Arianna Birindelli, Barbara Carrara, Bruno Compagnoni, Roberto Del Giudice, Sara Elisabetta Dester, Daniele Lomiento, Silvia Ruggiero, Lucio Taglietti, Fabio Viotti *(ASST Valcamonica Ospedale di Esine, Esine)*; Domenico Lacavalla, Savino Occhionorelli, Michele Rubbini *(Azienda Ospedaliero Universitaria San’Anna, Ferrara)*; Massimiliano Bernabei, Nicolò Fabbri, Marta Fazzin, Carlo V. Feo, Marco Torchiaro *(Azienda Unità Sanitaria Locale di Ferrara, Ferrara)*; Renato Costi, Edoardo Virgilio *(di Vaio Hospital, Fidenza)*; Lorenzo Arlia, Giuseppe Barbato, Ilenia Bartolini, Andrea Bottari, Chiara Bruno, Alessandro Bruscino, Carlotta Checcucci, Fabio Cianchi, Francesco Coratti, Rosita De Vincenti, Annamaria Di Bella, Massimiliano Fambrini, Laura Fortuna, Oreste Gallo, Giacomo Gigliucci, Luca Giovanni Locatello, Gherardo Maltinti, Jacopo Martellucci, Paolo Prosperi, Maria Novella Ringressi, Flavia Sorbi, Fabio Staderini, Antonio Taddei *(Azienda Ospedaliera Universitaria Careggi, Firenze)*; Alessandro Anastasi, Giuseppe Canonico, Emiliano Chisci, Linda Gabellini, Fabrizio Masciello, Stefano Michelagnoli, Tommaso Nelli, Luca Tirloni *(Ospedale San Giovanni di Dio, Firenze)*; Giovanni Alemanno, Armando Arminio, Renata Beck, Carlo Bergamini, Antonella Cotoia, Vincenzo Lizzi, Giuseppe Maccagnano, Vito Pesce, Antonio Luciano Sarni, Nicola Tartaglia, Fernanda Vovola *(Ospedali Riuniti Azienda Ospedaliera Universitaria, Foggia)*; Andrea Avanzolini, Antonio Bocchino, Raffaele Bova, Davide Cavaliere, Fabrizio D’acapito, Giorgio Ercolani, Francesca Fappiano, Carlo Alberto Pacilio, Leonardo Solaini *(Morgagni-Pierantoni, Forlì)*; Giancarlo D’Andrea, Laura Lavalle, Veronica Picotti *(Fabrizio Spaziani, Frosinone 03100)*; Andrea Barberis, Marco Filauro, Matteo Santoliquido *(E.O. Ospedali Galliera, Genoa)*; Alessandra Aprile, Fabio Barra, Raffaele De Rosa, Raquel Diaz, Simone Ferrero, Piero Fregatti, Claudio Gustavino, Michele Iester, Chiara Kratochwila, Andrea Massobrio, Davide Pertile, Stefano Scabini, Umberto Scovazzi, Domenico Soriero, Marco Sparavigna, Carlo Traverso, Aldo Vagge *(IRCCS Ospedale Policlinico San Martino, Genoa)*; Denise Gambardella, Manfredo Tedesco *(Giovanni Paolo II Hospital, Lamezia Terme)*; Stefano D’Ugo, Norma Depalma, Marcello Giuseppe Spampinato *(P.O.’Vito Fazzi’, Lecce)*; Angelo Airoldi, Ariberto Brivio, Marco Chiarelli, Ludovica Gibelli, Bonfanti Giulia, Samuele Grandi, Giovanni Pesenti, Fulvio Tagliabue, Mauro Zago *(ASST di Lecco - P.O di Lecco, Lecco)*; Riccardo Lenzi, Jacopo Matteucci, Luca Muscatello *(Ospedale Apuane, Massa)*; Gaia Colletti, Marco Guido Confalonieri, Andrea Costanzi, Colomba Frattaruolo, Andrea Locatelli, Michela Monteleone *(San Leopoldo Mandic, Merate (LC))*; Giorgio Badessi, Maria Caffo, Gerardo Caruso, Eugenio Cucinotta, Antonino Francesco Germano’, Carmelo Mazzeo *(Policlinico Universitario G. Martino of Messina, Messina)*; Camillo Leonardo Bertoglio, Paolo De Martini, Giovanni Ferrari, Alessandro Giani, Pietro Maria Lombardi, Michele Mazzola *(ASST Grande Ospedale Metropolitano Niguarda, Milan)*; Ludovica Baldari, Daniele Bissacco, Luigi Boni, Elisa Cassinotti, Maurizio Domanin, Lorenzo Pignataro, Sara Torretta, Santi Trimarchi *(Fondazione IRCCS Ca’ Granda - ospedale Maggiore Policlinico, Milan)*; Daniele Armellin, Silvia Basato, Laura Bernardi, Francesca Bunino, Giovanni Capretti, Francesco Maria Carrano, Carlo Castoro, Giovanni Colombo, Andrea Costantino, Francesca De Lucia, Armando De Virgilio, Matteo Di Bari, Fabio Ferreli, Francesca Gaino, Marco Gramellini, Carlotta La Raja, Luca Malvezzi, Salvatore Marano, Giuseppe Mercante, Flavio Milana, Gennaro Nappo, Georgios Peros, Francesca Pirola, Vanessa Rossi, Elena Russo, Giuseppe Spriano, Sara Tamburello, Alessandro Zerbi *(Humanitas Research Hospital, Milan)*; Alberto Aiolfi, Davide Bona, Andrea Sozzi *(Istituto Clinico Sant’Ambrogio, Milan)*; Laura Adamoli, Mohssen Ansarin, Luca Bertolaccini, Sabine Cenciarelli, Francesco Chu, Rita De Berardinis, Uberto Fumagalli Romario, Giacomo Pietrobon, Giulia Sedda, Lorenzo Spaggiari, Marta Tagliabue *(Istituto Europeo di Oncologia - IRCCS -Milano, Milan)*; Antonella Ardito, Maria Caputo, Paola Cellerino, Valentina D’alessandro, Elisa Galfrascoli, Maria Paola Giusti, Marco Lotti, Roberto Santambrogio, Marco Antonio Zappa *(Ospedale Fatebenefratelli e Oftalmico, Milan)*; Andrea Bondurri, Alessandro Michele Bonomi, Francesco Colombo, Michele Achille Crespi, Piergiorgio Danelli, Angelo Gabriele Epifani, Luca Ferrario, Alice Frontali, Claudio Guerci, Anna Maffioli *(Ospedale Luigi Sacco Milano, Milan)*; Francesco Ferrara *(San Carlo Borromeo, Milan)*; Luca Antonio Aldrighetti, Domenico Baccellieri, Luca Bertoglio, Giulia Bonavina, Massimo Candiani, Giorgio Candotti, Arianna Casiraghi, Laura Mariangela Castellano, Paolo Ivo Cavoretto, Roberto Chiesa, Federica Cipriani, Paola De Nardi, Guido Fiorentini, Filippo Gagliardi, Alessandro Galdini, Alessandro Grandi, Elena Marotta, Simonetta Massaron, Andrea Melloni, Pietro Mortini, Gianluca Nocera, Martina Piloni, Mirko Pozzoni, Francesca Ratti, Riccardo Rosati, Alessandro Ferdinando Ruffolo, Pierpaolo Sileri, Alfio Spina, Andrea Vignali *(San Raffaele Scientific Institute, Milan, Milan)*; Cristina Barberio, Luigi Beretta, Francesca Cavenago, Nora Di Tomasso, Stefano Fresilli, Giovanni Landoni, Stefano Lazzari, Gaetano Lombardi, Marilena Marmiere, Fabrizio Monaco, Gabriele Todaro, Stefano Turi, Alberto Zangrillo *(Università Vita-Salute San Raffaele, Milan)*; Francesca Bertolina, Giorgio Bogani, Stefano Bonomi, Pierfrancesco Cadenelli, Valentina Chiappa, Stefano Piero Bernardo Cioffi, Davide Citterio, Lara Valentina Comini, Umberto Cortinovis, Maurizio Cosimelli, Antonino Ditto, Marco Fiore, Massimiliano Gennaro, Lorenzo Giannini, Alessandro Gronchi, Marcello Guaglio, Marco Guzzo, Andrea Leva, Alberto Macchi, Elena Manzo, Fabio Martinelli, Ilaria Mattavelli, Vincenzo Maria Mazzaferro, Francesco Raspagliesi, Luigi Rolli, Laura Sala, Roberto Salvioni, Mario Santinami, Silvia Segattini, Luca Sorrentino, Carlotta Zaborra *(Fondazione IRCCS Istituto Nazionale dei Tumori, Milano, Milano)*; Alexandre Anesi, Gianmaria Casoni Pattacini, Mattia Di Bartolomeo, Francesca Pecchini, Arrigo Pellacani, Micaela Piccoli *(Azienda Ospedaliero Universitaria di Modena, Modena)*; Federico Fusini, Andrea Gattolin, Marco Migliore, Roberto Rimonda, Diego Sasia, Elisabetta Travaglio *(Regina Montis Regalis Hospital, Mondovì, Mondovì)*; Federica Brunetti, Marco Cereda, Marco Ceresoli, Luca Cigagna, Cristina Dell’Oro, Alessandro Fogliati, Robert Fruscio, Tommaso Grassi, Maini Marzia Isabella, Luca Carlo Nespoli, Massimo Oldani, Sara Ornaghi, Nicolò Tamini *(Ospedale San Gerardo, Monza)*; Carmine Antropoli, Antonio Castaldi, Alessio Palumbo *(Azienda Ospedaliera di rilievo nazionale Antonio Cardarelli, Naples)*; Gaia Altieri, Umberto Bracale, Francesco Corcione, Marcello De Luca, Giovanni Domenico De Palma, Maria Michela Di NUZZO, Ruggero Lionetti, Dalila Loredana Lo Bue, Gaetano Luglio, Gianluca Pagano, Roberto Peltrini, Nello Pirozzi, Francesca Paola Tropeano *(Federico II University of Naples, Naples)*; Alessia Aversano, Andrea Belli, Maria D’amico, Paolo Delrio, Francesco Izzo, Renato Patrone, Daniela Rega *(Istituto Nazionale Tumori Fondazione, Pascale IRCCS, Naples)*; Pietro Maida, Ester Marra, Gianpaolo Marte, Andrea Tufo *(ospedale del mare, Naples)*; Francesco Bianco, Antonio Cappiello, Simona Gili, Paola Incollingo, Alessandra Novi *(Ospedale S. Leonardo - ASL Napoli 3 sud, Castellammare di Stabia, Naples)*; Giulia Bagaglini, Claudio Iovino, Francesco Menegon Tasselli, Maria Paola Menna, Francesco maria Romano, Settimio Rossi, Guido Sciaudone, Francesco Selvaggi, Lucio Selvaggi, Francesca Simonelli *(Universitá della Campania ‘Luigi Vanvitelli’, Naples, Naples)*; Guido Coretti, Mario Pannullo, Adolfo Renzi *(Ospedale Fatebenefratelli, Napoli)*; Francesca Ascari, Giuliano Barugola, Giacomo Ruffo *(IRCCS Ospedale Sacro Cuore Don Calabria, Negrar di Valpolicella (Verona))*; Paolo Baroffio, Paolo Bellora, Laura Enrica Benedetti, Cristina Cerri, Giordana D’Aloisio, Maurizio Ferrari, Elisa Francone, Sergio Gentilli, Herald Nikaj *(Azienda Ospedaliero Universitaria Maggiore della Carità, Novara)*; Antonella Chessa, Alessandro Fiorini *(San Giovanni di Dio, Orbetello)*; Luca Campagnaro, Franco Chioffi, Pietro Ciccarino, Roberto Colasanti, Francesco de Falco, Fotios Kalfas, Gioacchino Mattisi, Giulia Nezi, Matteo Palma, Angelica Rizzoli, Domenico Rossi, Davide Russo, Renato Salvador, Francesco Volpin *(Azienda Ospedaliera di Padova, Padova)*; Guido Bissolotti, Stefano Fusetti, Francesco Lemma *(University of Padova, Padova)*; Vito Chiantera, Mariano Catello Di Donna, Giulio Sozzi *(ARNAS Civico Hospital, Palermo)*; Emanuele Cammarata, Sofia Campanella, Daniela Canzonieri, Adriana Cordova, Federico De Michele, Ettore Dinoto, Mara Franza, Leo Licari, Daniele Matta, Domenico Mirabella, Felice Pecoraro, Roberto Pirrello, Pierfrancesco Pugliese, Fernando Rosatti, Giuseppe Salamone, Francesca Toia, Massimiliano Tripoli *(Department of Surgical, Oncological and Oral Sciences. University of Palermo, Palermo)*; Cosimo Callari, Dario Di Miceli, Leo Licari *(FBF Buccheri La Ferla Palermo, Palermo)*; Alfredo Annicchiarico, Luca Bellanti, Michela Bergonzani, Roberto Berretta, Elisa Cabrini, Vito Andrea Capozzi, Fausto Catena, Federico Cozzani, Paolo Del Rio, Marco Domenichini, Anna Fornasari, Antonio Freyrie, Tiziana Frusca, Mario Giuffrida, Gennaro Perrone, Giulia Rossi, Matteo Rossini, Andrea Varazzani *(Azienda Ospedaliero - Universitaria di Parma, Parma)*; Vittorio Arici, Antonio Bozzani, Lorenzo Cobianchi, Matteo Filardo, Marika Sharmayne Milani, Franco Ragni *(Policlinico San Matteo, Pavia)*; Andrea de Manzoni Garberini *(Ospedale Civile Spirito Santo, Pescara)*; Edoardo Baldini, Diana Carpaneto, Michele Cauteruccio, Corrado Ciatti, Luigi Conti, Serena Gattoni, Pietro Maniscalco, Gerardo Palmieri, Calogero Puma Pagliarello *(G. Da Saliceto, Piacenza)*; Giuseppe Caristo, Raffaele Galleano, Michele Malerba *(Ospedale Santa Corona, Pietra Ligure (SV), Pietra Ligure)*; Marcello Calabrò, Francesca Farnesi, Elia Giuseppe Lunghi, Andrea Muratore, Nicoletta Sveva Pipitone federico *(Edoardo Agnelli, Pinerolo)*; Joel Reuben Abel, Lorenzo Andreani, D’arienzo Antonio, Vittorio Aprile, Riccardo Balestri, Giacomo Benettini, Stefano Berrettini, Luca Bruschini, Massimo Chiarugi, Federico Coccolini, Simone Colangeli, Camilla Cremonini, Lodovica Cristofani Mencacci, Iacopo Dallan, Silvia De Santi, Gregorio Di Franco, Lorena Di Girolami, Giacomo Fiacchini, Niccolò Furbetta, Stylianos Korasidis, Marco Lucchi, Andrea Morandi, Luca Morelli, Serena Musetti, Carlo Maria Neri, Matteo Palmeri, Miriana Picariello, Francesco Porcelli, Marco Puccini, Nicolo’ Roffi, Erica Statuti, Dario Tartaglia, Alberto Tonelli, Matteo Vianini *(Azienda Ospedaliero Universitaria Pisana, Pisa)*; Gianluca Baronio, Mauro Montuori, Enrico Pinotti *(Policlinico San Pietro, Ponte San Pietro)*; Stefano Maria Massimiliano Basso, Federica Maffeis, Paolo Ubiali *(Azienda Per L’assistenza Sanitaria N. 5 Friuli Occidentale, Pordenone)*; Lorenzo Aguzzoli, Saverio Coiro, Giuseppe Falco, Vincenzo Dario Mandato, Valentina Mastrofilippo, Simone Mele *(Azienda Unità Sanitaria Locale - IRCCS di Reggio Emilia, Reggio Emilia)*; Caterina Baldi, Carlo Corbellini, Gianluca Matteo Sampietro *(Ospedale Di Rho - ASST Rhodense, Rho)*; Massimo Dugo, Mauro Garino, Chiara Marafante, Antonio Masciandaro, Elisabetta Moggia, Alessandra Murgese *(Ospedale degli Infermi di Rivoli, Rivoli)*; Felice Eugenio Agro’, Gabriella Teresa Capolupo, Filippo Carannante, Marco Caricato, Vincenzo Denaro, Erica Mazzotta, Rocco Papalia, Giuseppe Pascarella, Alessandro Strumia, Biagio Zampogna *(policlinico universitario campus bio medico of rome, rome)*; Matteo Cinquepalmi, Marco Colasanti, Celeste Del Basso, Federica Falaschi, Nicola Guglielmo, Roberto Luca Meniconi, Alessandra Pecoraro, Sofia Usai *(Azienda Ospedaliera San Camillo - Forlanini, Rome)*; Giacomo Crescentini, Antonella Larcinese, Emanuele Picone, Giovanni Sinibaldi *(Fatebenefratelli Isola Tiberina, Rome)*; Annamaria Agnes, Salvatore Agnes, Sergio Alfieri, Francesco Belia, Valentina Bianchi, Giuseppe Bianco, Alberto Biondi, Paola Campennì, Valerio Cozza, Sabatino D’Archi, Domenico D’Ugo, Veronica De Simone, Marta Di Grezia, Sofia Di Lorenzo, Federica Ferracci, Valeria Fico, Gianluca Franceschini, Pietro Fransvea, Giulio Gasparini, Luca Gordini, Antonio La Greca, Francesco Litta, Celestino Pio Lombardi, Angelo Alessandro Marra, Angelo Parello, Marco Maria Pascale, Romeo Patini, Gilda Pepe, Roberto Persiani, Caterina Puccioni, Carlo Ratto, Fausto Rosa, Gianmarco Saponaro, Lorenzo Scardina, Tedesco Silvia, Giuseppe Tropeano *(Fondazione Policlinico Universitario Agostino Gemelli, Rome)*; Maria Benevolo, Daniele Bugada, Flaminia Campo, Maria Gabriella Dona’, Valentina Manciocco, Paolo Marchesi, Riccardo Mastroianni, Francesco Mazzola, Silvia Moretto, Raul Pellini, Gerardo Petruzzi, Barbara Pichi, Giuseppe Simone, Gabriele Tuderti, Jacopo Zocchi *(IRCCS ‘Regina Elena’ National Cancer Institute, Rome)*; Luigi Marino Cosentino, Andrea Sagnotta *(Ospedale San Filippo Neri, Rome)*; Roberta Angelico, Vittoria Bellato, Michela Campanelli, Marzia Franceschilli, Michele Grande, Giorgio Lisi, Tommaso Maria Manzia, Lorenzo Petagna, Bruno Sensi, Giuseppe Sica, Leandro Siragusa, Giuseppe Tisone *(Policlinico Tor Vergata Hospital, Rome, Rome)*; Marco Assenza, Barbara Binda, Massimo Biondi, Gioia Brachini, Placido Bruzzaniti, Mauro Casagrande, Flavia Ciccarone, Pierfranco Maria Cicerchia, Bruno Cirillo, Daniele Crocetti, Giancarlo D’ambrosio, Vito D’andrea, Francesca De Felice, Giorgio De Toma, Carlo Della Rocca, Giulia Duranti, Pietro Familiari, Enrico Fiori, Giovanni Battista Fonsi, Alessandro Frati, Stefania La Rocca, Filippo La Torre, Pierfrancesco Lapolla, Giovanni Marruzzo, Simona Meneghini, Andrea Mingoli, Francesco Pata, Andrea Picchetto, Antonella Polimeni, Diego Ribuffo, Maurizio Salvati, Antonio Santoro, Paolo Sapienza, Luigi Simonelli, Valentino Valentini, Martina Zambon, Giuseppa Zancana, Emma Zuppi *(Policlinico Umberto I, Rome)*; Simone D’Annunzio, Cosimo De Nunzio, Silvia Fiorelli, Mohsen Ibrahim, Chiara Loffredo, Domenico Massullo, Cecilia Menna, Rocco Monica, Massimiliano Pelli, Erino Angelo Rendina, Leonardo Teodonio, Andrea Tubaro *(Sant’Andrea Hospital, Sapienza University of Rome, Rome)*; Giulio Argenio *(AOU Ruggi, Salerno)*; Giulio Accarino, Accarino Giancarlo, Antonio Nicola Giordano *(San Giovanni di Dio e Ruggi d’Aragona, Salerno)*; Luca Cardinali, Elisa Sebastiani, Grazia Travaglini *(Madonna del Soccorso Hospital, San Benedetto del Tronto)*; Erika Andreatta, Emanuele Luigi Giuseppe Asti, Daniele Bernardi, Luigi Bonavina, Caterina Froiio, Andrea Lovece *(IRCCS Policlinico San Donato, San Donato)*; Chiara Copelli, Alfonso Manfuso *(IRCCS Casa Sollievo della Sofferenza, San Giovanni Rotondo)*; Pasquale Di Maio, Marco Giudice, Oreste Iocca *(Civic Hospital of Sanremo, Sanremo)*; Rosario Cennamo, Tommaso Cornali, Francesco Di Marzo *(Valtiberina, Sansepolcro)*; Cristian Altana, Francesco Bussu, Giampiero Capobianco, Anna Giacomina Carta, Sandro Ciccarello, Maria Laura Cossu, Pietrina Cottu, Giacomo De Riu, Salvatore Dessole, Francesco Dessole, Salvatora Dettori, Carlo Doria, Alessandro Fancellu, Claudio F Feo, Giorgio Carlo Ginesu, Giuliana Giuliani, Marco Giuseppe Iannuccelli, Massimo Madonia, Roberto Mancino, Olindo Massarelli, Gianfranco Meloni, Fabio Milia, Andrea Mulliri, Teresa Perra, Marco Petrillo, Antonio Piras, Franco Piredda, Francesco Pisanu, Alberto Porcu, Davide Rizzo, Angelino Sanna, Antonio Mario Scanu, Fabrizio Scognamillo, Damiano Soma, Anna Rita Tanca, Alessandro Tedde, Matteo Tedde, Luigi Angelo Vaira *(Cliniche San Pietro, A.O.U. Sassari, Sassari)*; Andrea Bartalini Cinughi de Pazzi, Osvaldo Carpineto Samorani, Daniele Fusario, Luigi Marano, Fabio Marino, Gaia Oldrà, Anna Lisa Pesce, Stefania Angela Piccioni, Luca Resca, Vincenzo Ricchiuti, Franco Roviello, Vinno Savelli *(Azienda Ospedaliero Universitaria Senese, Siena)*; Alberto Abrate, Pierpaolo Bordoni, Lorenzo Bosio, Guglielmo Clarizia, Francesco Fleres, Marco Franzini, Antonio Fratto, Pierluigi Giumelli, Alessandro Grechi, Alessandro Longhini, Fabrizio Lorusso, Elisa Scarnecchia, Federica Scolari, Alessandro Spolini *(Ospedale di Sondrio (ASST Valtellina e Alto Lario), Sondrio)*; Vincenzo Maiuri, Matteo Papandrea, Arturo Roncone *(Soverato Civil Hospital, Soverato)*; Lorenzo Conti, Andrea Rizzi, Marco Rovagnati *(Galmarini Hospital, Tradate, Lombardy)*; Alberto Brolese, Tommaso Cai, Francesco Antonio Ciarleglio, Francesca Dalprà, Gianni Malossini, Liliana Mereu, Irene Tamanini, Saverio Tateo, Giovanni Viel *(Santa Chiara Hospital, Trento)*; Enrico Battistella, Paolo Boscolo Rizzo, Cristoforo Fabbris, Marco Massani, Giacomo Spinato, Roberta Tutino *(Ca’ Foncello, Treviso)*; Ugo Grossi, Alessandro Iacomino, Simone Novello, Maurizio Romano, Serena Rossi, Giulio Santoro, Giacomo Zanus *(Ospedale Ca’ Foncello - Università di Padova (DISCOG), Treviso)*; Sokol Trungu *(Cardinale G Panico Hospital, Tricase)*; Giada Aizza, Gabriele Bellio, Selene Bogoni, Marina Bortul, Biagio Casagranda, Sara Cortinovis, Nicolò de Manzini, Davide Drigo, Paola Germani, Manuela Mastronardi, Lucia Paiano, Silvia Palmisano *(Cattinara University Hospital, Trieste)*; Pier Luigi Filosso, Francesco Guerrera, Matteo Marro, Mauro Rinaldi, Enrico Ruffini, Stefano Salizzoni *(Città della Salute e della Scienza, Turin)*; Laura Bardelli, Mattia Berselli, Giacomo Borroni, Eugenio Cocozza, Matteo Desio, Salomone Di Saverio, Bottazzoli Elisa, Giuseppe Ietto, Valentina Iori, Domenico Iovino, Lorenzo Livraghi, Valentina Marchionini, Stefano Megna, Emma Amal Nahal, Mara Palumbo, Valeria Quintodei, Alessandra Zullo *(University of Insubria, Ospedale di Circolo e Fondazione Macchi (Varese), Varese Lombardy)*; Lucrezia D’Alimonte, Giovanni Pirozzolo, Chiara Vignotto *(Dell’Angelo Hospital, Venezia)*; Tommaso Campagnaro, Andrea Caravati, Simone Conci, Carlotta De Cristofaro, Gabriele Gecchele, Tommaso Giuliani, Jacopo Graziosi, Alfredo Guglielmi, Salvatore Paiella, Corrado Pedrazzani, Tommaso Pollini, Simone Rattizzato, Andrea Ruzzenente, Roberto Salvia, Giulia Turri *(Azienda Ospedaliera Universitaria Integrata di Verona, Verona)*; Matilde Bacchion, Giovanni Butturini, Andrea Casaril, Alessandro Giardino, Harmony Impellizzeri, Marco Inama, Frigerio Isabella, Gianluigi Moretto *(Ospedale Pederzoli, Verona)*; Marco De Zuanni, Enrica Deiana, Mario Guglielmo *(Istituto Oncologico del Mediterraneo, Viagrande Catania)*; Alessandro Broglia, Claudia Casarini, Caterina Costanza Zingaretti *(Ospedale Civile di Voghera, Voghera)*; Marta Bonaldi, Giovanni Cesana, Francesco Mastriale, Stefano Olmi, Matteo Uccelli *(San Marco Hospital GSD, Zingonia (BG))*. |
| **Japan**: Yasuyuki Fukami, Takuya Saito, Tsuyoshi Sano *(Aichi Medical University, Aichi)*; Naoki Hirai, Kazuyoshi Hirota, Tetsuya Kushikata, Tasuku Oyama, Junichi Saito *(Hirosaki University Hospital, Aomori)*; Katsuhiko Ishibashi, Mizue Kamiyama, Kyongsuk Son, Kentaroh Tarao, Takayuki Yamada *(Chiba University Hospital, Chiba)*; Toshiya Shiga *(International University of Health and Welfare Ichilawa Hospital, Chiba)*; Chisaki Aze, Yoshihiko Deguchi, Hirotoshi Hasegawa, Tatsuki Hoshino, Yasushi Innami, Hiroyuki Inoue, Shingo Ito, Takeshi Nomura, Tomomi Ogihara, Reina Okada, Takashi Ouchi, Yuri Sekiya, Keikoku Tachibana, Emi Takano, Masae Yamamoto *(Tokyo Dental College, Ichikawa General Hospital, Chiba)*; Shuko Matsuda, Yuka Matsuki, Kenji Shigemi *(University of Fukui, Fukui)*; Teruyuki Hiraki, Yui Inoue, Shosaburo Jotaki *(Kurume University Hospital, Fukuoka)*; Tatsuro Abe, Masatoshi Eto, Junichi Inokuchi, Eiji Kashiwagi, Fumio Kinoshita, Satoshi Kobayashi, Ken Lee, Takashi Matsumoto, Keisuke Monji, Hidekazu Naganuma, Masaki Shiota, Ario Takeuchi *(Kyushu University, Fukuoka)*; Shinju Obara, Saori Takatsuki, Saori Tanaka *(Fukushima Medical University Hospital, Fukushima)*; Koji Iida, Kota Kagawa, Shuichiro Neshige *(Hiroshima University Hospital, Hiroshima)*; Tomohiro Chaki, Naoyuki Hirata, Satoshi Kazuma, Motonobu Kimizuka, Sho Kumita, Noriaki Nishihara, Sato Satoshi, Atsushi Sawada, Shunsuke Tachibana, Michiaki Yamakage *(Sapporo Medical University Hospital, Hokkaido)*; Yuta Nakamura, Kozo Sato *(Shonan Kamakura General Hospital, Kanagawa)*; Tomoya Irie, Tomoko Irisawa, Eiki Kanemaru, Yuko Koga *(Yokohama city university hospital, Kanagawa)*; Yoshihiko Chiba, Jun Makino, Shinnosuke Ohama, Shinichiro Okada, Kano Teruaki *(Yokosuka General Hospital Uwamachi, Kanagawa)*; Tatsuya Kida, Tomohide Takei *(Yokosuka Kyosai Hospital, Kanagawa)*; Kazuhiro Hanazaki, Hiroyuki Kitagawa, Tsutomu Namikawa *(Kochi Medical School Hospital, Kochi)*; Toshiyuki Mizota, Chikashi Takeda, Shintaro Yagi *(Kyoto University Hospital, Kyoto)*; Hiroshi Imai, Makoto Ishitobi, Manabu Kato, Kouhei Nishikawa, Takeshi Sasaki, Hiroshi Yonekura *(Mie University Hospital, Mie)*; Akihiro Kanaya *(Sendai Medical Center, Miyagi)*; Daisuke Irimada, Haruka Ishikawa, Yu Kaiho *(Tohoku University Hospital, Miyagi)*; Mitsuru Ida, Masahiko Kawaguchi, Kenji Kawamura, Munehiro Ogawa, Hiroshi Okada *(Nara Medical University, Nara)*; Shunji Endo, Yoshinori Fujiwara, Masaharu Higashida, Hisako Kubota, Toshimasa Okada, Hironori Tanaka, Tomio Ueno, Kazuhiko Yoshimatsu *(Kawasaki Medical School Hospital, Okayama)*; Motohiro Kikukawa, Akira Kuriyama, Susumu Matsushime *(Kurashiki Central Hospital, Okayama)*; Daisuke Hashimoto, Hishikawa Hidehiko, Haruaki Hino, Yoji Hisamatsu, Akio Kamiya, Hidefumi Kinoshita, Masato Kita, Toshinori Kobayashi, Taku Michiura, Hirokazu Miki, Tomohiro Murakawa, Hidetaka Okada, Tomohito Saito, Ryoichi Saito, Motohiko Sugi, Genichiro Sumi, So Yamaki, Tomohisa Yamamoto, Aya Yoshida *(Kansai Medical University, Osaka)*; Tatsuya Kambara, Sayaka Kanematsu, Okazaki Satoshi *(Kansai Medical University Kori Hospital, Osaka)*; Takeshi Hijikawa, Hiroaki Kitade, Hidesuke Yanagida *(Kansai Medical University Medical Centre, Osaka)*; Tomoyuki Fujita, Satsuki Fukushima, Naoki Tadokoro *(National Cerebral and Cardiovascular Center, Osaka)*; Taku Furukawa, Yusuke Iizuka, Yuji Otsuka, Masamitsu Sanui, Ikumi Sawada *(Jichi Medical University, Saitama)*; Hideki Iwahashi, Morikazu Miyamoto, Masashi Takano *(National Defense Medical College, Saitama)*; Tsutomu Mieda *(Saitama Medical University Hospital, Saitama)*; Chihiro Ando, Tetsuro Isada, Taku Ishizaki *(Todachuo Medical Hospital, Saitama)*; Qaed Bani Amer, Yuki Fujimoto, Sachi Ishida, Yasuma Kobayashi, Norifumi Kuratani, Tomoe Sakurai, Misa Takada *(Saitama Children’s Medical Center, Saitama City)*; Yutaka Iba, Junji Tsukagoshi, Akira Yamada *(Teine Keijinkai Hospital, Sapporo)*; Yuki Amano, Kentaro Fumoto, Shusaku Noro *(Nakamura Memorial Hospital, Sapporo City, Hokkaido)*; Ayataka Fujimoto, Naoto Kuroda, Kyoichi Tomoto *(Seirei Hamamatsu General Hospital, Shizuoka)*; Yukiyasu Okamura, Teiichi Sugiura, Katshuhiko Uesaka *(Shizuoka Cancer Center, Shizuoka)*; Natsuki Takemura *(International University of Health and Welfare, Mita Hospital, Tokyo)*; Kento Kuroda *(Jikei University Daisan Hospital, Tokyo)*; Megumi Hayashi, Satoi Kaneko, Izumi Kawagoe, Tsukasa Kochiyama, Ai Yamaguchi *(Juntendo University Hospital, Tokyo)*; Nobutsugu Abe, Tadao Ando, Mieko Chinzei, Kouichi Hirano, Yoichi Kobayashi, Ryota Matsuki, Hironori Matsumoto, Akira Motoyasu, Harumasa Nakazawa, Hikari Noguchi, Kaio Okamura, Motoaki Ono, Hiroyuki Seki, Eiji Sunami, Atsushi Tajima, Shinji Tanigaki, Kohji Uzawa, Hidenobu Watanabe *(Kyorin University Hospital, Tokyo)*; Yukari Furuhata, Satoshi Toyama, Tokujiro Uchida *(Medical Hospital of Tokyo Medical and Dental University, Tokyo)*; Yutaka Enomoto, Yuri Furukawa, Yoko Hasumi, Jinso Hirota, Katsuyuki Iida, Shingo Ikeda, Tatsuhiko Ikeda, Natsuko Kawamata, Yosuke Kawasaki, Minako Koizumi, Yoshiharu Kono, Shigeki Kuzuhara, Junichi Maeda, Mai Moriyama, Shoichi Nagamoto, Rinako Nakanishi, Michio Noda, Atsushi Seichi, Shintaro Takahashi, Kobayashi Takashi, Misuzu Takeda, Kenta Tanakura, Katsuyuki Terajima, Munechika Tsuji, Chiharu Ueshima, Naoya Yamamoto, Mari Yamamoto, Toshiya Yokota, Yuki Yoshioka, Sakoh Yuri *(Mitsui Memorial Hospital, Tokyo)*; Shintaro Iwata, Akira Kawai, Shuhei Osaki *(National Cancer Center Hospital, Tokyo)*; Masashi Ishikawa, Masae Iwasaki, Tomonori Morita *(Nippon Medical School Hospital, Tokyo)*; Reina Hirooka, Yoshinori Nakata, Shigehito Sawamura *(Teikyo University Hospital, Tokyo)*; Yusuke Ishida, Aya Kawachi, Takayuki Kobayashi, Fumiaki Nagashima, Naoki Suzuki *(Tokyo Medical University, Tokyo)*; Kohei Ando, Noriko Miyazawa, Yukio Tanaka *(Kugawa Hospsital for Orthopedic Surgery, Yamanashi)*. |
| **Jordan**: Khatab Abuissa, Alaa Aljabali, Amal Almasri, Majdi Ali Alqudah, Mera Alrabadi, Rana Alshara, Yasmeen Alzghoul, Ahmed Alzughoul, Ali Freihat, Emran Jeitan, Qais Owais, Aseel Smadi *(Al Iman Hospital, Ajloun)*; Mahmoud Abukhadra, Huthifa Abunawas, Abdallah Ahmed Mezel Al-Azzam, Ammar Aladaileh, Amer Alajalen, Ismail Albadawi, Mohammad Alhawatmeh, Mohannad Yaser Alkhaza’leh, Roa’a Salem Jwai’d Aln’eimat, Saba Alwahedy, Abdel Rahman Mohannad Ahmad Alwardat, Slsabela Dhoon, Khaled El-Qawaqzeh, Tarteel Hrerat, Enas Jaradat, Teeba Mubaydeen, Husam Sarayrah, Mohammad Abdel Elah A. Tarawneh, Mohammad Yousef Karasneh, Amany Zurgan *(Al Karak Hospital, Alkarak)*; Mohammad Alassaf, Sayel H. Alzraikat, Justin Z. Amarin, Ahmed Hijjawi, Rand Y. Omari, Haya H. Suradi, Louay Y. Zaghlol *(Abdali Hospital, Amman)*; Ahmad Abdalah, Ghaida Abdallah, Ammar Abu Tarieh, Tareq Abu-libdeh, Faisal Abualteen, Murad Al Abdallah, Zakaria Al Bdour, Seif Al Dahabrh, Farah Al-kasaji, Zaid Al-sheikh ali, Mohammad Al-thaher, Tareq Alnajjar, Mohannad Alqedrh, Mohammad Alsaleh, Mohammad AlZaatreh, Ghandi Amayreh, Faris Ayasra, Yazeed Ayasra, Ahmad Eid, Ibrahim Ghayada, Khaled Moh’d Ahmed Hasanein, Anas Hassouneh, Raid Hijazeen, Majedah Hmeidan, Ahmed Khaled, Barihan Khasawneh, Omar Kifayeh, Yasser Lafi, Abdulrahman Qasem, Jasem Seba, Qusai Semrin, Fda Sma, Mohammad Theab, Haitham Tumeh *(Al-Basheer Hospital, Amman)*; Muna Aba Zaid, Alaa Abazeed, Bader Abbad, Osaid Abbadi, Nizar Abu-Ishkerih, Malk Al-Osta, Reham Al-Zyadat, Yazan Alawneh, Bashar Omar Falah Alawneh, Majd Alhattab, Moh’d Mujahed Alkurdieh, Ziad Awwad, Mohammad Hammouri, Hamzeh Hussein, Sereen Khader, Mariam Khalil, Omar Mansour, Ahmad Othman, Haitham Qandeel, Mohammad Saleem, Fouad Souri, Ahmad Tahboub, Raed Tayyem *(Ibn Al Haitham Hospital, Amman)*; Munir Abdallah, Ibrahim Abdel-Hafez, Hamza Abu Obead, Hadeel Abudari, Ehab Abuhamour, Samer Abusadah, Amer Ahmad, Nimer Al-azzeh, Sofian Al-Adwan, Ali Al-Darabah, Mohammad Al-Qannas, Omar Aladawi, Basil Albaba, Qutaiba Alradawneh, Bourhan Alrayes, Mohammad Amaireh, Subhi Ashour, Reem Chabaan, Ammar Eskander, Ahmad Fawzi, Hanan Haidar, Manal Haij, Hisham Hamad, Haya Hamdan, Ahmed Hamss, Esraa Hassouneh, Shatha Husain, Ayah Hyasat, Zainab Ibrahim, Saja Jamaliah, Mohammed Khalil, Anas Massad, Ishak Mohamed, Mahmoud Qandeel, Dalia Qasrawi, Faisal Qassem, Mahmoud Raggad, Ebaa Rifai, Nedal Semreen, Reham Shehada, Marwan Shorman *(Islamic Hospital, Amman)*; Abdallah Al-Shibi, Shatha AlSA’AFIN, Morad Bani-hani, Bahaa Nimer, Sandra Nofal, Layth Qaraqe, Mohammed Salameh, Ma’moun Saleh, Ghaidaa Sanjuq, Alaa Sharabi, Adnan Sumadi, Alaa Sweiti, Jamal Yaghmour, Sabri Zaza *(Istiklal Hospital, Amman)*; Rahaf Alhindi, Eman Baninasr *(Jordan University Hospital, Amman)*; Nizar Abu-Ishkerih, Amro Abuleil *(Jordanian royal medical services, Amman)*; Mohamad K. Abou Chaar, Mahmoud Al-Masri, Hani Al-Najjar, Fade Alawneh *(King Hussein Cancer Center, Amman)*; Motasem Al-latayfeh, Abdel-Ellah Al-Shudifat, Abd Almonem Shaikh Ahmad *(Prince Hamza hospital, Amman)*; Abdelkarim Abdeljalil, Haneen Abdelrahman, Abdulmalek Abdulmalek, Lina Abedalqader, Laith Abu Abed, Amro Abuleil, Omar Al Smadi, Salameh Alarood, Mohammad Allouzi, Maha Almansour, Sari Almi’ani, Abdulhakim Alsaiad, Khaled Alshaikh, Farah Androus, Mirna Awbakh, Belal Azzam, Hammam Bany yasin, Maher Daoud, Lujain Dawod, Omar Dawod, Reem Elmusa, Saba Fadli, Farah Hammad, Majd Hunaiti, Elmi Ahmed Mohamed Jimaale, Mahmoud Muhtaseb, Rawan Owaimer, Zaki Qulaghassi, Saleh Romman, Rawand Saket, Said Sharawi, Alaa Tawalbeh *(The Speciality Hospital, Amman)*; Mohammed Al-howthi, Laila Ababneh, Roba Ababneh, Faris Jamal Abu Za’nouneh, Marah Abu-Mehsen, Sajedah Al DHOUN, Ahmad Al Khassawneh, Ahmed Al Sharie, Ayah Ahmad Al_shraideh, Tawfik Al-Dabaa, Salsabeel Al-Jarrah, Hothaifa Al-Jarrah, Hadeel Al-Othman, Zuhair Alaradi, Qutaiba Alshannaq, Rima Alsulaiman, Motasem Alzaqh, Yazan Alzu’bi, Ziad Audat, Mohammad Bahhour, Saja Bdour, Ro’a Khatatbeh, Fares Marji, Tagleb Mazahreh, Dina Nail, Amjad Nuseir, Asmaa Rabab’ah, Taher Sawadi, Omar Wafi, Ghina Zidan *(King Abdullah University Hospital/ Jordan University of Science and Technology, Ar Ramtha)*; Dima Abu muhfouz, Abdullah Nael, Ahmad Qasim *(Zarqa Hospital, AZ zarqa)*; Hazim Ababneh, Amar Al-Jarrah, Faisal Rawagah *(Ar Ramtha Govermental Hospital, Irbid)*; Dima.Y Abu Ismail, Luai Abu-Ismail, Wedad Al-dolat, Mai Alzoubi, Malak Alzoubi, Mu’taz Alzu’bi, Ruba Asha, Ali Guboug, Hasan Hussein, Almu’atasim Khamees, Shireen Rawashdeh, Mohammad Sanwar *(Irbid Specialty Hospital, Irbid)*; Adnan Ababneh, Basil Abu-Eisheh, Haneen Ahmad Alhami, Saif Aldeen Al Dwairi, Hana’ Al Shurman, Zakaria Al Yahya, Hussam Al-atiyah, Hasan Al-Balas, Anas Aljaiuossi, Ahmad Alkhatib, Shatha Alqawasmi, Sadeel Alqudah, Mahmoud Alshourman, Abdulsalam Bani Hamad, Anas Bany Issa, Raed Ennab, Sara Hamdoni, Zaid Hijazi, Ali Khafaja, Mohamed Mraiyan, Riyad Obeidat, Dina Olaywah, Mohamad Qudah, Mhamdd Talal *(Princess Basma Hospital, Irbid)*. |
| **Kazakhstan**: Askar Aidarov, Raikhan Bolatbekova, Aisulu Sarmenova *(Almaty Oncology Centre, Almaty)*; Almat Kodasbaev, Mukhtar Kulimbet, Orazbek Sakhov *(City Cardiology Center, Almaty)*; Ildar Fakhradiyev, Talgat Nurgozhin, Timur Saliev, Shynar Tanabayeva, Baurzhan Zhussupov *(City Clinical Hospital No. 4, Almaty)*; Ydyrys Almabayev, Sultan Amrayev, Berik Dzhumabekov, Maulen Malgazhdarov, Kanat Tezekbaev *(Karasay Central District Hospital, Almaty)*; Dilyara Kaidarova, Yerlan Kukubassov, Alima Satanova *(Kazakh Institute of Oncology and Radiology, Almaty)*. |
| **Kenya**: Michael Mwachiro, Robert Parker, Ian Simel *(Tenwek Hospital, Bomet)*; Shamshuddin Mohammedali, Claude Mwaria, Ralph Obure, Sayed Shah Nur Hussein Shah *(Aga Khan University Hospital Nairobi, Nairobi)*; Chetan Hirani, Muthoni Kibunyi, Claire Mailu, Victor Mwangi, Roy Ogenya, Lydia Osea *(Kenyatta National Hospital, Nairobi)*; Davies Cheruiyot, Omar Hirsi, Carolyne Muiru, Samuel Wanjara *(Nakuru Level V Hospital, Nakuru)*. |
| **Korea, Rep.**: Chang Woo Kim, Suk Hwan Lee *(Kyung Hee University Hospital at Gangdong, Seoul)*. |
| **Kuwait**: Ahmad Almulla, Mustafa Dashti, Omar Elghany, Dr abdullah Sultan *(Farwaniya Hospital, Farwaniya)*; Husain Al-Mahmeed, Jasim Alabbad, Ibtisam Albader, Shuaib Aldalal, Mahdi Baba *(Mubarak Al-Kabeer Hospital, Jabriya)*; Fareed Abdulsalam, Naser AlHajri, Athari Alwael, Brook Ayele, Hatem Marie, Ahmed Zaitoun *(Aljahra hospital, Jahra)*; Ahmad M. AlAli, Ahmed ALKhamis, Ous Alozairi *(Al-Adan Hospital, Kuwait)*; Sulaiman Almazeedi, Salman AlSabah *(Jaber Al Ahmad Al Sabah Hospital, Kuwait)*; Mostafa Abdelkarim, Asmaa Al Rashed, Saud Al Subaie, Tariq Al-Shaiji, Abdullatif Al-Terki, Omar Alhunaidi, Ali Aljewaied, Khaled AlZamel, Ahmed Farag, Hussein Hayati, Salah Termos, Sarah Wood *(Al Amiri Hospital, Kuwait City)*; Maha Al-Gilani, Mutlaq Al-Sihan, Haifa Alotaibi *(Zain hospital, Kuwait City)*. |
| **Latvia**: Dmitrijs Lobovs, Andrejs Pcolkins, Armands Sivins *(Latvian Center of Oncology, Riga)*; Ingus Arnolds Apse, Annija Evelīna Berga, Anna Ivanova *(Pauls Stradins Clinical University Hospital, Riga)*; Jevgenijs Demicevs, Igors Ivanovs, Aleksejs Kaminskis, Janis Opincans, Jānis Pāvulāns, Agris Rudzāts, Anna Udre *(Riga East CUH Gailezers Hospital, Riga)*. |
| **Lebanon**: Samer Dbouk, Imadeddine Farfour, Mohamad Rakka *(Al Zahraa Hospital University Medical Center, Beirut)*; Ingrid Antonios, Joe El Hage, Stephanie Hage, Lea Haiby, Hiba Kahi, Anthony Lichaa, Rita Milan Moussa, Michel Salameh, Riad Sarkis *(Hotel Dieu De France, Beirut)*; Obey Albaini, Tony Haykal, Bassem Safadi *(LAU Medical Center-Rizk Hospital, Beirut)*; Hussein Abou-Abbass, Reem Al Makari, Zeina Al Zein, Mariam Baidoun, Rania Itani, Mustafa Owiedat *(Makassed General Hospital, Beirut)*; Ali H. Abdel Sater, Michel Akl, Nour Badran, Sara El Mustapha, Fathallah Fatouh, Youssef Ghoussoub, Nader Saad, Fatima Serhan, Bassem Souleiman *(Rafik Hariri University Hospital, Beirut)*; Walid Alame, Peter Ghiya, Firas Ibrahim *(Sahel General Hospital, Beirut)*; Rasha Abdallah, Mohamad B. Kassab, Lina Karout, Samar Karout *(Rayak Hospital, Rayak)*; Aya Abdul Al, Hilal Chaaban, Ghinwa Fakih, Huzifa Haj-Ibrahim *(Saida Govenmental Hospital, Saida)*; Rabih Awad, Hadi El Assaad *(Nini Hospital, Tripoli)*. |
| **Libya**: Khalil Tamoos *(Alabyar General Hospital, Alabyar)*; Ahmed Aqeelah *(Althawra Hospital Albyda, Albayda)*; Ahmed Alfeqeeh, Alsnosy abdullah Khalefa mohammed, Hosam Muftah *(Almarj Teaching Hospital, Almarj)*; Taha Abdulrahman, Naeimah Ahseen, Hayat Omar Abunaaja *(Bani-walid general hospital, Bani_walid)*; Abdulmuez Abdulmalik, Abd El Jawad Al Gasi, Faraj Al maadany, Ghadah Alkadeeki, Sara Alsaeiti, Fatimah Elkhafeefi, Nasre Elrefai *(Al-jalaa Teaching/Trauma Hospital, Benghazi)*; Rofida Ahmad, Wafa Aldressi, Tarek Alhouni, Safya Alkarky, Hana Alkeelani, Rauoof Alkuwafi, Rema Benhariz, Nagat Bettamer, Musa Busarira, Hassan Bushaala, Tariq Diryaq, Osama Elbargathe, Tarik Eldarat, Mustafa Elfadli, Jamael Elfitori, Asma Elmahgoub, Reem Elsahti, Milad Gahwagi, Yosef Hassan, Fathi Hussain, Taha Mirsal, Marwa Mohammed, Ebtesam Saad *(Benghazi Medical Center, Benghazi)*; Wafa Aldressi, Rauoof Alkuwafi, Tarik Eldarat *(N. B. Hospital, Benghazi)*; Mohamed Amnaina, Haifaa Il hadad, Mostafa Mosabha *(Al-Wahda Hospital, Darna)*; Arowa hassan abdulrahman Alansari, Awatif Alhaje, Aaya Haron *(Ghadames general hospital, Ghadames)*; Akram Alkaseek *(Gharyan Central Hospital, Gharyan)*; Suhir Alsuwiyah, Mona Hamed *(Jadu General Hospital, Jadu)*; Ghozlan Yagoub *(Alhikma hospital, Misurata)*; Samer Khel, Sana Omar, Rowaida Yousef *(Misurata Cancer Center, Misurata)*; Abubaker Abdelmalik, Yahya Abosnaina, Ma’aly Abuhlaiga, Ahmed omar Abushahma, Alhosen Saleh M Aldelensi Alzubi, Asma Aljanfi, Mohamed alnaser Alnehum, Ahmad Alzedam, Anass Ben Amer, Malik Delhen, Galia Maderi, Faisel Matoug, Burooj Mohammed, Marwa Salem, Mohamed Sawalem *(Misurata Central Hospital, Misurata)*; Alhadi Jahan, Abdussalam Jahan *(Zahrawi Hospital, Misurata)*; Basma Alazabi, BahaUldin Alezabi, Ayman Almugaddami, Mona masaud Amro Alazabi, Amin Salem Ahmed Egdeer, Fatma Sefi *(Nalut Central Hospital, Nalut)*; Dania Burgan, Elhusain Kamoka, Abdulrauf Salamah *(National Cancer Institute, Sabratha - Libya, Sabratha)*; Alya Abdalhadi, Saedah Abdeewi, Mohammed Abdelkabir, Marwah AlHADAD, Ahmed Awrayit, Mabroka Eshnaf, Mohammad Yahmad *(Sabha Medical Center, Sebha)*; Abdulhafid Abudher, Khayriyah Alshareef, Faisl Elamin *(Yashfeen Clinic, Tajora-Tripoli)*; Farah Abojeila, Mohamed Aliwa, Rayet al islam Ben jouira, Sana Shagour *(National Heart Centre, Tajoura Heart Center Hospital, Tajoura)*; Ahmed Altobal, Ayman Meelad *(Tarhuna General Hospital, Tarhuna)*; Omar Aljuroushi, Hadeel Almadani, Hajer Attia, Ahmad Bouhuwaish, Amal Sharif Eljali, Ayyah Emran, Mabruka Omar, Radhwan Salim, Ashraf Samer *(Tobruk Medical Center, Tobruk)*; Nayrouz abdullah Abulshuwashi, Emadeddin T M Ben Khalifa, Hdaya Abdalla S Benabdalla, Hatem Elnageh, Ali Hammad *(Abu Saleem Trauma Hospital, Tripoli)*; Duha milad Abdullah, Marwa Abusalem, Esraa Ben esmael, Fatma Benmasoud, Laila Debri, Sumayya Essayah Dwaga, Randa Elmokhtar, Manal Ezeddin kamel Sheta, Houda Khalifa, Bushra Sayeh Mohamed Elhabashi, Balkees Younis *(Aljala Maternity Hospital, Tripoli)*; Bushray Almiqlash, Amna Elmabrouk, Ahmed Momen *(Alkhadra Hospital, Tripoli)*; Abdalrahem Essied Alzubi Alzoubi, Kusay Ayad *(Alkhalil hospital, Tripoli)*; Ameerah Mahdi Abraheem Hasan, Fawzie Musrati, Taha Suliaman *(Crown of Health Clinic, Tripoli)*; Malek Abusannoga, Mohammed Alawami, Ans Malek *(Medical Care Clinic, Tripoli)*; Hatem Ajaj, Fras Elhajdawe *(Metiga Hospital, Tripoli)*; Eman Abdulwahed, Hajir Aboazamazem, Mohammed Aboubeirah, Aya Alqurpaa, Entisar Alshareea, Sarra Aribi, Tomather Aribi, Marwa Biala, Reem Ghamgh, Mohannned Issa, Amani hamid Lamari, Mohammed Mufth *(Tripoli Central Hospital, Tripoli)*; Omar Sudig Abboud, Muad fathi khalleefah Abu hallalah, Saleh Abumahara, Sarah Aburima, Mohammed Adrees, Abdurahman Ahtash, Asmaa Al shukri, Hind Alameen, Rehab Alarabi, Alarabi Alarabi, Aisha Alelwany, Abdulkarim Alesmail, Noran Algadi, Khuloud Almaqrahi, Hadel Alosta, Mohamed Alsharedi, Rasha Bashir, Mahmoud Ben ghrema, Sari Bin nour, Sofian Elbarouni, Ahmed Elhadi, Abdulmohimen A Elkhadar, Esraa Elkouba, Ibrahim Ellojli, Nabiha Elmsherghi, Abdulmohimen Gammoudi, ABDUlRAOUF Ghariba, Fatima Zayed Gjam, Arwa Haidar, Hala Helal, Mohammed Huwaysh, Ahmed Khalid alhadheeri, Eslam Kriem, Ahmed Msherghi, Halim Osman, Mohamed Ali Ossman, Marwah Shilfeet, Laila Turshani *(Tripoli Medical Center/ Tripoli University Hospital, Tripoli)*; Nouran Albishty, Abdulmueti Alhadi, Salem Ali, Manal Ahmed Altoumi, Sumayyah Ghayth Bahroun, Eman Younes *(Zawia Teaching Hospital, Zawia)*; Hana Atarabulsi *(Taqwa Clinic, Zintan)*; Mohamed Elshaibi, Abdullmujeeb Othman, Ismail Ali Saleh *(Zintan Teaching Hospital, Zintan)*; Mohammed Alayan, Hayat Ben Hasan, Najat Shaban Ben Hasan, Rabab Shaban Ben Hasan *(Zliten Teaching Hospital, Zliten)*. |
| **Lithuania**: Vidmantas Barauskas, Edvinas Dainius, Zilvinas Dambrauskas, Albertas Dauksa, Inga Dekeryte, Rita Gudaityte, Kristijonas Jasaitis, Akvilė Koženiauskaitė, Virgilijus Krasauskas, Ausra Lukosiute-Urboniene, Almantas Maleckas, Romualdas Riauka, Vygintas Šlenfuktas, Saulius Svagzdys, Kestutis Urbonas, Tomas Vanagas, Linas Venclauskas, Donatas Venskutonis, Justas Zilinskas *(Lithuanian University of Health Sciences Kaunas Clinics, Kaunas)*; Marijus Ambrazevicius, Vilius Syminas *(Klaipeda Republic Hospital, Klaipeda)*; Oleg Aliosin, Agne Cizauskaite, Vitalijus Eismontas, Jonas Jurgaitis, Vytenis Mikutaitis, Donatas Petrauskas, Narimantas Evaldas Samalavicius, Dainius Simcikas, Algirdas Slepavicius, Albinas Tamosiunas, Vaidotas Turskis, Bernardas Vasiliauskas *(Klaipeda University Hospital, Klaipeda)*; Laura Aniukstyte, Audrius Dulskas, Gediminas Januška, Justas Kuliavas, Margarita Montrimaite *(National Cancer Institute, Vilnius)*; Albertas Cekauskas, Aiste Gulla, Azuolas Algimantas Kaminskas, Sarunas Kozenevskis, Tomas Poskus, Kestutis Strupas, Arunas Zelvys *(Vilnius University Hospital, Vilnius)*. |
| **Luxembourg**: Georges Decker, Audrey Noël *(Hopitaux Robert Schuman, Luxembourg)*. |
| **Madagascar**: Maheriandrianina Fanambinana Voahary Rajaonarivony, Randimbinirina Zakarimanana Lucas *(Centre Hospitalier de Soavinandriana, Antananarivo)*; Herimampionona E Andriantsoa, Mamisoa B Rasamoelina *(CHU Anosiala, Antananarivo)*; Rakotonarivo Aina Andrianina Vatosoa, Liantsoa Andriamanana, Casimir FP Rahantasoa Finaritra, Aristide Romain Raherison, Tsirimalala Rajaobelison, Haritiana Rakotoarisoa, Maharo Ramifehiarivo, Solonirina Harinarindra Ranaivoson, Hajamihamina Marina Parfaite Randriantsoa, Fanjandrainy Rasoaherinomenjanahary, Narindra Njarasoa Mihaja Razafimanjato, Jeannie BA Razafindrahita, Luc Hervé Samison, Solofoarimanana Solofoarimanana, Guillaume Odilon Tsiambanizafy *(Joseph Ravoahangy Andrianavalona Hospital, Antananarivo)*; Rakotonaivo Mamisoa Judicaël, Corinne Eulalie Solo *(CHU Tanambao I, Antsiranana)*; Tojomamy Herinjaka Ralaizafindraibe, Fanonjomahasoa Safiry Andofenohasina *(CHU Pzaga Androva, Mahajanga)*; Hanta Rasataharifetra, Vanona barijaona Razafindraibe, Angelin Sablon Herinirina *(CHU Analankininina, Toamasina (Tamatave))*. |
| **Malaysia**: Andre Das, Thanga Ganapathy, Yohesuwary Gunarasa, Kalyani Mariapan, Maathichsudhaar Muniandy, Mohd Firdauss Osman *(Hospital Kajang, Kajang, Selangor)*; Mohd Azem Fathi Mohammad Azmi, Jien Yen Soh, Wan zainira Wan zain, Andee Dzulkarnaen Zakaria, Zaidi Zakaria *(Hospital Universiti Sains Malaysia, Kelantan)*; Kheng Hooi Chan, Firdaus Hayati, Syamim Johan, Jin Jiun Mah, Ratha Krishnan Sriram, Sentilnathan Subramaniam *(Queen Elizabeth Hospital, Kota Kinabalu)*; Nur Shahirah Binti Muhammad Shahimi, Khairul Hazim Hamdan, Mohd Razali Ibrahim, Guhan Muthkumaran, Ju Ann Tan, Mohana Raj Thanapal *(Hospital Kuala Lumpur, Kuala Lumpur)*; Dayang Anita Abdul Aziz, Mae-Lynn Bastion, Mohd Hairul Nizam Harun, Farrah-Hani Imran, Norshamsiah Md Din, Ayesha Mohd Zain, Mushawiahti Mustapha, Syed Nabil, Ainal Adlin Naffi, Abd Jabar Nazimi, Roszalina Ramli, Bee Hong Soon *(University Kebangsaan Malaysia Medical Centre, Kuala Lumpur)*; Pavin Bal, Joo Qing Cheng, Mohd Rusdi Draman, Ahmad nazran Fadzli, Sakina Ghauth, WeiPin Hung, Suniza Jamaris, Peng Soon Koh, Sivakumar Krishnasamy, Yeong Sing Lee, Yew Toong Liew, Jeffery Zk Lim, Raymond Chung Siang Lim, Hiong Chin Lim, Siti Farhan Moh Pauzi, Shireen Anne Nah, Ashvin Krishna Nair, Doris Sin Wen Ng, Yuki Julius Ng We Yong, Kamarajan Ramayah, April Camilla Roslani, Rahmah Saaid, Anand Sanmugam, Mee Hoong See, Kanesh Kumaran Seevalingam, Neha Sethi, Srihari Singaravel, Syeda Nureena Syed Jafer Hussain Zaidi, Chu Yik Tang, Li Ying Teoh, Kian Boon Wong, Ruben Xavier, Ahmad Shuib Yahaya *(University Malaya Medical Centre, Kuala Lumpur)*; Norhafiza Ab. Rahman, Islah Munjih Ab. Rashid, Mohd Fahmi Abd Aziz, Mohd Norhisham Azmi Abdul Rahman, Nasser Muhammad Amjad, Shahidah Che Alhadi, Faisal Elagili, Mohd Nazli Kamarulzaman, Azmi Md Nor, Ahmad Faidzal Othman, Mohd Yusof Sainal, Mat Salleh Sarif *(International Islamic University Malaysia Medical Centre, Kuantan Pahang)*; Richelle Chua, Aini Fahriza Ibrahim *(Sarawak General Hospital, Kuching, Sarawak)*; Abdul Rauf bin Ahmad, Yii Ling Lau, Ka Yee Liew, Kandasami Palayan, Hui Yu Wong, Jasiah Zakaria *(Hospital Tuanku Ja’afar, Seremban, Negeri Sembilan)*; Nor Azimah Abd Aziz, Norazila Abdul Rahim, Noorneza Abdul Rahman, Bahiyah Abdullah, Mohamed Faizal Bin Sikkandar, Fatin Firman, Nik Mohd Hazleigh, Intan Kartika Kamarudin, Shahril Khalid, Mohd Fairudz Mohd Miswan, Akmal Zulayla Mohd Zahid, Norasyikin Mustafa, Kadhim Obaid, Mohd Yusoff bin Yahaya Yahaya, Ahmad Ramzi Yusoff *(Faculty of Medicine, Universiti Teknologi MARA, Sg. Buloh, Selangor)*. |
| **Malta**: Predrag Andrejevic, Jeremy Borg Myatt, Svetlana Brincat, Clifford Caruana, Kurt Lee Chircop, Francesca Chircop, Daniel Mario Chircop, Charles Cini, Viktoria Czok, Melanie Farrugia, Gabriella Grima, Fatimah MJ Hassan Almukhariq, Esther Muscat, Darryl Pisani, Mohsin Hassan Khan Roshan, Nicholas Schembri, Mohamed Shoukry, Neville Spiteri, Gilbert Tanti *(Mater Dei Hospital, Msida)*. |
| **Mexico**: Ziad Aboharp Hasan, Alberto Bazan Soto, Cedillo Rodrìguez Jonathan Rubén, Gustavo Esteban Lugo Zamudio, David Gerardo Miranda Gómez, José Alfredo Pérez Meave, Victor Pinto Angulo, Antonio Rojas Aguilar, Erik Efrain Sosa Duran, Alejandro Vàzquez Perez *(Hospital Juárez de México, Ciudad de México)*; Diego Arroyo, Jose Corona-Cruz, Hector Martinez-Said, Heriberto Medina-Franco, Manuel Mendez, Teresa Moreno y Suárez, Ma. Dolores Orta Díaz, Martha Patricia Pérez de León Vázquez, Angélica Azucena Soto Carvajal, Fidel Soto Hernández, Juan Ignacio Stenner, Adriana Valenzuela López *(Hospital Médica Sur, Ciudad de México)*; Mauricio Rene Hernandez, Rey Jesus Romero *(Hospital Covadonga, Cordoba)*; Aldo Bernal Hernandez, Ana Olivia Cortes-Flores, Carlos Jose Zuloaga Fernandez del Valle *(ANKER Oncologia Global Especializada, Guadalajara)*; Carlos Andres Colunga Tinajero, Jaime Orozco-Perez, Laura Gabriela Peña Balboa, Guillermo Yanowsky-Reyes *(Antiguo Hospital Civil de Guadalajara, Guadalajara)*; Víctor Hugo Alcalá Torres, Francisco José Barbosa Camacho, Irma Valeria Brancaccio Perez, Miguel Angel Calderon-Llamas, Aldo Camacho Gomez, Edgar-Joaquin Cortes-Torres, Esteban Cueva-Martinez, Oscar Durán Anguiano, Isaac Esparza Estrada, Roberto Fierro-Rizo, Paola Flores Becerril, Clotilde Fuentes Orozco, Miguel García Castillo, Alejandro Gonzalez Ojeda, Ana Sofia Gonzalez Rubio, Erick Alonso González-García de Rojas, Luis Humberto Govea-Camacho, Jose Aldo Guzman Barba, Juan Carlos Ibarrola Peña, César Íñiguez Martínez, Francisco Javier Llamas- Macias, Ruben Eduardo Morán Galaviz, Cesar Nuño-Escobar, Oscar-Everardo Olvera-Flores, Jaqueline Osuna-Rubio, Jose Victor Pérez-Navarro, Rodrigo Prieto-Aldape, Luis Ricardo Ramirez Gonzalez, Marco Vinicio Ramírez Sánchez, Emilio Reyes, Olaya Moramay Romero-Limón, Carlos Beas Ruiz-Velasco, José Antonio Sánchez Martínez, Guillermo Sanchez-Villaseñor, Ismael Sedano-Portillo *(Hospital de Especialidades, CMNO-IMSS, Guadalajara)*; Gabriela Ambriz Gonzalez, Pedro Berrones Moreno Berrones Moreno, Ana Beatriz Calderon Alvarado, José de Jesús Cárdenas Barón, Araceli Hernández, Francisco Javier León Frutos *(UMAE Hospital De Pediatria CMNO, Guadalajara Jalisco)*; Edgard Efren Lozada Hernandez, Eduardo Morales valencia, karla Daniela Pérez *(Hospital Regional e Alta Especialidad del Bajio, León)*; Francina Bolanos-Morales, Julio Herrera-Zamora, Patricio Santillan-Doherty *(Instituto Nacional de Enfermedades Respiratorias, Mexico)*; David Omar Arriaga Zavala, Salvador Francisco Campos Campos, Lizette Carmona, Antonio Galindo Nava, Blanca Santos, Juan Roberto Torres Cisneros *(Hospital Angeles Lindavista, Mexico city)*; Katya Bozada-Gutiérrez, Jose Jesus Herrera, Mucio Moreno Portillo, Francisco Pérez López, Mario Trejo-Avila, Carlos Valenzuela Salazar *(Hospital General Dr Manuel Gea González, Mexico city)*; Maria Regina Alvarez, Fernando Cordera, Marco Antonio de la Rosa Abaroa, Antonio Gómez-Pedraza, Roberto Hernandez, Antonio Maffuz-Aziz, José Antonio Posada, Carlos Robles Vidal *(ABC Medical Center, Mexico City)*; Jose-Luis Beristain-Hernandez, Blas Eduardo Quintero Sada, Nicolás Uxmal Soruco Heredia *(Centro Médico Nacional La Raza, Mexico City)*; Ernesto Anaya, Ricardo Arceo-Olaiz, Bernardo Gutiérrez Sougarret, Sonia Lopez Flores, Hector Martinez-Said, Francisco J. Ochoa Carrillo, Raymundo Alfredo Pérez Uribe *(Hospital Angeles Pedregal, Mexico City)*; Ludwigvan Adriano Bustamante Silva, Arianne I Lupián-Angulo, Christian Enrique Soulé Martínez *(Hospital Central Norte Pemex, Mexico City)*; Francisco César Becerra García, Ernesto Javier Guerrero Casillas, Edgar Uribe *(Hospital San Angel Inn Patriotismo, Mexico City)*; Nereida Esparza Arias, J.Mindy Hernández-Nava, David Isla-Ortiz, Javier Melchor-Ruan, Esmeralda Romero Bañuelos, Rosa Salcedo-Hernández, Diana Vilar-Compte, Paola Zinser Peniche *(Instituto Nacional de Cancerologia, Mexico City)*; Jorge L. Aguilar Frasco, Alejandro Alfaro-Goldaracena, Gabriela Alejandra Buerba, Emma Castro, Carlos Chan, Ruben Cortes, Ismael Dominguez-Rosado, Daniel Garay Lechuga, Miguel Herrera, Carlos Hinojosa, Gabriel Lopez-Pena, Edgar Martos, Heriberto Medina-Franco, Miguel Ángel Mercado, Paulina Moctezuma Velázquez, Roberto Moguel, Mariano Oropeza, Emmanuel Peña Gómez Portugal, Oscar Emmanuel Posadas-Trujillo, Noel Salgado-Nesme, Oscar Santes Jasso, Catherine Sarre *(Instituto Nacional de Ciencias Médicas y Nutrición ‘Salvador Zubirán’, Mexico City)*; Karla Yareli Hernandez Skewes, Cristian Jimenez, Roque Delfino Licona-Meníndez, Guillermo Montiel, Alberto Navarrete-Peón, Shannat Ortega, Fernanda Romero Lechuga, Oscar Roque, Fernando Sevilla *(Sociedad Española de Beneficencia, Pachuca)*; Rogelio González-López, Roberto Ángel Núñez-González, José Antonio Ortega-Jiménez *(Hospital de especialidades Puebla CMN Gral. de Div. Manuel Ávila Camacho, Puebla)*; Antonio Alvarado, Alfonso Alvarez Manilla Orendain, José Alberto Atristain Pesquera, Diego Frutos, Felipe Garcia, Juan Antonio González León, Marlin Guzman, Dorihela Herappe, Jesús Giovanni Inzunza Miranda, Miguel Angel Jimenez Botello, Ricardo Lerma, Fabián Hilario Mendoza Pedraza, Monica Noguez Castillo, Jose Anatolio Resendiz, Oscar Alejandro Sánchez García *(Hospital de especialidades del niño y la mujer, Querétaro)*; Laura Elena Fernandez Rios, Pamela Frigerio, Iset Garcia, Gloria Isela Mendoza Frías, Emmanuel Moreno, Raquel Quiroga *(Hospital Universitario ‘Dr. Gonzalo Valdés Valdés’ UAdeC, Saltillo, Coahuila)*; Alejandra Anzures Mendoza, Arantza Govela Hinojosa, Aldo Izaguirre *(Hospital Medica Universidad, Tampico)*; Luis Hdez Miguelena, Jose Manuel Luna Vazquez *(Hospital de Alta Especialidad de Veracruz, Veracruz)*; Indalecio Barcelata Rodriguez, Raul Ramos mange, Antonio Ramos-De la Medina, Carlos Reyes Utrera, David Zarate Saenz, Miguel angel Zavala gonzalez *(Hospital Español Veracruz, Veracruz)*; Francisco Andrade González, Ludivina Cortes, David Roman, Aaron Sanchez *(Hospital Regional de Xalapa Dr Luis F Nachon, Xalapa, Veracruz)*. |
| **Moldova**: Radu Gurghiș, Gheorghe Rojnoveanu, Marin Vozian *(Institute of Emergency Medicine, Chisinau)*; Petru Caraja, Gheorghe Croitor, Roman Mosneaga *(Repromed +, Chisinau)*. |
| **Mongolia**: Sarnai Erdene, Lundeg Ganbold, Sergelen Orgoi, Soyombo Orsoo, Altanchimeg Sainbayar, Erdene Sandag, Burmaa Sanjaa *(Mongolian National University of Medical Sciences, The first central hospital of Mongolia, Ulaanbaatar)*. |
| **Morocco**: Rita Ait benhamou, Abdessamad El Azhari, Bennis Ghita, Sara Lhassani, Sidi Mamoun Louraoui, Mounir Rghioui, Sarra Saaf *(Cheikh Khalifa International University Hospital, Casablanca City)*; Taha Gadouali, Iltimass Gouazar, Moniba Korch, Ahmed Letrache, Amina Louari, Anas Zamame *(Centre Hospitalier Universitaire Mohammed VI, Marrakech, Marrakech)*; Samia Ousouss, Meryem Zekhnini *(Centre Hospitalier Universitaire Oujda Mohammed VI, Oujda)*; Hafni Abderrazaq, Wijden Abichou, Hamza Ahlaou, Amal Baicha, Salma Baroudi, Ghita Chaoui, Hind El Azzazi, Saja El Masaoudi, Chiraz Hassoun, Hajar Moujtahid, Anas Riyahi, Ouijdane Zaim *(Centre Hospitalier Universitaire Ibn Sina Rabat, Rabat)*; Narjiss Aji, Imane Ammouze, Yasser Arkha, Hajar Bechri, Meryem Benchekroun Belabbes, Zaineb Benslimane, Lina Boualila, Othmane Bouanani, Abdessamad El Ouahabi, Leila Essakalli Hossyni, Boulaadas Malik, Mohammed Yassaad Oudrhiri, Taha ismail Sefrioui, Adam Tagmouti *(Hôpital des Spécialités - ONO, Rabat)*; Mustapha Bensghir, Abdelghafour Elkoundi *(Hôpital Militaire Mohammed V, Rabat)*; Othman Belarabi, Zakaria Houssaïn Belkhadir, Amine Benkabbou, Saber Boutayeb, Brahim El Ahmadi, Yassine El Bouazizi, Abdelilah Ghannam, Taha Kabbaj, Anass Mohammed Majbar, Raouf Mohsine, Amine Souadka *(Institut National d’Oncologie, Rabat)*. |
| **Namibia**: Badra Aliou Kone, Marcel Didier Ndayishyigikiye *(Ongwediva Medipark Teaching Hospital, Ongwediva)*; Akutu Munyika, Fillipus Ndatewapo, Peter Ngungi Njuki *(Onandjokwe Intermediate Hospital, Oniipa)*; Philipp Plarre, Srihari Singaravel *(Mediclinic Cottage Hospital, Swakopmund)*; Jenevive Abrahams, David W Borowski, Clara Jeketera, Kwasi Yeboah *(Welwitschia Hospital, Walvis Bay)*; John Tabiri Abebrese, Alfred Mureko, Pueya Rashid Nashidengo, Francis William Quayson, Justina Shikongo *(Windhoek Central Academic Hospital, Windhoek)*. |
| **Nepal**: Subarna Bhusal, Yam Dwa, Ashoo Joshi *(KIST Medical College and Teaching Hospital, Lalitpur)*. |
| **Netherlands**: Eva Berkeveld, Frank Bloemers, Alexander Borgstein, Suzanne Gisbertz, Sarah Mikdad, Mark van Berge Henegouwen *(Amsterdam UMC VUmc, Amsterdam)*; Roel Bakx, Marc Besselink, Daniel HL Lemmers, Anne-Jasmin Roelofs, Paul van Amstel, Claire van Helsdingen *(Amsterdam UMC, University of Amsterdam, Amsterdam)*; Ludi Smeele *(Antoni van Leeuwenhoek ziekenhuis, Amsterdam)*; Niels Harlaar, Frederik Jonker, Sjirk van der Burg, Justin Y. van Oostendorp *(Rode Kruis Ziekenhuis, Beverwijk)*; Saranda Ombashi, Tim van der Voort, Martijn van Geldorp *(Amphia, Breda)*; Lisanne Posma-Bouman *(Slingeland Ziekenhuis, Doetinchem)*; Hans Donald de Boer, Annette Olieman, Henriette Smid-Nanninga *(Martini General Hospital Groningen, Groningen)*; Jean-Paul P.M. De Vries, Rianne Hogenbirk, Schelto Kruijff, Milou Noltes, Pieter Steinkamp *(University Medical Center Groningen, Groningen)*; Tyche Derksen, Josephine Franken, Steven Oosterling *(Spaarne Gasthuis, Haarlem)*; Peter Nolte, Jelle van der List *(Spaarne Gasthuis, Hoofddorp)*; Ian Alwayn, Okker Bijlstra, Andries Braat, Ruth Bulder, Michèle de Kok, Robin Faber, Ben Goudsmit, Jaap Hamming, Sven Mieog, Alexander Vahrmeijer, Fenna E.M. van de Leemkolk, Joost van der Vorst, Jan van Schaik, Merel Verhagen *(Leiden University Medical Center, Leiden)*; Kim Albers, Larsa Gawria, Harry Van Goor, Michiel Warle *(Radboud Universitair Medisch Centrum, Nijmegen)*; Tessa M. van Ginhoven, Charlotte Viëtor *(Erasmus MC Cancer Institute, Rotterdam)*; Evert-Jan Boerma, Lara Lallitsch, Donald Schweitzer *(Zuyderland Medical Centre, Sittard/Heerlen)*; Wouter Leclercq, Julie Sijmons, Peter-Jan Vancoillie *(Máxima Medical Center, Veldhoven)*; Joop Konsten, Maarten van Heinsbergen *(VieCuri Medisch Centrum, Venlo)*; Nicole Dekker, Frank den Boer *(Zaans Medisch Centrum, Zaandam)*. |
| **New Zealand**: Julian Dimech, Kaveh Djamali, Carolyn Fowler, Naji Ghamri, Helen Houston, Reece Joseph, Astha Kantroo, Jin Kwun, Samuel Lie, Nicholas Lightfoot, Andrew MacCormick, Sue Olliff, Natasha Trilokekar, Daniel Wood *(Middlemore Hospital, Auckland)*; Aruna Ekanayaka, Chris Furkert, Asanga Nanayakkara, Divyansh Panesar, Rebecca Teague, Oliver Waddell *(North Shore Hospital, Auckland)*; Lauren Bidois, Zoe Clifford, Brodie Elliott, Frank Frizelle, Tamara Glyn, Eric Lim, Oliver Lyons, Jared Mclauchlan, Ella Nicholas, Michael O’Grady, Justin Roake, Harsh Singh *(Christchurch Hospital, Christchurch)*; Ramez Ailabouni, David Kieser, John Rietveld *(Southern Cross Hospital, Christchurch)*; Mohammed Alqassab, Mostafa Amer, Annie Chan, John Egbuji, Mairi Fullarton, James Haddow, Robyn Hawkins, Vui Teck Lu, Omar Mohyieldin, Thomas Oliver, Kai Sheng Saw, Ramanen Sugunesegran, James Wilkins, Esther Woodfield *(Dunedin Public Hospital, Dunedin)*; Joseph Baker, Jitoko Cama, Xavier Field, Alamea Fulivai, Ruhella Hossain, Ben Kelly, Jasen Ly, James McKelvie, Vivek Meiyappan, Cindy Xin Yi Ou, Vidit Singh, Chris Varghese, William Xu *(Waikato Hospital, Hamilton)*; Alex Hedley, Nick McIntosh, Hinerangi Temara *(Hawke’s Bay Hospital, Hastings)*; Jake Aitken, Brytt Frunt, Cheyaanthan Haran, Max Hardie Boys, Prashant Lakshman, Esther Pinfold, Sarah Rennie *(Wairarapa Hospital, Masterton)*; Sarah Cowan, Nigel Henderson, Jasmin King, Jessica Leary *(Taranaki Base Hospital, New Plymouth)*; Cameron Castle, Matt Gaston, Jeong Ha, Maira Haimona, Steffanie Jury, Zoe Lahood, Matthew Lim, Dillon MacIntyre, Josephine Mak, Annelise Neal, William Shelker, George Weeratunga *(Wellington Regional Hospital, Wellington)*; Liam Ferguson, Christopher Harmston, Chelsea L Heaven, Matthew James McGuinness, Imogen Watt, Henry Witcomb Cahill *(Whangarei Hospital, Whangarei)*. |
| **Nigeria**: Abdussemee Abdurrazzaaq, Hameedat Opeyemi Abdussalam, Ademola Adebanjo, Aminat Olayinka Ahmed, Opeyemi Busayo Borokinni, Olushola Kayode Fasiku *(Federal Medical Centre, Abeokuta)*; Usman Gwaram, Onyedika Okoye, Oluwole Olaomi *(National Hospital, Abuja)*; Ademola Adeyeye, Akinola Akinmade, Elizabeth Enoch, Samuel Fayose, Victor Kayode-Nissi *(Afe Babalola University Multi-System Hospital, Ado Ekiti)*; Innih Kadiri, Stella Oduah, Julius Olaogun *(Ekiti State University Teaching Hospital, Ado-Ekiti)*; Mohammed Abdull, Mohammed Kabir Abdullahi, Auwal Babayo Kwankiyel, Shahir Bello Umar, Kefas Bwala, Sulaiman Hassan, Abubakar Bappah Ja’afar, Umma Hani Ja’afaru, Saidu Kadas, Haruna Liman, Baje Makama, Aminu Mohammad Mohammad, Abdullahi Musa Kirfi, Funmilayo Oyediji, Husseini Yakubu Mohammed *(Abubakar Tafawa Balewa University Teaching Hospital Bauchi, Bauchi)*; Peter Agbonrofo, Oghenevwegba Akpoghor, Alexander Arekhandia, Oseyi Dawodu, Ekaniyere Edetanlen, Chizoba Efobi, Esezobor Egbor, Nosakhare Enaruna, Michael Ezeanochie, Ekene Ezenwa, Ferdinand Ijekeye, Joe Imumoren, Oseihie Iribhogbe, Omorodion Irowa, Moses Momoh, Nnamdi Nwashilli, Osarobo Obahiagbon, Ozoemene Obuekwe, Emeka Danielson Odai, Prof Vincent Odigie, Oluwatomi Odutola, Amina Okhakhu, Stanley Okugbo, Samson Oni, Ngozi Onyeagwara, Eustace Oseghale, Osarenkhoe Osemwegie, Philip Osho, Veronica Ugwi, Charles Uwagboe, Sofiat Yusuf *(University of Benin Teaching Hospital, Benin City)*; Adekunle Abiodun, Olufemi Akinloa, Moses Shodipo *(Federal Medical Centre Bida, Bida)*; Alhassan Abdullahi, Akinwale Afolabi, Ishak Lawal *(Federal Medical Centre, Birnin-Kebbi)*; Chidiebere Peter Echieh, Chimaobi Nwagboso, Ubong Udoh *(University of Calabar Teaching Hospital, Calabar)*; Bolaji Akanni, Solomon Anyimba, Isaac Chukwu, Sebastian Ekenze, Uchechukwu Ezomike, Ikponmwosa Gold, Ikenna Nnabugwu, Ikechukwu Nwafor, Louis Okolie, Kelechukwu Okoro, Blasius Okwara, Ndubuisi Onyemaechi *(University of Nigeria Teaching Hospital, Enugu)*; Khalifa Abdulsalam, Abayomi Arogundade, Mohammed Kura, David Nwosu *(Federal Teaching Hospital, Gombe, Gombe)*; Habiba Abdullahi, Eyaofun Agida, Oseremen Aisuodionoe-Shadrach, Hafees Ajibola, Godwin Akaba, Omolabake Ale, Terkaa Atim, Malachy Ayogu Emeka, Adeka Benard, Amina Buba, John Chinda, Stephen Edino, Sefiu Eniola, Stephen Garba, Aliyu Isah, Dennis Anthony Isah, Zaman Joshua, Ndubuisi Mbajiekwe, Philip Mshelbwala, Rafat Muhammad, Richard Offiong, Samson Olori, Anne Olute, Olabisi Osagie, Idoko Pius Ogolekwu, Bilal Sulaiman, Salisu Suleiman, Aminu Umar *(University of Abuja Teaching Hospital, Gwagwalada)*; Olukayode Abayomi, Rukiyat Abdus-salam, Sikiru A. Adebayo, Amos Adeleye, Samuel Ademola, Oludolapo Afuwape, Akinlabi Ajao, Sanusi Akinsola, Timothy Aladelusi, Omobolaji Ayandipo, Lateef Ayodele Baiyewu, Mosimabale Balogun, Israel Daodu, Ifeanyichukwu Kelvin Egbuchulem, Peter Elemile, Constantine Ezeme, Ayotunde James Fasunla, Olalere Gbolahan, Ifeanyi Iwuagwu, Taiwo Akeem Lawal, Afieharo Michael, Jemiludeen Morhason-Bello, Onyekwere Nwaorgu, Izegaegbe Obadan, Olubunmi Odeyemi, Omowonuola Ogundoyin, Olakayode Ogundoyin, Segun Ayodeji Ogunkeyede, Tolulope Ogunrewo, Oghenekevwe Okere, Titilola Oladejo, Ajibola Oladiran, Naomi Olagunju, Ifedolapo Olaoye, Olayinka Olawoye, Dare Olulana, Adeola Olusanya, Akinyinka Omigbodun, Paul Onakoya, Mathias Orji, Babatunde Osinaike, Oyeleye Oyelakin, Olugboyega Oyewole, Samuel Sule, Augustine O Takure, Ebere Ugwu *(University College Hospital, Ibadan)*; Adesoji Ademuyiwa, Bosede Afolabi, Peter Ajayi, Opeyemi Akinajo, Felix Alakaloko, Oluwole Atoyebi, Orimisan Belie, Chris Bode, Ihediwa Chibuike George, Olumide Elebute, Francis Ezenwankwo, Adedeji Fatuga, Oluwaseun Ladipo-Ajayi, Ayomide Makanjuola, Christian Makwe, Bolaji Mofikoya, Ephraim Ohazurike, Rufus Wale Ojewola, Kehinde Okunade, Adeyemi Okunowo, Thomas Olagboyega Olajide, Oluwafemi Oni, Justina Seyi-Olajide, Kehinde Tijani, Andrew Ugburo, Aloy Okechukwu Ugwu *(Lagos University Teaching Hospital, Idi Araba)*; Paul Abiola, Henry Abiyere, Idowu Adebara, Adebayo Adeniyi, Olabisi Adeyemo, Abimbola Ariyibi, Olakunle Babalola, Adewumi Bakare, Oluseyi Banjo, Peter Egharevba, John Obateru, Owolabi Ojo, Abiodun Okunlola, Adewale Olajide, Fatudimu Oluwafemi, Oluwole Oyeleye, Adedayo Salawu, Bakare Tajudeen Ishola Babatunde *(Federal Teaching Hospital, Ido Ekiti, Ido Ekiti)*; Moruf Abdulsalam, Aderinsola Adelaja, Abimbola Adeniran, Olalekan Ajai, Kazeem Atobatele, Olabamidele Ayodele, Funmi Bello, Omolara Faboya, Olufemi Idowu, Jeuel Idowu, Muntaqa Mustapha, Olufunmilade Omisanjo, Roland Osuoji, Julius Vitowanu, Omolara Williams *(Lagos State University Teaching Hospital, Ikeja)*; Anthonia Abe, Adewale Aderounmu, AbdulHafiz Adesunkanmi, Adewale Adisa, Temitope Ajekwu, Samuel Ajekwu, Olalekan Ajiboye, Adeleke Akeem Aderogba, Olusegun Isaac Alatise, Mustapha Babatunde, Simon Balogun, Adebimpe Ijarotimi, Adedayo Lawal, Olugbenga Ojo, Olalekan Olasehinde, Adebayo Olugbami, Akaninyene Eseme Ubom, Funmilola Wuraola *(Obafemi Awolowo University Teaching Hospitals Complex, Ile-Ife)*; Ademola Agbaje, Tajudeen Mohammed *(Obafemi Awolowo University Teaching Hospitals Complex Wesley Guild Hospital Unit, Ilesa)*; Lukman Abdur-Rahman, Abiodun Adeniran, Muideen Adesola, Ademola Adeyeye, Abdulrahman O. Afolabi, Isiaka Aremu, Iyehunwa Ayinmode, Jibril Bello, Grace Ezeoke, Sadiya Gwadabe, Gbadebo Ibraheem, Saidu Ibrahim, Munir’deen Ijaiya, Abdulrasheed Nasir, Peter Olalekan Odeyemi, Olukayode Ogunade, Afusat Olabinjo, Samuel Adegboyega Olatoke, Tope Olowogbayi, Stephen Onjefu, Ademola Popoola, Hadijat Olaide Raji, Nasiru Salawu, Hafeez Salawu, Tolulope Olayinka Sayomi, Segun Segun-Busari, Kenechukwu Uche-Okonkwo *(University of Ilorin Teaching Hospital, Ilorin)*; Olajide Abiola, Olalekan Anipole, Olumuyiwa Ogunlaja *(Bowen University Teaching Hospital, Iwo)*; Oluwaseyi Adesina, Barnabas Alayande, John Ebenezer, Kenneth Enwerem, Christian Isichei, Mercy Isichei, Pwaluke Luku, Paul Omiragi, John Onyeji *(Faith Alive Foundation, Jos)*; Joel Adze, Ifeanyi Aghadi, Akawu Auta, Sharon Duniya, Joseph Gyuro, Stephen Kache, Jamila Lawal, Jerry Godfrey Makama, Garba Mohammed, Amina Mohammed-Durosinlorun, Kabiru Salisu, Matthew Taingson, Nuhu Yusuf *(Barau Dikko Teaching Hospital, Kaduna)*; Happy Amos, James Daniel, Danjuma Sale *(Epsilon Specialist Hospital Barnawa, Kaduna)*; Idris Usman Takai, Lawal Abdullahi, Sani Abdullahi, Mamuda Abdulrahman, Mohammed Kabir Abubakar, Valentine Adikaibe, Misbahu Ahmad, Jameel Ismail Ahmad, Sani Ali Aji, Mohammed Salele Aliyu, Lofty-John Anyanwu, Maryam Babba Danagundi, Yusuf Halidu Bako, Nafisatu Bello-Muhammad, Ibrahim Umar Garzali, Muhammad Ghazali Hasheem, Ismail Hassan, Sadiq Hassan, Mustapha Ibrahim Usman, Nasiru Ismail, Yasir Nuhu Jibril, Musbahu Kurawa, Mahmoud Kawu Magashi, Bardi Martins, Usman Mohammed Bello, Saminu Muhammad, Abubakar Bala Muhammad, Shamsuddeen Muhammad, Sulaiman Muhammad Daneji, Ibrahim Aliyu Mukhtar, Dr Kabir Musa Adamu, Callistus Nwachukwu, Uchenna Kelvin Omeje, Ifeanyi James Orji, Abdurrahman Abba Sheshe, Ibrahim Suleiman, Sani Abdullahi Yunusa *(Aminu Kano Teaching Hospital, Kano)*; Maryam A, Kabiru Bello Abubakar, Saudat Garba Habib, Farrukh Mahmood *(Makkah Specialist Eye Hospital, Kano)*; Ibrahim Abolaji Alabi, Soliudeen Arojuraye, Mohammed Salihu *(National Orthopaedic Hospital Dala, Kano)*; Samuel Alade, Felix Kumolalo, Chiedozie Mogbo *(University of Medical Sciences Teaching Hospital Complex, Ondo)*; Olaolu Adebayo, Oluwasuyi Ige, Gbenga Jones, Tunde Odunafolabi, Chukwuma Okereke, Oluniyi Oyetunde Olubayo *(Federal Medical Centre, Owo, Ondo State)*; Chris Akani, Datonye Alasia, Justina Omoikhefe Alegbeleye, Oti Nimi Aria, Abimbola Awopeju, Kemebradikumo Edonkumoh, Onyeanunam Ekeke, Sotonye Fyneface-Ogan, Celestine John, Terhemen Kasso, Inye Korubo, Christie Mato, Rosemary Ogu, Charles Okpani, Anthony Okpani, Ijeoma Onwuagha, Ijeoma Oppah, Victory Oputa, Emmanuel Oranu, Ngozi Orazulike, Dr Job Otokwala, Henry A.A Ugboma *(University of Port Harcourt Teaching Hospital, Port Harcourt)*; Adekunle Ajayi, Ibukunolu Ogundele, Babatunde Salami *(Olabisi Onabanjo University Teaching Hospital, Sagamu)*; Okeoghene Ajagha, Yakubu Ali, Mudi Awaisu, Muhammad Daniyan, Lovely Fidelis, Stanley Emeka Nwabuoku, Oluseyi Ogunsua, Nasir Oyelowo, Tunde T. Sholadoye, Musliu Adetola Tolani, Alfa Yakubu *(Ahmadu Bello University Teaching Hospital, Zaria)*. |
| **North Macedonia**: Venko Filipce, Vladimir Rendevski, Blagoj Shuntov *(University clinic for neurosurgery, Faculty of medicine, University St.Cyril and Methodius, Skopje)*; Natalija Cokleska Shuntov, Lazar Todorovic *(University Clinic for Pediatric Surgery, Skopje)*; Gordana Georgieva, Tomislav Jovanoski, Katerina Jovanovska, Bisera Nikolovska, Igor Peev, Sofija Pejkova, Blagoja Srbov, Andrijana Trajkova, Viktor Trenchev *(University Clinic for Plastic and Reconstructive surgery, Faculty of Medicine, University St. Cyril and Mthodius, Skopje)*; Sasho Dohcev, Josif Janchulev, Skender Saidi, Oliver Stankov, Sotir Stavridis, Aleksandar Trifunovski *(University Clinic of Urology, Skopje)*. |
| **Oman**: John George Massoud *(Khoula Hospital, Muscat)*; Najib Abu Draz, Khalil Al Ajmi, Ghadeer Al Ajmi, Ayman Al Amri, Ghalib Al Badaai, Nawf Al Balushi, Zainab Al Balushi, Wameedh Al Bassam, Maather Al Farsi, Wadha Al Ghafri, Alaa Al Ghafry, Mohammed Al HOSNI, Moza Al Kalbani, Maha Al kalbani, Lamya Al Kharusi, Safiya Al Kharusi, Mohammed Al Mutani, Hani Al Qadhi, Amani Al Raisi, Nihal Al Riyami, Hilal Al Sabti, Maha Al shaibi, Rashid Al-Abri, Abdullah Al-Mujaini, Aya Al-Rashdi, Suha Alajmi, Zamzam Albadi, Hanadi AlBusaidi, Ahmed Alhadeethi, Noor Aljabri, Muhannad Alkazrooni, Nadiya Alkharousi, Al-Salt Alkharusi, Khadija ALKiyumi, Noof AlMaharbi, Sabaa Alsaadi, Marwa Alsharji, ALi Hamed ALSharqi, Fatma AlShehhi, Sareyah Alsibai, Thuwaiba Alsulaimani, Mustafa Alward, Mohammed Alwashahi, Hussain Alyafii, Adil Alzadjali, Tayseer Basi, Khadija Dalmar, Bashar Dawud, Mohamed Mourad Gargouri, Rajeev Kariyattil, Ahmed Omer Kenawy, Koshy Kochummen, Arif Kolethekkat, Mahesh Krishna Pillai, SureshKannan Ks, Farman Ali Laghari, attibele Palaksha Manjunatha, Joseph Kunju Mathew, Rahil Muzaffar, Asha Nair, Hafsa Ismail Ibrahim Naiya, Zuhail Nazar, Silja Pillai, Ferdous Rahim, Khalid Rahman, Shahila Sheik, Mohammad Salman Siddiqi, Edwin Stephen, Shahzad Younas *(Sultan Qaboos University Hospital, Muscat)*. |
| **Pakistan**: Mishal Fatima, Ayesha Javed, Muhammad ahsan Khan, Fatima Tuz Zahra Shakir, Riaz Hussain Siddiqui, Rizwan Sultan, Danish Syed, Ishtiaq Ur Rehman, Shahrukh Warraich *(Islamabad medical complex, Islamabad)*; Humera Naz Altaf, Mohammed Amir, Saleha yurf Asghar, Abu Bakar Hafeez Bhatti, Ali Haider Bangash, Dr. Sehrish Latif, Wajih Naqvi *(Shifa international hospital, Islamabad)*; Muhammad Tariq Abdullah, Saleha Abdullah, Arooj Ahmed, Muhammad Fahim Ahsan Ahsan, Fahad Akhtar, Sana Ali, Mehwish Ansar, Muhammad Aqib, Muhammad Arslan, Sumaira Ashraf, Azaz Ayubi, Tayyab Azam, Hamza Bhatti, Shahab Huda, Abeer Irshad, Nazia Ishaque, Tanwir Khaliq, Farheen Khan, Dr. Mumtaz Ahmad Khan Khan, Dr Namrah Mahmood, Ali Raza Malik, Tahir Malik, Komail Malik, Dr Manzer Mehmood, Dr. Fatima Mustafa, Dr Fazal Noor, Sarwar Nubair, Isbah Rashid, Waqar Saeed, Hannan Sajid, Sajid Shah, Dr Ali Shami, Minhaj us siraj Siraj, Mariam Tareen, M Burhan Ul Haq, Shahzad Hussain Waqar *(The Pakistan Institute of Medical Sciences, Islamabad)*; Manzar Abbas, Syeda Maria Ahmad Zaidi, Iffat Ahmed, Shahnoor Ahmed, Kunwar Armash Ahsan, Munazza Akhtar, Ramsha Akhund, Adil Al-Karim Manji, Hajra Arshad, Muhammad Ozair Awan, Aliya Aziz, Humaira Aziz, Shameen Bhutto, Faiqa Binte Aamir, Syed Raziuddin Biyabani, Uzma Chishti, Mohsin Chundrigar, Shayan Khalid Ghaloo, Waqqas Haroon, Shiraz Hashmi, Murtuza Hassan, Hina Inam, Nausheen Kassam, Abbas Kazmi, Majid Khan, Muhammad Osama Khan, Sameeta Kumari, Muhammad Jehangir Malik, Maheen Mansoor, Russell Seth Martins, Areeba Mubarik, Muhammad Musaab Munir, Muhammad Bazil Musharraf, Falak Naz, Shahryar Noordin, Arsalan Pervaiz, Javeria Bilal Qamar, M Fazlur Rahman, Pallavi Rani, Yasir Rasheed, Haleema Sadia, Marwah Saeed, Mohammad Safri, Shaharyar Salim, abida K. Sattar, Shahzad Shamim, Maryam Sherwani, Muhammad Ahsan Iqbal Siddiqui, Astad Sidhwa, Javeria Tariq, Isbaah Tejani, Usama Waqar, Fahad Zahid *(Aga Khan University, Karachi)*; Abid Jamal, Asad Ali Kerawala *(Cancer Foundation Hospital, Karachi)*; Muhammad Ali Ghufran, Amjad Siraj Memon, Lajpat Rai, Summaya Saeed, Khursheed Ahmed Samo *(Dr Ruth K.M. Pfau Civil Hospital, Karachi)*; Mariyah Anwer, Manisha Aswani, Shahneela Manzoor, Shoaib Muhammad *(Jinnah Post Graduate Medical Center, Karachi)*; Muhammad Arshad, Syeda Namayah Fatima Hussain, Inshrah Moin, Muhammad Kazim Rahim Najjad, Manal Nasir *(Liaquat National hospital, Karachi)*; Osama Bin Sohail, Ali Mangi, Naveed Markhand, Hassan Mushtaq, Aiman Tariq, Kashif Zia *(National Institute of Cardiovascular Diseases, Karachi)*; Dr. Hassaan Ahmed, Nasir Ahmed, Talha Ahmed Qureshi, Shafqat Ali Shaikh, Rabia Anwer, Owais Arshad, Bushra Ayub, Sehrish Batool, Muhammad Chatni, Tariq Chundrigar, Samina Saleem Dojki, Minahil Elahi, Bakhtawar Farooqui, Amna Ghouri, Nabeel Hassan, Zainab Hussain, Dr Hussaini, Fizza Iftikhar, Faizan Iqbal, Anum Javed, Dania Javed, Wajahat Kamal, Shifa Khan, Zeeshan Uddin Mughal, Ghulam Murtaza, Areeba Nadeem, Areeba Nasir, Fatima Nayyer, Kanwal Nisa, Yaseen Rauf, Dr. Umer Saeed Haroon, Shah Masabat Saleem, Raza Sayyed, Haseeb Seriwala, Aaqil Shah, Safia Siddiqui, Asher Tanweer Tanveer, Nida Tariq, Junaid Zaman, Mehroz Zamir *(Patel Hospital, Karachi)*; Sami ullah Niazi *(South City Hospital, Karachi)*; Muhammad Ayyub Anjum, Muhammad Yasir Naseem *(Government Mian Meer Hospital Lahore, Lahore)*; Ameer Afzal, Ehsan Ahmed, Jawad Ahmed, Shujat Ahmed Riaz, Ali Akbar, Bilal Akbar, Maria Ali, Imran ali Ali, Abrar Ashraf Ali, Ghazanfar Ali, Zahra Altaf, Ahmed Siddique Ammar, Hafiz Muhammad Arif Arif, Imran Aslam, Muhammad Mustehsan Bashir, Usman Farooq, Muhammad Farooq, Khalid Masood Gondal, Muhammad Javed Iqbal, Zafar Iqbal, Fahad Ismail, Muhammad Khurram Jameel, Muhammad Haris Janjua, Azwa Janjua, Prabesh Joshi, Mamoona Khadam, Romaisa Khan, Muhammad Asadullah Khawaja, Sobia Manzoor, Muhammad Mohsin, Khizra Mumtaz, Muhammad Musaab, Syed Asghar Naqi, Fatima Naumeri, Umer Nazir, Dr. Sadaf Noureen, Yaseen Rafi, Ali Raza, Sushil Rijal, Irfan Saleem, Muhammad Zeeshan Sarwar, Hafiz Syed Zaigham Ali Shah, Muhammad Sharif, Ayesha Shaukat, Usman Siddique, Hafiz Saqib Sikandar, Mohammad Sohail, Fatima Tu zahara, Yasmeen Usman, Balakh Sher Zaman, Misbah Zeb *(King Edward Medical University - Mayo Hospital, Lahore)*; Muhammad Farooq Afzal, Fateen Ahmad, Waqas Ahmed, Shahzad Akram, Azhar Alam, Danish Ali, Ahsan Ali, Ammar Tariq Alvi, Suleman asif Asif, Madiha Aslam, Muhammad Daood Daood, Muhammad Jawad, Usama Muhammad Kathia, Ismaeel Khalid, Anwar Khan, Muhammad Imran Khokhar, Zain Khurshid, Hamza Noman, Ahsan Raza, Dr Hassan Taha, Muhammad Zubair *(Lahore General Hospital, PGMI, AMC, Lahore)*; Noor Ul Huda Maria *(Punjab Institute of Neurosciences, Lahore)*; DrAtif A Janjua, Qamar Ashfaq Ahmad, Sheraz Ahmad, Imran Ali, Sana Amin, Momina Anjum, Muhammad Asjad, Aatif Aslam, Mahmood Ayyaz, Luqman Ali Bajwa, Akkasha Bakhtiar, Alia Bashir, Fizza Batool, Samiullah Bhatti, Roshan Butt, Usman Butt, Shabbar Hussain Changazi, Omer Iqbal Cheema, Muhammad Haris Chishti, Bushra Haq, Ali Hashmi, Muhammad Hassan, Usman Hassan, Khizar Hayat, Javaid Iqbal, Ayesha Iqbal, Amna Ishaq, Sundas Javed, Hina Javed, Aymen Javed, Muhammad Kashif, Muhammad Saud Khalid, Wasim Hayat Khan, Rohma Khan, Fatima Khan, Usman Latif, Arif Mahmood, Faizan Majeed, Kiren Malik, Rumaisa Masood, Fasiha Munawwar, Asma Mushtaq, Muhammad shoaib Nabi, Ahmad Nasir, Shazia Naureen, Dr Zahida Noreen, Muhammad Ghulam Qadir Qadir, Ahmad Uzair Qureshi, Dr. Gul e Raana Raana, Madeeha Rashid, Abdul Rehman, Sidra Riaz, Arfa Saboor, Muhammad Hamza Sadiq, Komal Saeed, Dr Humaira Saleem, Kiran Sarfraz, Zahid Shafiq, Aymen Shafqat, Hassan Shaukat, Fateh Sher, Rubina Sohail, Amina Suhail, Bilal Syed muhammad, Usama Umair, Muhammad Umar, Muhammad Usama, Natasha Usman, Kalsoom Waheed, Muhammad Waris Farooka, Shafqat Wasim, Tayyiba Wasim, Fahad Yasin, Zain Zafar, Imdad Ahmad Zahid, Sabahat Zaka, Salma Zaman Zaman *(Services Hospital Lahore, Lahore)*; Junaid Azad, Zareen Fatima, Muhammad Imran Anwar, Haroon Javaid Majid, Muhammad Ahmed Naseer, Muhammad Taimoor Shah *(Shaikh Zayed Hospital, Lahore)*; Ammar Ali, Muhammad Aslam, Muhammad Usama Aziz, Farhan Gohar, Dr. Waseem Humayoun, Hassan Khan, Shiza Naeem, Warda Tahir, Nabila Talat *(The Children’s Hospital & The Institute of Child Health Lahore, Lahore)*; Kashif Alvi, Muhammad Asif, Maria Mehmood *(District Headquarter Hospital - Mandi Bahauddin, Mandi Bahauddin)*; Muhammad Ali, Muhammad Hamid Chaudhary, Aniqa Shahzad, Muhammad Sher e Murtaza *(Chaudhary pervaiz elahi institute of Cardiology, Multan)*; Hijab Siddique, Waleed Anjum, Umer Farooq *(Tehsil Headquarters Hospital Pasrur, Pasrur district Sialkot)*; Manzoor Ahmad, Siddique Ahmad, Muhammad Alam, Fouzia Faizi, Shehla Faridoon, Musarrat Hussain, Muhammad Iftikhar, Yousaf Jan, Muhammad Kalim, Tilal Ahmed Raza, Farrukh Ozair Shah, Muhammad Shah, Imran Tahir *(Hayatabad Medical Complex, Peshawar)*; Hamza Khan, Waleed Mabood, Muhammad Naeem *(Khyber teaching hospital, Peshawar)*; Shabir Ahmad, Haseeb Ahmed, Jawad Ahmed, Hazrat Akbar Akbar, Muhammad Shaheer Akhtar, Noman Ali, Mehboob Ali, Sadaf Ali, Irfan Ali, Hasbi Allah Amin, Dr shehzadi Amin, Muhammad Asif, Bakhtawar Ayub, Muhammad Basir, Syed Imran Bukhari, Ahmad Faraz, Muhammad Salman Farsi, Ambrin Gul, Hina Gul, Ayaz Gul, Sana Gul, Dr Sajjad L Haider, Syed Ikramullah Ikramullah, Kashif Jan, Muhammad Khalid, Salman Khan, Muhammad faisal Khan, Faraz ahmad Khan, Iftikhar Mian, Aiman Moeen, Obaid Mohmand, Rehana Rahim, Naila Raziq, Riaz RehmAn, Azra Salim, Tanveer Shafqat, Faaiz Ali Shah, Suliman Shah, Muhammad Shakeel, Tahsin Ullah, Shams Ur Rehman, Sabaeena Wali, Farnaz Zahoor, Gulmeena Zeb, Dr Laila Zeb, Haseeb *(Lady Reading Hospital, Peshawar)*; Waqas Raza, Muhammad Tariq *(North West General Hospital and Research Centre, Peshawar)*; Zein el Amir, Daneyal Arshad, Mashal Daud, Umer Javed Chughtai, Mirza Hammad Rauf, Zaina Sajid *(Benazir Bhutto Hospital, Rawalpindi)*; Rubaba Abid, Ayesha Azam, Tallat Farkhanda, Kanwal Firdos, Tabinda Khalid, Sobia Malik, Javeria Mashal, Ruqyyah Salim, Madiha Saqib, Aneela Shaheen, Madiha Shahid, Irfana Yasmeen *(District Headquarter Hospital - Rawalpindi, Rawalpindi)*; Faryal Ahmed, Aneeqa Ali, Zarafshan Amjid, Muhammad Idrees Anwar, Iqtaza Arif, Anam Ashraf, Adam Umair Ashraf Butt, Faryal Azhar, Muhammad Daniyal Daniyal, Ramlah Ghazanfor Ghazanfor, Daniyal Ishtiaq, Manahil Jamil, Saad Javed, Ziaullah Khalid, Jahangir Sarwar Khan Khan, Muhammad Sarfraz Khan, Usama Mahmood, Ashraf Mahmood, Abdur Rehman Malik, Ramisha Naseer Nagra, Fuad Ahmad Khan Niazi, Maleeha Saleem, Bushra Saleem, Tayyaba Saleem, Raja Khalid Shabbir, Imtiaz Ahmed Shakir, Eesha Yaqoob *(Holy Family Hospital, Rawalpindi)*; Ashfaq Ahmad, Surgeon Hafiz Riaz Hussain Awan, Muhammad Usman Malik *(District Headquarter & Teaching Hospital - Sargodha, Sargodha)*; Waqar Bhatti, Azka Shaheen, Hassan Nawaz Yaqoob *(Social Security Hospital, Sheikhupura, Sheikhupura)*. |
| **Palestine**: Hadeel Awad, Balqees Mohamad *(Beit Jala Governmental Hospital (Al Hussein), Bethlehem, West Bank)*; Dalia Wafi *(Al-Shifa Hospital, Gaza)*; Mustafa Abu Jayyab, Hitham Toman *(European Gaza Hospital, Gaza)*; Hamdoon Abu-Arish, Ibrahim Al-Slaibi, Hussam I. A. Alzeerelhouseini, Fahmi Jobran, Raghad Lahlooh *(Al-Ahli Hospital, Hebron, West Bank)*; Sadi A. Abukhalaf, Fatima Aburayyan, Khalil Abuzaina, Ayat A. Aljuba, Roaa Aljunaidi, Tareq Alzughayyar, Adham Amro, Sarah Amro, Mays Dweik, Safa Halman, Qutaiba Qafisha, Farah Shaheen, Balqis Shawer, Daleen Shehadeh, Jihad Zalloum *(Governmental Hebron Hospital-Alia, Hebron, West Bank)*; Khaled Demyati, Mohammed Hajhamad, Zain Saqfalhait *(Rafidia Hospital, Nablus, West Bank)*. |
| **Panama**: Emmy Arrue Del Cid, Moises Cukier, Homero Rodriguez-Zentner *(Pacifica Salud Hospital, Panama)*. |
| **Paraguay**: Francisco Abed, Rafael Osmar Adorno Garayo, Ruben Aguilar, Ana Aguilar, Domingo Javier Aguilera Maidana, Diego José Almada Casañas, Teresa Almiron, Analia Arrua, Pedro Avila, Brenda Balmaceda, Alexandra Barreto, Analy Barreto Galeano, Belinda Barrientos Nuñez, Magdalena Benegas, Martin Berden, Amalia Bogarin, Jorge Bonnin, Edulfo Britez Barrios, Julio Burgos, Carlos Fabián Cárdenas Melgarejo, José De Jesus Casco Samudio, Sandra Cataldi, Jorge Augusto Centurion, Luz Maria Cespedes Ramirex, Katerine Colina, Gabriel Augusto Cuevas Almando, Karina De Bleecker, Antonella Dragotto, Eiji Eiwa, Juan Carlos Enciso, Carla Espinola, Lujan Estigarribia, Celso Ariel Fernandez, Rocio Ferreira, Blas Figueredo, Daniel Fleitas, Claudia Fretes, Martin Fretes, Walter Rene Fretes Gonzalez, Humberto Nicolas Galleano Ruiz, Julio Gauto, Claudia Gauto, Viviana Gomez, Nadia Gómez, Osvaldo Insfran, Liz Karina, Christian Legal, Oscar Llanes, Walter Martínez, Walter Rodrigo Martínez Torres, Kiichiro Matsumura, Esteban Daniel Mendoza Galván, Leila Mereles Noguera, Sergio Mora, Daniel Navarro, Lidia Noguera Roman, Analia Núñez, Lorena Ocampos, Alma Maria Ojeda Rojas, Derlis Joel Ojeda Villasboa, Juan Palacios, Jessica Pamela Portillo Sosa, Rossana Ramírez, German Ramos, Diego Samudio, Jorge Eduardo Sisa Acosta, Rodney Tellez, Porfiria Urquhart, Beatriz Vera, Jorge David Vera Florentin, Daniel Vidallet, Gilce Teresita Villalba, Jacob Vömel *(Hospital Central Instituto de Prevision Social, Asuncion)*; Laura Aquino, Marisa Natalia Martinez, Cesareo Saldivar Patiño *(Hospital General de Barrio Obrero, Asuncion)*; Omar Aguayo, Maria Esther Ferreira Aguilera, Federico García, Maria Giangreco, Derlis Osorio, Cynthia Marlene Rodríguez Sosa, Paulo Villanueva *(Sanatorio Migone Battilana, Asuncion)*; Sandra Elizabeth Centurion Rolon, Rozana Ohara, Juan Marcelo Portillo, Ruben Milciades Varela Cano *(Sanatorio Santa Julia - Grupo San Roque, Asuncion)*; Juan Manuel Baez Melgarejo, Francisco Vidal Candia, Carlos Chirico, Sofia Belen Diaz Pineda, Diego Alfonso Paiva Vera, Ayrton Facundo Valdovinos, Dahiana Fatima Velazquez *(Hospital de Trauma Manuel Giagni, Asunción)*; Diego Raul Abente Arriola, Analia Britos, Daniel Cantero, Jessica Centurión, Celso Ariel Fernandez, Ricardo Ignacio Olmedo Bareiro *(Sanatorio San Roque, Asunción)*; Dayhana Duarte, Susana Paredes, Carlos Pfingst Rojas *(Hospital Nacional de Itauguá, Itauguá)*; Angel Luis Agüero Delgado, Juan Marcelo Delgado Godoy, Helmut Alfredo Segovia Lohse *(Hospital Distrital de Lambaré, Paraguay, Lambaré)*; Oscar Benitez, Victor Luraschi, Joel Molinas *(Hospital General de Luque, Luque)*; Dulce Añasco, Alejandro Arévalo Barreto, Cristhian Chavez Rivaldi, Martin Chenu, Luz Garcia, Rosana Godoy, Gustavo Miguel Machain V, Maria Jose Martinez Velázquez, Brayan David Pedroso Alvarenga, Agustin Rodriguez Gonzalez, Rosa Sánchez, María Liz Sánchez, Herald Rene Segovia Lohse, Cesar Giuliano Sisa Segovia *(Hospital De Clínicas, San Lorenzo)*; Osmar Cuenca, Sara Melgarejo, Ever David Sosa Ferreira *(Hospital Universitario San Lorenzo, San Lorenzo)*. |
| **Peru**: Glenda Marina Falcon Pacheco *(Instituto Regional de Enfermedades Neoplásicas del Sur, Arequipa)*; Estefany Angela Flores Anaya, Evy Yamil Huaman Huallanca, Karla Melissa Marchan Palma, Robinson Mas Melendez, Liliam Palomino, Arazzelly Del Pilar Paucar Urbina *(Ayacucho Regional Hospital, Ayacucho)*; Jorge Ordemar, Manuel Quiroz, Jose Rios Chiuyari *(Hospital regional de lambayeque, Lambayeque)*; Victor Diego Caballero Sarabia, Carlos Eduardo Otiniano Alvarado *(Arzopispo Loayza National Hospital, Lima)*; Lorena Fuentes Rivera Lau, Christian Lozano, David Torres *(British American Hospital, Lima)*; Henry Rafael Acosta Castro, Gabriel Bayona-Alvarado, Giuliano Borda-Luque, Joao Cruz, Grecia Lizzetti, Victor Vasquez Morales, Milagros Niquen-Jimenez, Martin Ormeño, Liz Ortiz, Kewin Quispe de la Roca, Darwin Artidoro Quispe-Cruz, Edgar Sanz, Sebastian Shu Yip, Katherine Tataje poma, Ximena Paola Vasquez Ojeda, Gaby Susana Yamamoto Seto *(Cayetano Heredia National Hospital, Lima)*; Claudia Arias, Carlos Palacios, Juan Francisco Pintado Mejia, Jose Maria Silva Barandiaran *(Edgardo Rebagliati Martins National Hospital, Lima)*; Eduardo Huaman, Guillermo Enrique Reyes Gamonal, Claudia Suazo Carmelo, Sergio Zegarra *(Guillermo Almenara National Hospital, Lima)*; Jenner Betalleluz Pallardel, Diana Lizet Cruz Condori, Jose Vasquez Valverde *(Hospital de Emergencias Jose Casimiro Ulloa, Lima)*; Eduardo Huaman, Regina Amparo Ugarte Oscco, Cesar Alberto Vergel Cabrera *(Hospital Emergencia Ate Vitarte, Lima)*; Consuelo Diaz, Gian Mendiola, Angie Paredes Caturiny, Adolfo Salazar, Joel Jesús Sánchez Estupiñan, Marco Sandoval vaez *(Hospital Santa Rosa de Lima, Lima)*; Roy Arangoytia, Francisco Berrospi, Jorge Luis Bustamante Polo, Jimmy Cárdenas Coaquira, Fernando Cárdenas Escalante, Darwin Oliver Desposorio Armestar, Sheyla Katherine Diaz Mora, Jheff Laura, Aldo Lopez Blanco, Karoll Tatiana Meza Garcia, Jaime Moreno, Eduardo Payet, Joan Perez Villena *(Instituto Nacional de Enfermedades Neoplásicas, Lima)*; Daniel Cardenas, Yahaira Tatiana Carpio Colmenares, Luis Alcides García Barrionuevo, Miguel Roberto Li Valencia, Percy Mansilla Doria, Fiorella Palomino Escalante *(SANNA - Clínica El Golf, Lima)*; Mónica Falcón Coronado, Carlos Shiraishi Zapata, Pamela Valderrama Rocha *(Hospital Il Talara, Talara)*; Ulbar Edinson, Maria Rosa Ortiz, Vladimir Laureano Velasquez Huarcaya *(Hospital Belén de Trujillo, Trujillo, La Libertad)*. |
| **Philippines**: Andrei Cesar Abella, Ma. Carmela Andal, Willmar Anoso, Monica Maria Bejasa, Aldrin Cuasay, Jesus Fernandez, Eunice Mercado, Amabelle Moreno, Alejandro Palines, Trina Regalado, Cristeta Unira, Clarence Pio Rey Yacapin *(Batangas Medical Center, Batangas City)*; Carlo Angelo Cajucom, Mary Anne Carol Cueto, Charmagne Loren Ramos *(José R. Reyes Memorial Medical Center, Manila)*; Avril David, John Isaac Merin, Alberto Roxas *(Manila Doctors Hospital, Manila)*; Nipseey Pauloe Candelario, Marc Paul Lopez, Ramon Alberto Ramos, Khrissa Lea Elisa Violago *(Ospital ng Makati, Manila)*; Yvann Kenneth Benosa, Louie Czelline De Leon, Marlow Esguerra, Rene Luis Filarca, Corina Gaddi, Patrick Jovan Gagno, Jeffy Guerra, Angelica Aubrey Morla, Rivero Opano, Edgardo Penserga, Mark Jheric Tesil, Dr. Hazel Turingan *(Ospital ng Maynila Medical Center, Manila)*; Joel Aldana, Alyssa Carmina Almelor, Jaime Jr. Almora, Angeli Anne Ang, Maria Angelica Arada, Jeric Arbizo, Diana Barretto, Ronnie Baticulon, Kevin Ivan P. Chan, Elmer Jason Cruz, Pauleen de Grano, Juan Gabriel De Leon, Jojiemar De Pano, Maria Angela Dealino, Daryl Anne del Mundo, Hans Jesper Del Mundo, Czarlo Dela Victoria, Juan Miguel Roberto Delgado, Jan Erik Detran, Robert Zaid Diaz, Edward Arlu Dinoy, Efren Domingo, Heather Grace Dulnuan, Patricio III Dumlao, Carlo Elises, Emmanuel Estrella, Joan Marie Flor, Grace Gana, Bynlee Stuart Go, Ana Melissa Hilvano-Cabungcal, Cesar III Jacinto, Jan Alexeis Lacuata, Marie Carmela Lapitan, Renee Leen Laudato, Gerardo Legaspi, Ryle Li, Liana Mae Lobo, Claudine Lukban, Christopher John Macapugay, Nikko Magsanoc, Enrique Manalang, Jon-Alexis Montemayor, Jaysonnel Notario, Marie Dione Sacdalan, Jan Armelynn Santos, Abigail Sarmiento, Dennis Serrano, Samantha Siahetiong, Krisniel Florence Solis, Denorson Sotalbo, Erik Tongol, Germaine Ursabia, Patricia Ann Uy, Charmaine Grace Valeros, Karen Joyce Velasco, Vincent Venida, Hans Edison Yap, Mario Angelo Zamora *(Philippine General Hospital, University Of The Philippines Manila, Manila)*; Anika Johanna Agoncillo, David Vincent Antonio, Nicole Robyn Bangayan, Marinelle Castro, Grace Lynn Estanislao, Anne Kathleen Ganal-Antonio, Faiyazudin Amado Ibrahim, Peter Jarin, Laine Valerie Kongsun Ching, Josephine Alexandra Lim, Mariles Nazal, Amanda May Ong Vaño, Louie Racelis, Kirsten Ramos, Jeryl Anne Silvia Reyes, Ron Daniel Rivera, Jose Antonio Salud, Elva Marita Sarte, Dale Kingsley Sy, Eileen Grace Tancinco, Anna Mariel Torio, Avegail Niña Uy *(The Medical City, Pasig)*; Ryan rainiel Abary, Jesus Jr Dabalos, Vincent Siquian, Kenneth Yabut *(Quirino Memorial Medical Center, Quezon city)*; Jose Carlos Barcelon, Ma. Inez Carballo, Jo Ann Chiu *(Cardinal Santos Medical Center, San Juan City)*; Aeris Jane D. Nacion *(Divine Word Hospital, Tacloban)*. |
| **Poland**: Piotr Major, Michał Pędziwiatr, Justyna Rymarowicz *(Jagiellonian University Medical College, Krakow)*; Marcin Bobiński, Jan Kotarski, Karolina Rasoul-Pelińska *(Independent Public Teaching Hospital No 1 in Lublin, Lublin)*; Konrad Futyma, Pawel Miotla, Ewa Rechberger *(Medical University of Lublin, Independent Public Teaching Hospital No 4, Lublin)*; Bogdan Koczy, Karol Szyluk *(District Hospital of Orthopedics and Trauma Surgery, Piekary Śląskie)*. |
| **Portugal**: Pedro Botelho, Mariana Branco Lopes, Brigitta Cismasiu, Paulo Matos Costa, Jean-Michel Fallah, Gonçalo Teodoro Fernandes de Freitas, Filipa Ferreira, José Guilherme Gonçalves Nobre, Susana Henriques, Rui Lino, Alexandre Macedo, Cláudia Mendes, Ana Lúcia Preto Barreira, Nuno Ramos, Susana Candeias Rodrigues, Catarina Rolo Santos, Ricardo São Pedro, Melissa Silva, Carla Sousa *(Hospital Garcia de Orta, Almada)*; Rui Ribeiro, Jos´é Santos, Octavio Viveiros *(Clínica de Santo António - Grupo Lusíadas, Amadora)*; Ricardo Alves, Pedro Serralheiro, Tobias Teles *(Hospital Dr. Antonio José de Almeida, Cascais)*; Liliana B. Sousa, Dulce Helena Ferreira de Carvalho Carvalho, Rui Costa Soares, Cristina Costeira, João Graveto, Daniel Martins Jordão, Filipe Paiva-Santos, Pedro Parreira, Anabela de Sousa Salgueiro Oliveira, Paulo Santos-Costa *(IPO Coimbra, Coimbra)*; Constança Azevedo, Daniela Machado, Filipa Mendes, Miguel Semião *(Centro Hospitalar Cova da Beira, Covilha)*; Joana Bolota, Ana Margarida Cinza, Manuel Damasio Cotovio, Sofia Leandro, Joana Oliveira, Rita Pedroso de Lima, Mário Pereira, Miguel Rocha Melo, Cristina Velez *(Hospital do Espirito Santo, Evora)*; Paulo Cardoso, Nicole Cardoso, Joana Cristina Domingues, Pedro Henriques, Maria Isabel Manso, Ruben Martins, Gildasio Martins dos Santos, Henrique Morais, Rute Pereira, Tatiana Revez, Vera Inês Ribeiro, Ricardo Ribeiro, Ana Soares, Sandra Sousa, João Teixeira *(Hospital Faro, Centro Hospitalar Universitario do Algarve, Faro)*; José Azevedo, Ana Cabral, Catarina S. Rodrigues *(Hospital da Horta, E.P.E., Horta)*; Paulo Alves, Gonçalo Ferreira, Rita Gonçalves Pereira, Miguel Neves, Nuno Rama, Inês Sousa *(Centro Hospitalar de Leiria, E.P.E., Leiria)*; Mafalda Almeida, Ricardo Bettencourt Morais, Ana Campos, Cristina Caroça, Mariana Couto Bártolo, Luis Galindo, Ricardo Girao, José Pais, Nelson Silva, Carlos Vaz *(Hospital CUF Infante Santo, Lisboa)*; Jose Henrique Albuquerque Messias, Francisca Brito da Silva, André Caiado, Filipa Fonseca, Sandra Maurício, Inês Peyroteo, Rodrigo Ramos, Inês Salgado, Patrícia Santos *(Instituto Português de Oncologia de Lisboa Francisco Gentil, Lisboa)*; Joana Patena Forte, Maria Luís Sacras, Ema Santos *(Centro Hospitalar Universitário Lisboa Central, Lisbon)*; Pedro Azevedo, Beatriz Costeira, Filipe Neves, Susana Ourô, Marisa Peralta Ferreira, Mafalda Sousa Fernandes *(Hospital Beatriz Angelo, Loures)*; Joana Isabel Almeida, João Almeida Pinto, Rui Almeida-Reis, Tiago Correia de Sá, Marcelo José Maia Azevedo Costa, André Dias, Vânia Fernandes, Inês Ferraz, Catarina Gil Gil, Álvaro Gonçalves, Leticia Lima da Cruz, Catarina Lima da Silva, Luciana Lopes, Nuno Machado, Joana Marialva, Margarida Nunes Coelho, José Pedro, Cristiana Pereira, Ana Ribeiro, Rui Santos, Pedro Saraiva, Rita Silva, Francisca Tavares, Maria Teixeira, Nuno Teixeira, Bernardo Teixeira, Pedro Valente *(Centro Hospitalar do Tamega e Sousa, Penafiel)*; Edgar Amorim, Odilia Conde, Miguel F. Cunha, Beatriz Dias, David Dias, Ana Fazenda, José Pedro Fernandes dos Santos, Filipe Isidro, João Pedro Melo Neves, Juan Rachadell, Inês Isabel Sampaio da Nóvoa Gomes Miguel, Liliana Sequeira *(Centro Hospitalar Universitario do Algarve - Unidade de Portimão, Portimao)*; José Davide, Eurico Emanuel do Vale Gonçalves de Castro Alves, João Oliveira, Jorge Santos, Marisa Domingues Santos, Donzília Sousa Silva, Vítor Valente *(Centro Hospitalar do Porto, Porto)*; Alexandre Almeida, Juliana Almeida Rego, Vítor Alvarenga, Duarte Nuno Amaro, Joao Barbosa-Breda, Diogo Barreiro, Pedro Cabeça Santos, Carolina Carreiro, Sara Castanheira Rodrigues, Ana Salomé Cavaleiro Leitão de Carvalho, Bernardo Correia, Francisca Costa, Vítor Devezas, Nuno Dias, Carlos Silva Faria, Sara Fernandes, Joaquim Ferreira, Fabio Gomes, Cristina Granja, Filipa Jácome, João Lixa, Ana Magalhães, Mariana Magalhães Maia, Rita Martins, Mafalda Martins Sousa, Hugo Meleiro, Margarida Mendes, João Moreira, Joana Paiva, André Pereira, António Pereira-Neves, Teresa Pina-Vaz, Gabriela Pinheiro, Sílvia Pinho, Carina Ramos, Pedro Ramos, Patricia Ramos, Margarida Ribeiro, João Rocha-Neves, Hugo Santos-Sousa, Helena Silveira, Carolina Soares-Aquino, Ana Rita Teles, Susan Vaz, Paula Vieira, Rodrigo Vilares Morgado *(Centro Hospitalar e Universitário de São João, Porto)*; Luciana Cidade Costa, Marco Santos *(Hospital da Prelada, Porto)*; Catarina Baía, Rita Canotilho, Ana Margarida Correia, Ana Paula Ferreira Pinto, Jose Flavio Videira *(IPO Porto, Porto)*; Alberto Abreu da Silva, Mariana Claro, Daniel Costa Santos, Ana Cláudia Deus, João Vaz Grilo *(Hospital do Litoral Alentejano, Santiago do Cacém)*; Carlos Boto, Rita Branquinho, Sónia Rodrigues *(Centro Hospitalar Médio Tejo, Tomar)*; Fernanda Alves, Marlene Andrade, Carlos Baltazar Branco, António Castanheira, Cátia Ferreira, Gonçalo Guidi, André Guimarães, Clara Leal, Joana Lisboa, Luís Machado, Rita Marques, Prescillia Marques, Daniela Martins, Ana Melo, Mariana Morais, Cátia Moreira, Clara Mota, Mário Moura, Sara Nunes, Antonio Oliveira, João Pedro Reis, Inês Sá, Rita Sapage, Sílvia Silva, Patrícia Sousa, Rita Sousa, Diogo Sousa, Nádia Tenreiro, Ricardo Vaz Pereira, Bruno Vieira *(Centro Hospitalar de Trás-os-Montes e Alto Douro, E.P.E., Vila Real)*; Susana Lima Oliveira, José Pinto, André Tojal *(Centro Hospitalar Tondela-Viseu, Viseu)*. |
| **Qatar**: Samir Abdalla, Soliman Alkassem, Mhamed Samer Alkhatib, Fahad Aurif, Fayez Bin Omran, Nasir Muzaffar *(Al Wakrah Hospital - HAMAD MEDICAL Corporation, Doha)*; Sheraz Abayazeed Ahmed, Abdelrahman Abdelaal, Shereen Aboutaleb, Abdelrahman Abusabeib, Ahmad Abutaka, Khalid Ahmed, Ayman Ahmed, Mahmood AL Dhaheri, Mohammed Al Dosouky, Salim Al Lahham, Mohannad Al-Tarakji, Saif Almudares, Artefaa Alshamari, Ibrahim Amer, Saif Badran, Ahmed Elaffandi, Mohamed Elakkad, Walid Elmoghazy, Mohamed Ghali, Sugad Mohamed, Muhammad Muslim, Munzir Obaid, Amjad Qabbani, Ahmed Faidh Ramzee, Mohammad Sameer, Amjad Shah, Ibnouf Sulieman, Ali Toffaha *(Hamad General Hospital, Doha)*; Siddig Abdalla, Mohamed Abdelkareem, Malik Shakeel Ahmed, Omer Al-Yahri, Mohammed Aljanabi, Hatem Kamkoum, Mohammad Burhan Khan, Mazin Khattabi, Ejaz Latif, Musab Murad, Shameel Musthafa, Iqbal rasool Wani *(Hazm Mebaireek General Hospital, Doha)*. |
| **Romania**: Rania Berrami, Arnold Marchis *(Arad Municipal Hospital, Arad)*; Mircea Hogea *(Emergency Clinical Hospital Brasov, Brasov)*; Liviu Mugurel Bosinceanu, Victor Costache, Andreea Costache, Rad Costel Claudiu, Tudor Ștefan Dumitrescu, Silviu Tiberiu Makkai-Popa, Bogdan Moldovan, George-Ovidiu Muntean, Pisica Radu-Mihai, Codin Saon, Vlad Silaghi *(Sf. Constantin Hospital, Brasov)*; Aurel Mironescu, Adrian Papa, Lucian Vida *(Spitalul Clinic De Copii Brasov, Brasov)*; Cosmin Bezede, Andrei Chitul, Florin Grama *(Coltea Clinical Hospital, Bucharest)*; Valentin Calu, Octavian Enciu, Adrian Miron, Elena Adelina Toma *(Elias Emergency Hospital, Bucharest)*; Sergiu Catalin Baraian, Cezar Ciubotaru, Bogdan Diaconescu, Florin Iordache, Alexandru Neagu, Ionut Negoi, Cristina Niculae, Bogdan Stoica *(Emergency Clinical Hospital Bucharest, Bucharest)*; Cosmin Balan, Serban Ion Bubenek Turconi, Daniela Filipescu, Raluca Goicea, Mihai Stefan, Liana Valeanu *(Emergency Institute for Cardiovascular Diseases ‘Prof. Dr. C.C. Iliescu’, Bucharest)*; Nicolae Bacalbasa, Irinel Popescu, Dima Simona *(Fundeni Clinical Institute, Bucharest)*; Vlad Alexe, Olivia Batog, Mihnea Davidescu, Madalina Iliescu, Veronica Manolache, Natalia Motas *(Institute of Oncology Prof Dr Al Trestioreanu, Bucharest)*; Sebastian Ionescu, Gabriela-Mariana Militaru, Andreea-Madalina Serban *(Maria Sklodowska Curie Emergency Hospital, Bucharest)*; Octav Ginghina, Razvan Valeriu Iosifescu, Mara Mardare, Radu Mihail Mirica, Andrada Spanu, Andrei Bogdan Văcărașu *(Saint John Emergency Hospital, Bucharest)*; Radu Costea, Lucian Negreanu, Nicoleta Aurelia Sanda, Narcis Octavian Zarnescu, Eugenia - Claudia Zarnescu *(University Emergency Hospital Bucharest, Bucharest)*; Alexandra Beuca, Ciprian Cucoreanu, Andras David, Radu Drasovean, Ioan-Alexandru Florian, Ioan Stefan Florian, Catalin Magan, Apostu Raluca-Cristina, Radu Razvan Scurtu, Andrei Trif *(Cluj-Napoca Emergency County Hospital, Cluj-Napoca)*; Eduard Acatrinei, Patriciu Achimas-Cadariu, Tareg Al Momani, Miruna Babut, Cristina Bujoreanu, Bordianu Christian, Alma-Andreea Corpodean, Sergiu Crivat, Cătălin Dumitraș, Dan Tudor Eniu, Delia Florean, Ignat Florin, Vlad Alexandru Gata, Cojocaru Ion, Alexandru Irimie, Muresan Iulia Andrada, Patricia Jako, Katinka Kallos, Gabriel Lazar, Cosmin Lisencu, Puscas Marius-Emil, Sergiu Matei, Borz Mihnea Bogdan, Ioan Milotoiu, Dragos Stefan Morariu, Maximilian Muntean, Narita Nadia Maria, Codrut Cosmin Nistor-Ciurba, Alex Orădan, Alexandru Petrusan, Bogdan Petruț, Madalina-Claudia Pocol, Irina-Maria Puscas, Alin Rancea, Dragan Serghei, Elena-Bianca Stănică, Cristina Suciu, Stefan Titu, Calin Tohatan, Evelyn Vanea, Schitcu Vlad, Ioan Catalin Vlad *(Prof Dr Ion Chiricuta Institute of Oncology, Cluj-Napoca)*; Stelian Mogoanta, Carmen-Aurelia Mogoanta, Stefan Paitici *(Spitalul Judetean De Urgenta Din Craiova, Craiova)*; Mihail-Gabriel Dimofte, Sorinel Lunca, Ana-Maria Musina, Cristian Ene Roata, Cristian Sandu, Natalia Velenciuc *(Regional institute of Oncology Iasi, Iasi)*; Drochioi Ilie Cristian, Vlad Porumb *(Spitalul Clinic de Urgență Militar Dr Iacob Czihac, Iasi)*. |
| **Russian Federation**: Mikhail Kirov, Vsevolod Kuzkov, Igor Zabaldin *(City Hospital #1, Arkhangelsk)*; Yegor Molitvin, Makhmud Ramazanov, Shamil Yuzbekov *(Engels City Clinical Hospital Number 1, Engels)*; Roman Bilenko, Veronika Budyakova, Anokhin Evgeny, George Gendrikson, Gunko Irina, Stoian Iudin, Anna Kapustina, Ilya Karpov, Mikhail Kurtenkov, Andrey Litvin, Oksana Matanova, Polina Melashenko, Artem Pobelenko, Arina Provozina *(Immanuel Kant Baltic Federal University, Regional Clinical Hospital, Kaliningrad)*; Vladimir Durleshter, Pavel Markov, Vadim Pykhteev *(Territorial hospital #2, Krasnodar)*; David Gorin, Ayrat Kaldarov, Andrey Kriger *(AV Vishnevsky Center of Surgery, Moscow)*; Alexandr Burmistrov, Eduard Galliamov, Grigoriy Gololobov, Abdula Magomedaliev, Mirzemagomed Pirakhmedov, Erin Sergey, Nazir Yurkuliev, Григорий Карсотьян, Алексей Погонин, Магомедсалам Эльдерханов *(City Clinical Hospital named SI Spasokukotsky, Moscow)*; Vladimir Balaban, Sergey Barkhatov, Yuliya Churina, Aleksandr Derinov, Sergey Efetov, Ksenia Feoktistova, Yury Kitsenko, Viktor Kochetkov, Grigory Korolev, Kirill Lazarev, Yuliya Medkova, Valery Nekoval, Pavel Pavlov, Sergey Rodimov, Irina Rudenko, Alexandra Shlomina, Darya Shlyk, Ludmila Sidorova, Petr Tsarkov, Petr Tsugulya, Inna Tulina, Victor Zhurkovskiy, Albina Zubayraeva, Ярослав Краснов *(IM Sechenov First Moscow State Medical University, Moscow)*; Mikhail Agapov, Eduard Galliamov, Tatyana Garmanova, Victor Kakotkin, Ekaterina Kazachenko, Valery Kubyshkin, Alexandr Lukianov, Daniil Markaryan, Ekaterina Semina *(Moscow Research and Educational Center, Lomonosov Moscow State University, Moscow)*; Khasan Dzhumabaev, Sergey Gordeyev, Zaman Mamedli *(N.N.Blokhin Russian Cancer Research Center, Moscow)*; Evgeniya Aleksandrova, Egine Balayan, Zinaida Galchikova, Aleksandr Gusev, Victoria E. Khoronenko, Denis Kotov, Maria Shemetova, Emin Sugaipov, Pavel Suvorin, Kirill Usachev, Vladimir Varvarin, Анна Маланова *(P. HERTSEN MOSCOW ONCOLOGY RESEARCH INSTITUTE, Moscow)*; Arkady Bedzhanyan, Mikhail Bredikhin, Eduard Charchyan, Yulia Frolova, Konstantin Petrenko, Evgenya Tyurina *(Petrovsky National Research Centre of Surgery, Moscow)*; Татьяна Македонская *(Sklifosovsky Clinical and Research Institute for Emergency Medicine, Moscow)*; Alexander Abelevich, Andrey Bazaev, Alexey Yanishev *(Privolzhsky Research Medical University, Nizhny Novgorod)*; Kiselev Nikolai, Gaik Torgomyan, Vladimir Zagainov *(Volga District Medical Centre, Nizhny Novgorod Oblast)*; Vitaly Dorofeev, Igor Panov, Oleg Perepelitsa *(Nyagan District Hospital, Nyagan)*; Kseniya Arutyunyan, Nikita Burlov, Mariia Karpenko, Gleb Khrykov, Alexey Shilyaev, Kirill Shostka, Evgeny Zagaynov, Николай Манкевич, Иван Сердюков *(Leningrad Regional Clinical Oncology Dispensary, Saint Petersburg)*; Anastasia Novikova, Oleg Ten, Aleksandr Zakharenko *(Pavlov First State Medical University of St. Petersburg, Saint Petersburg)*; Anton Akulaev, Dmitrii Buzanakov, Roman Chernikov, Maxim Chernykh, Timur Dzhumatov, Zhanna Glushchenko, Gleb Kim, Nikita Kubin, Ivan Labetov, Andrej Povalij, Dmitry Shkarupa, Dmitry Shmatov, Andrei Shulgin, Nepomnyaschaya Swetlana, Alexey Trofimow, Olga Volkova, Denis Zinkevich, Anna Zolotoukho, Дмитрий Байбуз, Элла Григорян *(Saint Petersburg State University Hospital, Saint Petersburg)*; Golomidov Alexander, Aleksandr Butyrskii *(Municipal Emegency Hospital No.6, Simferopol)*; Evgeniy Drozdov, Andrey Koshel, Nikolay Shefer *(Tomsk regional oncology hospital, Tomsk)*; Petr Chavkin, Oleg Midlenko, Saydash Shamsutdinov, Evgeny Toneev, Novgorodskii Vladimir *(Ulyanovsk regional clinical center for specialized types of medical care, Ulyanovsk)*; Abakar Abdullaev, Mahir Gachabayov *(Vladimir City Emergency Hospital, Vladimir)*; Сергей Егоров, Юрий Кудрявцев, Анна Кудрявцева, Вячеслав Тен *(Private healthcare institution ‘RZD-Medicine’, Yuzhno-Sakhalinsk)*. |
| **Rwanda**: Oscar Rwego, Isaie Sibomana *(Kibuye Referral Hospital, Karongi)*; Oda Ituze, Christophe Mpirimbanyi, Sandrine Umutesi *(Kibungo Hospital, Kibungo)*; Fidele Byiringiro, Ainhoa Costas-Chavarri, Muneza Eugene, Ngoga Eugene, Sofía Garcés Palacios, George Gasana, Afrika Guido, Charlène Habarugira Inyange, Lule Joseph, Claudia Kabanyana, Patrick Kwizera, Nzabamwita Liliane, Yves Nezerwa, Muyenzi Leon Ngeruka, Alexandre Nyirimodoka, Jean Paul Rugambwa, Daniel MUHIRE Runanira, Musore Sam, Jean Paul Shumbusho, Ian Shyaka, Alphonse Marie Sibomana, Belise Stella Uwurukundo *(Rwanda Military Hospital, Kigali)*; Jc Allen Ingabire, Dusabimana Anitha, Mediatrice Batangana, Achille Bizimana, Nyinawabagesera Blandine, Gisele Bunogerane Juru, Muhawenimana Claudine, Nyirasebura Dancilla, Vincent Dusingizimana, Lambert Dusingizimana Rutayisire, Muhawenimana Emmanuel, Basonga Emmy, Ntirenganya Faustin, Gasengayire Gilbert, Sylvie Inyange, Magnifique Irakoze, Amol Kulkarni, Sonia Mukase, Severien Muneza, Emmanuel Munyaneza, Emmanuel Mutabazi, Isaie Ncogoza, J M Solange Nkubito, Steven Nshuti, Edmond Ntaganda, Domitille Nyirahabakurama, Jeannette Nyirahabimana, Barangwa Theophile, Jean Christian Urimubabo, Mukanyange Violette *(University Teaching Hospital of Kigali (CHUK), Kigali)*; Rene Mukezamfura, Ronald Tubasiime *(Kibogora Hospital, nyamasheke)*; Immaculee Mutimamwiza, Irénée Niyongombwa, Japhet Ntezamizero *(Bushenge Provincial Hospital, Rubavu, Nyamasheke)*. |
| **Saudi Arabia**: Mushabab Alshahrani, Nasir Magboul, Ahmed Mangahy *(aseer central hospital, abha)*; Abdulaziz Alghamdi, Abdulrahman Almutawa, Fahad Alqahtani *(King Abdulaziz National Guard Hospital, Al-Ahsa)*; AbdulAziz Al Hindi, Abdullah Albarrak, Abdullah Almunifi, Saad M. Alqahtani, Afnan Alsultan, Ahmed Alzahrani, Nasser Alzerwi, AlaELDEIN Elnaema, Musaed Rayzah *(King Khalid General Hospital, Al-Majmaah)*; Reda Abd ElGhany, Ahmed Al Ameer, Dauda Bawa, Mohammed Elzain, Fadi Wakill *(King Abdullah Hospital, Bisha)*; Abdulrahman Almuawi, Omar Mohamed Alsamahy, Mir Fahiem-ul-Hassan *(Maternity and Children’s Hospital, Bisha)*; Mohammed Al Duhileb, Hussain Al Jawad, Emad Al-Absi, Huda Alawami, Ghaleb Alazzeh, Amirah Aldhurais, Fozan Aldulaijan, Susan Alghamdi, Thabet Alghazal, Zahrah Alhajji, Abdulrahman Alkhatib, Nora Almana, Ashraf Almatar, Bikheet Almatar, Manal Alnaimi, Hadeel AlOmran, Sarah Alrubaish, Turki Alshammari, Munir Alsuwaimel, Abdullah Alwabari, Ali Alzahir, Abdulaziz Babaier, Ameera Balhareth, Sara Kanfar, Tariq Madkhali, Mumtaz Sarang, Tawfeeq Tawfeeq *(King Fahad Specialist Hospital, Dammam)*; Bader Alburayh, Ahmed Alkhalifah, Abddulrahman Almulhim *(King Fahad Hospital Hofuf, Hofuf)*; Malak Abutaleb, Mohammed Ageeli, Ehab Alameer, Nuraddin Alhakami, Barrag Alhazmi, Saud Alkhayrat, Saeed Almalki, Norah Durayb, Salman Ghazwani, Riyadh Hakami, Mohammed Miftah, Sameer Otayfah, AbdulRahman Sari, Momen Shawk *(King Fahd Central Hospital, Jazan University, jazan)*; Abdu Ayoub, Awsam Hakami, Hesham Hamaly *(Prince Mohd Bin Nassir Hospital, Jazan)*; Awaji Al nami, Hussain Alqaser, Mohammed Alsadiq, Orkia Belhadri, Mohammed Fouad, Othman Iskander, Hazem Khatab, Mohammed Othman, Mohammed Rida *(Sabia General Hospital, Jazan)*; Samer Al Athath, Mohammed Alharthi, Ameen Alherabi, Basmah Altuwayjiri, Khalid Bakier Mohammed, Muhannad Bayazid, Walid Bukhari, Mohammed Ghunaim *(International Medical Center, Jeddah)*; Mohammad Monir Abbas, Abdullah Abdullah, Bassam Addas, Nouf Akeel, Osman Al-Radi, Sonds Al-Shammakh, Ibtihal Alghamdi, Amani Alhaddad, Mohammed Alharthi, Murad Aljiffry, Alia Aljifri, Adil Aljohari, Maram Alkhatieb, Soha Alomar, Samir Alsulaimani, Abdulmalik Altaf, Mohammed Alyousef, Fatima Arab, Kholoud Awaji, Saleh Baeesa, Khalid Bajunaid, Mohammed Bangash, Mohammed Basendowah, Nasir Bustangi, Sara Farsi, Deema Farsi, Nada Farsi, Ali Farsi, Mohammed Ghunaim, Alaa Habeebullah, Ahmad Khoja, Ashraf Maghrabi, Nadim Malibary, Ziad Malibary, Mohammed Nassif, Anfal Nawawi, Abdulrahman J Sabbagh, Salma Sait, Abdulaziz Saleem, Ali Samkari, Hanaa Tashkandi, Nora Trabulsi *(King Abdulaziz University Hospital, Jeddah)*; Ahmed Alawi, Abdulaziz Alghamdi, Bassam Altalhi, Turki M Alzaidi, Heba Jaloun, Samiullah Mughal, Ahmed Organjee, Mohammed Rashwan, Abdulmajeed Abdulaziz Saeedi, Mustafa Samadony, Mohamed Shalaby, Mazen Zidan *(King Fahad Armed Forces Hospital, Jeddah)*; Mohammed Algarni, Majed Almuraee, Mubarak Alshahrani, Awwadh Althobaiti, Azah Althumairi, Ali Alyami, Alwaleed Alyami, Mohammed A Alzahrani, Hadi Hakami *(King Khalid National Guard Hospital, Jeddah)*; Ahmed Abd Elwahab, Abouelnour Abdelbaset, Nabil Abdulqawi, Abdullah Abdulrahem, Abdullah Abubakar, Mohammed Ahmed, Hessa Al Habes, Mohammed Al Jamahir, Rashed Al Qudhaya, Hajr Al Wadei, Ali Al-Qannass, Qusay Al-Qurashi, Mohammed Al-Urfan, Samer Alammari, Abdulbari Alawadhi, Adel Albaiti, Ibrahim Albakry, Maher Alkahal, Samer Alkarak, Omar Alkhanbashi, Mashhour Alqannas, Abdullah Alqattan, Murtagi Alraboui, Yasir Alsagoor, Mazen Alsakkaf, Nasser Alsharif, Mohammad Alyami, Hamad Alyami, Mohammed Alzamanan, Mahdi Alzamanan, Delia Cortés-Guiral, John Spencer Daniels, Amar Elawad, Said Elsagheer, Mohammed Fagihi, Mana Hajlan, Emad Hamdy, Mohammad Ibrahim, Safa Mahmoud, Taher Mahnashi, Atef Mansour, Haider Mohamed, Rawabi Mohammed, Mayad Moktash, Mohammad Mousa, Hussain Munyif, Hamoud Obied, Mohammed Samara, Tariq Azam Siddiqi, Youssef Taha *(King Khalid Hospital, Najran)*; Abdulrahman Al Amri *(Najran university hospital, Najran)*; Ghaleb Aboalsamh, Ohood AlAamer, Abeer Alaglan, Salman Alahmed, Khalid Alaqeely, Ali Albargawi, Abdullah A. Alghamdi, Sultan Alhabdan, Fahad Alhelal, Hani Alkattan, Mohammed Alnamshan, Mohammed Alnasser, Lolwah Alriyees, Meaad Alromaihi, Norah Alsabty, Maryam Alsafi, Jawaher Alsahabi, Nahar Alselaim, Azah Althumairi, Hassan Arishi, Manerh Bin Mosa, Khalid Bin Saad, Ahmed Binjaloud, Sarah Breakeit, Hattan Dagestani, Abdullah Eissa, Basmah Ghallab, Azza Madkhali, Arwa Mahfouz *(King Abdulaziz Medical City, Riyadh)*; Muath Abaalkhail, Muhammad Abukhater, Mehjabeen Adenwalla, Ghiath Al Saied, Yousof Alabdulkarim, Ahmed Alamri, Mohammed Alateeq, Majed Albarrak, Naif Aldhaam, Abdullah AlFakhri, Mohammad Alfarah, Ahmad Alghamdi, Bandar Alharthi, Mofarej Alhogbani, Aisha Mansoor Ali, Mohammed Alkahlan, Raed Almannie, Mohammed Almatrafi, Khalid Almohaimeed, Eman Almotairi, Fatema Almushawah, Hisham Almutawa, Suliman Alobaid, Loai Alqahtani, Saad Alqasem, Shahad Alqreen, Mohammed AlRayih, Mohammed Alreshidan, Khaled Alsaleh, Alanoud Alsamari, Nouf Alshammari, Abdulaziz Alshammari, Zeyad AlSolami, Fahd AlSubaie, Youssuf AlSuhaibani, Yazeed Alsuliman, Saleh Alsuwaydani, Abdulrahman Alturki, Saud Alzahrani, Gmaan Alzhrani, Mohammad Asiri, Tehmina Aziz, Mohammed Bafaquh, Maher Moazin, Marah Nadreen *(King Fahad Medical City, Riyadh)*; Ahmed Abu-Zaid, Mohammad Aburahmah, Lama Al Humaid, Aminah Al Nafesa, Saleh Al Nassar, Faisal Al Otaibi, Ismail A. Al-Badawi, Mohammad Al-Qattan, Amira Alabbasi, Hani Alahdal, Ibrahim Alahmadi, Ahmed Alahmari, Sara Alaqel, Ali Alassiri, Nof Albawardy, Khalid Aldaghiri, Homoud AlDahash, Maryam ALeissa, Dr. Saad Algarni, Ammar Alhaidari, Othman Alhammad, Naif Alhathal, Alaa Alhazmi, Norah Alhazzaa, Amal Alhefdhi, Boshra Alhelal, Meshari Alhuthayl, Abdullah Alkassim, Wafa Alkhayal, Ali Almalaq, Osama Almalik, Felwa AlMarshad, Meshal Almeshal, Razan Almesned, Hadi Almohsen, Hanan Almutairi, Alanoud Alomair, Osama Alomar, Faris Alomran, Naif H Alotaibi, Ahmed AlOtaibi, Moraya Alqahtani, Abdulmajeed AlQahtani, Saad Alqarni, Reem AlRakaf, Fay Alresaini, Omar Alrifai, Musab Alsakka, Ree,m Alsalamah, Yazeed Alsebayel, Muhannad Alsemari, Jaffar Alshahri, Saud Alshanafey, Khalid Alshehri, Yasir AlShehri, Saif Alsobhi, Thuraya AlSumai, Assma Altaher, Ibrahim Althubaiti, Abdullah Alzahrani, Muhammad Ansary, Ibrahim Asiri, Hizabr Elsheikh, Faisal Farrash, Zakaria Habib, Fuad Hashem, Maher Hassounah, Attiya Ijaz, Abdulaziz Jarman, Dana Kalagi, Wafa Khudier, Samer Koussayer, Nehal Mahabbat, Sabreen Maqbol, Mohamed Amir Mrad, Wessal Otaif, Eyas Othman, Rajeev Pant, Atif Rafique, Syed Raza, Bashayer Saeed, Hani Salem, Qutaiba Shah Mardan, Peter Spangenberg, Maha Tulbah *(King Faisal Specialist Hospital, Riyadh)*; Nadia Ajomah, Abdullah Albdah, Rami Alghamdi, Abdullah Almufarrih, AlHanouf Alsaloom, Norah Alsubaie, Sharfuddin Chowdhury, Kareem ElSanhoury, Mohammed Koumu, Abu Talha Siddiqui *(King Saud Medical City, Riyadh)*; Amal Abdulkareem, Kaleem Ahmed, Abdulrazag Ajlan, Khalid Akkour, Sultan A Al Amri, Rehab Al Moagal, Amro Al-Habib, Hazem Al-Mandeel, Ghadeer Al-Shaikh, Adel Alahaidib, Mohammed Alali, Ossama Alamri, Abdullah Alatar, Khalil Alawi, Nada Alayed, Ahmed Alburakan, Lateefa Aldakhyel, Abdulrahman Aldakkan, M Zahir Aldalati, Wassim Aldebeyan, Abdallah Alferdaus, Doaa Alfraidy, Lolowah Alghuson, Hani Alhalal, Basmah Alhassan, Fawzi Aljassir, Abdullah Aljunaydil, Abdulaziz Aljurayyan, AbdulAziz AlKanhal, Waleed H. Alkhamis, Abdulaziz Almaawi, Ahmad Almalki, Raed Almannie, Abdullah Almousa, Abdulaziz Alomar, Jalal Alowais, Awadh Alqahtani, Abdulaziz Alsaif, Nuha Alsaleh, Hisham Alsanawi, Sulaiman Alshammari, Ibrahim Alshaygy, Abdulmajeed Altoijry, Talal Altuwaijri, Ikhlass Altwejri, Nasser Alwehaibi, Reem Alyahya, Meshari Alzahrani, Abduljabbar Alzuhair, Khalid Arab, Zeneb Babay, Ali Bassi, Ahmad Bin Nasser, Areej Bokhari, Sherif Elwatidy, Hadeel Helmi, Abdullah Kattan, Saad Khashogji, Nayef Mansour Alshammari, Rafif Mattar, Hatan Mortada, Bushr Mrad, Thamer Nouh, Yasser Sabr, Mishary Shalhoub *(King Saud University, Riyadh)*; Turki Alajmi, Deem Alakeel, Rana Alhossaini, Ali Aljuzair, Muath Alrashed, Tareq Alsabahi, Ameen Alshehri, Talal Altahan, Mohammad Altuwaijri, Muath Alwabel, Mohamed Aly, Mosa Alzahrani, Adel Assiri, Ahmed Bakhsh, Mohammed Sbaih *(Prince Mohammed bin Abdulaziz Hospital, Riyadh)*; Jubran Al Faifi *(Riyadh Care Hospital, Riyadh)*; Shehanah Al Omair, Saad Alobaysi, Saad Alobaysi, Khalid Beidas, Nawaf Modahi, Norah Musallam *(Security Forces Hospital, Riyadh)*; Khaled El Gazzar, Khaled Ghabban, Ahmed Abdulmohsen, Hussam Adi, Amgad Afifi, Khaled AI Nwijy, Turki AI Zahrani, Jehad Akiely, Abdulaziz Al Harthi, Attiya Al zahrani, Feras Alahmad, Dr Yousef Alalawi, Dr. Ahmad Alayed, Abdallah Alghazo, Nawaf Alharthi, Sultan Alharthi, Mohammad Alnoaiji, Bandar Alqahtani, Abdulellah Alqarni, Noorsabah Alrubays, Mohammed Alshehri, Abdulmajeed AlShehri, Mamdouh Alzaibak, Majed Attoun Attoun, Ahmed Awaji, Yaquob Daghriri, Reda Daoud, Aymad Eltawab, Sherif Emara, Ayman Farouk, Anas Fatani, Mojahid Ghazi, Talal Khewater, Elsayed Monier, Nizar Musawa, Dr .Magdy S.El-Bahnasawy, Mahmoud Zakaria *(King Salman Armed Forces Hospital, Tabuk)*; Saleh ALghamdi, Waleed Althobaiti, Selmy Awad *(King Faisal Medical Complex, Taif City)*. |
| **Senegal**: Ibrahima Konate, Abdourahmane Ndong, Jacques Tendeng *(Saint-Louis Regional Hospital, Saint-Louis)*. |
| **Serbia**: Ivan Paunovic, Nikola Slijepcevic *(Centre for endocrine surgery, Clinical Centre of Serbia, Belgrade)*; Nouf Alajaji, Lidija Aleksić, Goran Barisic, Aleksandar Bogdanovic, Miljan Ceranic, Ivan Dimitrijevic, Keramatollah Ebrahimi, Daniel Galun, Željko Grubač, Nikola Grubor, Nenad Ivanović, Jelenko Jelenkovic, Stefan Kmezić, Djordje Knezevic, Zoran Krivokapic, Stojan Latinčić, Velimir Markovic, Slavko Matić, Marko Miladinov, Aleksandar Ninic, Maja Pavlov, Ivana Pavlovic, Ilija Pejovic, Dejan Radenkovic, Zeljko Radojkovic, Slobodan Rašić, Milorad Reljic, Predrag Sabljak, Aleksandar Sekulić, Vladimir Šljukić, Boris Tadic, Jovica Vasljević, Dejan Veličković, Marko Zivanovic *(Clinic for Digestive Surgery, Clinical Centre of Serbia, Belgrade)*; Pavle Gregoric, Zlatibor Loncar, Dusan Micic *(Clinic for emergency surgery, Emergency centre, Clinical centre of Serbia, Belgrade)*; Drago Jelovac, Srbislav Pajić, Milan Petrovic, Sanela Sumrak *(Clinic for Maxillofacial Surgery, School of Dental Medicine, University of Belgrade, Belgrade)*; Vladimir Bascarevic, Ivan Bogdanovic, Danica Grujičić, Rosanda Ilic, Miloš Joković, Mihailo Milićević, Filip Milisavljević, Aleksandar Miljković, Aleksandra Paunovic, Vuk Šćepanović, Aleksandar Stanimirovic, Marko Todorovic *(Clinic for Neurosurgery, Clinical Center of Serbia, Belgrade)*; Miljan Folic, Ana Jotic, Sanja Krejovic Trivic, Jovica Milovanovic, Aleksandar Trivic *(Clinic for Otorhinolaryngology and Maxillofacial Surgery, Clinical Center of Serbia, Belgrade)*; Lazar Davidovic, Stefan Ducic, Igor Koncar *(Clinic for Vascular and Endovascular Surgery, Serbian Clinical Center, Belgrade)*; Uros Bumbasirevic, Zoran Dzamic, Boris Kajmaković, Bogomir Milojevic, Nebojša Prijović, Marko Zivkovic *(Clinic of Urology, Clinical Center of Serbia, Belgrade)*; Aleksandar Lazic, Nebojsa Mitrovic, Dejan Stevanovic *(Clnical hospital centre of Zemun, Belgrade)*; Marko Buta, Ana Cvetkovic, Igor Djurisic, Merima Goran, Zorka Inic, Andjela Ivezić, Nikola Jeftic, Marko Jevric, Vladimir Jokic, Milan Kocic, Zoran Kozomara, Ivan Markovic, Srdjan Nikolic, Nada Santrac, Nevena Savković, Igor Spurnic, Dobrica Stevic, Dejan Stojiljkovic, Jovana Tripkovic, Nikola Vucic, Milan Zegarac *(Institute for Oncology and Radiology of Serbia, Belgrade)*; Tatjana Dejanovic, Vladimir Djukic, Srdjan Marinković, Miljan Milanovic, Nemanja Pijanovic, Milena Radosavljevic, Predrag Savic, Dejan Stojakov, Rastko Živić *(KBC Dr Dragisa Misovic-Dedinje, Belgrade)*; Sinisa Ducic, Mikan Lazovic *(University Children’s Hospital, Belgrade)*; Jovana Bojičić, Vladica Cuk, Jovan Juloski, Aleksandar Karamarkovic, Marko Kenic, Bojan Kovacevic, Igor Krdzic, Vladan Milutinović *(Zvezdara University Medical Center, Belgrade)*; Vladimir Djan, Velicko Trajkovic, Dragana Zivkovic *(Institute for Child and youth Health Care of Vojvodina, Novi Sad)*; Aleksandar Đermanović, Danica Golijanin, Dejan Lukic, Zoran Radovanovic, Dragana Radovanovic, Sanja Zahorjanski *(Oncology Institute of Vojvodina, Sremska Kamenica, Novi Sad)*. |
| **Singapore**: Adrian Chiow, Jun Xian Hing, Lip Seng Lee, Sey Kiat Terence Lim, Chi Wei Mok, James Ngu, Su-Ming Tan, Hiang Jin Tan, Nan Zun Teo *(Changi General Hospital, Singapore)*; Hana Arbab, Candy Choo, Doris Mae Dimatatac, Yee Low *(KK Women’s and Children’s Hospital, Singapore)*; Min Hoe Chew, Hui Wen Chua, Tousif Kabir, Frederick Koh, Sabrina Ngaserin, Lester Ong, Kiat Tee Benita Tan, Alvin Tan *(Sengkang General Hospital, Singapore)*; Wei-Wen Ang, Yew-Lam Chong, Jeffrey J Leow, Biquan Liu, Ming Ngan Aloysius Tan, Ern Yu Tan, Kar Yong Wong, Sharon Yeo *(Tan Tock Seng Hospital, Singapore)*. |
| **Slovak Republic**: Ladislav Czako, Branislav Gális, Arpád Panyko, Kristián Šimko *(University Hospital Bratislava, Bratislava)*; Radoslav Morochovič, Martin Paulo, Marián Sedlák *(L. Pasteur University Hospital, Košice)*. |
| **Slovenia**: Jan Grosek, Jurij Aleš Košir, Ales Tomazic *(University Medical Centre, Ljubljana)*; Leyla Al Mahdawi, Nuhi Arslani, Andrej Avsenak, Uros Bele, Andrej Bergauer, Krešimir Blažević, Dejan Bratus, Dalibor Cankoski, Andrej Cokan, Bojana Crnobrnja, Andraz Dovnik, Vojko Flis, Samo Fokter, Urska Gajsek, Minja Gregorič, Marko Hazabent, Tomaz Jagric, Niko Kavčič, Jure Knez, Nina Kobilica, Rok Kovačič, Lara Lukman, Urska Marolt, Jan Mlakar, Tamara Mohorko, Andrej Moličnik, Ivan Perić, Stojan Potrč, Milena Senica Verbic, Mateja Sirše, Jerneja Vidmar *(University Medical Centre, Maribor)*. |
| **Somalia**: Adnan Sayid Abdo, Abdifatah Dahir Ali, Sabra Aqil, Hassan Daoud, Abdilaahi Ahmed Hayir, Sadiq Ibrahim, Afnan Nuh, Kadra Omar, Ridwan Saeed *(Hargeisa Group Hospital, Hargeisa)*. |
| **South Africa**: Nikita Blake, Cassandra Ferreira, Corne Nel, Colin Noel *(Pelenomi Hospital, Bloemfontein)*; Nikita Blake, Marcel de Kock, Cassandra Ferreira, Nathan Kaplan, Emile Kayombo, Paballo Clinton Khaeane, Asha Malan, Wikus Wessel Mulder, Corne Nel, Colin Noel, Daylen Rutledge, Cornel Steyn, Gert Steyn *(Universitas Academic Hospital Complex, Bloemfontein)*; Naser Almgla, Mohamed Daoub, Hammaad Gamieldien, Thomas Hilton, Nazmie Kariem, Maahir Kariem, Christo Kloppers, Maritz Laubscher, Wanga Mtimkulu, Walid Mugla, Daniel Nel *(Groote Schuur Hospital, Cape Town)*; Ferhana Gool *(Mitchell’s Plain District Hospital, Cape Town)*; Timothy Hardcastle, Wasim Mahomed, Nivashen Pillay *(Inkosi Albert Luthuli Central Hospital, Durban)*; Kayla Rossini, Stephanie Van Straten *(Estcourt District Hospital, Estcourt, Kwa-Zulu Natal)*; Annamart Laubscher, Adelaide Lee, Colin Noel, Mohammed Yusuf Parker, Sinead Quirke *(Kimberley Provincial Hospital, Kimberley)*; Therese Du Preez, Adeline Jacobs, Reinier Swart *(Shelly Beach Hospital, Margate)*; Modise Zacharia Koto, Neha Kumar, Imraan Sardiwalla *(Dr George Mukhari Academic Hospital, Pretoria)*. |
| **South Sudan**: Mapuor Areu *(Juba Teaching Hospital, Juba)*. |
| **Spain**: Cristina Barrena López, Juan Campos Garcia, Paula Ferrara, Christoph José Klein Zampaña, Raquel Lopez, Hernan Sandoval *(Complejo hospitalario universitario de Albacete, Albacete)*; Guillermo Carreño, German Mínguez Ruiz, Antonio Rodriguez Infante *(San Agustín University Hospital, Avilés)*; Conrado Andrés Ros, Raquel Angulo Artal, Luis Barbier, Patricia Caja Vivancos, Guillermo García-Operé, Marta Jiménez-Jiménez, Héctor Marín, Mikel Oñate, Luis Antonio Pascua-Gómez, María Alexandra Pesántez Peralta, Mikel Prieto, Ibabe Villalabeitia Ateca *(Hospital Universitario Cruces, Barakaldo)*; Igor Alberdi San Roman *(San Eloy Hospital, Barakaldo)*; Ladislao Cayetano Paniagua, Laura Gomez Fernandez *(Consorci Sanitari de Terrassa, Barcelona)*; Joan Camí, Pablo Collera, Martínez David, Carlos Javier Gómez Díaz, Josep Maria Muñoz Vives, Jordi Querolt Coll, M.dolors Rosines Cubells, Cristina Soto Montesinos, Guillermo Triana, Marina Xicola *(Fundació Althaia - Xarxa Assistencial Universitària de Manresa, Barcelona)*; Alfonso Alias, Albert Baduell, Mariano Balaguer-Castro, María Pilar Camacho Carrasco, Marina Cámara Vallejo, Borja Campuzano Bitterling, Anna Carreras-Castañer, Guillem Claret, Francisco J. Cuesta-González, Alberto Di Somma, Joaquim Enseñat Nora, Neus Fabregas, Ada Ferrer Fuertes, Abel Ferrés, Cesar Ginesta, Jose Juan Gonzalez Sanchez, Isabel Gracia, Jhon Alexander Hoyos Castro, Montsant Jornet-Gibert, Xavier Morales, Alejandra Mosteiro-Cadaval, Leire Pedrosa, José Poblete Carrizo, Marina Renau-Cerrillo, Luis Alberto Reyes Figueroa, Pedro Roldan Ramos, Jordi Rumià Arboix, Marta Sabater-Martos, Josep M. Segur, Ramon Sieira-Gil, Thomaz E. Topczewski, Jorge Torales, Ramon Torné, Pere Torner, Victor Turrado-Rodriguez, Ricard Valero, Marian Vives-Barquiel *(Hospital Clinic Barcelona, Barcelona)*; Eulalia Ballester, Alberto Basterra Rincon, Vitiello Giulia, Belén Martin Arnau, Anna Sánchez López, Santiago Sánchez-Cabús *(Hospital de la Santa Creu i Sant Pau, Barcelona)*; Marta Gil, Silvia Martin, Monica Serrano-Navidad *(Hospital de Viladecans, Barcelona)*; Mauricio Agüero Mariño, Núria Casanova Torrequebrada, Berta Fabregó Capdevila, Marta Jimenez Toscano, Adrián López Campillo, Gemma Mancebo, Ester Miralpeix, Blanca Montcusí, Marina Munarriz, Ariadna Salvadó, Josep M Sole-Sedeno, Clara Téllez Marquès *(Hospital del Mar, Barcelona)*; Ana Centeno Álvarez, Ruth Lopez Gonzalez, Luisana Riba Combatti, Andrea Sanz Llorente *(Hospital Moisès Broggi (Consorci Sanitari Integral), Barcelona)*; Miguel Bejarano Serrano, MªPilar Canals Sin, Blanca Capdevila Vilaró, Maria Coronas Soucheiron, Mario Cuesta Argos, Irene de Haro Jorge, Yaiza Galvañ Félix, Luis García-Aparicio, Saura García Laura, Rebeca Leal, Albert Malet Contreras, Oriol Martin Sole, Irene Martínez-Padilla, Clara Massaguer, Ines Moraleda Gudayol, María Elena Muñoz Fernández, Pedro Palazon Bellver, Paco Parri, Cristina Pérez Costoya, Sonia Pérez-Bertólez, Jordi Prat-Ortells, Mireia Riba Martínez, Javier Rojas-Ticona, Josep Rubio-Palau, Leopoldo Tapia Moral, Xavier Tarrado, Francisco Vicario *(Hospital Sant Joan de Deu, Barcelona)*; Francesc Angles Crespo, David Bosch Garcia, Berta Escudero, Carlos Garcia Cardona, Jorge Nuñez *(Hospital Universitario Mutua de Terrassa, Barcelona)*; Sergi Bellmunt-Montoya, Javier de la Torre, Úrsula Acosta, Veronica Alonso Mendoza, Iago Alvarez Saez, Eva Andreu Riobello, Fernando Ascanio Gosling, Jose Bagan, Coro Bescós, Ruth Blanco-Colino, Irene Brana, Bartomeu Caimari, Marta Cicuendez López Ocaña, Anna Conesa, Alba de Pablo García-Cuenca, Natalija Dediulia, Francesc Duran-Valles, Eloy Espin-Basany, Martin Espinosa-Bravo, Daniel Gil-Sala, Jordi Giralt López de Sagredo, Maria Jose Gomez-Jurado, Susana González-Suárez, Ernesto Guerra-Farfan, Juan sebastian Guillén, María Guisasola Rabés, Viviana Andrea Hernández Angel, Maria Jurado Ruiz, Yuri Loaiza, Manuel Lopez, Susana Manrique, Maria Dolores Mateo Arzo, Joan Minguell-Monyart, Enric Miret Alomar, Sonia Monreal Clua, Nuria Montferrer Estruch, Víctor Morales Ariza, Clara Morales Comas, Judith Murillo, Jose Maria Nieto Rodriguez, Jorge Nuñez, Elena Vilardell Ortiz, Jorge Pamias, Montserrat Pascual Pascual Arellano, Remei Perera Sarri, Aleix Pons Bartroli, Lucia Porteiro Mariño, Nil Prat, Alfredo Pueyo Ferrer, Rosa Pujol Pina, Jordi Raurich-Leandro, Maria José Reche Padilla, Joaquín Rivero Déniz, Natalia Rodriguez, Ana Rodríguez-Tesouro, Fabian Rojas Portilla, Manuel Saez barba, Vanessa Sanchez Torrents, Berta Serrano, María Sol Siliato Robles, Christian Siso Raber, Iñigo Soler, Maria Carmen Suescun López, Jordi Teixidor Serra, Cristina Tello-Díaz, M Pilar Tormos Pérez, Marta Utrilla Pérez, Ramon Vilallonga, Inmaculada Vives, Irene Vives Roselló *(Vall d’Hebron University Hospital, Barcelona)*; Vázquez García César, Mireia Gili-Bueno, Carlos Gomez Roig, Gloria González, Aamer Malik, Maria del Mar Martí-Ejarque, Mario Rafael Medina Hernández, Carolina Pozo, Laura Ruiz-Villa, Nuria Sierra, Antonio Valle, Susana Velasquez *(Hospital Universitari Sagrat Cor, BARCELONA)*; Mirentxu Arrieta, Daniel Escobar, Unai Garcia De Cortazar, Arkaitz Lara, Alicia Rojo, Kiara Tudela, Idoia Villamor, Javier Aitor Zabala Lopez-Maturana *(Hospital Universitario de Basurto, Bilbao)*; Begoña Estraviz, Laura Fernández Gómez Cruzado, Melania González de Miguel, Aitor Landaluce-Olavarria, David Lecumberri *(Hospital Urduliz, Bizkaia)*; Victor luis Gomez corujo *(La Palma, Breña Alta)*; Silvia Bleda, Joaquin De Haro *(Hospital Los Mandronos, Brunete, Madrid)*; Mercedes Estaire Gómez, Carlos Martínez-Pinedo, David Padilla-Valverde, Rafael Picón Rodríguez, Francisco Javier Redondo Calvo, Daniel Sanchez-Pelaez *(Hospital General Universitario de Ciudad Real, Ciudad Real)*; Alfonso Espinosa Ruiz, José Manuel Morales-Puebla, Eduardo Rodriguez *(Hospital Quironsalud Ciudad Real, Ciudad Real)*; Ignacio Alcalá Rueda, Gonzalo Díaz Tapia, Alvaro Sanchez Barrueco *(Hospital Universitario General de Villalba, Collado Villalba, Madrid)*; Maria Dieguez López, Isabel López García, Pilar López-Toribio López *(Hospital Reina Sofia, Cordoba)*; Marina Bosch, Carolina Curtis Martínez, Alba Fernández Candela, Sandra Lario Pérez, Cristina Lillo García, Clara López de Lerma Martínez de Carneros, Francisco López Rodríguez -Arias, Inmaculada Oller, Saray Quinto, Luis Sánchez-Guillén, Antonio Sanchís López, Álvaro Soler-Silva, Daniel Triguero Cánovas *(Hospital General Universitario de Elche, Elche)*; Victoria Garcia Peces, Estefania Hernández-García, Guillermo Plaza *(Hospital Universitario de Fuenlabrada, Fuenlabrada)*; Rafael Abad Alonso, Francisca Aranda Lozano, Marta Calvo Fernández, Eneko Del Pozo Andres, Josune Etxabe Gurrutxaga, Francisco Javier Fernández Pablos, Francisco Javier Ibáñez-Aguirre, Begoña Ochoa Villalabeitia, Natalia Ortega, Amaia Sanz Larrainzar, Bakarne Ugarte-Sierra, Irune Vicente Rodriguez *(Hospital Universitario de Galdakao, Galdakao-Usansolo)*; Virginia Jimenez, Javier Jimenez Miramón, Jose M Jover, Tamara Llamero, Jose Luis Ramos Rodriguez, Ainhoa Valle Rubio *(Getafe University Hospital, Getafe)*; Maria Teresa Albiol, Codina Antonio, Marta Bertrand, Clara Codony, Olga Delisau-Puig, Ramon Farres Coll, Cristina Farrés Pla, Jorge Garcia-Adamez, Nuria Gomez Romeu, David Julià Bergkvist, Santiago Lopez-Ben, Eloy Maldonado-Marcos, Marcel Pujadas, Berta Tió, Francesc Tuca *(Hospital Universitari de Girona Dr. Josep Trueta, Girona)*; Elizabeth Bárcena, Esther Brea Gómez, Antonio Francisco Guisado Calderón *(Hospital Universitario Virgen de las Nieves, Granada)*; Elisabeth Fernandez Castro, Montserrat Mairal Fraile, Oscar Pastor *(Hospital General de Granollers, Granollers)*; Lucia Diego García, Cristina García Amador, Raquel Latorre Fragua *(Hospital Universitario de Guadalajara, Guadalajara)*; Marta Allue, Pablo Colsa, Teresa Gimenez Maurel, Raúl Latorre Tomey, Luis Francisco Martín Anoro, Luca Ponchietti, Marta Roldón Golet, Alejandra Utrilla Fornals *(Hospital General San Jorge, Huesca)*; Carlo Brugiotti, Carlos Jiménez Viñas, Fátima Sena-Ruiz *(Hospital Comarcal d’Inca, Inca, Mallora)*; Julio Plata-Bello *(Hospital Universitario de Canarias, La Laguna)*; Octavio Arencibia, Daniel Gonzalez Garcia-Cano, Maria Laseca, Antonio Navarro-Sánchez, Iván Soto-Darias *(Complejo Hospitalario Universitario Insular-Materno Infantil, Las Palmas de Gran Canaria)*; David Ortiz López, Maria Pelloni, Aida Cristina Rahy-Martín *(Hospital Universitario de Gran Canaria Doctor Negrín, Las Palmas de Gran Canaria)*; Sara Berrocal, Elima Pilar Cagigal Ortega, Jorge Calvera, Iria Cervera, Patricia Díaz Peña, Silvia Verónica Domínguez Ovejas, Francisco Donis, Diego Enjuto, Patricia Fernández Bernabé, Gema Fraga, Raúl Garcés García, Antonio García, Elena Garcia De Castro, Antonio García Domínguez, Miguel Angel García García, Judit Gonzalez, Carles Heredia Llinàs, Inés Hernández, Norberto Herrera Merino, Carmen Lucero León Gámez, Ana Luis Siles, Maria Marqueta De Salas, Paula Martinez Pascual, Almudena Ortiz Simón, Laura Osuna, Marta Perez Gonzalez, Antonio Ramos Bonilla, Adolfo Ramos-Luengo, Lorena Rodríguez Gómez, Jose Luis Uquillas *(Severo Ochoa University Hospital, Leganés)*; Miguel Angel Alonso Prieto, Marta Ballesteros-Pomar, Sara Busto Suarez, Mario Castaño, Laura Castillo Pardo, Mario De Arriba Alonso, Fernando Diez Burón, Anna Farrés Rabanal, Diego Fernández-Samos Fernández, David Garcia, Cristina García Pérez, Miguel García Sanz, Cesar Gracia, Javier Gualis, Gonzalo Gutiérrez Carrillo, Mateo Hevia, Eva Higuera Miguélez, Gregorio Laguna, Cristina Lombardia Gonzalez de Lera, Pablo López Martínez, Pasquale Maiorano, Sergio Marcos Contreras, Elio Martín Gutiérrez, Marta Molina, Irene Perez, Teresa Renedo Villar, Luis Angel Suarez Gonzalez *(Complejo Asistencial Universitario de León, León)*; Eva Alvarez Torres, Luz Bueno Rey, Zulay Adriana Calderon Barajas, Ana M Castaño-Leon, Juan Delgado Fernandez, Aida Fernández, Pablo Garcia Pimentel, Luis Jimenez-Roldan, Meta Levstek, Carolina Lugo Duarte, Rosalia Navarro Casado, Claudia Olea Vielba, Ana Maria Parada Rodriguez, Igor Paredes, Patricia Pascual-Cambero, Miguel Saiz Sánchez-Buitrago, Claudia Sarrais Polo, Victor Zarza Fernandez de Alegria *(12 de Octubre University Hospital, Madrid)*; Mireya Bonet, Alberto Encinas Vicente, Jose Miguel Villacampa Auba *(Fundación Jimenez Diaz University Hospital, Madrid)*; Maria Dolores Burgueño, Hanna Perez-Chrzanowska, Patricia Serrano Méndez *(Hospital Cantoblanco, Madrid)*; Thomas W. Jorgensen, Lucila Marquez, Mercedes Martin *(Hospital Central de la Cruz Roja San Jose y Santa Adela, Madrid)*; Diana Crego Vita, Mónica Huecas, Felipe Velasco *(Hospital Central De La Defensa Gomez Ulla, Madrid)*; Verónica Alen Villamayor, Santiago Alonso Bartolomé, Miguel Alonso Juarranz, Luis Felipe Ávila-Ramírez, Javier Buendia Pérez, Barbara Burgos-Blasco, Enrique Camarero Rodríguez, Paula Campelos Fernández, Claudia Celotti, Marco Ciappara Paniagua, Ignacio Cristobal, Jana Dziakova, Alejandro Encinas Bascones, Elena Fernandez-Martin, Inés Gil Prados, Jesus Gimeno Hernandez, Maria Alejandra Giraldo, Manuel Gomez Cervantes, Noemi Guemes Villahoz, Araceli Hernández Ramos, Maria Cruz Iglesias Moreno, Bibiana Lasses Martínez, Francisco Leyva Rodríguez, Leyre López Antoñanzas, Javier Martín Monterrubio, Jose Maria Martinez-de-la-Casa, Lourdes Montero Cruces, Jesús Moreno Sierra, Jose Maria Muguerza, Alexia Victoria Paluso Montero, Natalia Pérez Romero, Adriana Poch, Jaime Rodríguez de Alarcón, Adriana Ruano Campos, Rebeca Ruiz Roman, Patricia Saez Carlin, Cristina Sánchez del Pueblo, Lidia Sotillo Valenzuela *(Hospital Clinico San Carlos, Madrid)*; Armando Galvan, Miguel Ángel García Ureña, Enrique González, Manuel Medina Pedrique, Ana Maria Minaya Bravo, Alvaro Robin Valle de Lersundi, Carlos San Miguel *(Hospital del Henares, Madrid)*; Juan Aragón-Chamizo, Monica Ballon, Jose M Barrio, Jorge Caño Velasco, Beatriz Castro Catalan, Laura Cebolla Rojas, Renan Carlo Colombari, Miguel Cuende Diez, Paula Dujovne Lindenbaum, Oscar Gil-de-Sagredo, Silvia Kayser Mata, Pablo Lozano Lominchar, Olga Mateo-Sierra, Yeniffer Tatiana Moreno Salazar, Angela Moreno-Gutierrez, Melanie Morote, Uxue Murgoitio, Nikolas Palma Caucig, Lucía Polanco Pujol, Alejandro Prosperi, Begoña Quintana-Villamandos, María Belén Ramírez Senent, Cristina Rey Valcarcel, Javier Rio, Diego Fernando Ruiz Chiriboga, Mercedes Sanz, Maria Tudela *(Hospital General Universitario Gregorio Marañón, Madrid)*; María Adrien Lara, Raquel Alarza, Isabel Alonso Sebastian, Mariano Artés Caselles, Laura Calles-Sastre, Marcos Casas Sánchez, Aritz Equisoain Azcona, Lucía Fuentes, Lucia Gil Cidoncha, Sofía Herrero Gámiz, Eva Iglesias Garcia, Jose Luis Lucena De La Poza, Mª Eugenia Marín Martínez, Pilar Martín Rodrigo, Joaquin Manuel Muñoz Rodríguez, Verónica Polaino, Alberto Pueyo Rabanal, Xabier Remirez Arriaga, Xiana Rial, Laura Román García de León, Miguel Sánchez Suárez, Maria eugenia Torguet muñoz *(HOSPITAL PUERTA DE HIERRO MAJADAHONDA, Madrid)*; Borja Bazán Inostroza, Lara Blanco Terés, Alba Correa Bonito, Angela de la Hoz, Marcello Di Martino, Gustavo Eisenberg, Javier Garcia Septiem, Jose Maria Lopesino, Rocio Maqueda, Elena Martin-Perez, Jose Luis Muñoz de Nova, Jorge Prada, Julia Revuelta Ramírez, Ana Rodríguez Sánchez, Álvaro Valdés de Anca *(Hospital Universitario de la Princesa, Madrid)*; Laura Colao García, David Díaz Pérez, Enrique Esteban Agustí, Pablo Galindo Jara, Ana Belén Gallardo, Cesar Garcia, Maria Gutierrez Samaniego, Miguel Angel Hernandez Bartolome, Lorenzo Rabadan, Barriga Sánchez Raquel, Javier Serrano González *(Hospital Universitario de Torrejón de Ardoz, Madrid)*; Beatriz Diéguez, Miguel Hernández-García, Manuel Losada *(Hospital Universitario del Sureste, Madrid)*; Carolina Alfonso Carrillo, María Belén Alonso Bartolomé, Estibaliz Alvarez, Mario Alvarez-Gallego, Paula Aragón-Ramos, Pablo Cesar Arteaga Asensio, Luis Asensio Gomez, Diego Carrion, Nuria Chavarrias, Alvaro de Arriba, Alexander Forero-Torres, Jose Antonio Gazo Martínez Gazo, Alberto Gegúndez Simón, Juan Gómez Rivas, Natalia Gonzalez Alcolea, Carolina Gonzalez-Gomez, Sara Gortázar de las Casas, María Alexandra Heras Garceau, Alicia Hernández Gutierrez, Luis Lassaletta, Fuad Samir Lopez Fernández, Marta Mancheño, Blanca Mateos-Serrano, José Manuel Morales-Puebla, Isabel Pascual Miguelañez, Maria Fernanda Pedrero Escalas, Julio Peñarrocha, Yolanda Perez, Maria Isabel Prieto-Nieto, Teresa Rivera Schmitz, Laura Rodrigáñez, Ines Rubio-Perez, Isabel Sanchez Cuadrado, José Ignacio Sánchez Méndez, Jorge fernando Tone, Carlos Toribio Vazquez, Aitor Urbieta, Santiago Valderrabano Gonzalez, Alvaro Yebes, Ignacio Zapardiel *(Hospital Universitario la Paz, Madrid)*; Diego Córdova García, Manuel Diez Alonso, Francisca Garcia-Moreno Nisa, Laura Jiménez, Inmaculada Lasa, Belen Matías-García, Fernando Mendoza-Moreno, Nelson Morales Palacios, Marina Pérez González, Ana Sánchez Gollarte, Alejandro Sanchez Pellejero, Cristina Vera Mansilla *(Hospital Universitario Principe de Asturias, Madrid)*; Rafael Barberá, Lourdes Montes-Jovellar, Fátima Sánchez Fernández *(HOSPITAL UNIVERSITARIO RAMON Y CAJAL, Madrid)*; Alberto Cabañero Sánchez, Sara Fra Fernández, Nicolas Moreno Mata *(Hospital Universitario Ramón y Cajal, Madrid)*; Alfredo Abad Gurumeta, Ane Abad-Motos, Barbara Algar-Yañez, Gutiérrez Bérénice, Fernando Corella, Olga De la Varga-Martínez, Paula Fernandez-Valdes-Bango, Sandra Maria Gadin-Lopez, José María Martínez-Gómiz, Elena Nieto-Moreno, Ana Nieto-Moreno, Montserrat Ocampos Hernandez, Maria Laura Pelegrina-Lopez, Javier Ripollés-Melchor, Alicia Ruiz Escobar, Elena Sáez-Ruiz, Rosa Sanz-Gonzalez, Beatriz Vazquez-Rivero *(Infanta Leonor University Hospital, Madrid)*; Marta Alcaraz Fuentes, Antonio Lara, Belén Vielva del Campo *(HOSPITAL SANITAS LA ZARZUELA, MADRID)*; Fernando Alcaide Matas, Simbad Costas-Ochoa, José María García Pérez, Elizabeth Marmol, Paula Troncoso Pereira *(Hospital Mateu Orfila, Mahon)*; Maria Del Pilar Concejo Cutoli, Maria Teresa Fernández Martín, Juan Ramón Gómez López, Juan Carlos Martín del Olmo *(Hospital Medina del Campo, Medina del Campo (Valladolid))*; Pablo Calvo Espino, Paloma Guillamot Ruano, Javier Páramo Zunzunegui *(Hospital Universitario de Móstoles, Móstoles)*; Antonio Albarracín Marín Blázquez, Alfonso Aliaga-Sanchez, Marta María Arroyo Domingo, Miriam Artés Artés, Ana Isabel Avellaneda Camarena, Aida Blaya, Emny Rochell Bobadilla Romero, Milagros Carrasco Prats, Pedro Vicente Fernández-Fernández, Antonio-José Fernández-López, Lorena Galindo Iñiguez, Damián García Escudero, Víctor Javier García Porcel, Vanesa Garcia Soria, Clara Giménez Francés, Antonio Javier Gomez Poveda, Rebeca Gonzalez Celdran, Francisco Miguel González Valverde, Laura Guillamon Vivancos, Elena Gurrea-Almela, Alejandra Jara Maquilón, Jose David Jimenez Parra, Abd Al Aziz Lanagrán Torres, Raquel Lax Perez, Carmen maria Lopez Lopez, Francisco Machado, Alfonso Marco Garrido, Florencio Manuel Marin Martinez, Jesús Aarón Martínez Alonso, Victoria Martínez Muñoz, Esther Medina, Olimpia Molina, José Manuel Muñoz Camarena, Julian Onate, Pedro Antonio Parra Baños, Emilio Peña Ros, Enrique Rubio, Miguel Ruiz-Marín, Marina Sánchez Robles, Carlos Sanchez Rodriguez, María Valero Soriano, María Teresa Yepes García *(Hospital General Reina Sofía, Murcia)*; Felipe Alconchel, Andrés Balaguer Román, Valentín Cayuela, Ana Conesa, Ana Delegido García, Pedro José Gil Vázquez, Beatriz Gómez Pérez, Paula Gómez Valles, Francisco Gómez-Bosch, Vicente J. León-Muñoz, Francisco Martínez, Joaquin Moya-Angeler, Alvaro Navarro-Barrios, Tatiana Nicolás-López, Pablo Ramirez Romero *(Hospital Universitario Virgen de la Arrixaca, Murcia)*; Emiliano Cano-Trigueros *(Morales Meseguer University Hospital, Murcia)*; María concepcion Alonso González, Leticia Gómez Viana, Ana Pastor Zapata *(Complexo Hospitalario Universitario de Ourense, Ourense)*; Elisa Contreras Saiz, Tamara Díaz Vico, Daniel Fernández Martínez, Maria Fernandez-Hevia, Luis Joaquín García Flórez, Raquel Rodríguez-Uria, Sandra Sanz, Lorena Solar-Garcia, Aida Suárez Sánchez *(Hospital Universitario Central de Asturias (HUCA), Oviedo)*; Jaume Gelonch, Heura Llaquet Bayo, Dietmar Reinaldo *(Hospital de Palamós, Palamós)*; Miriam Abdulkarim Polo, Asier Aguirre, Marta Alomar Bofill, Santiago Baena, Ashish Bartakke, Carmen Cabeza Oliver, Marta Castro Suárez, José Andrés Cifuentes Rodenas, Olga Claramonte Bellmunt, Enrique Colás-Ruiz, Ant`Onia Crespí Mir, Anabel De la Llave Serralvo, Mar Escales, Isabel Rosa Fernández Burgos, Pablo Gandia Gonzalez, Juan Lliteras Jorge, María Cristina Martínez Canto, Javier Mata, Andreu Morell, Naila Pagès, Sara Pérez Palao, Cristian Plaza Valiente, Catalina Ramon Barcelo, Carmen Reyero Fernández, Albert Reyes Claret, Leticia Rodriguez Vaquero, Maialen Saez de Vicuña Salinas, Pilar Santos Cidon, Concepcion Segura, Aina Serra, Maria Serra guivernau, Maria Soler Pedrola, Marta Tasende, Marta Torrent Lluch, María Inmaculada Valldeperas Hernández, Rosa-Maria Yañez-Lpez *(Hospital Universitario Son Llàtzer, Palma de Mallorca)*; Andrea Craus-Miguel, Laura Fernández Vega, Natalia Pujol Cano, Juan José Segura-Sampedro *(Son Espases University Hospital, Palma de Mallorca)*; Susana Bella Romera, Laura Colet Oliver, Ivan Dot Pascuet, Mireia Duart, Enrique Jose Ruiz Velasquez, Isabel Tello Galindo *(Hospital Universitari Sant Joan, Reus)*; Eduardo Arrea Salto, Jaume Cámara Cabrera, Marta Camats Terré, Juan Gabriel Castro Ríos, Marta de la Rosa-Estadella, Anna Fernandez-Colorado, Anna Gasulla-Rodriguez, Antonio Gimenez-Gaibar, Elena Gonzalez, Yeray Maldonado Sotoca, Matilde Molina-Corbacho, Montserrat Monfort Mira, Bernardo Núñez, Josep Rodoreda, Marta Santos Espí, Arnau Verdaguer Figuerola *(Corporación Sanitaria Parc Taulí, Sabadell (Barcelona))*; Elisa Angela Diego-Alonso, Jaime López-Sánchez, Luis Muñoz-Bellvis, Jacobo Trebol *(Complejo Asistencial Universitario de Salamanca, Salamanca)*; Borja Aguinagalde, Cristina Gabriela Alzate Arsuaga, Ainhoa Andres Imaz, Iñigo Arana, Coro Aranzabal Urrutia, Mikel Armendariz, Lorena Arrabal, Iñigo Augusto, Adolfo Beguiristain, Laura Busto, Ainhoa Cuevas, Itziar De Ariño, Alejandro Elúa, Leticia Fernandez, Arantza Fernandez-Monge, Lander Gallego, Alba Garcia, Amaia Garcia Dominguez, Gregorio Garmendia, Carmen Grañén, Pelayo Hevia Rodríguez, Maria Iraola, José Miguel Izquierdo, Raul Jimenez, Aintzane Lizarazu, Jon Ander Lizarbe, Claudia Cristina Lopes Moreira, Iker López, Ruth Marquina González, Tania Pastor, Adrián Recio Ayesa, Ana Paula Riverola Aso, Carballo Rodríguez, Araceli Rodrìguez, Maria Inmaculada Ruiz Montesinos, Patricia Torres, Jose Undabeitia, Ainara Villafruela, Jon Zabaleta *(Hospital Universitario Donostia, San Sebastian)*; Maria Barrionuevo Ramos, David Blanco, Marta Calderón, Eva Corral Rubio, Angel Cuadrado-García, Daniela Cubek, Ignacio Fariña, Maria Fernandez LLorente, Natalia Flores Amador, Mariana García Virosta, Luis Garcia-Sancho Tellez, Adelina Gonzalez Martinez, Maria Jose González-Gimeno, María Hernandez, Rubén Herreros Ruiz-Valdepeñas, Carmen Jimenez Sanchez, Eduardo Llamazares Cobo, María López, Javier López-Martin, Teresa Martínez Marivela, Luis Miguel Martinez Parra, Paloma Maté Mate, Paloma Medrano, Manuel Moriche Carretero, Sara Núñez O’Sullivan, Manuel Ortega Oria de Rueda, Irene Ortega Vázquez, María Begoña Pastor Nieto, Clara Pérez, Antonio Perez Ferrer, Antonio L. Picardo, Remedios Revilla Amores, Carmen Rodríguez Haro, Jose Alberto Rojo López, Fatima Sanchez Cabezudo Noguera, Cristina Sanz, Daniel Serralta de Colsa, Cristina Valor Garcia, Marta Velasco Martinez *(Infanta Sofía University Hospital, San Sebastian de Los Reyes)*; Iván Andújar Lara, Aranzazu Calero-Lillo, Enrique López-Ruiz *(Fundació Hospital de l’Esperit Sant, Santa Coloma de Gramenet)*; Alberto Díaz García, Alejandro Menéndez Moreno, Luis Eduardo Pérez-Sánchez *(Hospital Universitario Nuestra Señora de Candelaria, Santa Cruz de Tenerife)*; Ángela Pascual, Miguel Adeba García, Marta Alonso Fernández, Javier Alvarez Gama, Roberto Ballestero, Loreto Berjon De La Vega, Paola Calleja Hermosa, Mariana Carrillo-Rivas, Alfonso Casado, Andrea Cerveró, Bonifacio Cimadevilla Calvo, Patricia Corriols Noval, Paloma de la Dehesa Cueto-Felgueroso, Miren Jasone Diez Zapirain, José Estevez Tesouro, José Antonio Fernández-Dívar Sánchez, Lucia Garcia Alcalde, Juan García Cardo, Tito García Moreno, Daniel García-López, Belen Garcia-Montesinos Perea, Miren Gonzalez Benito, Rocio Gonzalez-Aguado, Miguel Ángel Gordo Vega Gordo-Vega, Jose Gutierrez-Banos, Jaime Jimeno Fraile, Yolanda Jubete Castañeda, Patricia Lopez Gomez, Antonia Jesús López López, Rubén Martín-Láez, David Mato Mañas, Carmelo Morales-Angulo, Dieter Morales-Garcia, Marcelo Moreno Suarez, Sara Naranjo, Jose Luis Rabago, Jose Manuel Rabanal, Rocio Revuelta Zorrilla, Adriana Reyes Echeverría, Juan Carlos Rodríguez-Sanjuán, Juan Ramón Sanz, Isabel Simal Badiola, Guillermo Tejón, Enrique Toledo Martínez, Raquel Varea Malo *(Marqués de Valdecilla University Hospital, Santander)*; Miguel Angel Freiria Eiras *(Hospital HM Rosaleda, Santiago de Compostela)*; Begoña Fadrique, Tania Funes, Elena González Revilla *(Hospital General de Segovia, Segovia)*; Isaias Alarcón, Daniel Aparicio Sánchez, Pablo Beltran Miranda, Carmen Cepeda-Franco, María Josefa Cuevas López, Sandra Dios-Barbeito, Virginia María Durán Muñoz-Cruzado, Carlos Gonzalez De Pedro, Rosa M Jimenez-Rodriguez, Francisco Manresa-Manresa, Gabriel Marin, Cristobalina Martin-Garcia, Sara Martínez-Núñez, Juan Manuel Martos Martinez, Lucas Mengíbar, Felipe Pareja Ciuro, Eduardo Perea del Pozo, Veronica Pino Diaz, José Pintor-Tortolero, Claudia Quintero-Pérez, Irene Ramallo-Solís, Maria Luisa Reyes Diaz, Mercedes Rubio Manzanares Dorado, Alejandro Sanchez Arteaga, Ana Senent-Boza, Luis Tallon-Aguilar, Jose Tinoco-Gonzalez *(Hospital Universitario Virgen del Rocio, Sevilla)*; Luis-Cristobal Capitan-Morales, Juan Cintas Catena, Juan-Carlos Gomez-Rosado, Javier Valdes-Hernandez *(Hospital Universitario Virgen Macarena, Seville)*; María Del Campo Lavilla, Alina López De Fernández, Jose Ramon Oliver Guillen *(Hospital Santa Bárbara, Soria)*; Miriam Abellan Lucas, Mar Achalandabaso Boira, Alba Diaz Padillo, Maria Gómez Romero, Linda Grace Puerto Tamayo *(Hospital Universitari De Tarragona Joan XXIII, Tarragona)*; El mostafa El Yaqine Er Raoudi, Melody García Domínguez, María Ángeles Gascón Domínguez, Marta González Pérez, Alba Hernáez Arzoz, Joana San Anton *(Hospital Obispo Polanco, Teruel)*; Cristian Ruminot, Hander Guillermo Acosta Diaz, Daniella Laguado *(Hospital Universitario Infanta Elena, Valdemoro, Madrid)*; Kristina Aghababyan, Jr Alba, Enrique Artigues, Jose Bagan, Rocío Belda, Miguel Beltran, Jose Ignacio Blanes, Elena Campos Carot, Miriam Cantos, Laura Costa, Carolina Ferrer Gomez, José María Gallego Sánchez, Oscar Gil-Albarova, Juan Gilabert-Estellés, Emilio Lopez Alcina, Isabel López Sánchez, Severiano Marin Bertolin, Carolina Martinez-Perez, Antonio Melero Abellán, Claudia Mulas Fernández, Judith Murillo, Beatriz Novoa, Ruth Nuñez, Pablo Renovell Ferrer, Guijarro-Jorge Ricardo, Carolina Soledad Romero Garcia, Miguel Sanfeliu Giner, Silvia M. West, Enrique Zapater *(Consorcio Hospital General Universitario, Valencia)*; Julio Domenech, Ignacio Miranda, Ana Monís, Alejandro Roselló Añón, María José Sangüesa, Jorge Sanz Romera *(Hospital Arnau de Vilanova, Valencia)*; Marcos Adrianzén, Rafael Badenes, Maria Barrios Carvajal, Mireia Bauzá, Ana Benítez Riesco, Jose Antonio Carbonell Lopez, María-Carmen Fernández-Moreno, Ricardo Gadea Mateo, Francisco Garcia, Maris Luisa García-Pérez, Luisa Paola Garzon, Ana Izquierdo, Maria Lapeña Rodríguez, Carlos León-Espinoza, Fernando Lopez, Rosa Martí Fernández, Sara Martínez Castro, Berta Monleón López, Isabel Mora Oliver, David Moro-Valdezate, Ernesto Muñoz Sornosa, Sara Palomares Casasús, Leticia Pérez Santiago *(Hospital Clínico Universitario de Valencia, Valencia)*; Mara Albert Fort, Reyes Balanzá, Lorena Bermell Marco, Juan Carlos Bernal-Sprekelsen, Carla Carratalá Pérez, Claudia Pilar Clemente Tomas, Marta Córcoles, Jose Ángel Diez Ares, Carlos Domingo Del Pozo, Nuria Estellés Vidagany, Gonzalo Garrigos, Mar Gimeno Gimeno Vicente, Blanca Alastrue Giner, Segundo Gomez-Abril, Paloma Gonzalez, Paula Gonzálvez Guardiola, Guillermo Sebastian Martínez Fernández, Charo Martínez García, Lucía Martínez-Costa, Luz María Moratalla Charcos, Sergio Navarro Martínez, Carmen Payá-Llorente, Álvaro Pérez Rubio, Nuria Peris, Eduardo Picazo Pineda, Jordi Planelles Gómez, Francisco Ripoll Vidal, Andrea Roca, Enrique Salmerón-González, Raquel Segura Roselló, Ramon Trullenque Juan, Cristina Verdejo Gimeno, Jose Miguel Zaragozá García, Gema Zomeño Bravo *(Hospital Universitario Doctor Peset, Valencia)*; Marcos Bruna Esteban, Hanna Cholewa, Milton de Jesus, Matteo Frasson, Luis Oliver García, Jorge Sancho-Muriel *(Hospital Universitario y Politécnico La Fe, Valencia)*; Héctor J Aguado, Antonio Jose Alonso Villalba, Francisco Ardura, Carmen Elena Badillo Bercebal, María Bedate Núnez, Alvarez-ramos Begoña Aranzazu, Juan Beltrán de Heredia, Hugo Bermejo, Juan Berrocal Cuadrado, Maria Bragado González, Juan Bustamante-Munguira, Raul Calvo Gonzalez, Yolanda Carrascal, Ana Belén Casas Marcos, María Carmen Cervera, Sergio Chávez Valladares, Ángel Cilleruelo Ramos, Susana Cortes, Jesús Crespo-Sanjuán, María Cuaresma, Luis Antonio Cuellar Martin, Beatriz De Andrés-Asenjo, Carlos Ferreras García, Alvaro Fuentes-Martín, Abel Ganso, Elena García García, Luis Garcia-Florez, Virginia Garcia-Virto, Juan Gatón, Julio Alberto Gobernado Tejedor, Tania Gómez Sanz, Jose Ignacio González Martín, María Begoña Gregorio Crespo, Ana Herranz Arriero, Jose Herreros, Marta Ibáñez Nieto, César Infante, Carlos Jezieniecki, Moises Juarez, Gregorio de Jesus Labrador Hernandez, Ana Patricia Legido Morán, Ricardo León Fernández, Alejandro Leon-Andrino, Almudena Llorente, Maria Lopez pais, Mauricio Loucel Bellino, Sergio Martin, Miguel Angel Martin-Ferrero, Gonzalo Martinez Municio, Elvira Mateos Alvarez, José María Matilla, Luis María Merino Peñacoba, Mario Montes-Manrique, Fernando Natal Álvarez, David C Noriega, Henar Nuñez Del Barrio, Francisco Javier Ortiz de Solórzano Aurusa, Lucía Pañeda, Victoria Pascual Escudero, Miguel Pascual Samaniego, Adela Pereda, Marta Pérez Febles, Laura Pesquera, Maria Plata, Emma Puertas Ruiz, Mario Rodriguez-Lopez, Alejandro Romero de Diego, Ana Ruano, María Ruiz Soriano, Cristina Sánchez Torralvo, Silvia Santiago Maniega, Bárbara Segura-Méndez, Clarisa Simon Perez, José Soro-García, Paula Suárez Mansilla Suárez-Mansilla, Jeancarlos Trujillo Díaz, Mª Esther Valsero Herguedas, Andrea Vazquez Fernandez, Eduardo Velasco García, Ana Zabalza, Álvaro Zamora Horcajada *(Hospital Clínico Universitario de Valladolid, Valladolid)*; Fernando Acebes García, Enrique Asensio Díaz, Martín Bailón, Alejandro David Bueno Cañones, Alvaro Centeno Velasco, Ekta Choolani Bhojwani, Fernando Labarga Rodríguez, Sara Mambrilla, Pablo Marcos-Santos, Toledano Miguel, David Pacheco Sánchez, Baltasar Pérez-Saborido, Pilar Pinto, Katherine Plua, Javier Sanchez Gonzalez, Vicente Simo, Francisco J Tejero-Pintor, Rosalía Velasco López, Sandra Veleda Belanche *(Hospital Universitario Río Hortega, Valladolid)*; Fabiola Eugenia Michel Campos, Iciar Muñoz Lindez, Marc Vallve-Bernal *(Hospital Insular Nuestra Señora de Los Reyes, Valverde)*; Maria Balluerca, Maite Camuera, David Garcia, Pablo Helguera, Imanol Herrero, Patricia Paunero Vazquez, María Sánchez-Rubio, Isami Soeda, Alba Vazquez Melero *(Hospital Universitario Araba, Vitoria-Gasteiz)*; María Dolores Arribas Del Amo, Vicente Manuel Borrego Estella, Estefania Casas, Néstor Castán Villanueva, Daniel Delfau Lafuente, Elena Delgado Blanco, María Domingo, Ismael Gil Romea, Azucena Gonzalo, Emilio Lagunas Lostao, Noelia Lete Aguirre, Yaiza Martinez Lahoz, Jesús Víctor Pérez-Tierra Ruiz, Maria del Mar Soriano, Isabel Valero Lázaro *(Hospital Clinico Universitario Zaragoza, Zaragoza)*; Esmeralda Carnicer Escusol, Guillermo Pola Bandres, Laura Sánchez Blasco *(Hospital General de la Defensa, Zaragoza)*; Juan Luis Blas Laina, Beatriz Cros, Jorge Escartin, Patricio Andrés Freile Pazmiño, David Garcia-Aguilera, Ana Mejía Casado, Ana Nogués, Issa Talal El-Abur, Carlos Yánez *(Hospital Royo Villanova, Zaragoza)*; Victoria Duque Mallén, Isabel Gascon Ferrer, Carlos Gracia-Roche, Ursula María Jariod Ferrer, María Victoria Simón Sanz, Tomas Uson *(Hospital Universitario Miguel Servet, Zaragoza)*. |
| **Sri Lanka**: Waseem Ahamed, Sumudu Jayasinghe, Eranda Karunadasa, Prathibha Nelihela, Selvaratnam Srishankar, Sujeewa Priyantha Bandara Thalgaspitiya, Dickson Wickramarathna *(Teaching Hospital Anuradhapura, Anuradhapura)*; Nagenthiram Harivallavan, Umesh Jayarajah, Pirashanthan Nagarasa, Eranga Perera, Kanapathipillai Piratheep, Vithranage Srimantha Dewsiri Rodrigo, Charitha Sooriyabandara, Meera Thayalan, Rajendram Thayaparren *(District General Hospital Chilaw, Chilaw)*; Arulprashanth Arulanantham, Oshan Basnayake, Kanishka De silva, Gagana Ganga, Anuja Nadeeshan Kumarasinghe, Mahanada Udukala, Koculen Vimaleswaran, Prashanthan Yogarajah *(Cancer Institute Maharagama, Colombo)*; Ravindri Jayasinghe, Probhodana Ranaweera, Mohamed Rishard, Dharmabandhu N Samarasekera, Sanjeewa Seneviratne, Sivasuriya Sivaganesh, Dakshitha Wickramasinghe *(National Hospital of Sri Lanka, Colombo)*; Chamal Fernando, Nishantha Mendis, Prabuth dulanjan Weeraddana *(Base Hospital Horana, Sri Lanka, Horana)*; Balasingam Balagobi, Balasupramaniam Sathesan, Thanusan Vimalakanthan *(Teaching Hospital,Jaffna, Jaffna)*; Lanka Dasanayake, Malitha Patabendige, Dj Wickramasooriya *(Teaching Hospital, Mahamodara, Galle, Mahamodara, Galle)*; Kugarajh Ganeshapillai, Shanthamoorthy Gishanthan, Sathyamoorthy Prasanna *(District General Hospital Polonnaruwa, Polonnaruwa)*; Malitha Patabendige *(Base Hospital, Pothuvil)*; Pramodh Chandrasinghe, Dileepa Ediriweera, Sumudu Kumarage *(North Colombo Teaching Hospital, Ragama)*; Asanka Jayawardane, Athula Kaluarachchi, Malitha Patabendige, Pituwala Liyanage Adithya Sirisena *(De Soysa Hospital for Women, Sri Lanka)*. |
| **Sudan**: Abubakr Abubakr, Hozifa Mohamed, Ismail Mustafa, Mogahid Sharfeldein *(Al-Hassahissa Teaching Hospital, Al-Hassahissa)*; Samir Ismail, Reem Musa, Monira Sarih *(Elduiem Teaching Hospital, Elduiem)*; Mussab Abaker, Eman Adam Abdalla, Mohamad Abdelbagi, Alsafe Adlan, Khalid Ahmed, Ahmed Alnaeem, Safa Atyah, Yasir Awad, Rsheeda Dawelbait, Mohamed EL Hag, Ibrahim Elbashir, Sami Eldirdiri, Samwal Eldirdiri, Mohammed Eltahier Abdalla Omer *(Gadarif teaching hospital, Gadarif city)*; Lana Abdalgadir Ahmed Mohamed, Hiba Hassan Rehmtallah Ahmed, Mohammed balla Yousif balla *(Aldosougi Specialised Hospital, Khartoum)*; Abdulmoiz Aljafari, Abdulmalek Aljafari, Elsagad Mohamed, Abdulkader Mohammad, Abdulkader Shaar, Mohamed nafea Shaar *(Bashair Teaching Hospital, Khartoum)*; Maab Hussein Yousif Elhafiz, Mohammed Husien Yosif Elhafiz, Abubakr Ali Mohammed Alhassan Humidan *(Best Care Hospital, Khartoum)*; Almigdad Ali, Hisham Eljack, Mohamedyasin Elrashid *(Hajelsafie Teaching Hospital, Khartoum)*; Manhal Ahmed, Albrra Alhag, Douaa Ali, Mohammed ahmed Babikir, Imad Bakheit, Hytham K. S. Hamid, Sami Mohamed, Walid Shaban, Nidhal Siddig, Mohamed Suliman, Rihab Yasir Abdelmagid, Abubaker Yassin *(Ibrahim Malik Teaching Hospital, Khartoum)*; Omer Abbas, ElTahir Abdelrahim, Muhammed Ahmed, Mohamed Elobaid, Adil Hashim, Ali Mohamed, Omer Mohamed, Khalid Osman, Abdallah Yousif *(Kuwaiti Specialized Hospital, Khartoum)*; Safaa Abdulaal, Marwa Elsadig, Mohammed Hussain *(Ribat university hospital, Khartoum)*; Malaz Amir Ataalmanan, Doha Amir AtaAlmanan, Tebyan Amir AtaAlmanan, Alaa Elgaili, Maria Mohamed, Usra Omara, Alkhansa Osman, Amna Osman, Safaa Saad *(Soba University Hospital, Khartoum)*; Mohammed Elmujtba Adam Essa Adam, Abdelkareem Ahmed *(Medical and cancer research Institute, Nyala)*; Ali Adil Ali karar *(Al-rajhi, Omdurman)*; Aya ALhassan, Mohanad Alsidig, Mohamed Eljack, Mohammed Ibrahim Mohammed Ali, Awad Kamal Awad Osman, Liena Mohamed, Samah Ibrahim Omer Mohamed Osman Mohamed Osman, Altayeb Mohammed, Ayman Mohammed, Khadija Muhmmed, Eltahir Musa, Egbal Sahal Abdelmajed *(Bugaa Specialised Hospital, Omdurman)*; Khalid Z.M.Y Hamid, Omnia M. M Yousif *(AUmmdawaban Hospital, Ummdawaban)*; Mahmoud Saleh *(University of Gezira Hospital, Wad Madani)*; Osman Bashir, Yassein Elhussein, Omar Madani *(Gezira Trauma and Orthopedic center, Wad Medani)*. |
| **Sweden**: Eva Angenete, Jennifer Park *(Sahlgrenska University Hospital, Gothenburg)*; Lars Gillberg, Monir Jawad, Linas Pieteris, Yousif Saeed, Sergej Safonov, Martin Spångfors *(Central Hospital in Kristianstad, Kristianstad)*; Henrik Andersson, Hans Bahlmann, Karin Björnström Karlsson, Michelle Chew, Lara Daham, Lina De Geer, Helen Didriksson, Gunilla Gagnö, Carina Jonsson, Anna Oscarsson *(Linköping University Hospital, Linköping)*; Pamela Buchwald, Mathilde Delorme, Marie-Louise Lydrup *(Skane University Hospital, Malmo)*; Shahin Mohseni, Arvid Pourlotfi, Souheil Reda *(Orebro University Hospital, Örebro)*; Kajsa Anderin, Nina Blomme, Josefin Segelman, Malin Simlund, Anders Thorell, Fredrik Wogensen *(Ersta Hospital, Stockholm)*; Daniel Danielsson, Peter Elbe, Rebecka Hultgren, Boel Hynning, Fredrik Klevebro, Karin Lind, Ebba K Lindqvist, Carl Montan, Ernesto Sparrelid, Clara Svenberg Lind, Jeremy Wales *(Karolinska University Hospital, Stockholm)*; Andreas Älgå, Martin Nordberg, Emil Pieniowski *(South General Hospital, Stockholm)*; Ioannis Gkekas, Niklas Löfgren, Karolina Niska, Martin Rutegård, Malin Sund, Robert Zürner *(Umea University Hospital, Umea)*. |
| **Switzerland**: Silvio Däster, Otto Kollmar, Savas Soysal, Stephanie Taha-Mehlitz, Athanasios Tampakis *(University Hospital Basel, Basel)*; Dominik Valentin Flury, Marie Heyne-Pietschmann, Gregor Jan Kocher *(Inselspital, Bern University Hospital, University of Bern, BERN)*; Ana M Calinescu, Barbara Wildhaber *(Geneva University Hospitals, Geneva)*; Markus Gass, Jürg Metzger, Andreas Scheiwiller *(Luzerner Kantonsspital, Luzern)*; Lukas Briner, Oliver Dwidar, Eleftherios Gialamas, Steven Grandjean, Pietro Ricciardi, Marc-Olivier Sauvain *(Hopital de Pourtales, Neuchatel)*; Alexandra Calmels, Christodoulou Michel *(Hôpital de Sion, Centre Hospitalier du Valais Romand, Sion)*; Morena Antonilli, Dimitrios Christoforidis, Alessandra Cristaudi, Jacopo Galafassi, Maria Luisa Gasparri, Francesco Mongelli, Martino Munini, Andrea Papadia, Sotirios Georgios Popeskou *(Ente Ospedaliero Cantonale, Ticino (Lugano, Bellinzona, Locarno, Mendrisio))*; Michel Adamina, Alex Alfieri, Thomas Bächler, Matteo Giardini, Christian Gingert, Laura Guglielmetti, Inna Meyer, Tobias Müller, Daniel Schöni, Folco Solimene, Frank Weidner *(Kantonsspital Winterthur, Winterthur)*; Marco Bueter, Daniel Gero, Karoline Horisberger, Amelie Müller, Carmen Portenkirchner, Andreas Thalheimer, Jeannette Widmer *(Universitätsspital, Zürich)*. |
| **Syrian Arab Republic**: Ahmad Hmaideh, Alaa Kour, Saleem Swaes *(Al-shifa Hospital, Afrin, Aleppo)*; Omar Alannaz, Ramez Aljasem, Ezeddin Dabbagh, M.wafa Hamoud Alhussein, Amr Hamza, Mazen Mohammad, Abd El-Fattah Mouhandes, Mohamad Shareeda, Mohammad Nour Shashaa, Mousa Sifat, Sheraa Tahhan, Rahaf Toutounji *(Abd Al Wahab Agha Hospital, Aleppo)*; Mohamed Abdulrahman, Albaraa Abdulsalam, Ziad Al jarad, Hala Alayyoubi, Dina Alfarra, Ahmad Alhamid, Mahmoud Alnasser, Mulham Gharib, Ruwaid Gharib, Obaida Kabel, Osama Kherallah, Abdulqader Klaho, Mohamed shukri Najjar, Ahmad Razouk, Joud Shiekhoni *(Al-Shahbaa Private Hospital, Aleppo)*; Wael Alkhaleel, Amira Almosa, Mohammad Aloulou, Maen Alzaeem, Khaled Arnaout, Muhamad Zakaria Brimo Alsaman, Mohamad Estanbouli, Hanadi Hawa, Ahmad nbrass Kabawi, Ghina Maarawi, Yehia Mashhadany, Sana Shaikh Torab *(Aleppo Private Hospital, Aleppo)*; Waleed Abd, Tayma Abd Alghafour, Hani Abdalnour, Nazeeh Al Aktaa, Marwan Al Aliwy, M Maher Al arje, Tasneem Al najjar, Ahmad Al-Mouakeh, Ahmad Aldakhil, Aos Alhamid, Hasan Alhasan, Mohamad Shadi Alkarrash, Jumaa Alkhamis, Sara Alshekh, Ahmad Yamen Arnaout, Oula Azizeh, Mohammed Dalaleh, Aghyad Danial, Zain Douba, Raghad Ghadri, Mohammad Maen Ghannam, Anas Ghawi, Ahmad Ghazal, Luma Haj Kassem, M.yasser Halwani, Shahed Hamadieh, Mohammad Hanino, Ala aldeen Hasan, Haya Jawish, Nasr Ullah Kabbani, Saadullah Kabbany, Mohamad Kadi, Lama Kadoura, Ahmad Amir Kayali, Ammar Kayyali, Aya Kelzia, Amana Kezze, Abdullah Khalaf, Maymona Khayata, Mohammad Nour Kitaz, Mohammad Kour, Raghad Makki, Ruqaya Masri, Joseph Masri, Noor Masri, Muhammad Mazketly, Shiar Mustafa, Ezzeldin Nashed, Yaman Qoudra Danial, Hasan Raslan, Rima Salem, Mohammed Asaad Salem, Thabet Sayed Bakir, Manaf Sharabaji, Majd Sheikh Alganameh, Duaa Sheikh Kadro, Joudy Shurbatji, Mohammad noor Sultan, Dana Sultan, Hala Swied, Bakri Tarras Jarkas, Tayf Toutounji, Fadi Ward, Rama Zazo, Aya Zazo, Zainab Zeino *(Aleppo University Hospital, Aleppo)*; Mohamed Alaktaa, Wael Alsado, Hala Fares, Nouran Hawa, Hamza Ibrahim, Abdulmonem Kawas, Mourad Niazi, Bayan Toutounji *(St Louis Hospital, Aleppo)*; Mohammad Abdow, Mohamad Ahmad, Tareq Ahmad, Rawan Al Azhar, Hiba Al Hage Diab, Rahaf Al Mulke, Mohammad Al-Jadaan, Ahmad Alhussein, Zahra Alnajem, Omar Alneser, Bayan Alsaid, Elias Azar, Lubna Bakr, Ammar Hamza, Yara Houdifa, Sami Jomaa, Samer Kefo, Hiba Mardini, Alaa Mohammad, Fadi Rayya, Fatima Razzouk, Mohannad Saleh, Anwar Shamandi, Ammar Youzkatli Alkhatib *(Al-Assad University Hospital, Damascus)*; Omar Abdul Salam, Dany Ajami, Yusra Al-Sabbagh, Mohammad Alhamid, Ahmad Alkhaledi, Danny Hadidi, Massa Jabra, Mamdouh Kallas, Antoine Laktine, Joelle Naem, Naya Naoum *(Al-Bairouni University Hospital, Damascus)*; Ghena Mohammed fawaz Ashour, Ahmad Alhouri, Deema Fallouh, Ahmad N Mohammad, Ahmad Rmman, Ali Slitin, Christine Tanos, Ibrahim Wakim, Hanaa Zahrawi *(Al-Hilal Hospital, Damascus)*; Majd Abouassi, Layth Abouassi, Ayham Aboutrab, Basel Ahmad, Ahmad Mustafa Ahmad, Aya Ahmad, Afaf Ahmad, Mohamad Alabras, Mohammad Albasheer, Ola Alkasser, Mhd Khaled Alkasser, Ali Alkhdor, Marah Azkoul, Mohammad Karam Chaaban, Haya Deeb, Basel Jazieh, Basel Kouz, Youssef Kozah, Raied Mohamad, Ammar Mohammad, Antoine Naem, Mhd Zied Nasri, Mohammed Mouaz Shebani, Mohammed Shwin, Alaa Sommaq, Amal Youssef *(Al-Mouwasat University Hospital, Damascus)*; Tala Al Asadi, Suhad Alkhateb, Rasha khalil Alsayyad, Roula Altom, Mhd Amin Alzabibi, Batoul Bakkar, Hlma Ismail, Salma Khadem Alsrouji, Sara Melhem, Mohamad Sankari, Mosa Shibani, Noura Shujaa *(Damascus Hospital, Damascus)*; Ibrahim Adham, Salma Al- Houssami, Maram Balouli, Ghassan Jisry, Yazan Rahmeh, Simon Yanni, Sally Yousef *(Obstetrics and gynaecology University hospital, Damascus)*; Aline Ahmad, Marah Ali, Abdulrahman Almjersah, Aya Almoustafa, Hiba Dahhan, Bashar Haj Hassan, Alaa Hamdan, Ali Hammed, Salah Hammed, Rawan Harfoush, Rama Hasan, Natalie Jarkas, Moufid Mahfoud, Ahmed Moussa, Alaa Sulaiman, Siba Suliman *(Tishreen University Hospital, Latakia)*. |
| **Taiwan, China**: Yu-Ning Hu, Jun-Neng Roan, Meng-Ta Tsai, Yi-Chen Wang *(Taiwan, Tainan, National Cheng Kung University, College of Medicine, National Cheng Kung University Hospital, Department of Surgery, Tainan)*. |
| **Thailand**: Varut Lohsiriwat, Thammawat Parakonthun, Nicha Srisuworanan, Voraboot Taweerutchana *(Faculty of Medicine Siriraj Hospital, Mahidol University, Bangkok)*; Somcharoen Saeteng, Sophon Siwachat, Apichat Tantraworasin *(Faculty of Medicine, Chiang Mai University, Chiang Mai)*. |
| **Trinidad and Tobago**: Shane Charles, Danielle Dimsoy, Shane Khan, Akash Ramsaroop, Shivanand Ramsubhagh *(San Fernando General Hospital, San Fernando)*. |
| **Tunisia**: Karim Ayed, Oussama Baraket, Mohamed Hedi Ghalloussi, Abbassi Imed *(Hôpital Habib bougatfa bizerte, Bizerte)*; Nahla Kechiche *(University Hospital Fatouma Bourguiba, Monastir)*; Waad Farhat, Ammar Houssem *(Sahloul Hospital, Sousse)*. |
| **Turkey**: Erdal Birol Bostanci, Volkan Oter, Erol Pişkin *(Ankara CITY Hospital, Ankara)*; Cihangir Akyol, Sibel Demirel, Mehmet Ali Koç, Can Konca, Özge Yanık *(Ankara University Medical School, Ankara)*; Basak Bolukbasi, Huseyin Gobut, Emre Gülçek, Kerim Karabulut, Ramazan Kozan, Sezai Leventoğlu, Farshad Noori, Elif Özeller, Can Şahin, Ali Yalcinkaya, Mesut Yavaş, Aydın Yavuz *(Gazi University Medical Faculty Hospital, Ankara)*; Utku Akgor, Omer Cennet, Hilmi Anil Dincer, Murat Gultekin, Nazlı Orhan, Mustafa Oruç *(Hacettepe university hospital, Ankara)*; Serdar Culcu, Lütfi Doğan, Cemil Yüksel *(SBÜ Ankara Onkoloji Eğitim ve Araştırma Hastanesi, Ankara)*; Alp Yildiz, Aybala Yildiz *(Yildirim Beyazit University Yenimahalle Training and Research Hospital, Ankara)*; Perihan Ekmekçi, Baturay Kansu Kazbek, Ülkü Ceren Köksoy, Deniz Serim Korkmaz, Zeynep Şahan Çeti̇nkaya, Eli̇f Aybeni̇z Yildirim, Hakan Yilmaz, Metin Yığman *(UFUK ÜNİVERSİTESİ TIP FAKÜLTESİ DR.RIDVAN EGE SAĞLIK ARAŞTIRMA UYGULAMA MERKEZİ HASTANESİ, ANKARA)*; Nasuh Utku Dogan, Selen Doğan, Mehmet Sakinci *(Akdeniz University Hospital, Antalya)*; Ozgen Isik, Murat Şen, Tuncay Yılmazlar *(Bursa Uludag University School of Medicine, Bursa)*; Utku Özgen, Ugur Sungurtekin *(Pamukkale University School of Medicine, Denizli)*; Arda Isik *(Erzincan University Hospital, Erzincan)*; Yener Aydin, Ilker Ince, Ali Bilal Ulas *(Ataturk University School of Medicine, Research and Training Hospital, Erzurum)*; Hüseyin Cahit Yalçın *(Dr Ersin Arslan Eğitim ve Araştırma Hastanesi, Gaziantep)*; Mehmet Kaplan, Tuğba Kaplan, Elif Tugce Kaplan *(NCR International Hospital, Gaziantep)*; Afag Aghayeva, Bilgi Baca, Inci Sahin *(Acibadem Altunizade Hospital, Istanbul)*; Zumrud Aliyeva, Akif Enes Arikan, Erman Aytac, Onur Dülgeroğlu, Volkan Ozben, Cihan Uras *(Acibadem Atakent Hospital, Istanbul)*; Emre Sivrikoz *(Acibadem Bakirkoy Hospital, Istanbul)*; Akif Enes Arikan, Ismail Ahmet Bilgin, Bahadır Bozkırlı, Güralp Onur Ceyhan, Ismail Hamzaoglu, Halil Kara, Tayfun Karahasanoğlu *(Acibadem Maslak Hospital, Istanbul)*; Olus Api, Mehmet Ceyhan, Aylin Pelin Cil, Telce Aysen Gurbuz, Cumhur B�Lent Urman *(American Hospital, Istanbul)*; Ahmet Akbas, Yunus Emre Aktimur, Yuksel Altinel, Fikret Calikoglu, Serdar Demirgan, Ahmet Guray Durmaz, Gulcin Ercan, Candas Ercetin, Hasim Furkan Gullu, Nadir Adnan Hacım, Serhat Meriç, Kamil Özdoğan, Merve Tokocin, Mehmet Alim Turgut, Talar Vartanoglu, Hakan Yigitbas *(Bagcilar Research And Training Hospital, Istanbul)*; Hüsnü Aydın, Ece Bahçeci, Mestan Bilmez, Elif Binboğa, Sinan Binboga, Gokhan Demirayak, Bengi Demirayak, Keziban Doğan, Ahmet Cem Dural, Murat Ekin, Sina Ferahman, Fatih Guven, Mehmet Karabulut, Sema Karakaş, Selçuk Köse, Ayşe Büşra Önder, Ismail Umut Onur, Nuri Alper Sahbaz, Levent Yaşar, Ulviye Yigit, Güneş özlem Yıldız *(Bakirkoy Dr. Sadi Konuk Training And Research Hospital, Istanbul)*; Ümmühan Zeynep Bilgili, Bayram Doğan, Cevper Ersoz, Abdullah Ilktac, Senad Kalkan, Eyyüb Selim Ünlü *(Bezmialem Vakif University, Faculty of Medicine, Istanbul)*; Halil Alis, Süleyman Büyükaşık, Burak Kankaya *(Istanbul Aydin University Hospital, Istanbul)*; Oguzkagan Batikan, Ensar Çakır, Musa Murat Caliskan, Mahmut emin Çiçek, Anil Demi̇r, Selim Doğan, Emre Erdoğan, Mert Güler, Muhammed Gürlük, Ufuk Oguz Idiz, Rozan Kaya, Cebrail Oğuz, Ömer Akay Ömer, Serkan süleyman Özpak, Hüsnü Şevi̇k, Mert Mahsuni Sevinc, Cihad Tatar, Oğuzhan Teki̇n, Furkan Türkoğlu, İshak Yıldız *(Istanbul Education and Research Hospital, Istanbul)*; Gokhan Atis, Mehmet Çağlar Çakıcı, Ayberk İplikçi, Ozgur Kazan, Asif Yildirim *(Istanbul Medeniyet University, School of Medicine, Istanbul)*; Mustafa Yücel Boz, Rahim Horuz, Mustafa Soytas *(Istanbul Medipol University Hospital, Istanbul)*; Müserref Beril Dincer, Ali Fuat Kaan Gok, Irem Karatas, Metin Keskin, Kemalettin Koltka, Mukadder Orhan Sungur, Ilker Ozgur *(Istanbul University - İstanbul Faculty of Medicine, Istanbul)*; Tuba Banaz, Tugan Bese, Sukru Cebi, Fuat Demirkıran, Ergin Erginöz, Sefa Ergün, Betul Ibis, M. Bugrahan Karaca, Mehmet Faik OZcelık, Ahmet Necati Sanli, Akif Turna, Server Sezgin Uludağ, Mehmet Velidedeoglu, Beyza Irem Yabaci, Abdullah Kağan Zengin *(Istanbul universty - Cerrahpaşa Medical faculty, Istanbul)*; Cem Emir Guldogan, Emre Gundogdu, Mehmet Mahir Ozmen *(Istinye University Hospital, Istanbul)*; Mehmet Abdussamet Bozkurt, Yasi̇n Kara, Ali Kocataş, Adem Özcan *(Kanuni Sultan Suleyman Training and Research Hospital, Istanbul)*; Oktay Akça, Elif Akova Deniz, Berk Cimenoglu, Recep Demirhan, Elif Cansu Gundogdu, Alper Kafkasli, Ahmet Kale, Kemal Saracoglu, Ramazan Sarı, Muhammed Fatih Simsekoglu *(Kartal Dr. Lutfi Kirdar Training and Research Hospital, Istanbul)*; İbrahim Fethi Azamat, Emre Balik, Dursun Buğra, Burak Giray, Cemil Burak Kulle, Cagatay Taskiran, Dogan Vatansever *(Koç University Medical School, Istanbul)*; Aykhan Abbasov, Hakan Yanar *(Liv Hospital Ulus, Istanbul)*; Ahmet Akmercan, Gül Çakmak, Tuncay Kötan, Tunc Lacin, Ayten Saracoglu, M. Umit Ugurlu, Tumay Umuroglu, Tevfik Kıvılcım Uprak, Esra Yamansavci Şirzai, Bedrettin Yıldızeli *(Marmara University, School of Medicine, Istanbul)*; Elif Baran, Emre Bozkurt, Sinan Ömeroğlu, Mert Tanal, Aydın Eray Tufan, Mehmet Uludag, Gürkan Yetkin *(Sisli Hamidiye Etfal Training and Research Hospital, Istanbul)*; Murat Kalın, Omer Faruk Ozkan, Hanife Şeyda Ülgür *(University of Health Science Umraniye Education and Research Hospital, Istanbul)*; Tayfun Bisgin, Muhammet Berkay Sakaoglu, Selman Sökmen *(Dokuz Eylul Univ. Hospital, Izmir)*; Deniz Caglar, Candan Cicek, Demre Delipinar, Emre Divarci, Egemen Ozdemir, Hilmican Ulman, Seda Dilek Yetut, Feyme Zafer *(Ege University Hospital, Izmir)*; Ismail Sert *(Izmir Egepol Surgical Oncology Hospital, Izmir)*; Burçin Abud, Murat Akalin, Cengiz Aydin, Bulent Calik, Emine Burcu Cigsar, Semra Demirli Atıcı, Ayberk Dursun, Mustafa Emiroglu, Hüseyin Erdi̇nç, Tayfun Kaya, Gizem Kilinc, Yasemin Kirmizi, Merve dilara Öney, Mustafa Onur Oztan, Semra Salimoğlu, Cem Tugmen, İbrahim Uyar *(University of Health Sciences Tepecik Training and Research Hospital, İzmir)*; Sertaç Ata Güler, Alican Güreşin, Ozan Can Tatar, Nihat Zafer Utkan *(Kocaeli University Teaching Hospital, Kocaeli)*; Kemal Arslan, Mustafa Bilal Hamarat, Ismail Hasirci, Mehmet Serkan Ozkent, Mehmet Eşref Ulutaş, Burak Yilmaz *(Konya Training and Research Hospital, Konya)*; Mertcan Akçay, Yesim Akdeniz, Emrah Akin, Fatih Altintoprak, Enes Baş, Zulfu Bayhan, Guner Cakmak, Recayi Çapoğlu, Fehmi Çelebi, Hakan Demir, Enis Dikicier, Ugur Can Dulger, Necattin Firat, Emre Gönüllü, Ahmet Tarik Harmantepe, Muhammet Burak Kamburoğlu, Belma Kocer, Ibrahim Furkan Küçük, Baris Mantoglu, Ali̇ Muhtaroğlu, Kayhan Ozdemir, Merve Yigit *(Sakarya Faculty Of Medicine, Sakarya)*; Zehra Alan köylü, Engi̇n Aybar, Mert Candan, Ahmet Burak Ciftci, Eli̇f Çolak, Huseyin Eraslan, Gultekin Ozan Kucuk, Süleyman Polat, Ahmet Can Sarı, Mustafa Safa Uyanik, Kürşat Yemez *(Samsun Training and Research Hospital, Samsun)*; Çağrı Büyükkasap *(Siirt State Hospital, Siirt)*; Kayahan Eyuboglu, Ali Guner, Murat Emre Reis *(Karadeniz Technical University Farabi Hospital, Trabzon)*; Nurullah Damburacı, Omer Karahan, Barış Sevinç *(Uşak University Medical School, Uşak)*; Fatma Ayca Gultekin *(Zonguldak Bulent Ecevit University School of Medicine Research and Training Hospital, Zonguldak)*. |
| **Uganda**: Okedi Francis Xaviour, Odongo Samuel *(Adjumani General Hospital, Adjumani)*; Mohammed Hirsi, Gaston Turinawe *(Hoima regional referral hospital, Hoima)*; Isakwa Ibrahim, Isabirye Isa, Isaac Mubezi *(Iganga district hospital, Iganga)*; Daniel Asiimwe, Mumbere Bienfait, Isaac Edyedu, Franck K. Sikakulya *(Kampala International University Teaching Hospital, Ishaka)*; Andrew Kakeeto, Esther Namutosi, Mutekanga Nicholus *(Jinja regional referral hospital, Jinja)*; Ssekitooleko Badru, Treasure Ibingira, Ehanga Idi Marcel, Racheal Kirabo, Hervé Monka Lekuya, Ronald Mbiine, Cephas Nakanwagi, Christine Namugenyi, Joseph Ntege, Arnold Ntege, Henry Othieno misanga, Nantambi Rose, John Baptist Ssenyondwa, Nimanya Stella Alice *(Mulago Referral hospital, Kampala)*; Lois Asiimwe, Clara Atieno Odhiambo, Francis Basimbe, Brian Bbosa, Walter Dreak Erabu, Bili Hussein, Joel Kiryabwire, Castro Kisuule, Ronald Kiweewa, Moses Magezi, Lynette Mpagi Katassi, Dionizi Muganga, Didace Mugisa, Othiniel Musana, Fredderick Mutyaba, Twaha Muwanga, Peter Muwanguzi, Assumpta Nabawanuka, Teddy Nakirijja, Goretti Nassali, Rosemary Nassanga, Moses Nassimu, Peter Ssekweyama, Peter Ssenyonjo, Kagga Ssenyonjo, Eden Micheal Ssettabi, Linda Turyabahika, Justine Walusimbi, Daniel Zaake *(St Francis Hospital Nsambya, Kampala)*; Herman Lule, Benson Oguttu *(Kiryandongo Hospital, Kigumba)*; Paul Matovu, Dr. Denis Oluka, Mujuni Samson *(Kisiizi Hospital, Rukungiri)*; Otolia Isaac, Okello Simon Peter *(Soroti Regional Referral Hospital, Soroti)*. |
| **Ukraine**: Vladyslav Hordoskyi, Andrii Kurmanskyi, Sofiia Zhurba *(Kyiv City Hospital No 7, Kyiv (Kiev))*; Maksym Boruta, Andrey Kebkalo, Volodimir Tyselskiy *(Kyiv Regional Clinical Hospital, Kyiv (Kiev))*; Andriy Beznosenko, Olha Bubliieva, Viktor Cherniienko, Slava Kopetskyi, Yevhenii Kostiuchenko, Bogdan Maksymenko, Alina Minich, Sikachov Sergei *(National cancer institute, Kyiv (Kiev))*; Ihor Yovenko *(Medical Home Odrex, Odessa)*. |
| **United Arab Emirates**: Khaled ElSisy, Ahmed Ibrahim, Mohamed Mashhour *(Mediclinic Alnour Hospital, Abu Dhabi)*; Amin El Helw, Ravi Trehan *(Sheikh Shakhbout Medical City, Abu Dhabi)*; Sattar Alshryda, Vikram Somashekhar Basappanavar, Hisham M. Elsayed, Rajnish Garg, Suresh Kailasam Sivamurthy, Sabina Khan, Safeena Kherani, Rubina Lone, Ibrar Majid, Awadelkarim Mohamed, Abid Qazi, Mohamed Serour *(Al Jalila Children’s Speciality Hospital, Dubai)*; Mohamed Mashhour, Nayzak Raoof, Gavin Spence *(Burjeel Hospital for Advanced Surgery, Sheikh Zayed Road, Dubai)*; Ferial Mohamed Ali Abbas, Maryam Babar *(Dubai Hospital, Dubai)*; Sheela Anne George Varayannoor, Diary Mohammed, Muna AbdulRazzaq Tahlak *(Latifa Women and Children Hospital, Dubai)*; Khalid Alawadi, Ehab Aldlyami, Amar Alomar *(Medcare Orthopaedic and Spine, Dubai)*; Taiceer Abdulwahab, Murad Abdunabi, Mohamed Mashhour *(Mediclinic City Hospital Dubai, Dubai)*; Rakesh Kundra, Mohamed Mashhour *(Mediclinic Parkview Hospital, Dubai)*; Amr El Yamany, Mohamed Mashhour *(Mediclinic Wellcare Hospital, Dubai)*; Hossam Al Mahdy, Antony Louis Rex Michael *(Neurospinal Hospital, Dubai)*; Maitha Alqemzi, Hayder Alsaadi, Bilal Elyafawi, Hamda Khansaheb *(Rashid Hospital, Dubai)*; |
| **United Kingdom**: Nour Abdel-Fattah, Rachel Dbeis, Ruari Jardine, Priyanka Kunte, Gianluca Maresca, Stuart McIntosh, Lucy McLeod, Ahmed Nassar, Ashrafun Nessa, Laura Osborne, Peter Osei-Bonsu, William Paine, Jelizaveta Pereca, Shafaque Shaikh, Rachel Vaughan, Kanastana Yasotharan *(Aberdeen Royal Infirmary, Aberdeen)*; Jenny Ferry, Megan Kershaw, Angharad King, Joanna Krawczyk, Cheri Price, Anushka Sieunarine, Andrew Temperton, Vimla Victor, Sophie Walker, Matthew Williams, Thomas Woodhouse *(Nevill Hall Hospital, Abergavenny)*; Andras Farkas, Maria Hobrok *(Bronglais General Hospital, Aberystwyth)*; Rhona Kilpatrick, Matthew Newman *(University Hospital Monklands, Airdrie)*; Aalap Asurlekar, Stephen Dalgleish, Peter Davies, Joseph Farrimond, Elizabeth Lindsay, Joe Littlechild, Mariam Malik, Suad Nimale, Vaila Robertson, Nicole Robin, Wan Nee Shue, Robert Sinnerton *(Stracathro Hospital, Angus)*; Giles Faria, Adam Fell, Ifeanyi Kem Onubogu, Jai Relwani *(William Harvey Hospital, Ashford)*; Oluwaseun Adeboyejo, Mandeep Singh Bindra, Kim Borsky, Muhammad Umar Fawad, Shoaib Fahad Hussain, Fadi Issa, Aiman Jamal, Catriona Luney, Neale Marlow, Aman Sethi, Haleema Siddique, Abin Varghese, James Vaz *(Stoke Mandeville, Wycombe General, Aylesbury)*; Demin Li, Caitlin Rigler, Rajen Tailor *(Horton Hospital, Banbury)*; Matthew Bye, Elorm Daketsey, Jane Elford, Rahela Islam, Rosalind Jones, Tharangani Kathiravan, Sujatha Kumari, Kavitha Mattam, Richard Morris, Daniel Murray, Amy Nixon, Carwyn Roberts, Zoe Seymour, Arshad Siddiqui, Chris Smith, Lianne Stevenson, Odai Yaghi *(Ysbyty Gwynedd, Bangor, North Wales)*; Asem Almaghrebi, Josephine Kahiu, Nikhita Patel *(Barnet General Hospital, Barnet)*; Anish Koneru, Emma Long, Ayesha Rahman, Selina Ravenscroft, Tobias Stedman, Gianina Tutoveanu, Alex Ward, Lucy Wibmer *(Barnsley Hospital NHS Foundation Trust, Barnsley)*; Katie Cross, Mohammed Fakhrul-Aldeen, Kayleigh Spellar *(North Devon District Hospital, Barnstable)*; Shahrukh Ahmad, Joshua Boyes, John Ferns, Matthew Freudmann, Saqib Ghumman, Siddhartha Handa, Abdul Jalil, Manish Kaushal, Panna Patel, Peter Sodde, Sachith Sreenivasan, Sook Han Yee *(Furness General Hospital, Barrow in Furness)*; David Ensor, Mark Gotecha, Shaikh Sanjid Seraj, Premalatha Sharavanan, Anuhya Vusirikala *(Basildon University Hospital, Basildon)*; Rishi Das, Nikhil Ponugoti *(Basingstoke and North Hampshire Hospital, Basingstoke)*; Rosalind Grace Beckett, Soraya Conroy, Joel Corkill, Angharad Davies, Katie Gilmore, Lysander Gourbault, Thomas Parsons, Tobias Roberts *(Royal United Hospital Bath, Bath)*; Beibit Bashabayev, Cristina Croitoru, Emmet Dorrian, Michael McGreevy, Niamh Murphy *(Belfast City Hospital, Belfast)*; Firas Aljanadi, Joseph Doyle, Hashim Elshibly, Reubendra Jeganathan, Mark Jones, Jamal Khan, Maryam Khan, Alexandra Lee, Nyasha Mutsonziwa, Rebecca Reid *(Royal Victoria Hospital, Belfast)*; James Archer, Christopher Bache, Andre Cardoso Almeida, Abdulrahman Odeh, Max Pachl, Rebecca Schembri *(Birmingham children’s hospital, Birmingham)*; Anna Gateley, Victoria Hodgetts Morton, Helen van Vliet *(Birmingham Womens and Childrens Hospital, Birmingham)*; Audrey Kwong, Elaine Leung, Sudha Sundar, Megan Williamson *(City Hospital, Dudley Road, Birmingham)*; Irshad Ahmed, Abdulrahman Alsaggaf, Amarbaj Chandock, Nnaemeka Chidumije, Sharad Karandikar, Tabassum Khan, Arab Rawashdeh, Ali Sallam, Rishi Singhal, Syed Osama Zohaib Ullah *(Heartlands Hospital, Birmingham)*; Ruhina Alam, Yvonne Asiedu, Aneel Bhangu, Alina-Maria Budacan, Shafiq Ahmad Chughtai, Anant Desai, Anvay Deshpande, Mohammed Farid, Samuel Ford, Wiliam Foy, Adam Gittins, Ewen Griffiths, Jonathan Herron, Michael Howells, Rizwana Imran, Tobias James, Sivesh K Kamarajah, Rugved Kulkarni, Tsun Yu Kwan, Catherine Leng, Andraay Leung, Ansar Mahmood, Thomas McLelland, Paul Nankivell, Alessandro Parente, Ishan Radotra, Keith Roberts, Bhaskar Satsangi, Neil Sharma, Manjunath Siddaiah-Subramanya, Muhammad Harris Siddique, Thomas Smith, Wai Cheong Soon, Veena Surendrakumar, Fabio Tirotta *(Queen Elizabeth Hospital Birmingham, Birmingham)*; Salem Jamhour, Dhanyata Narendra, Suresh Panchakshariah, Narendra Babu Siddaiah *(Royal Orthopaedic Hospital, Birmingham)*; Mohamed Albendary, Edward Balai, Alvaro Bedoya-Ronga, Felicia Elena Buruiana, Ali Yasen Y. Mohamedahmed, Lopa Patel, Rajeev Peravali, Shahin Qadri, Miski Scerif, Abdul Sillah, Jenny Wright *(Sandwell General Hospital, Birmingham)*; Laith AlSaket, Jayant Cherukat, Mohamed Saleem Noor Mohamed, Arab Rawashdeh, Rishi Singhal *(Solihull Hospital, Birmingham)*; Nauman Ahmed, Clarissa Ern Hui Fang, Abin Holla, Zayne Mahmud-Ahmad, Mohammad Masaarane, Surya Narayan, Pornjittra Rattanasirivilai, Irena Shiderova, Asma Sultana, Silvian Tan, Hasan Usmani, Osborne Peter Vaz *(East Lancashire Hospitals NHS Trust, Blackburn)*; Jonathan Barker, Mohammed Elniel, Lucy Fell, Fatma Khan, Geerthan Nagachandra, Lara Rimmer *(Blackpool Victoria Hospital, Blackpool)*; Utkarsha Basu, Tsitsi Chituku, Kingsley Lasing, Devaraj M Navaratnam, Milind Rao, Aliaa Shamardal, Athula Tennakoon, Nisar Haider Zaidi, Sadaf Zehra *(Pilgrim Hospital, Boston)*; Thomas Baker, Genevieve Brixton, Rotimi David, Tobias Klatte, Agata Majkowska, Joanna Manson, Ryan Potter, James Yea *(Royal Bournemouth Hospital, Bournemouth)*; Muntadhir Al-uzri, Omar AlShakhshir, Jordan Bickerdyke, Peter Bobak, Victoria Brown, Alexios Dosis, Mohamed Hashem, Max Hayden, Harry Hodgson, Irfan Jumabhoy, Fiona Langlands, Kenneth Linton, Mariana Matias, Amelia Milton, Hannah Louise Morley, Frances Mosley, Christopher Raine, Matthew Rose, Amy Round, David Selwyn, Isabel Slark, Alexander Sumpner, Jonathan Wareing, Sam Wareing, Jessica Watson, Claire Winton *(Bradford Royal Infirmary, Bradford)*; Ashish Gupta, Rahi Karmarkar, Lavinia Margarit, Rucira Ooi, Amr Shalaby, Shahrima Sheefat, Aurelia Vas, Islam Zarad *(Princess of Wales Hospital, Bridgend, Bridgend)*; Mazin Mohamed, Chloé Monnier, Mohamed Radhi *(Royal Sussex County Hospital, Brighton)*; Mai Baquedano, Catherine Bradshaw, Massimo Caputo, Lakith Kapuge, Benjamin Martin, Filippo Rapetto, Mohamed Shalaby, Ella Teasdale *(Bristol Royal Hospital for Children, Bristol)*; Sarah Biggs, Natalie Blencowe, Abigail Campbell, Florence Caslake Holding, Ffion Dewi, Ettorino Di Tommaso, Lauren Dixon, Ysabelle Embury-Young, Gustavo Guida, Kathryn Hogan, Lucy Huppler, Caitlin Jordan, Thomas Andrew Maccabe, Catherine Macleod-Hall, Tina Anto Menachery, Samir Pathak, Ryan Preece, Reesha Ranat, Jonathan Randall, Sophie Rozwadowski, Douglas West *(Bristol Royal Infirmary, Bristol)*; Jack Bellerby, Matthew Chan, James Cullen, Richard Donovan, Tomos Edwards, Rhiannon Frostick, Kate Gregory, George Higginbotham, Kalina Hristova, Kueni Igbagiri, Tarik Jichi, Rhys Luckwell, Sophie McDonald, Joshua Moreau, Dimitri Pournaras, Sarah Shiels, Thomas Tilston, Matt Towner *(Southmead Hospital, Bristol)*; Agnes Hamilton-Baillie, Neil Ryan *(St Michael’s Hospital, Bristol)*; Adewale Ayeni, Najam Husain, Keren Pathmanathan, Olatoyosi Williams *(University Hospitals of Derby and Burton, Burton upon Trent)*; Aneish Mangarai, Ashwini Virgincar *(West Suffolk Hospital, Bury St Edmunds)*; Amina Akhtar, Rohan Ardley, Evripidis Tokidis *(Chesterfield Royal Hospital, Calow)*; John Hardie, Sarmad Kazzaz, Stratton King, Jayson Roberts *(Frimley Health NHS FT - Frimley Park, Camberley)*; Karim Abdelraouf Moawad, Amit Agrawal, Abdulrahman Al-Mohammad, James Ashcroft, Susannah Ashfield, Alan Askari, Anita Balakrishnan, Deepika Bhojwani, Garance Biosse-Duplan, Patrick Coughlin, Nick Dai, Richard Justin Davies, Henry Dunne, Amer Durrani, Michael Feretis, Daniel I. Fernando, Alice Fae Ferreira, Brian Fish, Fanourios Georgiades, Marios Ghobrial, Andreas V Hadjinicolaou, Rob Hammond, Sarah Hardwick, Yaldasadat Hashemipour, Thusitha Hettiarachchi, Victoria Hudson, Amy Huseyin, Peter Hutchinson, Ekpemi Irune, Claire Jackson, Asif Jah, Niel Kang, Wasim Khan, Maaz Khan, Angelos Kolias, Harry Kyriacou, Siong-Seng Liau, Andre Chu Qiao Lo, Samuel Long, Liam Masterson, Claudia Gabriela Mitrofan, Ahmed M. H. A. M. Mostafa, Suzanne Murphy, Robert O’Neill, Reece Patel, Mohamed Rabie, Will Raby-Smith, Siobhan Mairead Rooney, Sabrina Helena Rossi, Neil Russell, Eniola Salau, KT Matthew Seah, Nicholas Segaren, Farakh Shahzad, Arnaldo Neves Santos Silva, Aminder Singh, Jeremy Solly, Harry Spiers, Grant D. Stewart, Kendrick To, Adam Townson, Rebekka Troller, Sara Venturini, Catriona Walker, James Wheeler, Christopher Y.K. Williams, Kai Yuen Wong, Athanasios Xanthis *(Addenbrooke’s Hospital, Cambridge)*; Navid Ahmadi, Olivia Baker, Raisa Bushra, Aman Coonar, Fabio Falconieri, John Hogan, Asanish Kalyanasundaram, Anum Mir, Eyal Ran Nachum, Maria Nizami, Mohamed Osman, Dulan Samaraweera, Daniel Sitaranjan, Christopher Smith, Ismail Vokshi, Sze Hui Wong, Emma Rose Michelle Woolcock *(Royal Papworth Hospital, Cambridge)*; Roa Hassan, Nikhil Math, Mark Yao *(Kent and Canterbury Hospital, Canterbury)*; Saad Azher, Tom Combellack, Natasha Harley, Ghis Tahhan *(University Hospital Llandough, Cardiff)*; Idrees Ahmed, Christopher Bowler, Sarah Choi, Elinor Davies, Victoria Evans, Rhodri Evans, Gemma Harrell, Andrew Hughes, Robert Mcleod, David Owens, Michael Shinkwin, Samuel Stevens *(University Hospital of Wales, Cardiff)*; Tosin Akinyemi, Funbi Ayeni, Khalid Bhatti, Ruben Canelo Professor, Basem Hamzah, Syed Mannan *(Cumberland Infirmary, Carlisle)*; Charlotte Bee, Pawan Kumar Dhruva Rao, Ishtiak Mahamud, Riyam Mistry *(Glangwili General Hospital, Carmarthen)*; Arjun Paramasivan, Gabriela Bocsa, Andrew Brown, Conor Carroll, Bhargavi Chandrasekar, Arthur Day, Sophie Dodd, Nicola Eardley, Emma Ewins, Lucy Fuller, Sukhpreet Gahunia, Parmilan Gill, Joseph Hanna, Tristan Heath, Christopher Khoory, Emily Krishnan, Weisang Luo, Nichola Manu, Leo Maric, Emmeline Martin, Alex Moore, Sunanda Roy Mahapatra, Olivia Lumiap Serevina, Dale Vimalachandran *(Countess of Chester Hospital, Chester)*; Rosanne Ching, Zainab Dhorat, Hal Munton, Dr Shiva S Tripathi *(Lancashire Teaching Hospitals NHS Foundation Trust, Chorley)*; Reem AlAbdulwahed, Amanda Czyz, Adeoye Oluwakanyinsola Debo-Aina, Aniket Deshpande, Hadyn K.N. Kankam, Dana Low, Benjamin Paolini, Aswathy Pavithran, Sunita Saha, Sreelakshmi Suresh, Anjelli Wignakumar *(Colchester Hospital University, Colchester)*; William Campbell, Martin King, Sarah Small *(Causeway Hospital, Coleraine)*; Haneen Abed, Mariam Baig, Phoebe Brobbey, William Carlos, Danny Chandla, Timothy Davis, Fatema Dhaif, Kathryn Dickson, Peter Goodwin, Sarah Henning, David Izadi, David Jeevan, Valdone Kolaityte, Maria Kolokotroni, Nicola Mackay, Adam Pilarski, Jenardan Sellathurai, Smruta Shanbhag, Alastair Stephens, Arthika Surendran, Thomas Ward, Doaa Zeidan *(University Hospitals Coventry and Warwickshire NHS Trust, Coventry)*; Mohammed Balbola, Chi Hoi Lee, Jimmy Mena, Emila Paul *(Leighton Hospital, Crewe)*; Antony Bateman, Ash Bhalla, Hasan Daoud, James Davies, Gareth Davies-Jones, Abdulla Ebrahim, Lucy Gossling, Amit Goyal, Ahmed Hamad, Hannah Javanmard-Emamghissi, Anuja Joshi, Joanne McKay, Emma Ogden, Ziyan Sheng, Lavanya Umashankar, Lotte Weenink, Emily Weisfeld *(Royal Derby Hospital, Derby)*; Emmanuel Adebajo, Sorour Borayek, Mark Cribb, Nabil Elmaleh, Emily Heywood, Omar Mulla *(Doncaster Royal Infirmary, Doncaster)*; Omar Ahmed, Tasleem Akhtar, Kerrie Aldridge, Vishal Amin, Jonathon Dawson, Rachael Dolan, Zi Hao Reuel Heng, Alexander Hollis, Akash Jangan, Jo Lau, Aamer Mughal, Jeremy Newman, Rachel Olive, Shayan Shahidi, Atif Sharif, Julian Sonksen, Arooj Syed, Isaac Wahnon, Michael Wall, Stephanie Weedon *(Russell’s Hall Hospital, Dudley)*; Jeyakumar R Apollos, Megan Kerr, Muneeb Zafar *(Dumfries and Galloway Royal Infirmary, Dumfries)*; Sheng wei Chiam, Stephen Davison, Laura Doherty, Christopher Donoghue, Alastair Faulkner, Yong Kiat Goh, Katie Hoban, Jeremy Lee Jun Shern, Alasdair MacInnes, Jaiganesh Manickavasagam, Rebecca McNicol, Sudhara Niriella, Eimear O’Connell, Ramaa Parulekar, Kalpana Ragupathy, Mohammad Rahman, Arthur Chen Wun Tan, Kimberly Tiang *(Ninewells Hospital, Dundee)*; Lynn Darragh, Jennifer Foreman, Ian McAllister, Cherith Semple *(Ulster Hospital Dundonald, Dundonald)*; Sathyaseelan Arumugam, Tom Collicott, Gargeshwari Krishnamurthy Guru Raghavendra, Khizar Khan, Cho Ee Ng, Irvita Sharma, Shreyas Supparamaniam, Sabina Wallace-King *(University Hospital North Durham, Durham)*; Paul Cullis, Beniamino Forte, Phillip Holt, Merrill McHoney, Abigail Semple *(Royal Hospital for Sick Children, Edinburgh)*; Caitlin Brennan, Paul Brennan, William Cambridge, Thomas Drake, Andrew Durden, Mark Galea, David Henshall, Gustav Linder, Richard McGregor, Joshua McIntyre, Gabriel Metcalf-Cuenca, Samuel Molyneux, Joel Norton, Jay Jaemin Park, Alexandra Rice, Lydia Robb, Lauren Ross, Cameron Simpson, Richard JE Skipworth, Gordon Snowden, Andrew Tambyraja, Daniel Thompson, Gareth Turnbull, Marianne Watters, Tim White *(Royal Infirmary of Edinburgh, Edinburgh)*; Connor Boyle, Filip Brzeszczyński, Katherine Hodge, Alexander Laird, Yasuko Maeda, Hugh Paterson, Scott Smith, Peter G Vaughan-Shaw *(Western General Hospital, Edinburgh)*; Mariam Ahmed, Asuvathan Aravinthan, Markus Baker, Zachary Baxter, Miles Benjamin, Anupama Jeyakumar, William Lloyd, Mana Rahimzadeh, Nithin Thoppuram *(Epsom & St Helier University Hospitals NHS Trust, Epsom)*; Katerina Anesti, Morgan Bayley, Eleanor Burden, Joanne Cozens, Urszula Donigiewicz, Douglas Ferguson, Alex Goubran, Thomas Hubbard, Wei Jia, Matthew Jukes, Edward David Lumley, Lisa Massey, Frank McDermott, John McGrath, Aye Myintmo, Michael Ng, Katie-Louise Parker, Jonathan Phillips, Heather Pringle, Niroshini Rajaretnam, Amin Siddig, Oliver Small, Guang Yim *(Royal Devon and Exeter Hospital, Exeter)*; Sadia Afzal, Kais Al Suyyagh, Gemma Faulkner, Michael Greenhalgh, Ghazal Hodhody, Joann Lum, Olivia McCabe-Robinson, Joseph McKay, William Ryan, Bethan Salmon, Chloe Smart, Henry St Aubyn Bilton, Matthew Walmsley, Patrick Watmough, Leo Watton *(Royal Bolton Hospital, Farnworth)*; Barry Dent, Hannah Emerson, Porfyrios Korompelis, Wendy McCormick, Stuart Rundle, Kiran Singisetti, Karen Vejsbjerg, Cara Vincenti, Thomas Watkinson, Benjamin Wormald *(Gateshead Health NHS Foundation Trust, Gateshead)*; Daniel Carson, David Holroyd, Nigel Jamieson, Marina Nagiub, Jonathan Pugh, Rachel Thomas *(Glasgow Royal Infirmary, Glasgow)*; Rachael Boardley *(New Stobhill Hospital, Glasgow)*; Elsie Bridgman, Mustafa El Sheikh, Simon Lammy *(Queen Elizabeth University Hospital, Glasgow)*; Mark Danton, Ciaran Doherty, William Flynn, George Gradinariu, Ed Peng *(Royal Hospital for Sick Children, Glasgow)*; Joseph Atley, Molly Bradbury, Simon Dwerryhouse, Vipul Garg, Rajesh Gopireddy, Lydia Newton, Tsi Njim, Muni Pinjala, Manuk Wijeyaratne *(Gloucestershire Royal Hospital, Gloucester)*; Alaa Awad Hussein Ameri, Bence Atkari, Gakul Bhatta, Monica Bogdan, Christopher Chien Liang Liao, Joe Hwong Pang, Laura Parry, Vamsi Velchuru *(James Paget Univeristy NHS Foundation Trust Hospital, Great Yarmouth)*; Mei ying Chin, Abi Hayward, Salasiah Othman, Anna Sayers, Soorya Siva *(Inverclyde Royal Hospital, Greenock)*; Ahmed Abubakar, Claudia Dyball, Manal Javid, Taran Khurana, Kar Yeung Kenneth Ko, Stephen McAleer, May Ting Tan *(Diana Princess of Wales Hospital Grimsby, Grimsby)*; Jonathan Horsnell, Thumuluru Kavitha Madhuri, Emily Moore, Isabella Murray, Jamie Patel, Alex Tan *(Royal Surrey County Hospital, Guildford)*; Farah Akthar, Emily Crane, Rishi Dhir, Praveen Gopinath, Sofia Green, Mohamed Habad, Salah Hammouche, Dalia Hammouche, Manal Jmaileh, Tjun Wei Leow, Htoo Lu, Jehangir Mahaluxmivala, Qasim Sohail, Catrin Sohrabi, Simon Wimsey *(Princess Alexandra Hospital, Harlow)*; Oliver Cohen, Christopher I’Anson, Matthew Wright *(Harrogate District Hospital, Harrogate)*; Hani B Abdul-Jabar, Sibusiso Ndlovu, Matthew Reid, Louie Lynn Sajorda, Muhammad Harris Siddique, Abhinav Singh *(Northwick Park Hospital, Harrow)*; Cosimo Alex Leo, Carolynne Vaizey, Janindra Warusavitarne *(St Mark’s Hospital, Harrow, London)*; Lucy Maling, Sheena Seewoonarain, Christopher To *(East Sussex Healthcare (Conquest hospital and Eastbourne District General Hospital), Hastings)*; Abdelmonem hassan eid Abdelmonem, Mustafa Al-Yaseen, George W V Cross, Krisztian Deierl, Vivek Dubey, Ahmad Ja’far, Riana Patel, Sybghat Rahim, Parisah Seyed-Safi, Yat Wing Smart, Emad Soliman, Jija Thomas *(West Hertfordshire Hospitals NHS Trust, Hertfordshire)*; Phillipa Beesley, Michael Biggs, Peter Davies, Laura Deacon, Laura Garden, Sean Howells, Megan Oldbury *(Huddersfield Royal Infirmary, Huddersfield)*; Faazil Ahmed, Shereen Ajab, Vasileios Arzoglou, Alice Ball, Alison Cairns, Megan Chrysikopoulou, Amit Deshmukh, Ghazi Elshafie, Momin Eltayeb, Alasdair Findlay, Francesca Gatta, Mithila Govind, Jessica Harvey, Benjamin Huggon, Shah Jehan, Navid Kabuli, Eiman Khalifa, Sameera Khaliq, Mahmoud Loubani, Matthew Robert Marples, Rhona Martin, Adam McClean, Gautam Modak, Shahani Nazir, Sandhya Od, Andrew Parrish, Abirami Perumal Kanniappan, Andrew Quin, Piravin Kumar Ramakrishnan, Ritika Rampal, Brianda Ripoll, Razan Rislan, Robert Taylor, Dwarakesh Thalamati, Joshua Totty, Ben Ward, Arun Watts, Alex Wilkins, Jih Dar Yau *(Hull University Teaching Hospitals NHS Trust, Hull)*; Shobhit Arya, Adeela Ashraf, Aman Bhargava, Shamira Hassan, Elizabeth Kmiotek, Suzette Samlalsingh *(King George Hospital, Ilford)*; Georgios Karagiannidis, Evangelos Mallidis, Fahed Youssef *(Ipswich Hospital, Ipswich)*; Tariq Alhammali, Pamela Dawson, Waleed Fahmy, Benjamin Gowers, Naren Kumar, Hannah Lennox-Warburton, Michelle Lynch, Adam Mohammad, Salam Musa, Arnav Sahu, Sharan Sambhwani, Nomaan Sheikh, Ormond Mark Taylor, Michelle Wright *(Kettering General Hospital, Kettering)*; Marwa Al-Azzawi, Nicola Hall, Michael Helley, Iain Hood, Emma Howie, Shannon Jordan, William Norton, Charlie Saunders, Duncan Stickle, Euan Toshney, David Watt, Andrew Whitfield *(Crosshouse University Hospital, Kilmarnock)*; Ahmad Al-Shaye, Ahmad Al-Sukaini, Ahmad Attia, John Britton, Theo Clarke, Simona Ippoliti, Ignatius Liew, Gabriella Weisz, Ampere Yiu, Hesham Youssef *(Queen Elizabeth Hospital King’s Lynn, King’s Lynn)*; Areej Abdel-Fattah, Muhammad Adeel Akhtar, Hwan Heo *(Victoria Hospital Kirkcaldy, Kirkcaldy)*; Mhairi Clark, Gareth Clarke, Benjamin Parkin, Fraser Rae, Calum Sreenan, Roxane Stienstra, Lindsay Wallace, Michael Wilson *(Forth Valley Royal Hospital, Larbert)*; Salman Arif, Anupama Barua, Michael Blackwell, Grainne Bourke, Conor Bowe, Mahbub Chowdhury, Elliott Cochrane, Raquel Constantino-duarte, Sophie Earl, Ikechukwu Ejiofor, Walid Elmahdy, Betsy Evans, Antonella Ferrara, Alice Fort-Schaale, Claire Hardie, Michael Ho, Azar Hussain, Neeraj Kalra, Anastasios Kanatas, Chung Yan Vernon Lee, Stefan Louette, Ryan Mathew, Stephanie Milne, Emily Salt, Priya Shankar, Alexander Spyridoulias, Peter Szedlak, Josh Twigg, Ryckie Wade, Christopher West, James Wright *(Leeds General Infirmary, Leeds)*; Antonios Athanasiou, Adam Barlow, Elaine Borg, Luke Bradshaw, Alessandro Brunelli, Abbigayle Buckton-Perkins, Gemma Burdge, Eleanor Clarke, Peter Coe, Camilla Davies, Elizabeth Doxford-Hook, Sheila Fraser, Joanna Gibson, Andrea Giorga, Konstantinos Gioutsos, Sunjay Jain, Angelika Kaufmann, Alexandros Laios, Susanna Lam, Terence Lo, Marie Lucas, Emma MacInnes, Sushil Maslekar, Catherine Moriarty, Christopher Nahm, Mohamed Otify, Adam Peckham-Cooper, Cecilia Pompili, Charmian Reynoldson, Phoebe Robertson, Loren Rushton, Lucy Scott, Nanaki Singh, Helen Skinner, Giles Toogood, Danielle Trigg, Louise White, Jessica Whitney, Robert Wickstead, Alastair Young *(St James’s University Hospital Leeds, Leeds)*; Shahbaz Abdullah, Metesh Acharya, Nina Al-Saadi, Keng-Leong Ang, Rebecca Boyles, Edward J Caruana, Karishma Chandarana, Ravi Chotalia, Mohammed Fiyaz Chowdhry, Rachel Chubsey, Chiraag Thakrar Karia, Emmanuel Katsogridakis, Marinos Koulouroudias, Kudzayi Kutywayo, Kaylan Lad, Dominic Lam, Giovanni Mariscalco, Rayhaan Mohammed, Apostolos Nakas, Anishka Pabari, Shriyam Patel, Sridhar Rathinam, Athanasios Saratzis, Matthew Stephens, Evangelia Ioanna Tsiourva, Mustafa Zakkar *(Glenfield Hospital, Leicester)*; Alison Armstrong, Robert U Ashford, Wen Jie Chin, Srujan Rajesh, Oliver Lauridsen Siaw, Baljit Singh *(Leicester General Hospital, Leicester)*; Alex Boddy, Vijay Chavda, Neil Flint, Irina Georgieva, Mark Higgins, Jitendra Mangwani, Prashant Naik, Ninad Nigalye, Jvalant Nayan Parekh, Kathleen Wolff *(Leicester Royal Infirmary, Leicester)*; Cameron Kuronen-Stewart, Beatrix Weber *(Gilbert Bain Hospital, Lerwick)*; Zubair Ahmed, Joseph Attard, Amirah Azhar, Nikhil Kulkarni, Lydia Prusty, Dinesh Thekkinkattil *(Lincoln County Hospital, Lincoln)*; George Garas, Peter Gaskell, Asif Hasan, Terence Jones, Arjun Kattakayam, Christopher Loh, Raimundas Lunevicius, Natalie Maple, Stylianos Papalexandris, Shirley Pringle, Alasdair Santini, Andrew Schache, Richard Shaw, Ajay Sud *(Aintree University Hospital, Liverpool)*; Harriet Corbett, Samuel Ebbs, Sian Falder, Margaret Hanley, Rachel Harwood, Rong Khaw, Anna McNamara, Ijeoma Okonkwo, Arvind Rammohun *(Alder Hey in the Park, Liverpool)*; Muhammad AbdulHakeem, Syed Al Nahian, Mark Field, Amer Harky, Ronan Kelly, Bilal Kirmani, Abinash Panda, Florentina Luiza Popescu, Mostafa Snosi, Ahmed Torky, Mauin Uddin, Paulino Nathaniel III Zamesa *(Liverpool Heart and Chest Hospital, Liverpool)*; Shakil Ahmed, Kiran Altaf, Ryan Baron, Hannah Barrow, Philip Cornford, Declan Dunne, Hannah Earnshaw, Raja Eid, Jeremy Guilford, Lydia Hawker, Stephen Kaye, Rebecca Lenihan, Rachel McKinney, Luca Pagano, Rohith Rao, Tom Rayner, Vito Romano, Susannah Shore, Dana Sochorova, Robert Sutton, Francesco Torella, Thomas Ward, Chloe Ward *(Royal Liverpool University Hospital, Liverpool)*; Simon Clark, Tarek Elmoslemany, Michael Jenkinson, Kevin Kelly, Vijay Kumar Kumar, Chris Lemos, Talanayar Mahalingam, Christopher Paul Millward, Vijayam Priya Nair, Vinita Sangai, Sarbpreet Sarao, Rajesha Srinivasaiah, Sudhir Venkataramaiah *(The Walton Centre NHS Foundation Trust, Liverpool)*; Dominique Hughes, Luca Lancerotto, Lucy Li *(St Johns Hospital, Livingston)*; Madlen Dewi, Eshan Mazumdar, Dema Motter, Jennifer Reid *(Royal Glamorgan Hospital, Llantrisant)*; Tony Chen, Isobel Hatrick, Holly Lyle, Freyia Mahon-Daly *(Charing Cross Hospital, London)*; Niarah Ahmad, Guy Benshetrit, Zoe Burdon, Rupen Dattani, Nina Jyne Minette Dela Cruz, Jordan Faulkner, Madeleine Garner, Catrin Morgan, Rebecca Morgan, Toby Noton *(Chelsea and Westminster Hospital, London)*; Tanzeela Gala, Tom Georgi, Christopher Mifsud, Sujan Patel, Rose Stahl, Stella Vig *(Croydon University Hospital, London)*; Muhammad Usman Ali, Sarah Kelly, Lokesh Lokesh, Michaela Paul, Daniel Shaerf, Hemant Sheth, Fiammetta Soggiu *(Ealing Hospital, London)*; Charlotte Holbrook, Ijeoma Nwachukwu *(Evelina London Children’s Hospital, London)*; Katie Adams, Maame Aduse-Poku, Tamara Alexander, Gill Arbane, James Arlidge, Craig Bailey, Melissa Baldwin, Heena Bidd, Aina Brunet-Garcia, Gary Colville, Alexa Curtis, Christopher Delaney, Yasser Diab, Toby Dixson, Mothana Gawad, Siew-Ling Harrison, Christopher Holt, Craig Johnstone, Findlay MacAskill, Sarah McCrindle, Kate Millar, Osama Naji, Rafal Niziol, Leyla Osman, Arun Sahai, Abegail Salvana, Ahmad Sayasneh, Michael Shaw, Sarah Tian, Annabelle White, Danny Jon Nian Wong, Valerie Wong *(Guy’s and St Thomas’ Hospitals, London)*; Christina Fotopoulou, Jennifer Ploski, Srdjan Saso *(Hammersmith Hospital, London)*; Katina Theodoropoulou, Alexandra Valetopoulou *(Homerton University Hospital, London)*; Keyoumars Ashkan, Kathleen FM Fan, Angela Hancock, Josephine Jung, Maria Alexandra Velicu, Zoe Williams, Elizabeth Yeung *(King’s College Hospital, London)*; John Dabis, Ryan Geleit, Ian Gill, Thomas Holme, Nardeen Kader, Irfan Merchant, Akarshan Naraen, David Rawaf, Nashat Siddiqui *(Kingston, London)*; Qurrat Al Ain Atif, Rory Cuthbert, Cameron Dott, David Ferguson, Lana Huang, Priyanka Iyer, So Hee Kim, Srinath Ranjit, Varun Sarodaya, Lucy Tomasetti, Tayyub Yasin *(Newham University Hospital, London)*; Syed Zain Bukhari, Mamun David Dornseifer, Manojkumar S Nair *(North Middlesex University Hospital, London)*; Mohit Bhatia, Basel Haddadin, Rehana Hafeez, Ahmed Mansy, Ahmed Mattar, Frank Smedley *(Princess Royal University Hospital, London)*; Shihab Chowdhury, Brian Davidson, Nikolaos Dimitrokallis, Giuseppe Fusai, Jack Gilliland, Camila Hidalgo Salinas, Ioannis Kostakis, Reza Mirnezami, Reza Motallebzadeh, Krishnakumure Patel, Helen Quah, Massimo Varcada *(Royal Free Hospital, London)*; Anam Anzak, Abhirup Banerjee, Louis Boyce, Rhiannon Brignall, Laura Clementoni, Evangelos Efthimiou, Sarah Epton, Martina Faimali, Despoina Iakovou, Jayan Dewantha Jayasinghe, Inês Leal Silva, Kaifeng Liang, Cortland Linder, Annamaria Minicozzi, Lalin Navaratne, Zoi Nikoloudaki, Henry Nnajiuba, Mohamed Hazem Okail, Elliot Onochie, Sankhya Prakash Vel, Rajesh Sivaprakasam, Catrin Sohrabi, Mohamed Adhnan Thaha, Bhaskar Thakur, Kalpesh Vaghela, Rosie Yeoward, Vincent Yip *(Royal London Hospital, London)*; Sibtain Anwar, Teofila Bueser, Aung Oo, Julie Sanders, Ashley Thomas, Marta Wachtl, Mateusz Zawadka *(St Bartholomew’s Hospital, London)*; Mohamed Abouelazayem, Raluca Belchita, Caroline Hing, Nicholas Judkins, Antonio Leyte Golpe, Preemal Patel, Gowthanan Santhirakumaran, Jeremy Smelt, Carol Tan, Dimitrios Tsironis *(St George’s Hospital, London)*; Anita Eseenam Agbeko, Joachim Amoako, Chang Park, Khaled Sarraf, Joseph Shalhoub, Kapil Sugand *(St Mary’s Hospital, London)*; Jochem Caris, Hemant Kocher *(The London Clinic, London)*; David Choi, Ciaran Hill, Chan Hee Koh, Hugo Layard Horsfall, Hani Marcus, William Muirhead *(The National Hospital for Neurology and Neurosurgery, London)*; Poppy Alport, Peter Barry, Charlotte Benson, John Butler, Hannah Dawson, Paula Fagan, Jonathan Hannay, Andrew Hayes, Anna Heeney, Robin Jones, Cyrus Kerawala, Erik Mayer, Anissa McClelland, Rachel O’connell, Edward Phillips, Gausihi Sivarajah, Myles Smith, Alannah Smrke, Dirk Strauss *(The Royal Marsden NHS Foundation Trust, London)*; Calisha Allen, Ivy Lim, Max Little, Chetan Parmar, Alexandra Sharpe, Benjamin Zakaria *(The Whittington Hospital, London)*; Nicholas Kalavrezos, Natalie Marzouqa, Deepti Sinha *(University College London Hospital, London)*; Tom Abbott, Stefano Andreani, Michael Bath, Mona Behravesh, Brendan Berry, Emma Collins, Isabella Drummond, Isabelle Farrow, Ora Jesner, Stephanie Kwok, Valerie Lan-Pak-Kee, Sriveena Naganathar, Patrick O’Hagan, Amar Odedra, Funlayo Odejinmi, Christine Ozone, Laura Roberts, Lesley Sheach, Matthew Short, Shawn Jia Hwang Tan, Zoe Thursz, Charlotte Trainer, Zaker Ullah *(Whipps Cross University Hospital, London)*; Md Tanveer Adil, Safia Ahmed, Chelise Currow, Jonathan Dennis, Alistair Hardy, Haidee Harrison, Anna Kaleva, Aneesah Khan, Shantata Kudchadkar, Minil Patel, Meysoon Qurashi, Amit Raithatha, Jayesh Sagar, Rishi Talwar, Ali Tarfiee *(Luton and Dunstable University Hospital, Luton)*; Ziad Al-Khaddar, Farhat Naaz Amir, Mumtaz Bughio, Fares Ftaieh, Wut Hmone, Andrew Kennedy-Dalby, Soumitra Mukherjee, Sara Nausheen, Christopher Smart *(Macclesfield District General Hospital, Macclesfield)*; Dinesh Balasubramaniam, Balaji Jayasankar, Eoin O’Farrell, Mahmud Riad *(Tunbridge Wells Hospital, Maidstone)*; Louai Abdeh, Saravpreet Ahluwalia, Kailash Bhatia, Natalie Cheyne, Haris Naseem, Gopalkrishna G Verma, Eilidh Waddell, Moez Zeiton *(Manchester Royal Infirmary, Manchester)*; Numera Arif, Nabeel Cheema, Usman Halim, Charlie Moret, Paul Peters, John Michael Ranson, Jigar Shah, Andrew Wheelton *(North Manchester General Hospital, Manchester)*; Ethan Clough, Henry de Berker, Sara Faily, Paul Farrelly, Helen Figgins, Ana Jeelani, Matthew Murphy, Jaya Nichani, Lucinda Pigott, Mamta Shah, Gabrielle Thompson, Jonathan Yates, Jade Chen Zhao *(Royal Manchester Children’s Hospital, Manchester)*; Sujesh Bansal, Kailash Bhatia, Swarnendu Dey, Ellie Humphry, Rahul Norawat, Noha Tageldin *(St Marys, Manchester)*; Mohammed Abdelaziz, Moslem Abdelghafar, Ikenna Anderson Aneke, Thomas Bradley, Benjamin Brown, Katie Campbell, Charles Carey, Daniel Coakley, Natasha Elson, Verity Haffenden, Sam McNally, Shahd Mobarak, Emma Murray, Bradley Pittam, Michael Preston, Carlo Ross, Safdar Sarwar, Md Abu Sayed, Nazanin Shahrokhi, Catriona Shenton, Sahiba Singh, Anuradha Venugopal *(Wythenshawe Hospital, Manchester)*; Ashwin Bhadresha, Chu-Hao Chiang, Sean Garcia, Rakesh Koshy, Mubasher A Qamar *(Queen Elizabeth the Queen Mother Hospital Margate, Margate)*; John Adam, James Hadfield, Igor Maleyko, Amar Rangan, Andrea Sangheli, Srivishnu Thulasiraman, Anil Varma, Yks Viswanath *(James Cook University Hospital, Middlesbrough)*; Alexander Martin, Sotiris Mastoridis, Chin Hang Sophia Sin, Anjana Singh, Rachel Soulsby, Amanda Taylor, Wai Huang Teng, Ruben Thumbadoo *(Milton Keynes University Hospital, Milton Keynes)*; Junaid Aamir, Mustafa El-lami, Mandeep Koundu, Louise Le Blevec, Ebrahim Mahomed, Connor McGladdery, Samuel Newman, Lysia Richmond *(Royal Lancaster Infirmary, Morecambe)*; Francesco Abbadessa, Corey Chan, Nicholas Dawe, Chathuranka De Silva, Mariantonia Ferrara, John Hammond, Roxane Hillier, Mustafa R Kadhim, John Moir, Stephanie Myszkowski, Thomas Salisbury, Peter Sudworth, Samuel Tingle, Timothy Williams *(Newcastle Upon Tyne Hospitals NHS Foundation Trust, Newcastle upon Tyne)*; Lauren Best, David Bosanquet, Amisha Cameron, Ryan Chiang, Natalie Duric, Arthur Ee, Sarah Elgarf, Hawys Freeman, Tracy James, Dominic Knight, Owen Lewis, Steffan Morgan, Ellie Morgan, Alexandra Munteanu, Setthasorn Zhi Yang Ooi, Sajitha Parveen, Filip Sianos, Ishani Sinha, Sumi Surendran, Tamas Szakmany, Harriet Whewell *(Royal Gwent Hospital, Newport)*; Dimitrios Angelou, Rachael Crompton, Maebh Doohan, Amy Doran, Jonathan Hagan, Katie McCaughey, Mark McKeever, Maria Pantelidou, Reem Salman, Samar Shalaby, Richard Thompson *(Daisy Hill Hospital, Newry)*; Avinash Aujayeb, Rebecca Critchley, Benjamin Emmerson, John Guirguis, Karl Jackson, Nicole McLaughlin, Anna Porter *(Northumbria NHS Hospital Trust, North Shields)*; Ebru Freed, Marcu Vasilica *(Northampton General Hospital, Northampton)*; Dinnish Baskaran, Oliver Claydon, Rachael Collins, Raashad Hasan, Zahra Hussain, Ahmar Iftikhar Talib, Jian Shen Kiam, Vasileios Kouritas, George Lafford, Sam Norton, Joshua Ozua, Aravindh Ramalingam, Haisam Saad, Sari Samman, Bhavesh Tailor, Teri Toi, Daniel Watts *(Norfolk and Norwich University Hospital, Norwich)*; Sara Alsaad, Pritpal Aujla, Nathan Burnside, Sarah Cadwell-Sneath, Zoe Chia, Michelle L Collins, Simon Craxford, Nivedita Das, Brett Doleman, Ahmed M El-Sharkawy, Nathalie Fennell, Ketankumar Gajjar, Catherine Grundy, Karen Hassell, Mohammad Hawari, Adeel Ikram, Trisha Kanani, Harmanpreet Kaur, Zubair Khanzada, Maisie Lau, Ben Marson, Alastair Morton, Francis Githae Muriithi, Benjamin Ollivere, Simon Parsons, Oliver Pumphrey, Rebecca Rollett, Laura Sandland-Taylor, Kathryn Steele, Muhammad Sarmad Tamimy, Elena Theophilidou, Nathan Tyson, Ravinder Vohra, Rini Vyas, Sven Watmore, Helen Weaver, Lisa Whisker, Fady Yanni *(Nottingham City Hospital, Nottingham)*; Asad Abbas, Alfred Adiamah, James Bailey, Ayan Banerjea, Balamurali Bharathan, James Blackwell, Catherine Boereboom, Hilary Brewer, Iain Cameron, Abeed Chowdhury, Christopher Deacon, Charis Demetriou, Edward Dickson, Andrew Edwards-Bailey, Jonathan Evans, Sarah Fendius, Laurence Glancz, Dhanwant Gomez, Gordon Gregory, David Humes, Adeel Ikram, Jamaall Jackman, Aseel Khanfer, Amanda Koh, Christopher Lewis-Lloyd, Ben Marson, Neil Mathias, Eyas Mohamed, Daniel Morris, Yulanda Myint, Gael R Nana, Yasar Nassif, Alex Navarro, Jimmy Ng, James Chean Khun Ng, Alan Norrish, Benjamin Ollivere, Olamide Oyende, Mohammed Patel, Melroy Rasquinha, John-Joe Reilly, Sudip Sanyal, Jaspreet Kaur Seehra, Anneka Shah, Paul Thomas, Kathryn Thomas, Amari Thompson, Benjamin Varghese, Dawit Worku, Fadzlien Zahari *(Queens Medical Centre, Nottingham)*; Akshath Adapa, George A. Antoniou, Mohammed Ashrafi, Bhalchandra Bhalerao, Daniel Cohen, Daniel Doherty, Matthew Gray, Tom Havenhand, Ioanna Kyrou, Siddharth Lokanathan, Ashwani Nugur *(Royal Oldham Hospital, Oldham)*; Thomas Banks, Manikandar Srinivas Cheruvu, Paul Cool, Debashis Dass, Salam Ismael *(Robert Jones and Agnes Hunt Orthopaedic Hospital, Oswestry)*; Helen Beech, Matthew Byrne, Pengchi Chen, Neel Doshi, Stefanos Gorgoraptis, Ali Asgar Hatim Ali, Lamiese Ismail, Jerocin Vishani Loyala, Sotiris Mastoridis, Naomi Neal, Benjamin Ng, Orna Ni Bhroin, Muna Patell, Omar Ben Forge Risk, Hooman Soleymani majd, Stuart C Winter, Ho Ying Flora Wong *(Churchill Hospital, Oxford)*; Roshneen Ali, Abdur-rafee Ameen, Elizabeth Belcher, Christopher Bretherton, Hamez Gacaferi, Mario Ganau, Matej Goricar, Anne Hughes, Deva Jeyaretna, Gs Jutley, Nikhil Lal, Balint Viktor Lovasz, Chris McKinnon, John McNamara, Benjamin Ng, Claudia Paul, Puneet Plaha, Ganeshan Ramsamy, Karishma Shah, Sanskrithi Sravanam, Vikas Sud, Max van Essen, Kate Wallwork *(John Radcliffe Hospital, Oxford)*; Mohamed Hassan Fathy Hassan Abdallah, Sher Aslam, Khurram Ayub, Bhuvanshyam Bhaktavatsalam, Joanna Carvalho, Benjamin Dean, Stevan Jordan, Richard Myatt, Jonathan Norris, Muhammad Ather Siddiqi, Fraser Thomson *(Nuffield Orthopaedic Centre, Oxford)*; Ala Alasadi, Ellen Groundwater *(Royal Alexandra Hospital, Paisley)*; Carina Banziger, Jamie Deyell, Lauren Donnelly, Thomas Samuel William Greensmith, Thomas Harding, Laura Inglis, Dominic Lee, Esther Oyewusi, Rachel Pennington, Christie Hok Yung Shum, Zack Slevin, Ryan Tan *(Perth Royal Infirmary, Perth)*; Shivanie Acharya, Pantelis Amditis, Sanjay Asopa, Supriya Balasubramanya, Aladdin Bashir, Maham Khan, Sathya Lakpriya, Joshua Lau, Rachel Marshall-Roberts, Douglas Miller, Sarah Miller, Nader Moawad, Hossam Nawara, Hope Nwinee, Luke Rogers, Sera Sarsam, Simran Singh, Sebastian Smolarek, Zaheer Tahir, Andrei Tanase, Aye Chan Thu, Zuzanna Wieczorek, Lu Yao *(Derriford Hospital, Plymouth)*; Ioannis Biliatis *(Poole Hospital, Poole)*; Lara Armstrong, Jessica Lockhart, Kevin McElvanna *(Craigavon Area Hospital, Portadown)*; James Ashbridge, Fanny Belais, Janet Berry, Nadine Di Donato, Matthew Dipper, Jim Khan, Charlotte Parfitt, Samuel Stefan *(Queen Alexandra Hospital, Portsmouth)*; Rajeev Advani, Stephen Canty, Madhu Chaudhury, Mariya Dunbobbin, Leanne Dupley, Rukmini Ghosh, Isabel Hughes, Priyatma Khincha, Ana Pardilho, Edward Parkin, Pradnya Patkar, Adrian Pearce, Isaac Phang, Kazim Riaz, Ioannis Sarantitis, S Ali Raza Shehrazi, Paul Turner *(Royal Preston Hospital, Preston)*; Fitzgerald Anazor, Sarah Brown, Radhika Chadha, Emily Crawley, Sofia Farina, Joseph Fennelly, Jiali Gao, Avadhut Kulkarni, Louise Kuo, Mariam Lami, Francesca Lewis, Mark Maher, Gianfranco Messina, Dilip Nair, Gregory Neal-Smith, Bryony Peiris, Hannah Sellars, Harriet Shearman *(Royal Berkshire Hospital, Reading)*; Sophie Bird, Umer Chaudhry, Najeed Khan, Harshadkumar Dhirajlal Rajgor *(Alexandra Hospital, Redditch)*; Ajay Belgaumkar, Zain Elahi, Anum Ghani, Prabhat Prakash Narayan, Anu Sandhya *(East Surrey Hospital, Redhill)*; Mostafa Abdelkarim, Faizah Ali, Tanushree Dewan, Nicola Gallagher, Ahmed Hassan, Karishma Khan, Maisoon Matareed, Sreedutt Murali, Bhaven Murji, Ketevan Papidze, Gowtham Sundaram Venkatesan, Lola Tillson-Hawke, Gireesha Tudawe *(Glan Clwyd Hospital, Rhyl)*; Bashar Abdeen, Syed Nayyar Afaque, Saad Bilal Ahmad, Aishah Ahmed, Aoun Ali, Mohamed Amin, Laura Cervini, Saeef Haque, Ken Weixing Ho, Samrat Mukherjee, Shivakumar Shankar, Lilanthi Wickramarachchi, Yu Hsuen Yang, Hafsa Younus *(Queen’s Hospital Romford, Romford)*; Matthew Hampton, Kiran Madhvani *(Rotherham District General Hospital, Rotherham)*; Majed Al Najjar, Dinesh Alexander, Fayez Almari, Samer Bitar, Ruth Chelva, Daniel Fountain, Abdullah Gabr, Mohamed Galhoum, Abdullah Hanoun, Kenneth Koo, Christina Lipede, Abdul Madni, Musheer Hussain Mohamed, Zaf Naqui, Omar Pathmanaban, Ajay Radhakrishnan, Ashwanth Ramesh, Kohila Sigamoney, Tricia Tay, Richard Unsworth *(Salford Royal Hospital, Salford)*; Graham Branagan, Victoria Morrison-Jones, Navamayooran Thavanesan *(Salisbury NHS Foundation Trust, Salisbury)*; Muzaffar ahmad Ahmad, Nur Amalina Che Din, Sabeen Majid, Ugam Shah *(Scunthorpe General Hospital, Scunthorpe)*; Yahia Al-Tamimi, Andrew Bacon, James Catto, Yasin El-Wajeh, Mairéad Kelly, Ricardo Mohammed-Ali, Ola Rominiyi, Sanad Saad, Saurabh Sinha, Oliver Tame *(Royal Hallamshire Hospital, Sheffield)*; Dylan Chew, Martha Fatima Irene De La Cruz Monroy, Anna-Victoria Giblin, Richard Jackson, Rathan Jeyapalan, John Kiely, Cieran McGrory, James Murray, Jaydip Ray, Daniel Tadross, James Tomlinson, Alex Ward *(Sheffield Teaching Hospital NHS Foundation Trust, Sheffield)*; Yen Nee Jenny Bo, Mohammad Iqbal, Aarti Lakhiani, Guleed Mohamed, William Parry-Smith, Banchhita Sahu *(Shrewsbury and Telford Hospitals, Shrewsbury)*; Aya M Abbas, Naffis Anjarwalla, Jihène El Kafsi, Alasdair Gordon, Samer Jallad, Ehab Kahka, Snigdha Komatineni, Mehran Lashari, Julia Lord, Diana Marujo, Rajesh Midha, Mark Medhat Mikhail, Mafdi Mossaad, Arjun Patel, Tanvir Rafe, Anthony Rayner, Sawsan Saeid, Nilofar Samadi, Hao Meng Yip *(Frimley Health NHS FT - Wexham Park, Slough)*; Ahmed Abdalla, Raiyyan Aftab, Nathan Curtis, Thomas Dudding, Shoura Karar, Rebekah McCullough, Aaron Julius Punnen, Atiqur Rahman, Simon Williams *(Southampton General Hospital, Southampton)*; Dmitri Artioukh, Aloka Suwanna Danwaththa Liyanage, Krishnan Gokul, Karthikeyan Iyengar, Muyed Mohamed, Khushroo Suraliwala, Bianca Wadham, Chamindri Weerasinghe *(Southport and Formby District General Hospital, Southport)*; Muhammad Aleem, Christopher Brown, Russell Cathcart, Olivia Cohen, Sarfraz Jamali, Miklos Kassai, Thomas Walker, Gbolahan Williams *(Jersey General Hospital, St.Helier)*; Rachel Baumber, Rebecca Brinkler, Zara Hayat, Adam Hunt, James Parry *(Royal National Orthopaedic Hospital, Stanmore)*; Samuel Adegbola, Govind Dhillon, Barnaby Farquharson, Shareef Mahdi, Tien Yeoh, Clarence Yeoh *(Lister Hospital, Stevenage)*; Stephen Bromage, Somashree Chatterji, Melissa Condon, Suku George, Smitha George, Claire Hall, Janette Hunt, David Johnson, Vivek Kaushik, Magda Kujawa, Anil Kumar, Muhammad Isfandyar Khan Malik, Fatema Munshi, Sreedevi Nair, Eloka Okoye, Jen Hong Ong, Joanna Rooney, Milan Rudic, Inderpaul Samra, Jamel TaharAissa, Catherine Toksoy, Nimisha Vallabh, Imad Wafaie, Imad Zakieh, Yuhao Zhang *(Stepping Hill Hospital, Stockport)*; Maimoona Afzal, Erminia Albanese, Daisy Evans, Gomathy Gopal, Anthony Jaipersad, Ben-Lawrence Kemah, Isaac Kobe, Ayesha Mahmud, Gourab Misra, Bobby Sachdev, Uzma Sadia, Jessica Spalding *(Royal Stoke University Hospital, Stoke-on-Trent)*; Elizabeth Jones, Amy Lovett *(University Hospitals of North Midlands, Stoke-on-Trent)*; Christin Henein, Jia Y Ng, Dimiter Shinkov *(Sunderland Eye Infirmary, Sunderland)*; Chris Hartley, Amarkumar D Rajgor, Holt Walters *(Sunderland Royal Hospital, Sunderland)*; Alasdair Ball, Holly Digne-Malcolm, Simon Fallis, Mohammed Hamid *(Good Hope Hospital, Sutton Coldfield)*; Hannah Boyd-Carson, Marta D’Auria, Manas Dube, Elizabeth Gemmill, Elisa Lenzi, Dimple Sapre, Arjun Shajpal, Wajiha Tehniyat, Nicholas Watson *(King’s Mill Hospital, Sutton-In-Ashfield)*; Ghazia Ahmed, Sankar Ananth, Daniel Ashworth, Peter Cripps, Anna Davies, Dharminder Dhillon, Jennifer Edmondson, Richard Egan, Rhiannon Harries, Alastair Henry, Madhav Kittur, Godfrey Lau, Zoe Li, Miriam Nyeko-Lacek, Harvey Rich, Ketan Shah, Lydia Tang *(Morriston Hospital Swansea, Swansea)*; Rute Castelhano, Sherwin Ng, Vinay Shah, Paul Stanier, Olivia Wharf, Khine Myat Win *(Great Western Hospital, Swindon)*; Thomas Badenoch, India Cox, Shanice Cox, Catriona Daly, Kaveh Davoudi, Paul Foster, Christian Grieco, Marianne Hollyman, Louise Hunt, Bhavani Kamalakannan, Andrew Kelly, Hamad Khan, Neil Lenus, Hamish Macdonald, Jo Morrison, Ellen Nelissen, Laura Newitt, Hammad Parwaiz, Emma-Tina Segall, George Slade, Andrew Stevenson, Charles Geoffrey Dermot Stewart, Samuel Wenham, Amy Whitworth *(Musgrove Park Hospital, Taunton)*; Rakan Kabariti, Tahir Khaleeq, Pradyumna Naredla, Varkha Rattu, Jae Rhee, Asohanpal Sohanpal, Rosemary Sykes *(Princess Royal Hospital, Telford)*; Cait Bleakley, Joel Bowen, Aubrey Dickason, Ekemini Ekpo, Matthew Sargent, Rupert Scott, Jonathon Sheen, Ellen Thomas, Amrita Virdi *(Torbay and South Devon NHS Trust, Torquay)*; Katherine de Rome, Victoria Russell, Kapil Sahnan *(Hillingdon Hospital, Uxbridge)*; Ahmed Ammar, Ahmed Elsayed, Janahan Sarveswaran *(Pinderfields Hospital, wakefield)*; Nimai Desai, Srinivasan Gangadharan, Fahad Hossain, Ghiath Ismayl, Nikhil Khadabadi, Thomas Moores, Mohamed Saleem Noor Mohamed, Shweta Pawar, Aasim Saleemi, Mark Simmons, Sita Techaboonanake, Muhammad Jawad Zafar *(Walsall Manor Hospital, Walsall)*; Thomas Alexander, Hannah Emerson, Alice Llambias-Maw *(Warrington & Halton Teaching Hospitals NHS Trust, Warrington)*; Khaled Abdelgalel, Verda Amin, Ruvinder Athwal, Anuj Bhatnagar, Vasileios Charalampakis, Leslie Chi Yan Cheung, Muha Hassan, Jonathan Lee, Praveena Mahalingam, Sean Ramcharan, Tin Sein, Shahbaz Zafar *(South Warwickshire NHS Foundation Trust, Warwick)*; Ngozi Anyaugo, Nitya Chandratreya, Jazal El-Qudah, Venkat mukund reddy Galiveeti, Alyazeed Haddadin, Sattam Halaseh, Erfan Massri, Olumide Oyinloye, Ken Philip, Obafemi Wuraola *(Weston General Hospital, Weston-super-Mare)*; Aenone Harper Machin, Molly Jakeman, Li Yenn Yong *(Whiston Hospital, Whiston)*; Timothy Board, Ayman Gabr, Abdulhakim Hassan, Asim Rajpura *(Wrightington, Wigan & Leigh NHS Foundation Trust, Wigan)*; Ketan Agarwal, Sanabel Al-Maghrabi, Jennifer Allan, Joanna Craven, Bryony David, Bala Krishnan, Oliver Lane, Morag McLellan, Simon Powell, Luke Render, Sarah Shamim, Adam Truss, Raman Vinayagam *(Wirral University Teaching Hospital, Wirral)*; Ahmed Dhaif, Fraser Maxwell, Fiona Ross *(Wishaw General Hospital, Wishaw)*; James Blair, Sabri Bleibleh, Govind Singh Chauhan, Varun Dewan, Richard Dias, William Drew, Awais Habeebullah, Lydia Hiddema, Zainab Iftikhar, Jasprit Kaur, Sean Milner, Chinedu Ndegbu, Kishan Rajdev, Jeremy Reid, Nirbhaibir Singh, Osmond Thomas, Raghavan Vidya, Nuha Yassin *(Royal Wolverhampton NHS Trust, Wolverhampton)*; Mas’ud Adewusi, Islam Ahmed, Roudi Bachar, Sin Ting Natalie Cheng, Abigail Duckett, Sara El Falaha, Karim Hassan, Tajnin Mitu, Chun Ooi, Elsie Tan, Megan Walker, Sophie Watson *(Wrexham Maelor Hospital, Wrexham)*; Mohamed Ashiq Mohamed Salim, Malaz Abbakar, Vanessa Badas, Radu Chirvasuta, Joe Drybrough, Alexandra Hamshere, Annabelle Harrison, Rebecca Ireson, Nelson Kamali, Abigail Lau, Sumant Luhana, Luisa MacDonald, Barbara Ribeiro, Alistair Savage, Tanisha Sharma, Paul Swift, Rachel Taute, Alex Wilkins *(York Teaching Hospitals NHS Trust, York)*. |
[truncated: 32,042 more chars]
